# LONP1 targets HMGCS2 to protect mitochondrial function and attenuate chronic kidney disease

**DOI:** 10.15252/emmm.202216581

**Published:** 2023-01-11

**Authors:** Mi Bai, Mengqiu Wu, Mingzhu Jiang, Jia He, Xu Deng, Shuang Xu, Jiaojiao Fan, Mengqiu Miao, Ting Wang, Yuting Li, Xiaowen Yu, Lin Wang, Yue Zhang, Songming Huang, Li Yang, Zhanjun Jia, Aihua Zhang

**Affiliations:** ^1^ Department of Nephrology, State Key Laboratory of Reproductive Medicine Children's Hospital of Nanjing Medical University Nanjing China; ^2^ Jiangsu Key Laboratory of Pediatrics Nanjing Medical University Nanjing China; ^3^ Nanjing Key Laboratory of Pediatrics Children's Hospital of Nanjing Medical University Nanjing China; ^4^ Key Laboratory of Molecular Pharmacology and Drug Evaluation Yantai University Yantai China; ^5^ Renal Division Peking University First Hospital Beijing China

**Keywords:** chronic kidney disease, HMGCS2, LONP1, mitochondrial dysfunction, Organelles, Urogenital System

## Abstract

Mitochondria comprise the central metabolic hub of cells and their imbalance plays a pathogenic role in chronic kidney disease (CKD). Here, we studied Lon protease 1 (LONP1), a major mitochondrial protease, as its role in CKD pathogenesis is unclear. LONP1 expression was decreased in human patients and mice with CKD, and tubular‐specific *Lonp1* overexpression mitigated renal injury and mitochondrial dysfunction in two different models of CKD, but these outcomes were aggravated by Lonp1 deletion. These results were confirmed in renal tubular epithelial cells *in vitro*. Mechanistically, LONP1 downregulation caused mitochondrial accumulation of the LONP1 substrate, 3‐hydroxy‐3‐methylglutaryl‐CoA synthase 2 (HMGCS2), which disrupted mitochondrial function and further accelerated CKD progression. Finally, computer‐aided virtual screening was performed, which identified a novel LONP1 activator. Pharmacologically, the LONP1 activator attenuated renal fibrosis and mitochondrial dysfunction. Collectively, these results imply that LONP1 is a promising therapeutic target for treating CKD.

## Graphical abstract

Activation of the mitochondrial protease Lon protease 1 (LONP1) attenuates mitochondrial dysfunction and renal fibrosis in chronic kidney disease (CKD) mice, suggesting that targeting LONP1 might represent a novel therapeutic strategy for CKD.

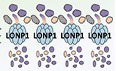

**The paper explained**

**Problem**

Chronic kidney disease (CKD) is a chronic renal structural and functional disorder caused by various diseases and conditions. The incidence of CKD worldwide is more than 10%, and there is a lack of effective treatment. A considerable proportion of patients progress to end‐stage renal disease (ESRD). Renal fibrosis is a common pathology leading to ESRD and it is directly related to the prognosis of renal function. Kidney fibrosis progresses gradually until it is necessary to rely on renal replacement therapy to maintain life. Therefore, it is urgent to elucidate the molecular mechanism of the occurrence and development of renal fibrosis and to find effective intervention targets to delay its progression.

**Results**

Here, we identified LONP1/HMGCS2 signaling in proximal tubular cells as a therapeutic target for retarding kidney fibrosis. Tubular‐specific *Lonp1* overexpression mitigated renal injury and mitochondrial dysfunction in two different models of CKD, and these outcomes were aggravated by Lonp1 deletion. Mechanistically, LONP1 downregulation caused mitochondrial accumulation of the LONP1 substrate, HMGCS2, which disrupted mitochondrial function and further accelerated CKD progression. More importantly, we identified a novel LONP1 activator, which may be a promising therapeutic for treating CKD.

**Impact**

Mitochondrial dysfunction in proximal tubular cells contributes to CKD progression, while the specific therapeutic targets to improve mitochondrial function in proximal tubular cells are still limited. These results not only demonstrate the important role of the LONP1/HMGCS2 signaling pathway in delaying renal fibrosis, but also suggest that the pathway may be a therapeutic target for CKD by activating LONP1.

## Introduction

Chronic kidney disease (CKD), the end stage of various kidney diseases, has become a worldwide health problem with a rapidly increasing prevalence, high morbidity, and economic burden (Zhang *et al*, 2012; Webster *et al*, 2017). Renal tubular‐interstitial fibrosis is a common pathophysiological pathway involved in the development of multiple CKDs that lead to end‐stage renal disease (ESRD). Proximal tubular cells, which are rich in mitochondria and require high energy to meet the tremendous energy demands of tubular reabsorption and secretion, are particularly sensitive to various insults such as hypoxia, oxidative stress, and toxins (Chevalier, 2016). Several reports have shown that the impairment of proximal tubular cells can initiate and promote an inflammatory and profibrogenic response, activate mesenchymal fibroblasts, and drive the production of many extracellular matrix proteins, which ultimately results in tubulointerstitial fibrosis and a continuous loss of renal function (Liu, 2011; Leung *et al*, 2013; Meng *et al*, 2014; Liu *et al*, 2020). Hence, identifying novel endogenous kidney protectants and specific mechanisms by which to mitigate proximal tubular cell injury can provide insights into new treatment strategies for CKD.

Proximal tubular cells are highly dependent on mitochondria to maintain their cellular functions and viability. As the energy factory of cells, mitochondria maintain cellular homeostasis through a complex and sophisticated monitoring system, which includes the timely degradation of damaged misfolded proteins and the elimination of mutant mitochondrial DNA (mtDNA) and free radicals (Jiang *et al*, 2020). Data from previous studies showed that mitochondrial dysfunction is not only an early event in kidney injury, but also contributes to CKD development and progression, and maintaining normal mitochondrial function can effectively alleviate kidney injury (Yuan *et al*, 2012; Bai *et al*, 2019; Miguel *et al*, 2021). Thus, looking for intervention targets to maintain the normal functions of mitochondria and restoring the renal tubular mitochondrial function in CKD may be an important research direction for preventing and treating CKD.

The Lon protease 1 (LONP1) is a highly conserved ATP‐dependent protease that ensures mitochondrial proteostasis and regulates adaptive responses to cellular stress (Bota & Davies, 2001; Lu *et al*, 2003; Bahat *et al*, 2015). Previous findings have shown that LONP1 upregulation helps lung fibroblast cells adapt to acute stress and is important for preserving normal cell viability (Ngo *et al*, 2011) and overcoming the hypoxic, metabolic, and proteotoxic stress associated with the oncogenic transformation of tumor cells (Lu, 2017). In addition, LONP1 can protect cardiomyocytes from injury due to ischemia/reperfusion (Venkatesh *et al*, 2019). Previously, we reported that reduced LONP1 expression in podocytes contributed to the pathogenesis of podocytopathy (Gong *et al*, 2021). However, no reports have demonstrated the involvement of LONP1 in renal fibrosis.

LONP1 dysfunction directly leads to the failure of mitochondrial protein degradation, and the accumulation of abnormal mitochondrial proteins leads to a series of cellular and tissue injuries. In this study, we found that hydroxymethyl glutaryl coenzyme A synthase (HMGCS2) might be an LONP1 substrate. HMGCS2 (which belongs to the HMG‐CoA synthase family) is a mitochondrial enzyme that catalyzes the first reaction of ketogenesis, a metabolic pathway that provides lipid‐derived energy for various organs during times of carbohydrate deprivation, such as fasting (Geisler *et al*, 2019). HMGCS2 has been suggested to play important roles in diabetes, tumor, Alzheimer's disease, and intestinal cell differentiation (Wan *et al*, 2018, 2019; Cheng *et al*, 2019; Kim *et al*, 2019; Zou *et al*, 2019; Wang *et al*, 2019b). Recent data indicated that high HMGCS2 expression caused reactive oxygen species (ROS) accumulation and loss of the mitochondrial membrane potential (MMP), which then induced diabetic cardiomyopathy (Wang *et al*, 2020a). However, the exact role of HMGCS2 in renal fibrosis remains unclear.

Here, we identified LONP1 as an endogenous mitochondrial regulator in renal tubular cells under CKD conditions, in both rodents and humans. Tubule‐specific *Lonp1* overexpression mitigated mitochondrial dysfunction and markedly increased tubular injury and renal fibrosis in two mouse models of CKD (one involving unilateral ureteral obstruction [UUO] and one involving 5/6 nephrectomy [5/6Nx]), but these outcomes were aggravated by tubule‐specific *Lonp1* abrogation. These results were confirmed *in vitro*, using transforming growth factor (TGF)‐β1‐treated renal tubular epithelial cells. Mechanically, we found that HMGCS2 is a possible substrate for LONP1 and that mitochondrial HMGCS2 accumulation disrupted mitochondrial function and further aggravated CKD. We validated these results by demonstrating that the pharmacological activator of LONP1 could attenuate renal fibrosis and mitochondrial function. Therefore, our findings provide a rationale for designing targeted LONP1 activators as therapeutic agents against CKD.

## Results

### LONP1 expression in fibrotic kidneys of CKD patients and UUO mice

**Figure 1:**
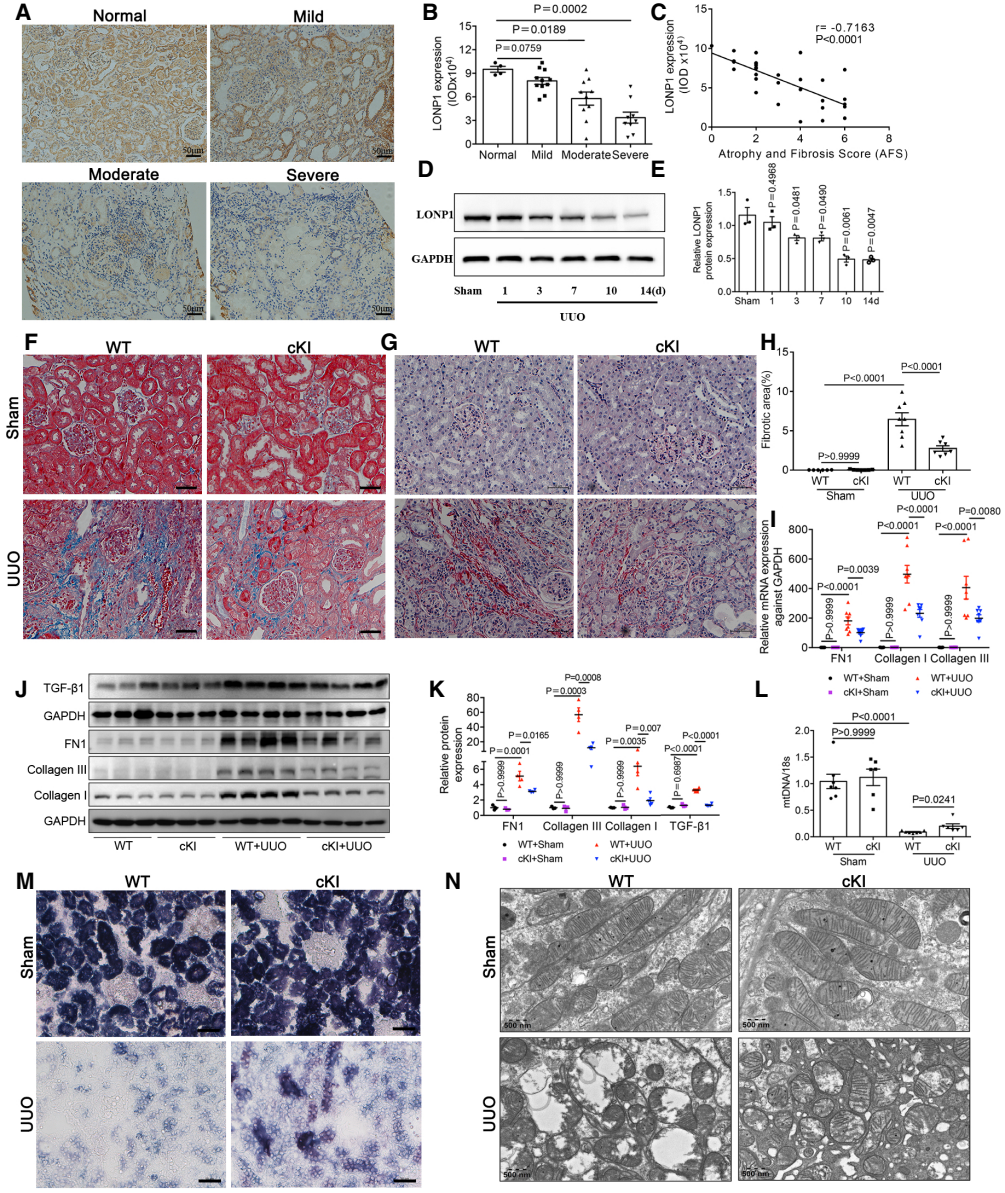
LONP1 is down‐regulated in the kidneys of CKD patients and mice and proximal tubular‐specific overexpression of *Lonp1* alleviates renal injury and mitochondrial dysfunction in UUO model AImmunohistochemical analysis of LONP1 expression in CKD children with mild (*n* = 11), moderate (*n* = 10), or severe fibrosis (*n* = 9). *N* = 4 in Normal group. Scale bar: 50 μm.BImmunohistochemical semi‐quantitative IOD analysis of LONP1.CPearson correlation analysis of LONP1 and atrophy and fibrosis score (AFS) in renal biopsy specimens.DWestern blot analysis for the expression of LONP1 in UUO models at different time points.EDensitometric analysis for the expression of LONP1 (*n* = 3, biological replicates).FDeposition of total fibrosis in kidney tissues was determined by Masson's trichrome staining. Scale bar: 50 μm.GSirius red staining in WT and cKI mice after UUO. Scale bar: 50 μm.HFibrotic area statistics of Sirius red staining in WT and cKI mice after UUO (*n* = 6 in WT+Sham group, *n* = 8 in cKI+Sham or WT+UUO group, *n* = 7 in cKI+UUO group, biological replicates).IqRT‐PCR analysis of FN1, Collagen I and Collagen III in WT and cKI mice after UUO (*n* = 7 in WT+Sham group, *n* = 8 in the other three groups, biological replicates).J, KWestern blot and densitometric analysis for the expression of FN1, Collagen I, Collagen III and TGF‐β1 (monomer) in WT and cKI mice after UUO (*n* = 3 or 4, biological replicates).LqRT‐PCR analysis of mtDNA expression in WT and cKI mice after UUO (*n* = 6 or 7, biological replicates).MSuccinate dehydrogenase (SDH) staining in WT and cKI mice after UUO. Scale bar: 50 μm.NTransmission electron microscopy images of the mitochondria in tubular cells in WT and cKI mice after UUO. Scale bar: 500 nm. Data information: In (B, E), data are presented as mean ± SEM. Student's *t*‐test. In (H–L), data are presented as mean ± SEM. One‐way ANOVA. Source data are available online for this figure.

**Figure EV1:**
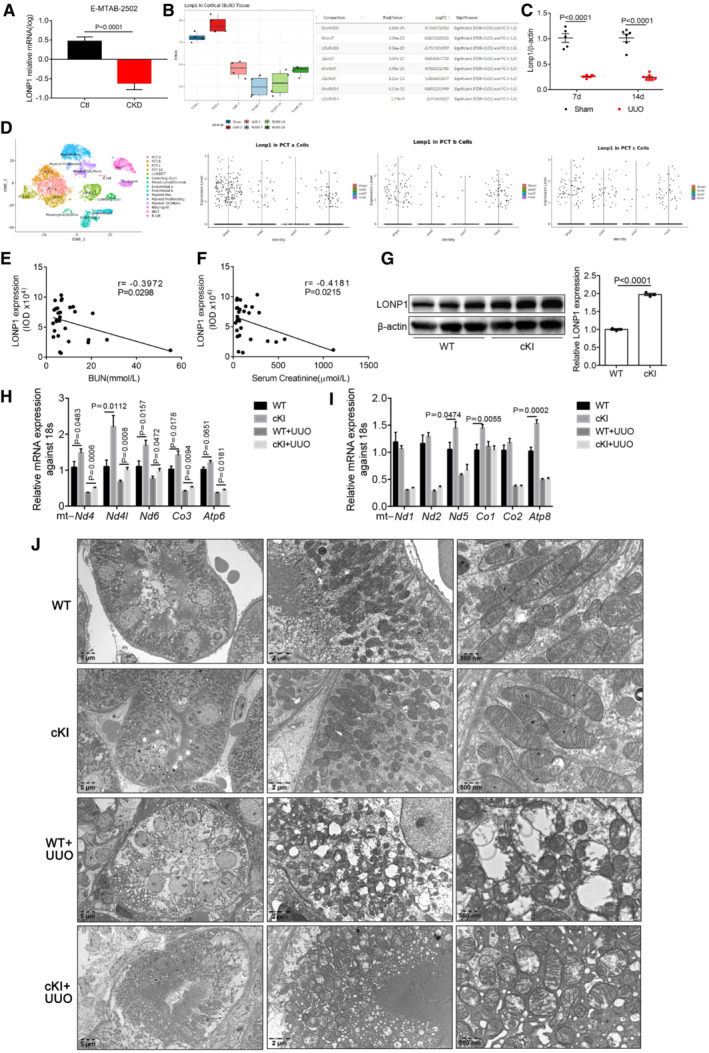
LONP1 was down‐regulated in CKD kidneys based on the analysis of online datasets and proximal tubular‐specific overexpression of *Lonp1* alleviated the reduction of mitochondrial genes expression and protected mitochondrial morphology in UUO model A
*Lonp1* expression from online human RNA sequencing data (Data ref: Yi‐An, 2014). *N* = 20 in Ctl group an *n* = 19 in CKD group (biological replicates).B
*Lonp1* expression from online mouse UUO model RNA sequencing data (Data ref: Denby *et al*, 2020). *N* = 4 in each group (biological replicates).CThe mRNA expression of *Lonp1* in our UUO models (*n* = 5–7, biological replicates).D
*Lonp1* expression from kidney single cell datasets Gene Atlas of Reversible Unilateral Ureteric Obstruction Model (http://www.ruuo‐kidney‐gene‐atlas.com/).E, FPearson correlation analysis of LONP1 and BUN and Serum Creatinine in CKD patients (*n* = 30).GWestern blot and densitometric analysis for the expression of LONP1 of proximal tubular cells isolated from WT and cKI mice (*n* = 3, biological replicates).H, IqRT‐PCR analysis of mitochondrial genes in WT and cKI mice after UUO (*n* = 8 in each group, biological replicates).JTransmission electron microscopy images of intact renal tubule cells and mitochondria in WT and cKI mice after UUO. Scale bar: 5 μm, 2 μm and 500 nm. Data information: Data are presented as mean ± SEM. Student's *t*‐test. In the boxplot of B, the central band represents the median line; the boxes represent range between 25 and 75%; the whiskers represent range within the 1.5 IQR (Inter‐Quartile Range). Source data are available online for this figure.

To examine the association of LONP1 with CKD, we performed immunohistochemical (IHC) staining with biopsied kidney tissues from 30 patients with CKD. Compared with the richness of signal in control human kidneys, significant downregulation of LONP1 was observed in patient kidneys, primarily in the renal tubular cells (Fig 1A and B). The mRNA levels of CKD patients also decreased in an online dataset (Kang *et al*, 2015) (Fig EV1A). More importantly, the level of LONP1 expression negatively correlated with the degree of kidney fibrosis, as assessed by the atrophy and fibrosis score (AFS; *r* = −0.716, *P* < 0.001; Fig 1C), as well as negatively correlated with BUN and Scr in CKD patients (Fig EV1E and F). Then, we studied UUO models at different time points and found that LONP1 expression decreased in a time‐dependent manner (Figs 1D and E, and EV1C). We verified the reduction of *Lonp1* mRNA levels in UUO models using an online database (Conway *et al*, 2020) (Fig EV1B and D).

### Tubule‐specific *Lonp1* overexpression or deletion attenuated or aggravated CKD induced by UUO

To ascertain the role of LONP1 in CKD, we generated proximal tubule *Lonp1* conditional knock‐in mice (cKI). Compared with wild‐type (WT) mice, the expression of LONP1 in renal tubule cells of cKI mice was approximately doubled (Fig EV1G). With the UUO model, *Lonp1*‐specific overexpression in renal proximal tubules significantly attenuated tubular brush border loss, tubule atrophy, cellular infiltration, and tubulointerstitial fibrosis in the kidneys determined by Masson's trichrome and Sirius red staining (Fig 1F–H). The production of extracellular matrix components (Collagen I, Collagen III, and fibronectin 1 [FN1]) was lower in cKI mice (Fig 1I–K). The profibrotic factor TGF‐β1 also decreased after *Lonp1* overexpression (Fig 1J and K). In addition, mitochondrial dysfunction was ameliorated significantly, as evidenced by restored mitochondrial genes expression (Fig EV1H and I), mtDNA copy numbers (Fig 1L), succinate dehydrogenase (SDH) activity (Fig 1M) and mitochondrial morphology (including mitochondrial swelling and disorganized fragmented cristae in the renal tubular cells of the UUO model) (Figs 1N and EV1J).

**Figure 2:**
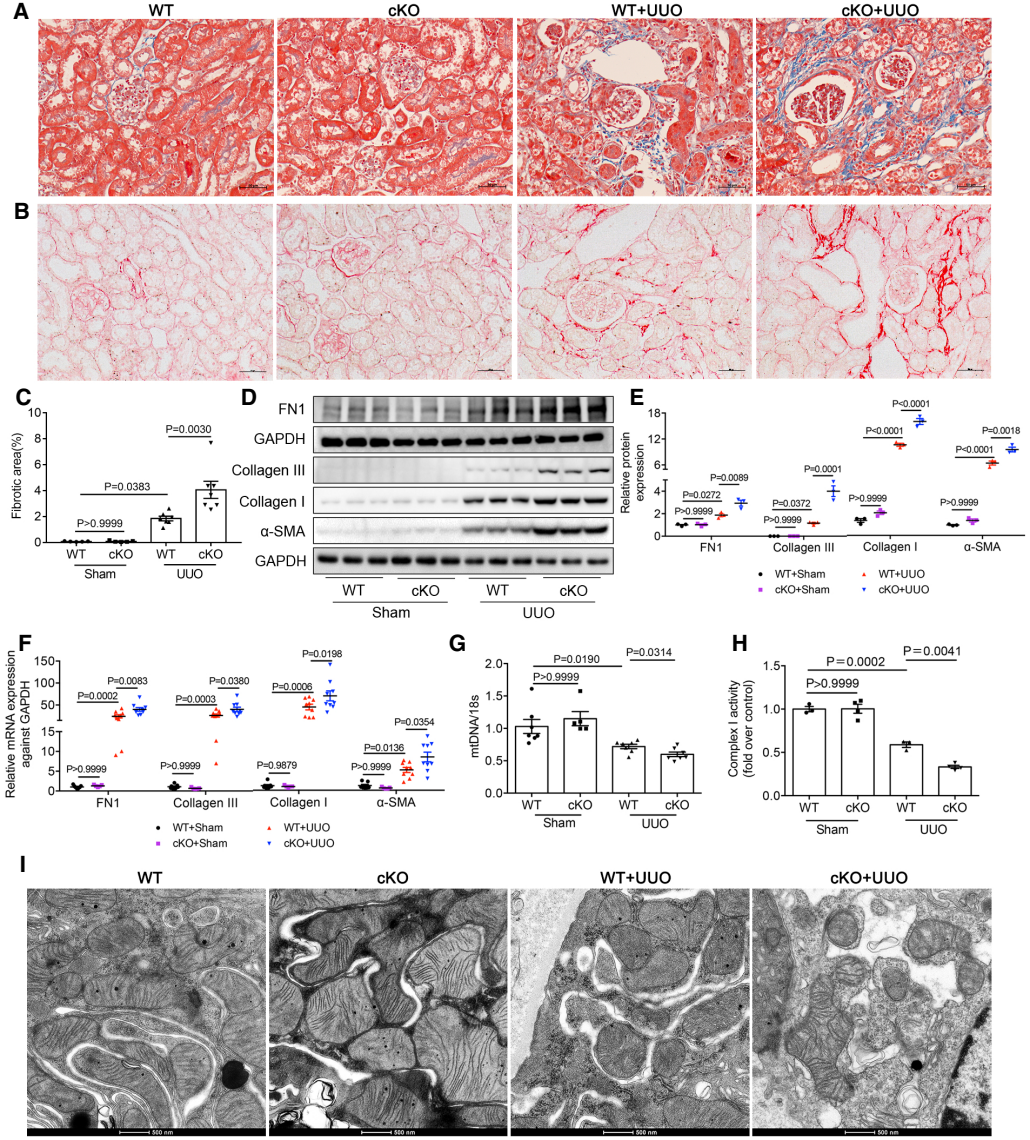
Proximal tubular‐specific deletion of *Lonp1* aggravates renal injury and mitochondrial dysfunction in UUO model ADeposition of total fibrosis in kidney tissues was determined by Masson's trichrome staining. Scale bar: 50 μm.BSirius red staining in WT and cKO mice after UUO. Scale bar: 50 μm.CFibrotic area statistics of Sirius red staining in WT and cKO mice after UUO. *N* = 5 in Sham groups. *N* = 7 in UUO groups, biological replicates.D, EWestern blot and densitometric analysis for the expression of FN1, Collagen I, Collagen III and α‐SMA in WT and cKO mice after UUO (*n* = 3, biological replicates).FqRT‐PCR analysis of FN1, Collagen I, Collagen III and α‐SMA in WT and cKO mice after UUO (*n* = 7 or 5 in Sham groups, *n* = 9 in UUO groups, biological replicates).GqRT‐PCR analysis of mtDNA expression in WT and cKO mice after UUO (*n* = 7 or 5 in Sham groups, *n* = 7 in UUO groups, biological replicates).HMitochondrial respiratory chain complex I enzymatic activity in WT and cKO mice after UUO (*n* = 4, biological replicates).ITransmission electron microscopy images of the mitochondria in tubular cells in WT and cKO mice after UUO. Scale bar: 500 nm. Data information: Data are presented as mean ± SEM. One‐way ANOVA. Source data are available online for this figure.

**Figure EV2:**
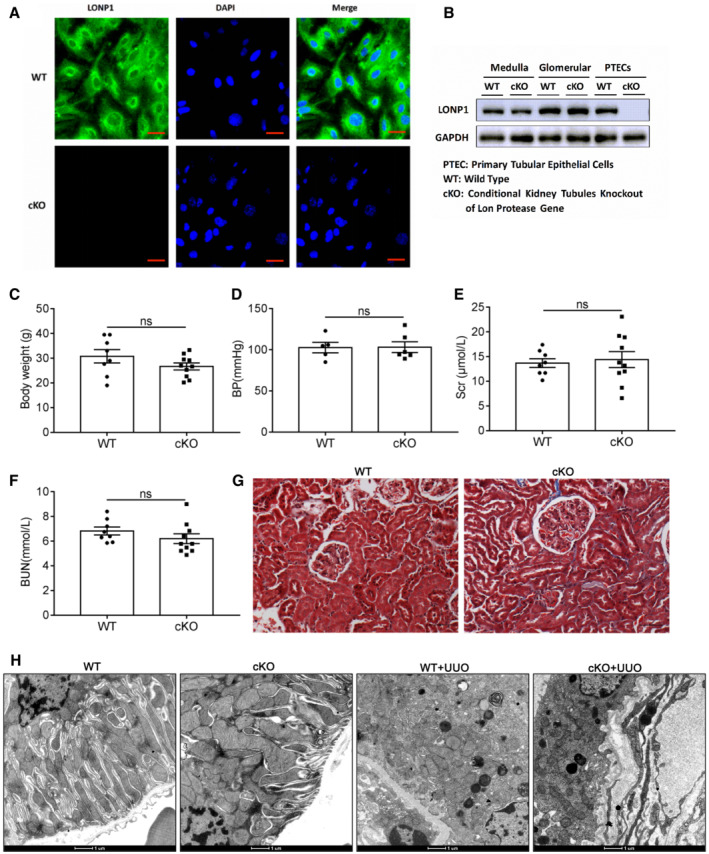
Validation of proximal tubular‐specific deletion of *Lonp1* (cKO), physiological indexes of aged cKO mice, and mitochondrial morphology in UUO model ALONP1 immunofluorescence staining of primary renal tubular epithelial cells (PTECs) isolated from WT and cKO mice. Scale bar: 20 μm.BWestern blot analysis for the expression of LONP1 in medulla, glomerular and PTECs.C–GBody weight (*n* = 8 or 10), blood pressure (BP, *n* = 5 or 6), serum creatinine (Scr, *n* = 8 or 10), blood urea nitrogen (BUN, *n* = 8 or 10) and Masson's trichrome staining in elderly WT and cKO mice (18 months). Scale bar: 50 μm.HTransmission electron microscopy images of the mitochondria in tubular cells in WT and cKO mice after UUO model. Scale bar: 1 μm. Data information: Data are presented as mean ± SEM. Student's *t*‐test. Source data are available online for this figure.

Next, we generated proximal tubule *Lonp1* conditional knockout (cKO) mice. Compared with WT mice, LONP1 was almost not expressed in primary proximal tubule cells extracted and cultured from cKO mice (Fig EV2A and B). In contrast to our previous report showing that podocyte‐specific *Lonp1* knockout was lethal, mice with tubule‐specific *Lonp1* knockout grew and developed normally. Even in elderly mice (18 months), we observed normal body weights, blood pressures (BPs), serum creatinine (Scr) levels, blood urea nitrogen (BUN) levels, and physiological conditions, when compared with the WT group (Fig EV2C–G). We then created UUO model in the cKO and WT mice. Masson's trichrome and Sirius red staining indicated that *Lonp1* deficiency in renal proximal tubules correlated with expanded pathological damage, resulting in proximal tubule brush border loss and tubulointerstitial fibrosis in the kidneys (Fig 2A and B). The fibrotic area also confirmed deteriorated renal lesions in *Lonp1*‐cKO UUO mice (Fig 2C). Compared with WT UUO mice, *Lonp1*‐cKO UUO mice displayed increased deposition of extracellular matrix components (Collagen I, Collagen III, and FN1) and increased alpha smooth muscle actin (α‐SMA) protein (Fig 2D and E) and mRNA (Fig 2F) expression. We also checked the status of the renal mitochondrial functions in the UUO model. As expected, the mtDNA copy numbers (Fig 2G) and Complex I activity were markedly reduced by the specific *Lonp1* deficiency in the renal proximal tubular cells (Fig 2H). Transmission electron microscopy‐based examination of the mouse kidney sections revealed that the mitochondrial morphology deteriorated markedly in the *Lonp1*‐cKO mice (Figs 2I and EV2H).

Together, these data indicate that tubule‐specific overexpression or deletion of *Lonp1* alleviated or aggravated UUO‐induced renal fibrosis and mitochondrial dysfunction.

### Specific renal tubular overexpression or deficiency of *Lonp1* alleviated or aggravated CKD conditions induced by 5/6Nx model

**Figure 3:**
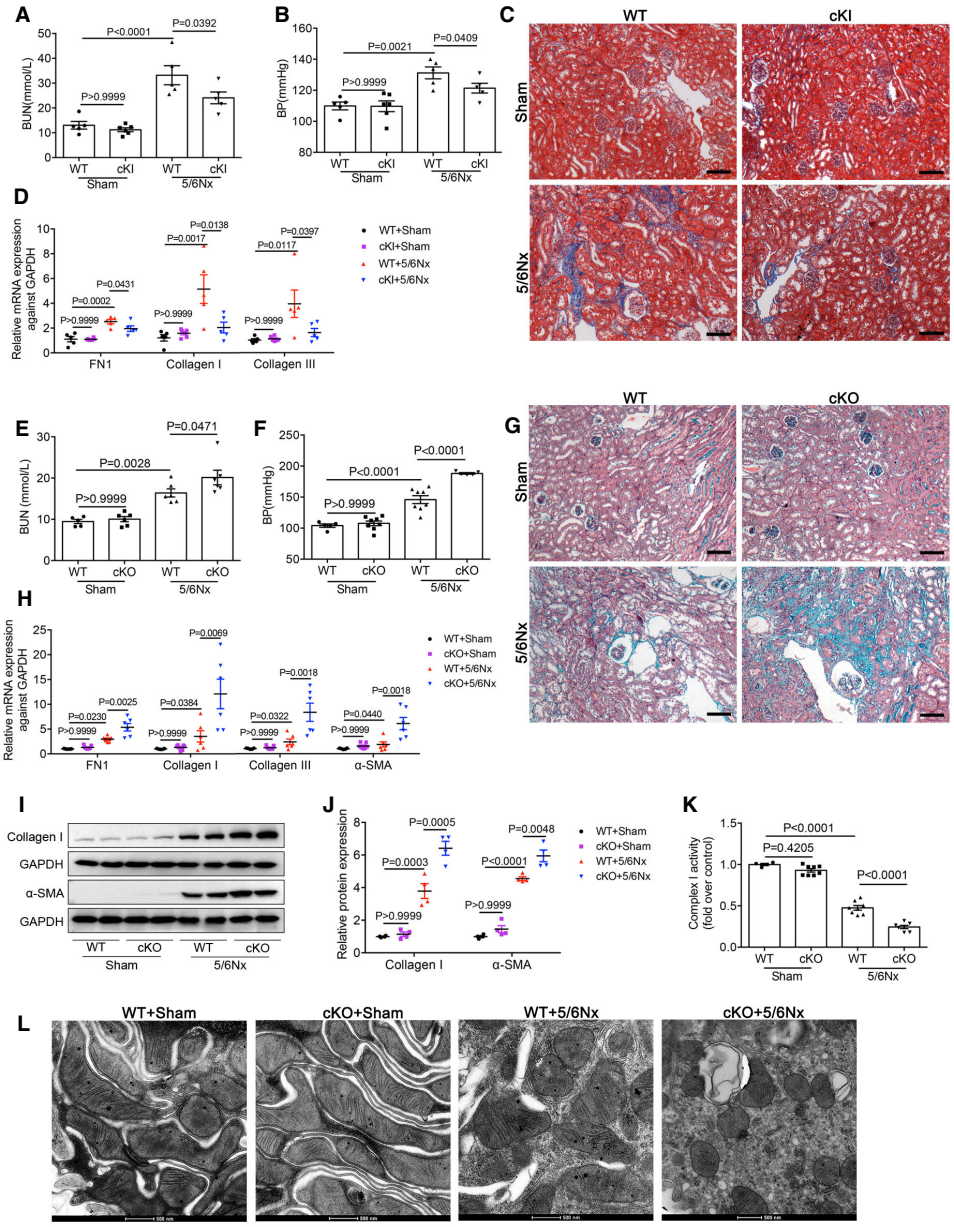
Proximal tubular‐specific overexpression or deletion of *Lonp1* mitigated or aggravated renal injury and mitochondrial dysfunction in 5/6Nx model AAnalysis of blood urea nitrogen (BUN) after 5/6Nx model in WT and cKI mice (*n* = 6 in cKI+Sham group, *n* = 5 in the other three groups, biological replicates).BSystolic blood pressure was measured by the computerized tail‐cuff system (*n* = 6 in cKI+Sham group, *n* = 5 in the other three groups, biological replicates).CMasson's trichrome staining of WT and cKI mice after 5/6Nx. Scale bar: 100 μm.DqRT‐PCR analysis of FN1, Collagen I and Collagen III in WT and cKI mice after 5/6Nx (*n* = 6 in cKI+Sham group, *n* = 5 in the other three groups, biological replicates).EAnalysis of blood urea nitrogen (BUN) after 5/6Nx model in WT and cKO mice (*n* = 5 in WT+Sham group, *n* = 6 in the other three groups, biological replicates).FSystolic blood pressure in WT and cKO mice (*n* = 5 in WT+Sham or cKO+5/6Nx group, *n* = 8 in cKO+Sham or WT+5/6Nx group, biological replicates).GMasson's trichrome staining of WT and cKO mice after 5/6Nx. Scale bar: 100 μm.HqRT‐PCR analysis of FN1, Collagen I, Collagen III and α‐SMA in WT and cKO mice after 5/6Nx (*n* = 5 in WT+Sham group, *n* = 7 in cKO+Sham group, *n* = 6 in 5/6Nx groups, biological replicates).I, JWestern blot and densitometric analysis for the expression of Collagen I and α‐SMA in WT and cKO mice after 5/6Nx (*n* = 4, biological replicates).KMitochondrial respiratory chain complex I enzymatic activity in WT and cKO mice after 5/6Nx (*n* = 4–8, biological replicates).LTransmission electron microscopy images of the mitochondria in tubular cells of WT and cKO mice after 5/6Nx. Scale bar: 500 nm. Data information: Data are presented as mean ± SEM. One‐way ANOVA. Source data are available online for this figure.

**Figure EV3:**
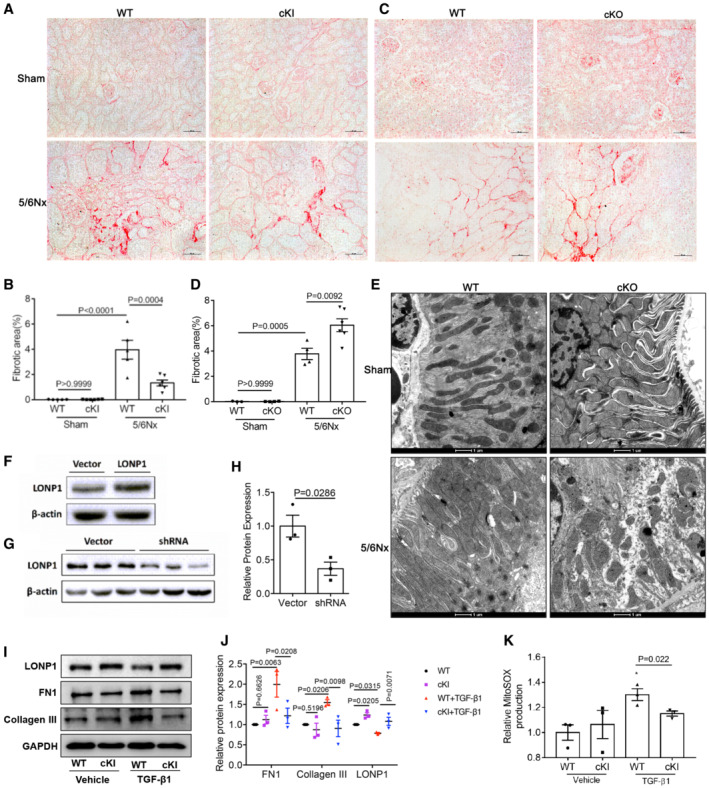
Proximal tubular‐specific overexpression or deletion of *Lonp1* mitigated or aggravated renal fibrosis and mitochondrial morphology damage in 5/6Nx model and the protective effect of LONP1 in primary proximal tubular cells A, BSirius red staining and fibrotic area statistics of WT and cKI mice in 5/6Nx model. Scale bar: 50 μm (*n* = 5 in WT groups, *n* = 6 in cKI groups, biological replicates).C, DSirius red staining and fibrotic area statistics of WT and cKO mice in 5/6Nx model. Scale bar: 50 μM (*n* = 3–6, biological replicates).ETransmission electron microscopy images of the mitochondria in tubular cells in WT and cKO mice after 5/6Nx model. Scale bar: 1 μm.FWestern blot and densitometric analysis for the expression of LONP1 after transfected with vector or *Lonp1* plasmid.G, HWestern blot and densitometric analysis for the expression of LONP1 after transfected with vector or Lonp1 shRNA (*n* = 3, biological replicates).I, JThe proximal tubular cells were isolated from WT and Lonp1 cKI mice. The primary cells were treated with TGF‐β1 for 24 h. Western blot and densitometric analysis for the expression of LONP1, FN1 and Collagen III (*n* = 3 in each group).KQuantification of mitochondrial ROS production (*n* = 3, biological replicates). The primary cells were treated with TGF‐β1 for 4 h. Data information: In (H), data are presented as mean ± SEM. Student's *t*‐test. In (B, D, J, K), data are presented as mean ± SEM. One‐way ANOVA. Source data are available online for this figure.

To further identity the effects of LONP1 in CKD, we next established a 5/6Nx mouse model in cKI and cKO mice. As shown in Fig 3A and B, we found significantly decreased BUN and systolic BP levels in cKI 5/6Nx mice, when compared with those in WT 5/6Nx mice. Masson's trichrome (Fig 3C) and Sirius red staining (Fig EV3A and B) and quantitative reverse transcriptase‐polymerase chain reaction (qRT‐PCR) analysis revealed that kidney fibrosis induced by 5/6Nx was repressed in cKI mice, compared with that in the WT group (Fig 3D). Conversely, we observed significantly higher BUN and systolic BP levels in cKO 5/6Nx mice than in WT 5/6Nx mice (Fig 3E and F). Masson's trichrome (Fig 3G) and Sirius red staining (Fig EV3C and D) confirmed that *Lonp1* deficiency in the renal proximal tubules increased the pathological damage, as cKO 5/6Nx mice exhibited more severe renal lesions and higher fibrotic area than WT 5/6Nx mice. We also demonstrated that specifically knocking out *Lonp1* in proximal tubules also promoted the deposition of extracellular matrix components (FN1, Collagen I and Collagen III) and α‐SMA expression (Fig 3H–J). In line with this discovery in UUO mice, *Lonp1* deficiency in renal proximal tubules showed further reduced Complex I activity (Fig 3K), more serious morphological damage to the mitochondria (Figs 3L and EV3E) in the remnant kidney, when compared with those parameters in WT 5/6Nx mice.

### LONP1 suppressed TGF‐β1‐Induced fibrotic responses and mitochondrial dysfunction in HK2 cells

**Figure 4:**
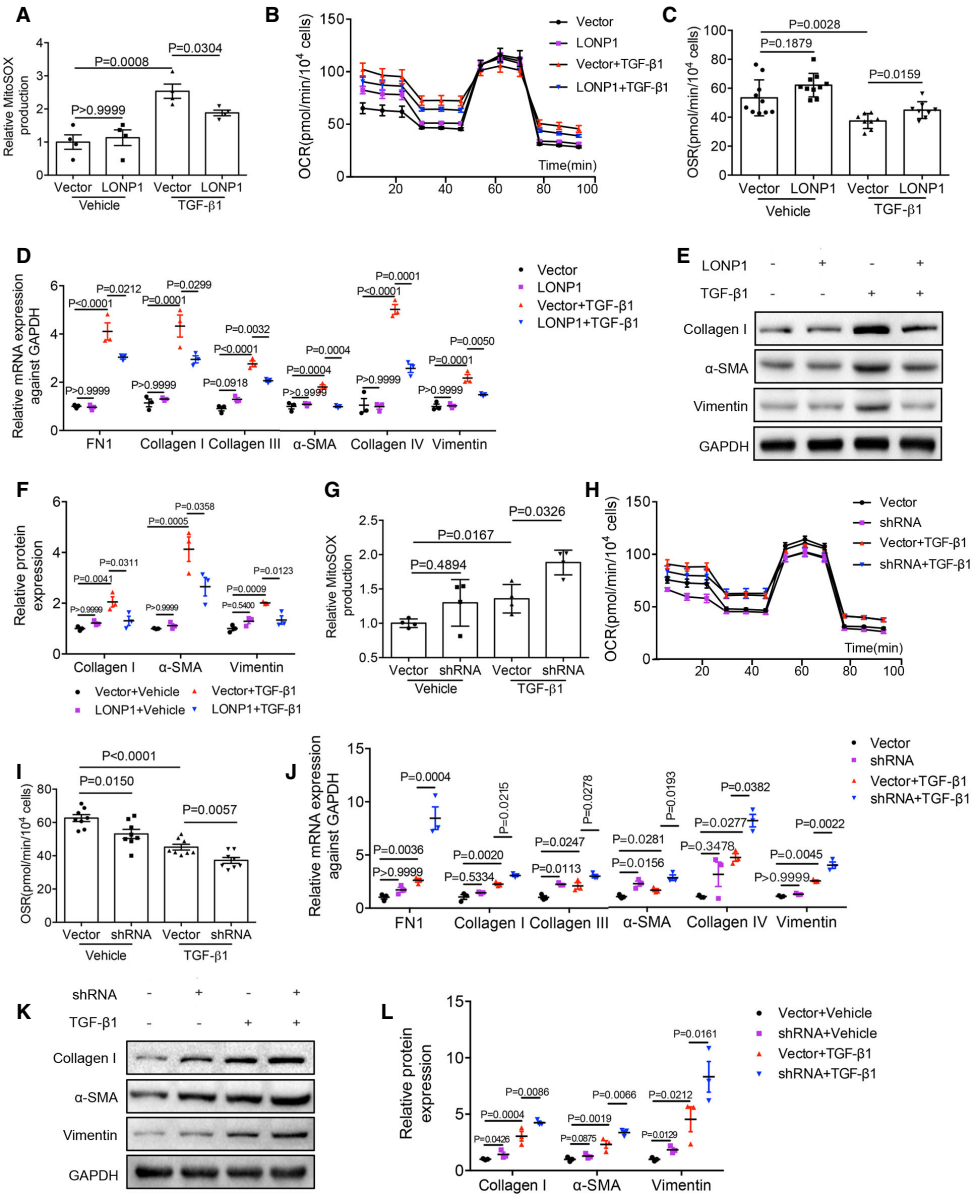
LONP1 attenuated TGF‐β1‐induced mitochondrial dysfunction and fibrotic response in HK2 cells AQuantification of mitochondrial ROS production (*n* = 4, biological replicates).B, CMeasurement of oxygen consumption rate (OCR) using an XF96 Extracellular Flux Analyzer. OSR, spare respiratory capacity (*n* = 8–10 in each group, biological replicates).DqRT‐PCR analysis of FN1, Collagen I, Collagen III, Collagen IV, α‐SMA and Vimentin (*n* = 3, biological replicates).E, FWestern blot and densitometric analysis for the expression of Collagen I, α‐SMA, and Vimentin (*n* = 3, two independent experiments were carried out).GQuantification of mitochondrial ROS production (*n* = 4, biological replicates).H, IMeasurement of OCR and OSR using an XF96 Extracellular Flux Analyzer (*n* = 8 or 10 in each group, biological replicates).JqRT‐PCR analysis of FN1, Collagen I, Collagen III, Collagen IV, α‐SMA and Vimentin (*n* = 3, biological replicates).K, LWestern blot and densitometric analysis for the expression of Collagen I, α‐SMA, and Vimentin (*n* = 3, two independent experiments were carried out). Data information: In (A–F), HK2 cells were transfected with vector or *Lonp1* plasmid, and then treated with TGF‐β1 (10 ng/ml) for 24 h. In (G–L), HK2 cells were transfected with vector or *Lonp1* shRNA, and then treated with TGF‐β1 (10 ng/ml) for 24 h. Data are presented as mean ± SEM. One‐way ANOVA. Source data are available online for this figure.

To further examine the role of LONP1 in renal tubular cells, we transfected HK2 cells with a *Lonp1*‐overexpression plasmid or the vector (Fig EV3F) and then treated the cells with TGF‐β1. Considering that LONP1 is an important mitochondrial protease, we first examined indicators related to mitochondrial functions. Compared with the control group, HK2 cells overexpressing LONP1 show attenuation in the increase in mitochondrial ROS production induced by TGF‐β1 (Fig 4A). Overexpressing LONP1 in HK2 cells significantly alleviated the spare respiratory capacity (OSR) (Fig 4B and C). As shown by our qRT‐PCR (Fig 4D) and western blot (Fig 4E and F) data, LONP1 overexpression significantly inhibited the synthesis of Collagen I, Collagen III, Collagen IV, and FN1, and the upregulation of α‐SMA and Vimentin, induced by TGF‐β1. These results were verified in primary proximal tubular cells isolated from WT and LONP1 cKI mice (Fig EV3I–K).

Next, we transfected HK2 cells with a vector encoding *Lonp1* short‐hairpin RNA or a control vector (Fig EV3G and H) and then treated them with a vector encoding TGF‐β1. LONP1 inhibition aggravated mitochondrial dysfunction. We measured the oxygen‐consumption rate using a Seahorse XF‐96 Extracellular Flux Analyzer and found that the OSR decreased after LONP1 was downregulated. Moreover, the inhibition of LONP1 further aggravated TGF‐β1‐induced mitochondrial ROS production and the decreased OSR (Fig 4G–I). In addition, qRT‐PCR (Fig 4J) and western blots (Fig 4K and L) results showed that LONP1 inhibition significantly promoted Collagen I, Collagen III, Collagen IV, α‐SMA, and Vimentin synthesis, and further aggravated the TGF‐β1‐induced increase of the above fibrotic indicators.

Collectively, these data reveal that LONP1 played a substantial role in preventing TGF‐β1‐induced mitochondrial dysfunction, extracellular matrix production, and the cell‐phenotype transition, indicating LONP1 acted as a protective factor against renal fibrogenesis.

### LONP1 may play a renal‐protective role by degrading HMGCS2

**Figure 5:**
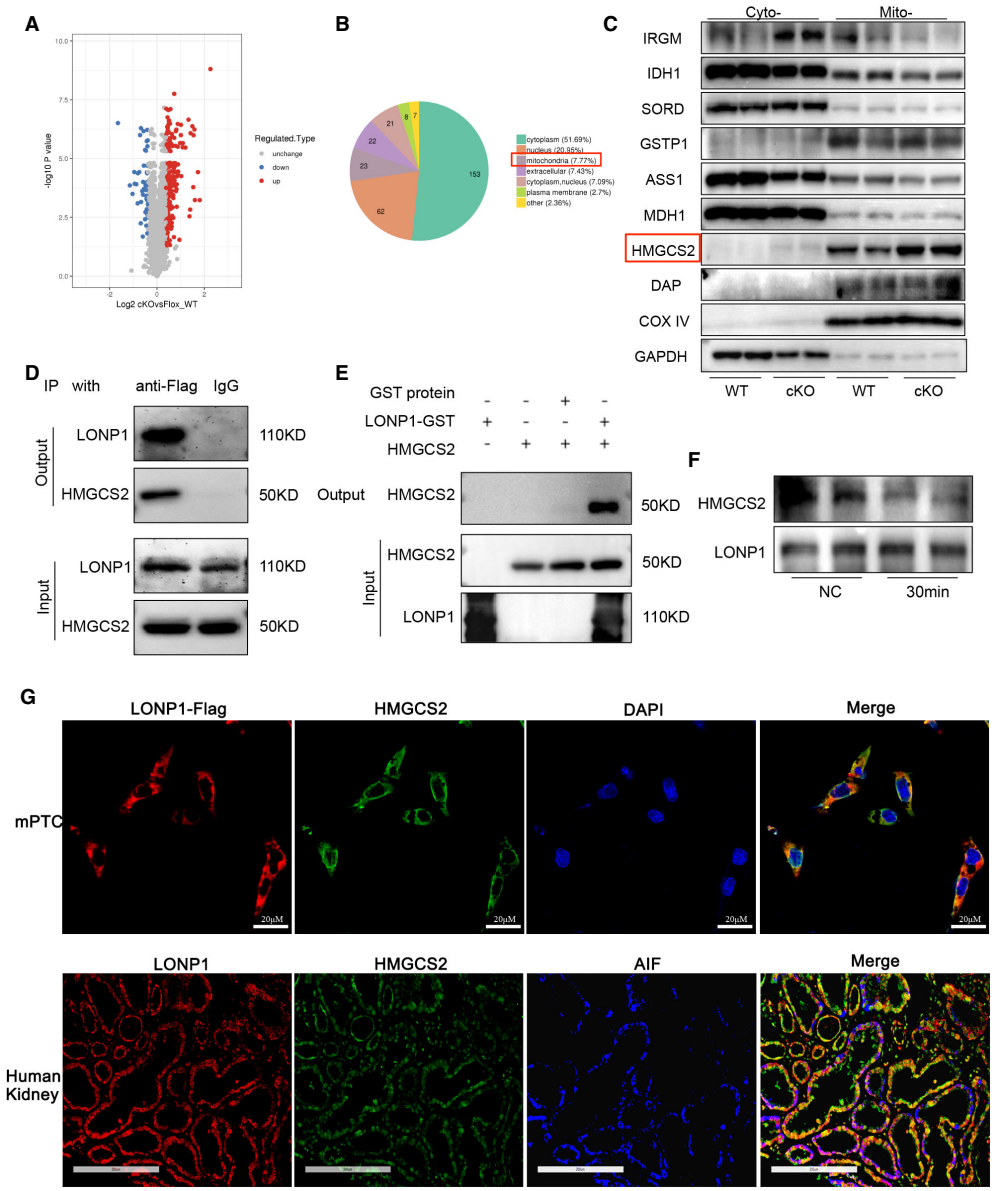
HMGCS2 may be the degradation substrate of LONP1 The proximal renal tubules of *Lonp1* cKO mice were extracted and detected by mass spectrometry (*n* = 3, biological replicates).Subcellular localization of up‐regulated proteins.Western blot validation of the up‐regulated proteins in mitochondria (*n* = 2, biological replicates).CoIP analysis of interaction between LONP1 and HMGCS2.GST pull down analysis of interaction between LONP1 and HMGCS2.Enzymatic hydrolysis of LONP1 to HMGCS2.Immunofluorescence co‐localization analysis of LONP1, HMGCS2 and mitochondria in mPTC (up, scale bar: 20 μm) and human kidney tissue (down, scale bar: 200 μm). Source data are available online for this figure.

To further explore the mechanism of the renal‐protective effect of LONP1, we performed isobaric tags for relative and absolute quantification (iTRAQ)‐based quantitative proteomic analysis to assess differential protein‐expression levels in the extracted proximal renal tubules of WT and *Lonp1*‐cKO mice (Fig 5A). Considering that knocking out *Lonp1* resulted in the accumulation of downstream protein substrates, we further analyzed the localization of upregulated mitochondrial proteins (Fig 5B). Western blot analysis showed that HMGCS2 expression increased significantly in extracted renal tubule mitochondria from *Lonp1*‐cKO mice (Fig 5C). LONP1 directly associated with HMGCS2, as determined by co‐immunoprecipitation (Fig 5D). Pull‐down analysis based on glutathione S‐transferase (GST) showed that the LONP1‐GST fusion protein could capture HMGCS2 (Fig 5E). In cell‐free experiments, we also found that LONP1 could catalyze enzymolysis of the HMGCS2 protein (Fig 5F). We then performed a fluorescence co‐localization assay in mPTC and human kidney sections. We found that LONP1 and HMGCS2 were coexpressed in renal tubular cells and that they colocalized with apoptosis‐inducing factor mitochondria‐associated 1 (AIF), a mitochondrial marker, in human kidney tissues (Fig 5G).

**Figure 6:**
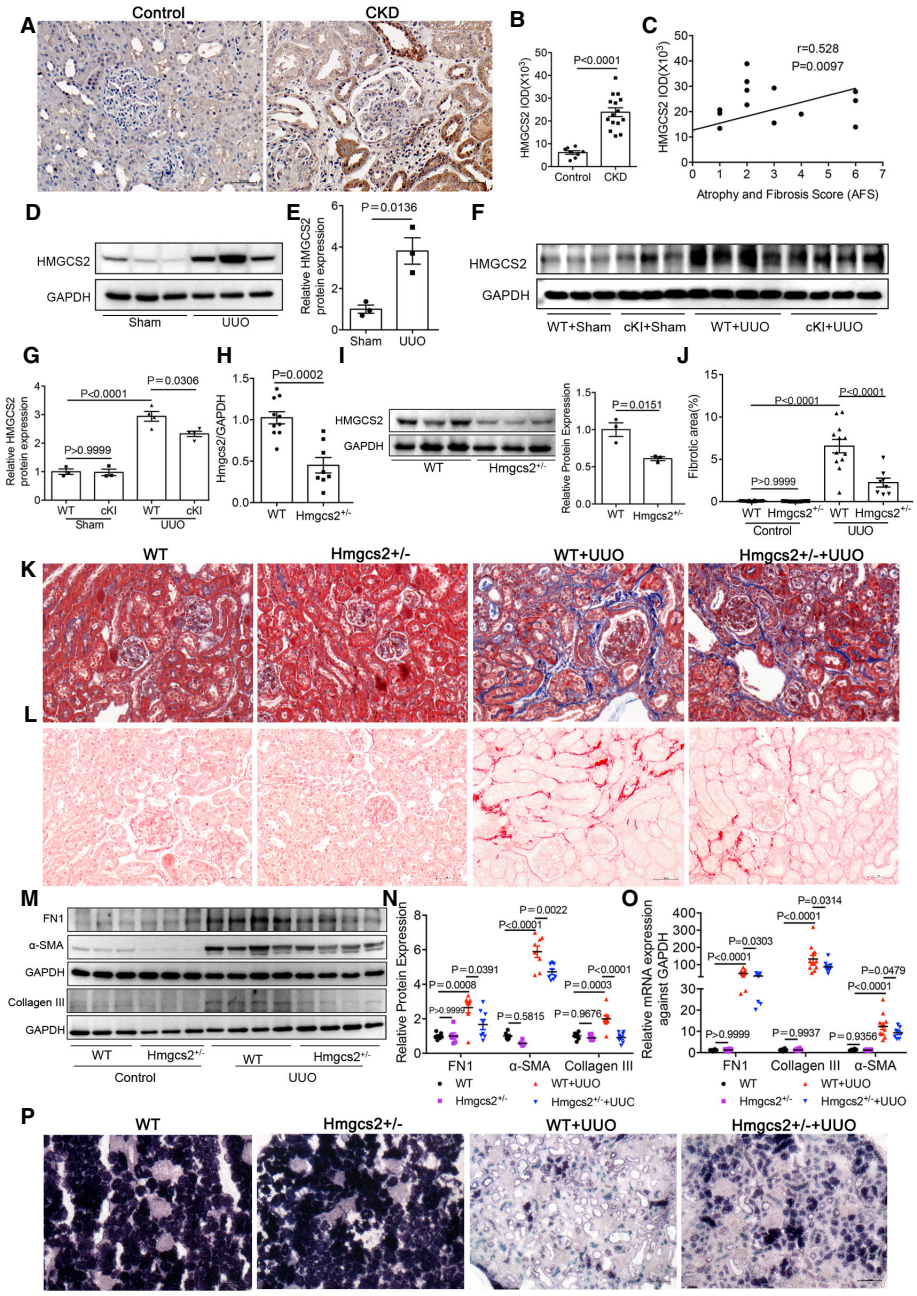
Effect of HMGCS2 on mitochondrial function and renal fibrosis AImmunohistochemical analysis of HMGCS2 expression in CKD children. Scale bar: 50 μm.BImmunohistochemical semi‐quantitative IOD analysis of HMGCS2 (*n* = 8 in Control group, *n* = 15 in CKD group).CPearson correlation analysis of HMGCS2 and atrophy and fibrosis score (AFS) in renal biopsy specimens.DWestern blot analysis for the expression of HMGCS2 in UUO model.EDensitometric analysis for the expression of HMGCS2 in UUO model (*n* = 3, biological replicates).F, GWestern blot and densitometric analysis of HMGCS2 in WT and cKI mice of UUO model (*n* = 3 or 4, biological replicates).H, IqRT‐PCR and western blotting analysis of Hmgcs2 expression in WT and Hmgcs2^+/−^ mice (*n* = 3, biological replicates).JFibrotic area statistics of Sirius red staining in WT and Hmgcs2^+/−^ mice of UUO model (*n* = 12 in WT groups, *n* = 8 in Hmgcs2^+/−^ groups, biological replicates).KDeposition of total fibrosis in kidney tissues in WT and Hmgcs2^+/−^ mice after UUO was determined by Masson's trichrome staining. Scale bar: 50 μm.LSirius red staining of WT and Hmgcs2^+/−^ mice of UUO model. Scale bar: 50 μm.M, NWestern blot and densitometric analysis for the expression of FN1, α‐SMA and Collagen III in WT and Hmgcs2^+/−^ mice after UUO (*n* = 6 in control groups, *n* = 8 in UUO groups, biological replicates).OqRT‐PCR analysis of FN1, α‐SMA and Collagen III in WT and Hmgcs2^+/−^ mice after UUO (*n* = 10 or 12 in WT groups, *n* = 8 in Hmgcs2^+/−^ groups, biological replicates).PSuccinate dehydrogenase (SDH) staining in WT and Hmgcs2^+/−^ mice after UUO. Scale bar: 100 μm. Data information: In (B, E, H, I), data are presented as mean ± SEM. Student's *t*‐test. In (G, J, N, O), data are presented as mean ± SEM. One‐way ANOVA. Source data are available online for this figure.

Next, to investigate the role of HMGCS2 in terms of mitochondrial function and renal fibrosis, we firstly performed IHC staining with biopsied kidney tissues from CKD patients. Compared with the control human kidneys, significant upregulation of HMGCS2 was observed in patient kidneys, primarily in the renal tubular cells (Fig 6A and B). More importantly, the level of HMGCS2 expression positively correlated with the degree of kidney fibrosis, as assessed by the atrophy and fibrosis score (AFS; *r* = 0.528, *P* = 0.010; Fig 6C). We also found the HMGCS2 expression was significantly increased in UUO model (Fig 6D and E), which was downregulated after *Lonp1* overexpression (Fig 6F and G).

*In vivo*, we generated *Hmgcs2* ^+/−^ mice (Fig 6H and I) and established a UUO model. Masson's trichrome (Fig 6K) and Sirius red staining (Fig 6L and J) revealed that kidney fibrosis induced by UUO was repressed in *Hmgcs2* ^+/−^ mice, compared with that in the WT group. Western blot and qRT‐PCR analysis also showed *Hmgcs2* knockdown alleviated the deposition of extracellular matrix components (FN1 and Collagen III) and α‐SMA expression (Fig 6M–O). The SDH activity also attenuated in *Hmgcs2* ^+/−^ mice after UUO (Fig 6P).

**Figure EV4:**
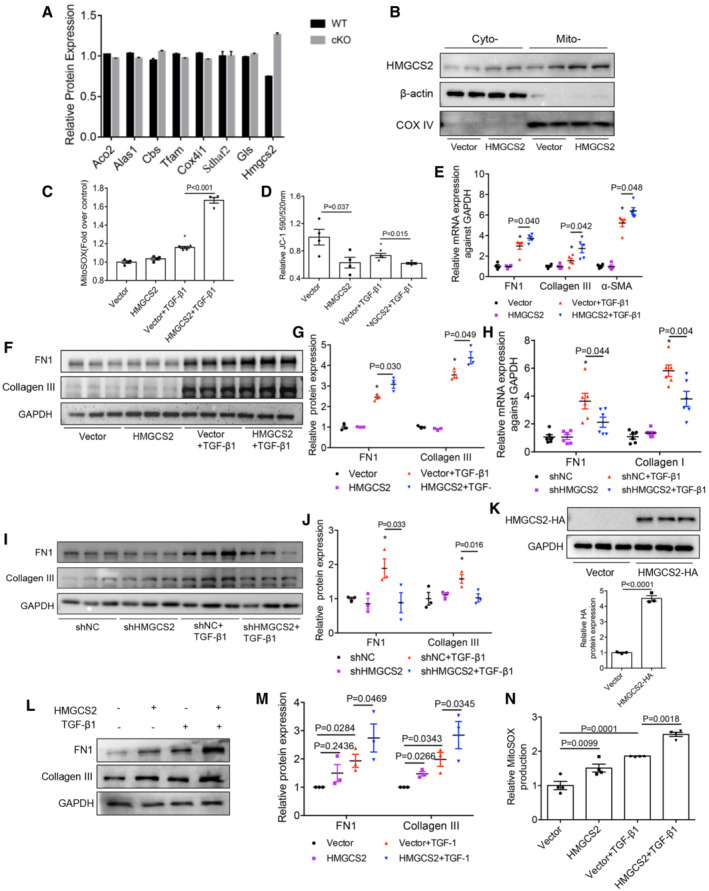
HMGCS2 aggravated TGF‐β1‐induced mitochondrial dysfunction and fibrotic response in mPTCs and primary proximal tubular cells AThe expression of known substrates of LONP1 and HMGCS2 in our proteomics (*n* = 3, biological replicates). Compared with Hmgcs2, there was little difference in their expression in WT and Lonp1 cKO groups.BWestern blot showing the intramitochondrial localization of HMGCS2 in mPTC cells after transfected with Vector or *Hmgcs2* plasmids (*n* = 2, biological replicates).CQuantification of mitochondrial ROS production in mPTC cells after transfected with Vector or *Hmgcs2* plasmids following TGF‐β1 treatment (*n* = 4 in each group, biological replicates).DQuantitation of mitochondrial membrane potential (ΔΨm) by JC‐1 staining in mPTC cells after transfected with Vector or *Hmgcs2* plasmids following TGF‐β1 treatment (*n* = 4 in each group, biological replicates).EqRT‐PCR analysis of FN1, Collagen III and α‐SMA in mPTC cells after transfected with Vector or *Hmgcs2* plasmids following TGF‐β1 treatment (*n* = 4 or 5, biological replicates).F, GWestern blot and densitometric analysis for the expression of FN1 and Collagen III in mPTC cells after transfected with Vector or *Hmgcs2* plasmids following TGF‐β1 treatment (*n* = 3 in each group, biological replicates).HqRT‐PCR analysis of FN1 and Collagen I in mPTC cells after transfected with shNC or sh*Hmgcs2* plasmids following TGF‐β1 treatment (*n* = 6 in each group, biological replicates).I, JWestern blot and densitometric analysis for the expression of FN1 and Collagen III in mPTC cells after transfected with shNC or sh*Hmgcs2* plasmids following TGF‐β1 treatment (*n* = 3 in each group, biological replicates).KThe proximal tubular cells were isolated from C57BL/6J mice and infected with HMGCS2‐lentivirus (pLVX‐Puro‐mHmgcs2‐HA) or control lentivirus (vector). Western blot and densitometric analysis for the expression of HA (*n* = 3 in each group, biological replicates).L, MThe infected primary cells were treated with TGF‐β1 for 24 h after infected with HMGCS2‐lentivirus or control lentivirus. Western blot and densitometric analysis for the expression of FN1 and Collagen III. Three independent experiments were carried out and quantification of the abundance of these proteins is shown in panel (*n* = 3 in each group).NThe infected primary cells were treated with TGF‐β1 for 4 h. Quantification of mitochondrial ROS production (*n* = 4 in each group, biological replicates). Data information: In (K), data are presented as mean ± SEM. Student's *t*‐test. In (C–E, G, H, J, M, N), data are presented as mean ± SEM. One‐way ANOVA. Source data are available online for this figure.

*In vitro*, we transfected mPTCs with an Hmgcs2‐overexpression plasmid or the control vector, and then treated the cells with TGF‐β1 *in vitro*. Compared with the control mPTC transfectants, the overexpressed HMGCS2 was mainly increased in the mitochondria (Fig EV4B). Consistent with our *in vivo* results, HMGCS2 overexpression further aggravated the production of TGF‐β1‐induced mitochondrial ROS; the decreased of mitochondrial membrane potential; and increased the synthesis of FN, Collagen III, and α‐SMA (Fig EV4C–G). These results were validated in primary proximal tubular cells isolated from C57BL/6J mice (Fig EV4K–N). Conversely, inhibiting HMGCS2 expression significantly inhibited the synthesis of Collagen I, Collagen III and FN1, induced by TGF‐β1 (Fig EV4H–J).

Collectively, these results demonstrate that LONP1 attenuated mitochondrial dysfunction and renal fibrosis, possibly by degrading HMGCS2 to prevent the accumulation of HMGCS2 under CKD conditions.

### An LONP1 activator effectively suppressed TGF‐β1‐induced fibrotic responses in mPTCs and attenuated UUO and UIRI‐induced renal fibrosis and mitochondrial dysfunction

**Figure 7:**
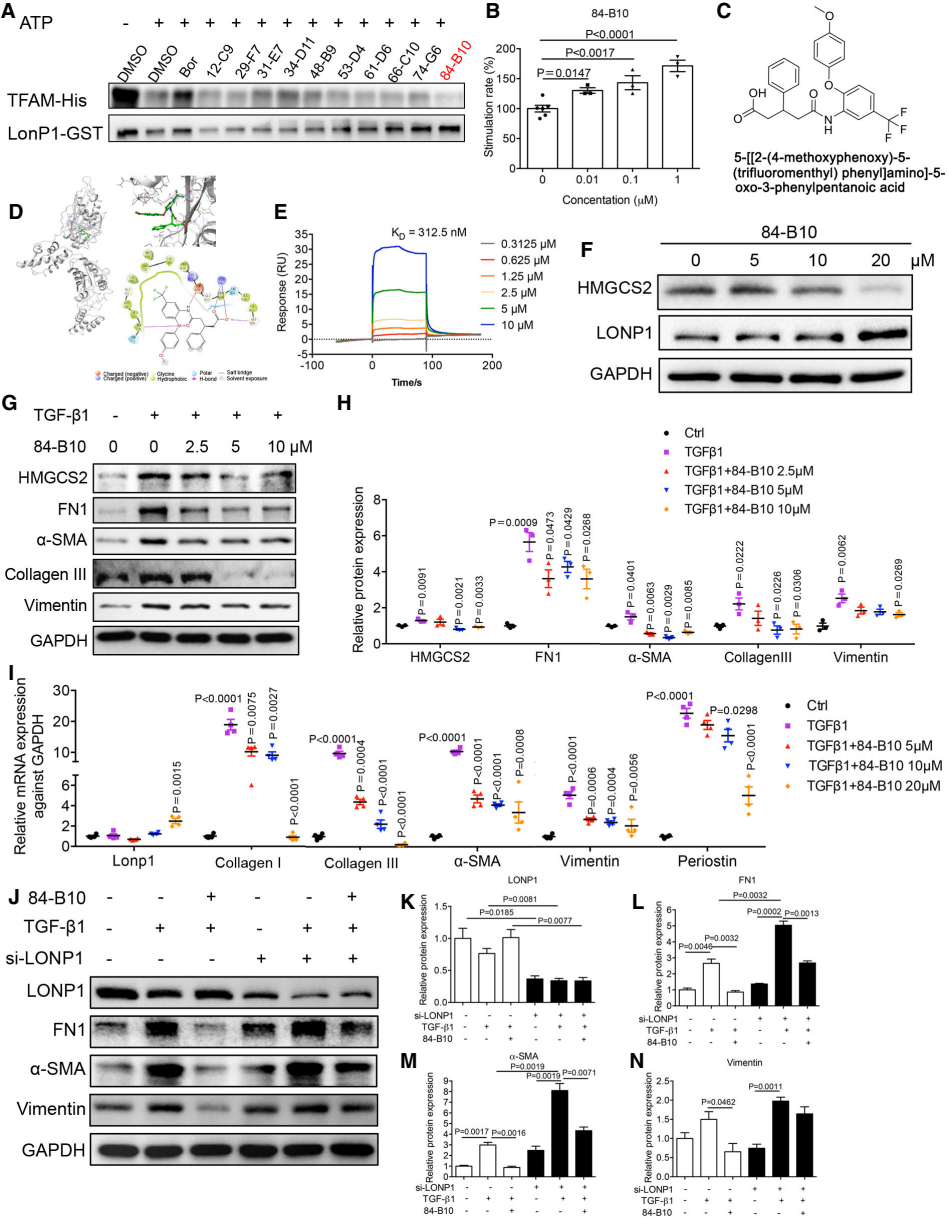
The anti‐fibrotic response effect of a novel LONP1 activator in mPTC cells A10 high docking score molecules showed LONP1 activating effect in cell free LONP1 protease activity assay system, of which 84‐B10 showed stronger activity. Bortezomib (Bor), a reported LONP1 inhibitor, is involved as a contrast.B84‐B10 dose dependently induced the peptidase activity of LONP1 as estimated by detecting the fluorogenic di‐peptide substrate (Z‐AA)_2_‐Rh110 (*n* = 6 or 3, biological replicates).CChemical structure of 84‐B10.DProposed binding model of 84‐B10 binding to the human LONP1 catalytic domain. Three and two‐dimensional illustrations of the interaction between 84‐B10 and human LONP1 catalytic domain.EPhysical interaction between 84‐B10 and LONP1 performed by Surface Plasmon Resonance (SPR).FProtein levels of HMGCS2 and LONP1 were estimated in mPTCs treated with different concentration of 84‐B10 or vehicle (0.1% DMSO) for 24 h.G, HRepresentative immunoblot and densitometric analysis of HMGCS2, FN1, Collagen III, α‐SMA and Vimentin in mPTCs treated as indicated. Three independent experiments were carried out and quantification of the abundance of these proteins is shown in panel (*n* = 3 in each group).IqRT‐PCR analysis of *Lonp1*, Collagen I, Collagen III, α‐SMA, Vimentin and Periostin transcript levels in mPTCs treated as indicated (*n* = 4 in each group, biological replicates).J–NRepresentative Western blot and densitometric analysis of LONP1, FN1, α‐SMA and Vimentin of each group was shown. Three independent experiments were carried out and quantification of the abundance of these proteins is shown in panel (*n* = 3 in each group). mPTCs were transfected with siNC or siLONP1, and then pre‐treated with 84‐B10 (10 μM) 2 h before TGF‐β1 (10 ng/ml) treatment for another 24 h. Data information: Data are presented as mean ± SEM. Student's *t*‐test. Source data are available online for this figure.

The data described above led us to investigate the therapeutic potential of targeting of LONP1. We thus screened for effective LONP1 activators. Molecular docking was adopted for screening purposes, and molecules with high docking scores were further tested by performing cell‐free LONP1 protease‐activity assays. Among the 19 molecules showing an LONP1‐activating effect, molecule 84‐B10 (chemical name: 5‐[[2‐(4‐methoxyphenoxy)‐5‐(trifluoromethyl) phenyl] amino]‐5‐oxo‐3‐phenylpentanoic acid) exhibited the most significant effect, as revealed by the highest degradation level of the known substrate transcription factor A, mitochondrial (TFAM) (Fig 7A). Protease activity assay based on the fluorogenic dipeptide substrate demonstrated that 84‐B10 dose‐dependently stimulated the enzymatic activity of LONP1 (Fig 7B). As shown by the binding models displayed as 2D and 3D diagrams, 84‐B10 can form four conventional H‐bonds with TRP770, ASP852, LYS898, and GLY893, as well as one salt bridge with LYS898 (Fig 7C and D). Further, the binding affinities of 84‐B10 with recombinant LONP1 protein was quantified with a Surface Plasmon Resonance (SPR) facility. Result revealed reliable binding activity between the molecules, with the binding affinities (KD value) calculated as 312.5 nM (Fig 7E).

The effect of the molecular 84‐B10 on LONP1 was further verified in mPTCs. Notably, 84‐B10 significant reduced HMGCS2 protein expression in mPTCs; meanwhile, LONP1 expression remained invariant at low 84‐B10 concentrations, but dramatically increased at 84‐B10 concentrations above 10 μM (Fig 7F). Subsequently, the cell protective effect of 84‐B10 was evaluated. mPTCs were pre‐treated with 5, 10, or 20 μM 84‐B10 for 2 h and then stimulated with TGF‐β1. We tested the protein levels of HMGCS2 after 84‐B10 and TGF‐β1 treatment. The result showed that HMGCS2 expression increased after TGF‐β1 treatment and was downregulated by LONP1 activation (Fig 7G and H). Along with the decrease in HMGCS2 expression, the protein levels of the fibrosis markers FN1, α‐SMA, Collagen III, and Vimentin were markedly repressed (Fig 7G and H). The mRNA‐expression level of *Lonp1* increased at an 84‐B10 concentration of 20 μM (Fig 7I). Based on these results and those presented in Fig 7F, we inferred that 84‐B10 could activate the enzyme activity of LONP1 at a low concentration and stimulate LONP1 transcription and translation at a high concentration. Along with the stimulation on LONP1, TGF‐β1‐induced partial epithelial‐mesenchymal transition and matrix generation was dose‐dependently alleviated by 84‐B10, as demonstrated by the transcription levels of Collagen I, Collagen III, α‐SMA, Vimentin, and Periostin (Fig 7I). Notably, the alleviating effect of 84‐B10 on fibrosis markers is weakened if LONP1 is knockdown by siRNA (Fig 7J–N), demonstrating that the tubular epithelial cell protection effect of 84‐B10 is dependent on LONP1. It is known that injured renal tubules can secrete various factors to act on fibroblasts and promote their activation. Therefore, we silenced LONP1 with shRNA in renal tubular cells for 24 h and then collected the supernatant to stimulate NRK‐49F cells and found that it could promote the activation of fibroblasts (evidenced by increasing expression of FN1 and Collagen III) (Appendix Fig S1A–D). We also evaluated the direct influence of 84‐B10 on rat kidney fibroblasts NRK‐49F. As is shown in Appendix Fig S1E–G, TGF‐β1 stimulated the activation of NRK‐49F cells (evidenced by increasing expression of FN1 and α‐SMA), which was dose dependently inhibited by 84‐B10.

**Figure EV5:**
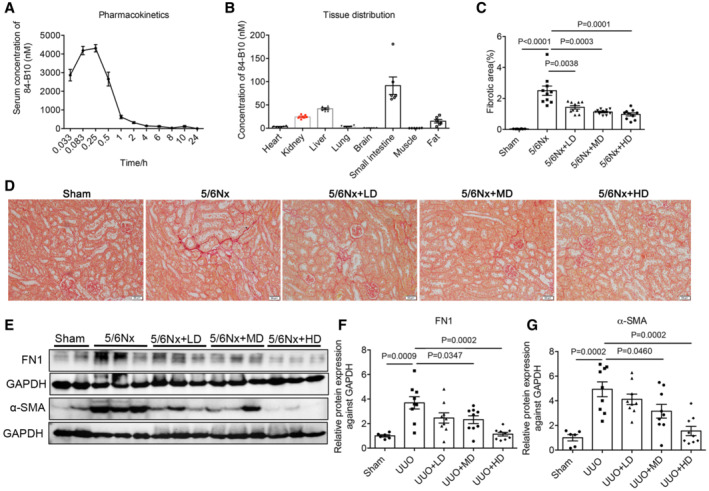
Pharmacokinetics characteristic of 84‐B10 and its anti‐fibrosis effect in 5/6Nx mice APlasma‐concentration versus time profiles of 84‐B10 in mice (*n* = 6, biological replicates).BTissue distribution of 84‐B10 in mice 30 min after injection (*n* = 6, biological replicates).C, DSirius red staining and fibrotic area statistics of 5/6Nx mice treated with LONP1 activator 84‐B10. Scale bar: 50 μm (*n* = 8 in WT group, *n* = 10 in other three groups, biological replicates).E–GWestern blot and densitometric analysis for the expression of FN1 and α‐SMA in 5/6Nx mice treated with 84‐B10 (*n* = 6 in WT group, *n* = 9 in other three groups, biological replicates). Data information: Data are presented as mean ± SEM. Student's *t*‐test. Source data are available online for this figure.

The plasma pharmacokinetics (PK), tissue distribution and kidney protective efficacy of 84‐B10 was further verified *in vivo*. After a single intraperitoneal injected of 84‐B10 (5 mg/kg), the plasma‐concentration versus time profiles of 84‐B10 were detected using LC–MS/MS (Fig EV5A), and the calculated PK parameters were: T_1/2_, 2.16 h; T_max_, 0.25 h; C_max_, 4,523.0168 nM; AUC_0–inf_ 4,653.7194 (h × nmol/l). Tissue concentration of 84‐B10 was estimated 30 min after 84‐B10 injection (intraperitoneal, 5 mg/kg). As shown in Fig EV5B, 84‐B10 was mainly distributed in small intestine (91.34 ± 18.68 nM/g tissue weight), liver (41.68 ± 2.09 nM/g tissue weight) and kidney (24.32 ± 1.72 nM/g tissue weight). Based on these data, the efficacy of 84‐B10 was further verified using UUO, 5/6Nx and unilateral ischemia reperfusion injury (UIRI) induced renal fibrosis murine models.

**Figure 8:**
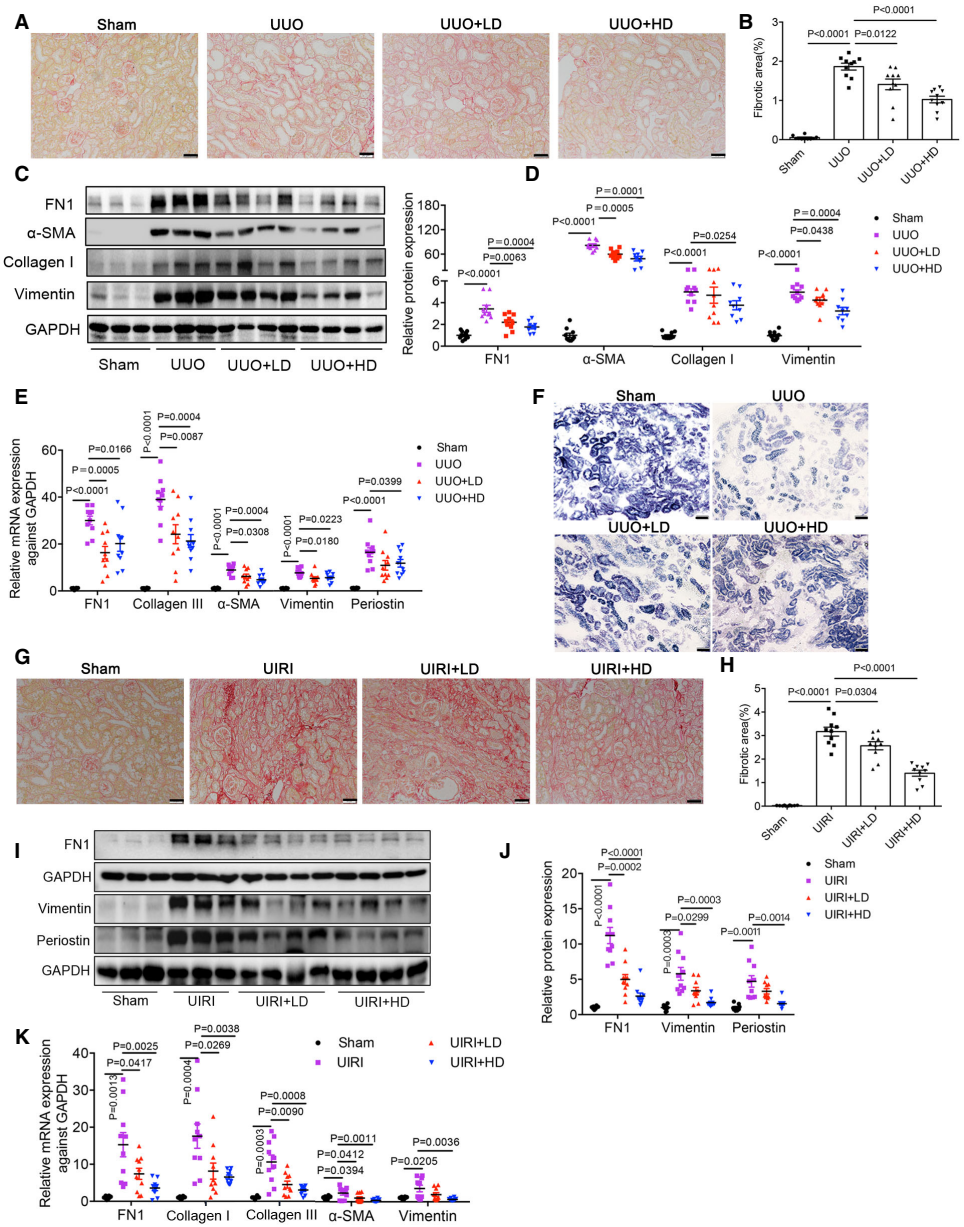
Anti‐renal fibrosis and maintenance of mitochondrial function of LONP1 activator in UUO and UIRI models AFibrosis deposition of kidney tissues was determined by Sirius red staining in UUO model. Scale bar: 50 μm.BQuantification of fibrotic area in UUO model (*n* = 10 in each group, biological replicates).C, DRepresentative Western blot images and densitometric analysis of FN1, Collagen I, α‐SMA and Vimentin protein levels in kidney tissues of UUO model (*n* = 9–10 in each group, biological replicates).EqRT‐PCR analysis of FN1, Collagen III, α‐SMA, Vimentin and Periostin mRNA levels in kidney tissues of UUO different groups (*n* = 10 in each group, biological replicates).FRepresentative images of succinate dehydrogenase (SDH) staining in UUO model. Scale bar: 50 μm.G, HFibrosis deposition of kidney tissues was determined by Sirius red staining and quantified in UIRI model (*n* = 10 in each group, biological replicates). Scale bar: 50 μm.I, JWestern blot and densitometric analysis of FN1, Vimentin and Periostin protein levels in kidney tissues of UIRI model (*n* = 8–10 in each group, biological replicates).KqRT‐PCR analysis of FN1, Collagen I, Collagen III, α‐SMA and Vimentin mRNA levels in kidney tissues of UIRI different groups (*n* = 8–10 in each group, biological replicates). Data information: Data are presented as mean ± SEM. Student's *t*‐test. Source data are available online for this figure.

In treating the UUO model, mice were pre‐treated with a low dose (0.5 mg/kg) or a high dose (7.5 mg/kg) of 84‐B10 before UUO surgery and were then treated daily for 7 consecutive days and sacrificed. Measuring the serum concentrations of BUN, Scr, aspartate aminotransferase (AST), alanine aminotransferase (ALT), lactate dehydrogenase (LDH), and creatine kinase‐MB (CK‐MB) showed that therapeutic doses of 84‐B10 elicited no obvious renal, hepatic, heart, and systemic toxicity (Appendix Fig S2A–F). Sirius red staining of kidneys from 84‐B10‐treated mice revealed a recovered morphology with a low degree of fibrotic deposition on day 7 after UUO (Fig 8A and B). Consistently, the protein (FN1, Collagen I, α‐SMA, and Vimentin) (Fig 8C and D) and mRNA (FN1, Collagen III, α‐SMA, Vimentin, and Periostin) (Fig 8E) expression levels of fibrotic factors were also significantly lower in 84‐B10‐treated mice. Furthermore, both low‐dose and high‐dose 84‐B10 treatment restored the mitochondrial functions of UUO kidneys, as demonstrated by the improved SDH activity (Fig 8F). In 5/6Nx mice, 84‐B10 treatment also dose‐dependently attenuated the kidney fibrosis and reduced the expression of fibrotic factors (Fig EV5C–G). Further, we extended our studies to ischemic injury induced renal fibrosis. Mice were subjected to 45 min of unilateral ischemia reperfusion injury (UIRI) and received 84‐B10 treatment 72 h after reperfusion. After 19 consecutive days of low dose (2.5 mg/kg) and high dose (5 mg/kg) 84‐B10 treatment, the extracellular matrix accumulation estimated by Sirius red staining (Fig 8G and H), as well as protein (Fig 8I and J) and mRNA (Fig 8K) expression levels of fibrotic factors was dose dependently ameliorated. In addition, the low expression of key fatty acid oxidation enzymes (ACOX1 and ACADM) in UIRI mice was also rescued by 84‐B10 treatment, indicating an improved mitochondrial function (Appendix Fig S3). The therapeutic doses of 84‐B10 also showed no obvious renal, hepatic, heart, and systemic toxicity (Appendix Fig S2G–L).

## Discussion

Renal tubular injury and tubulointerstitial fibrosis represent the final common pathway to ESRD. Among residential renal cells, tubular epithelial cells act as the principal effectors in initiating and mediating renal fibrosis. The apoptosis and inflammation of tubular epithelial cells are directly related to the severity of renal fibrosis and the damage to renal function. Therefore, protecting proximal tubular cells against injury is essential for preventing the development and progression of CKD. Here, we found *Lonp1* deletion impaired mitochondrial homeostasis and aggravated renal fibrosis through HMGCS2 accumulation in CKD mice or cellular models, whereas *Lonp1* overexpression or treatment with an LONP1 activator alleviated these injuries.

Ensuring the normal quantity and function of mitochondrial proteins is very important for maintaining the steady state of mitochondria (Palikaras & Tavernarakis, 2014). Dysregulated mitochondrial protein transcription and translation, as well as the loss of mitochondrial protein activity, can lead to imbalanced mitochondrial homeostasis, which results in an abnormal increase of ROS or abnormal mitochondrial synthesis, fusion, and division (Han *et al*, 2014; Bueno *et al*, 2015). However, mitochondria are the main site of ROS production and mitochondrial proteins are prone to oxidative modification, resulting in protein misfolding and assembly. The accumulation of these abnormal proteins can directly lead to imbalanced mitochondrial homeostasis and cellular dysfunctions, which are involved in tumorigenesis, aging, chronic degenerative disease, and kidney disease (Hamon *et al*, 2015; Jadiya & Tomar, 2020; Song *et al*, 2021). It is very important to remove these abnormal mitochondrial proteins and control the quality of mitochondrial proteins in order to maintain mitochondrial homeostasis and cellular functions.

Lon protease is the most abundant and important protease in mitochondria, and is involved in degrading abnormal mitochondrial proteins, especially those with oxidative damage, or misfolded and improperly assembled proteins (Matsushima *et al*, 2010). Previously, it was found that knocking out Lon protease directly led to the death of mouse embryos (Quiros *et al*, 2014). Decreasing the level or activity of Lon protease led to the abnormal accumulation of mitochondrial proteins, which is associated with abnormal development of the central nervous system (Wang *et al*, 2019a), liver cell dysfunction (Tian *et al*, 2011), cardiac stress (Smyrnias *et al*, 2019), insulin resistance (Lee *et al*, 2011), and tumor development (Gibellini *et al*, 2018). Our previous findings revealed that reduced LONP1 expression in podocytes contributed to the pathogenesis of podocytopathy (Gong *et al*, 2021). In this study, we observed a prominent decrease in LONP1 expression (mainly renal tubular LONP1 expression) in kidney biopsy tissues from patients with CKD, compared with the expression levels in control kidney tissues, with varying degrees of renal fibrosis. Moreover, the LONP1‐expression level correlated negatively with the degree of kidney fibrosis, suggesting a key role for LONP1 in renal fibrosis. Based on these findings, we hypothesized the decreased LONP1 expression in renal tubules might mediate renal fibrosis in CKD. By using renal tubular *Lonp1*‐cKO or *Lonp1*‐cKI mice and proximal tubular epithelial cells, we demonstrated for the first time that LONP1 prevents the progression of renal fibrosis in two CKD models (UUO and 5/6Nx) by maintaining mitochondrial homeostasis.

To clarify the mechanism whereby LONP1 is involved in maintaining mitochondrial function in CKD, we performed iTRAQ analysis to assess differential protein‐expression levels in tubules extracted from WT and *Lonp1*‐cKO mice. Among the identified differentially expressed proteins, HMGCS2 was markedly upregulated in the mitochondria of tubules extracted from *Lonp1*‐cKO mice, when compared with that in WT controls. By performing co‐immunoprecipitation, GST pull‐down, and enzymolysis assay, we confirmed that HMGCS2 might be a substrate for LONP1. HMGCS2 is a mitochondrial enzyme that catalyzes the first reaction of ketogenesis and related research has mainly focused on its roles in diabetes, tumor, Alzheimer's disease, and intestinal cell differentiation (Shi *et al*, 2017; Wang *et al*, 2017, 2019b, 2020b; Wan *et al*, 2018; Kim *et al*, 2019; Tomita *et al*, 2020). Increased HMGCS2 expression in diabetes enhanced the production of ketone bodies, thus leading to diabetic cardiomyopathy (Wang *et al*, 2020a), whereas the function of HMGCS2 in diabetic nephropathy remains controversial. Tomita *et al* (2020) reported that SGLT2 inhibitors promoted ketone body rise through HMGCS2, thereby inhibiting mTORC1 in proximal renal tubules, explaining the protective effect of SGLT2 inhibitors on kidney. Knockout of HMGCS2 resulted in the loss of renal protective effect of SGLT2 inhibitors in diabetic kidney disease (DKD) and non‐DKD kidneys (Tomita *et al*, 2020). Zhang *et al* (2011) found that diabetic kidneys exhibit excess ketogenic activity resulting from increased HMGCS2 expression, thus leading to tubular injury. A recent study found that interference with *Hmgcs2* in podocytes or glomerulus‐specific knockout of *Hmgcs2* could effectively prevent high‐fructose induced mitochondrial dysfunction and reduce podocyte damage (Fang *et al*, 2021). However, no direct evidence has indicated that HMGCS2 is involved in renal fibrosis. In this study, knockdown of *Hmgcs2* attenuated renal fibrosis and mitochondrial dysfunction induced by UUO model, which was consistent with the findings that overexpressing HMGCS2 in proximal tubular epithelial cells impaired mitochondrial function and aggravated production of the extracellular matrix. It has been reported that the inhibition of HMGCS2 in the liver will cause the impairment of hepatic ketogenesis, which can promote liver fibrosis through the various factors derived from the injured hepatocytes and activation of macrophages (Puchalska *et al*, 2019; Mooli & Ramakrishnan, 2022). This is not consistent with our experimental results. The possible reason is that although fibrosis is a general reparative or reactive reaction in organs, the mechanisms involved in fibrosis in different organs are not the same. HMGCS2 plays a non‐ketogenic role in extrahepatic mitochondria contrasting to its ketogenic action in liver mitochondria (Puchalska & Crawford, 2017). Venable *et al* (2022) found that the expression of HMGCS2 in the kidney increased under fasting conditions but did not affect the production of circulating ketone bodies, suggesting that HMGCS2 in kidney may have different functions. In our study, HMGCS2 promoted renal fibrosis by damaging mitochondrial function.

Considering the tubular‐protective effect of LONP1 and that no specific LONP1 activator has been previously reported, molecular docking was performed to screen for potential LONP1 activators. The LONP1‐activating effect of candidate molecules with high scores was further tested by substrate protein‐based protease activity assay, fluorogenic dipeptide substrate‐based protease activity and SPR assay in a cell‐free system, as well as in cultured mPTCs. Among the candidate molecules identified, the novel molecule 84‐B10 showed the most promising LONP1‐activating effect and a clear interaction with LONP1. Excitingly, 84‐B10 showed remarkable antifibrotic effect on both renal tubular epithelial cells and fibroblasts *in vitro*. *In vivo*, 84‐B10 protected mitochondria and antagonized renal fibrosis in UUO, 5/6Nx and UIRI mice. No organ and systemic toxicity were observed with 84‐B10 treatment under the therapeutic dosages ranging from 0.5 to 10 mg/kg/day for 8–42 days, indicating a wide therapeutic window of 84‐B10 and thus ensure its safety for potential clinical usage. However, the mechanism whereby 84‐B10 activates LONP1 remains elusive. Based on the result that the mRNA and protein level of LONP1 remain unchanged at the concentration below 10 μM while significantly increased at the concentration of 20 μM, 84‐B10 may function to activate the enzyme activity of LONP1 at low concentration and stimulate the transcription and expression of LONP1 at high concentration. In addition, since LONP1 is reported to be assembled as six protomers and couples the ATPase cycle into motion to drive substrate translocation and degradation by binding to three ADPs (Lin *et al*, 2016), 84‐B10 may also help promote the formation of LONP1 hexamers. Further mechanistic study and clinical testing of the safety and efficiency of 84‐B10 will be carried out in the future.

The limitation of our study is that, to date, some specific mitochondrial substrate proteins for LONP1 have been reported, including oxidized mitochondrial aconitase (ACO2) (Bota & Davies, 2002), cystathionine β‐synthase (CBS) (Teng *et al*, 2013), 5‐aminolevulinic acid synthase (ALAS1) (Tian *et al*, 2011), and TFAM (Matsushima *et al*, 2010; Lu *et al*, 2013), among others. These reported substrates were not differentially expressed based on our proteomics data (Fig EV4A), but this does not rule out whether they play roles in LONP1 dysfunction in renal fibrosis, which requires further investigation. Another limitation of the present study was the absence of experiments designed to investigate the molecular pathways leading to reduced LONP1 expression in CKD. Previous reports have demonstrated some factors can regulate the expression or activity of LONP1, such as SIRT1 (Kalvala *et al*, 2020), Akt (Ghosh *et al*, 2019), and eIF2α (Onat *et al*, 2019). Theoretically, some of these factors may also participate in the renal fibrosis of CKD by regulating LONP1.

Overall, our results suggest that LONP1, to the best of our knowledge, acts as an important factor in the renal fibrosis of CKD by degrading HMGCS2 and thereby regulating mitochondrial homeostasis. Agonizing the LONP1‐signaling pathway may provide a new approach for maintaining renal mitochondrial function and impeding the progression of clinical CKD.

## Materials and Methods

### Study approval

All experimental procedures regarding human tissue were approved by the Research Ethics Board of Children's Hospital of Nanjing Medical University (201703048). Proper informed consent was obtained from all human subjects and that the experiments conformed to the principles set out in the WMA Declaration of Helsinki and the Department of Health and Human Services Belmont Report. All animal experiments were performed in accordance with the National Institutes of Health guidelines for the care and use of animals in research and approved by the Institutional Animal Care and Use Committee at Nanjing Medical University (2007001‐7, 2102005‐1). For treatment experiments, control or experiment groups were assigned randomly; where possible researchers were blinded to the treatment group, but blinding was not possible for mouse groups due to institutional requirements for labeling Lonp1 cKO or cKI or Hmgcs2^+/−^ animals.

### Reagents and antibodies

Dulbecco's modified Eagle's medium (DMEM)‐F12 (C11330500CP), fetal bovine serum (FBS, 10091148) and 0.25% trypsin‐0.02% EDTA (25200‐056) were purchased from GIBCO (Waltham, MA, USA). Antibodies of anti‐LONP1 (HPA002192), anti‐flag (F1804) were purchased from Sigma‐Aldrich (St. Louis, MO, USA), anti‐TFAM (22586‐1‐AP), anti‐ACOX1 (10957‐1‐AP) and anti‐ACADM (55210‐1‐AP) were obtained from Proteintech Group (Rosemont, IL, USA), anti‐HMGCS2 (20940s, for IP) and anti‐COX IV (4844s) were obtained from Cell Signaling Technology (Beverly, MA, USA) and normal mouse IgG was from santacruze (sc‐2025). Antibodies of anti‐α‐SMA (ab7817), anti‐FN1 (ab2413), anti‐Collagen IV (ab6586), anti‐Vimentin (ab92547) and anti‐AIF (ab32516) were from Abcam (Cambridge, MA, USA). Antibody of TGF‐β1 was from Affinity (AF1027). Antibodies of anti‐HMGCS2 (A14244, for western blot, IHC and IF), anti‐immunity related GTPase M (IRGM, A10968), anti‐isocitrate dehydrogenase (NADP (+)) 1 (IDH1, A2169), anti‐sorbitol dehydrogenase (SORD, A2118), anti‐glutathione S‐transferase pi 1 (GSTP1, A5691), anti‐argininosuccinate synthase 1 (ASS1, A2012), anti‐malate dehydrogenase 1 (MDH1, A7563), anti‐death associated protein (DAP, A6150) and anti‐HA (AE008) were from ABclonal (Wuhan, China). Anti‐Collagen III (bs‐0549R), Collagen I (bs‐10423R), β‐actin (bs‐0061R) and GAPDH (bs‐2188R) were purchased from Bioss (Beijing, China). Recombinant Human HMGCS2 protein (ab114900) and TFAM protein (ab103784) were from Abcam. TGF‐β1 (100‐21‐10μg) was from Pepro Tech (Rocky Hill, USA).

### Patients

Renal biopsy samples were obtained from CKD patients who were undergoing diagnostic evaluation at the Department of Nephrology, Children's Hospital of Nanjing Medical University. Renal biopsy samples were collected based on the criterion of having at least 10 glomeruli in a paraffin‐embedded tissue sample available for histological sectioning. A total of 30 subjects (age range 3–14 years old) were enrolled and mostly pathologically diagnosed with IgA nephropathy. The patient information was listed in Table EV1. Normal renal tissues were collected from patients without proteinuria who received a partial nephrectomy of a benign renal tumor.

### Cell culture and intervention

The HK‐2 (obtained from FuHeng Biology Cell Bank, Shanghai, China, cat: FH0228) or mPTC (obtained from ATCC, cat: CRL‐3361) cell line was maintained in DMEM‐F12 medium containing 10% FBS. The rat kidney fibroblast cell line NRK‐49F (obtained from ATCC, cat: CRL‐1570) was maintained in DMEM medium containing 10% FBS. All cells were cultured at 37°C with 5% CO2. The cell lines were authenticated by STR profiling. The cells were transfected with *Lonp1* or *HMGCS2* plasmids or shRNA using lipo3000 (cat: L3000015, ThermoFisher, IL, USA) according to the manufacturer's instructions and then stimulated with TGF‐β1 at a concentration of 10 ng/ml or control buffer (vehicle) for 24 h. The human shLONP1 plasmids were obtained from Santa Cruze (sc‐97290‐SH) and the mouse sh*Lonp1* plasmids were obtained from RiboBio (Guangzhou, China. Sequence: GCTGCATACAAGATCGTAA). The mouse sh*Hmgcs2* plasmids were from PPL (Public Protein/Plasmid Library, China. Sequence: CGACTTCTACAAACCAAACTT). For pharmaceutic intervention alone, cells were treated with 5, 10 or 20 μM 84‐B10 for 24 h; for the pharmaceutic intervention combining with TGF‐β1, cells were pre‐incubated with 2.5, 5, 10 or 20 μM 84‐B10 or vehicle (0.1% DMSO) for 2 h before stimulated with TGF‐β1 (5 or 10 ng/ml) or vehicle for 24 h; while for testing the LONP1 dependent effect of 84‐B10 intervention, the cells were transfected with *Lonp1* siRNA (cat:siG1512031128‐28/34/41, RiboBio, Guangzhou, China) using lipo2000 (cat:11668500, ThermoFisher, IL, USA) according to the manufacturer's instructions. Then the cells were stimulated with 10 μM 84‐B10 or vehicle for 2 h before stimulated with TGF‐β1 (5 or 10 ng/ml) or vehicle for 24 h.

### Primary proximal tubular cell culture and intervention

We isolated primary proximal tubular cells from WT and Lonp1 cKI mice and then treated the cells with TGF‐β1 at a concentration of 10 ng/ml or control buffer (vehicle) for 24 h. We also isolated the primary proximal tubular cells from C57BL/6J mice and infected with *Hmgcs2*‐lentivirus (pLVX‐Puro‐mHmgcs2‐HA) or control lentivirus (vector) (obtained from Public Protein/Plasmid Library, China). Then the cells stimulated with TGF‐β1 at a concentration of 10 ng/ml or control buffer (vehicle) for 24 h.

### Animal

#### Generation of a renal proximal tubular *Lonp1* conditional knockin mouse (cKI) strain

The *Lonp1* ^*flox/flox*^ knock‐in mice (genetic background: C57BL/6J) were purchased from Beijing Viewsolid Biotech Co., Ltd. Using the CRISPR system, insert the transcription termination signal original STOP between the pCAG promoter and *Lonp1* sequence, construct the pCAG‐loxp‐STOP‐loxp‐Lonp1 skeleton vector and transfer it into the male pronucleus of the fertilized egg to construct the transgenic mice, and then crossed with the Kap‐Cre transgenic mice (The Jackson Laboratory, Stock No: 008781, genetic background: C57BL/6J) to generate F1. Then, F1 mated backcross with the *Lonp1* ^*flox/flox*^ knock‐in mice to generate renal proximal tubular *Lonp1* conditional knock‐in (cKI) mice.

#### Generation of a renal proximal tubular *Lonp1* conditional knockout mouse (cKO) strain

The *Lonp1* ^*flox/flox*^ knock‐out mice (genetic background: C57BL/6J) were presented from professor Lu Bin (Wen Zhou Medical University, China). The *Lonp1* ^*flox/flox*^ mice mated with mice carrying the Kap‐Cre transgenic mice to generate F1 (*Lonp1* ^flox/+^; Kap‐Cre+). Then, F1 mated backcross with the *Lonp1* ^*flox/flox*^ mice to generate renal proximal tubular *Lonp1* conditional knockout (*Lonp1* ^flox/flox^; Kap‐Cre+, cKO) mice.

#### Generation of *Hmgcs2* ^+/−^ mouse strain

Hmgcs2‐KO mice (Strain NO.T004234, genetic background: C57BL/6J) were purchased from GemPharmatech (Nanjing, China). Heterozygous F0 generation mice were obtained by freezing sperm resuscitation. After hybridization of F0 generation mice, a sufficient number of F1 generation heterozygous mice (*Hmgcs2* ^+/−^) were obtained for experiment (8 weeks old, male and female).

#### Unilateral ureteral obstruction (UUO) model

In the experiment, following anesthesia, the left ureter was ligated at the ureteropelvic junction with a 4–0 silk suture through a left flank incision. Mice were sacrificed after 7 or 14 days.

#### Five‐sixths nephrectomy (5/6Nx) model

The 5/6Nx was performed in 12–16 weeks old male WT and cKO mice through one steps as described previously.(Souza *et al*, 2015) In detail, the left kidney was decapsulated via a left back flank incision to respect the upper and lower poles (silk ligature before resecting was used to control bleeding). Next, the entire right kidney via a right flank incision. After 12 weeks, the blood pressure of the mice was measured by the computerized tail‐cuff system (BP‐2000; Visitech Systems, Apex, NC) according to the manufacturer's specifications. The urine was collected and analyzed for urine protein and creatinine using automatic biochemical analyzer.

#### Unilateral ischemia/reperfusion injury (UIRI) model

In the experiment, male mice were anesthetized, and the left kidney was exposed through a median ventral incision. Ischemia was induced by clamping the renal pedicle with nontraumatic microaneurysm clamps for 45 min. During the experiment, body temperature of mice was controlled at 36.5–37.5°C.

#### Pharmaceutical treatment

Wild type C57BL/6J mice (7 weeks old, male) were purchased from the Model Animal Research Center of Nanjing University (Nanjing, China) and were allowed to acclimate to the housing environment for a week. 84‐B10 was dissolved in DMSO at as a stock solution of 20 mM and stored at −80°C. For intervention of UUO model, the mice were pretreated with 84‐B10 at 0.5 mg/kg/day (low dose, LD) and 7.5 mg/kg/day (high dose, HD) via intraperitoneal (i.p.) injection 24 and 2 h before UUO surgery. Then the mice were treated daily for 7 consecutive days and sacrificed 2 h after the final injection. For intervention of 5/6Nx model, the mice were treated with 84‐B10 at 2.5 mg/kg/day (LD), 5 (mid dose, MD) and 10 mg/kg/day (HD) once daily via i.p. injection from day 15 to 56 after nephrectomy. Mice were sacrificed 2 h after the final injection. For intervention of UIRI, the mice were treated with 84‐B10 at 2.5 mg/kg/day (LD) and 5 mg/kg/day (HD) via i.p. injection 72 h after UIR surgery. The mice were treated daily for 19 consecutive days and sacrificed 2 h after the final injection.

All mice were maintained on a 12‐h light–dark cycle in a temperature‐controlled (19–21°C) room, were fed a standard rodent diet and were allowed free access to drinking water.

All animal protocols and procedures were approved by the Institutional Animal Care and Use Committee at Nanjing Medical University, China.

### Immunohistochemical staining

Kidneys were fixed in 4% paraformaldehyde and embedded in paraffin. Paraffin sections of each specimen were cut at a thickness of 3 μm, and a standard protocol, using xylene and graded ethanol, was employed to deparaffinize and rehydrate the tissue. The sections were incubated in 3% hydrogen peroxide for 20 min, boiled in modified Citrate Antigen Retrieval Solution (Beyotime, P0086) for 10 min. The slides were then incubated overnight at 4°C with LONP1 (1:500) or HMGCS2 (1:100). After washing with phosphate buffer saline, the biotin‐conjugated secondary antibody was applied, and the signals were detected using Dako REAL™ EnVision™ Detection System K5007 (Denmark).

### Masson trichrome staining

Harvested kidney tissues from mice were fixed with 4% paraformaldehyde, embedded in paraffin, and sectioned transversely. After dewaxing and gradient ethanol hydration, kidney sections (2 μm) were stained with Weigert's hematoxylin solution for 10 min, rinsed in distilled water for 5 min, stained with Masson ponceau acid fuchsin solution for 10 min, rinsed in distilled water for 2 min, placed in 1% phosphomolybdic acid solution for 5 min, and immersed in aniline blue solution for 5 min. Collagenous matrix was stained green or blue by Masson staining. The renal pathological changes were observed under microscope.

### Sirius Red staining

Sirius Red staining kit was from G‐CLONE (cat: RS1220, Beijing, China). Sirius Red staining was completed according to the manufacturer's instructions. Kidney tissues were fixed by immersion in 4% paraformaldehyde for 24 h, routinely dehydrated and paraffin‐embedded. These sections were 3 μm thick and then dewaxed in xylene and gradually hydrated in gradient ethanol. The slides were stained with drops of Sirius Red stain for 1 h and rinsed twice with water. After dehydration with 95 and 100% ethanol and transparency with xylene, the slides were sealed with neutral gum.

### SDH staining

SDH activity staining kit for frozen sections was from GENMED SCIENTIFICS (cat: GMS80042.2, Shanghai, China). SDH staining was completed according to the manufacturer's instructions. Frozen tissue sections of 10 μm thickness were prepared. Add 50 μl GENMED cleaning solution to cover the whole sample surface and remove the cleaning solution, add 50 μl GENMED working solution, and incubate in 37°C incubator for 10 min, away from light. After wash for three times using cleaning solution, add 50 μl of fixative solution for 15 min at room temperature. After wash for three times using cleaning solution, mount the slides with neutral gum and photographed under the microscope.

### Mitochondrial complex activity

Mitochondrial OXPHOS complex I enzyme activity was measured by a Complex I (Abcam, cat: ab109721) Enzyme Activity Microplate Assay Kit according to the manufacturers' protocols. Briefly, isolated mitochondria samples were prepared using mitochondrial lysis kit (GENMED, cat: GMS10018V.A) at final protein appropriate concentration respectively, and the samples were loaded to the wells of the microplate. The microplate was incubated and then assay solution was added to each well. Optical density was measured in a kinetic mode at appropriate temperature for suitable minutes.

### Transmission electron microscopy

To evaluate the mitochondrial morphology in mouse renal cortex, tissues were collected and fixed in 1.25% glutaraldehyde‐0.1 mol/l phosphate buffer. After postfixing in 1% OsO4‐0.1 mol/l phosphate buffer, ultrathin sections (60 nm) were cut on a microtome, placed on copper grids, stained with uranyl acetate and lead citrate, and examined in an electron microscope (JEOL JEM‐1010; Tokyo, Japan) as previously described (Gong *et al*, 2016).

### RNA extraction and real‐time quantitative PCR (qRT‐PCR)

Total RNA from cultured cells and renal tissues were extracted by Trizol reagent (cat: 9109, TaKaRa Biotechnology, Japan) according to the manufacturer's instructions. Reverse transcription was performed using HiScript II Q RT SuperMix for qPCR (cat: R222‐01, Vazyme Biotechnology, Nanjing, China) according to the manufacturer's instruction. The qRT‐PCR was performed using AceQ qPCR SYBR Green Master Mix (cat: Q131‐02, Vazyme Biotechnology) on the ABI Prism 7500 Sequence Detection System (Foster City, USA). The temperature cycling conditions were 95°C for 10 min followed by 40 cycles of 95°C for 15 s and 60°C for 1 min. The relative expression level of mRNA was normalized to the relative expression of GAPDH and calculated using the 2^−ΔΔCt^ method. The primers were designed by Primer 5.0 software (http://Frodo.wi.mit.edu) and the primer sequences are shown in Table EV2 and Appendix Table S1.

### Western blotting assays

Cells and renal tissues were lysed in RIPA buffer (cat: P0013K, Beyotime, Shanghai, China) containing the protease inhibitor cocktail (cat: 04693132001, Roche, Basel, Switzerland). Lysates were separated by SDS–PAGE. BCA protein assay kit (cat: 23227, ThermoFisher) was used to determine the protein concentration. Equivalent samples were used for primary antibodies against LONP1 (1:1,000), FN1 (1:1,000), Collagen I (1:1,000), Collagen III (1:1,000), α‐SMA (1:3,000), TGF‐β1 (1:1,000), Vimentin (1:1,000), HMGCS2 (1:1,000), IRGM (1:1,000), IDH1 (1:1,000), SORD (1:1,000), GSTP1 (1:1,000), ASS1 (1:1,000), MDH1 (1:1,000), DAP (1:1,000), COX IV (1:1,000), TFAM (1:1,000), ACOX1 (1:1,000), anti‐ACADM (1:1,000) and GAPDH (1:5,000), and then added HRP‐labeled secondary antibody (1:5,000). The blots were visualized with Amersham ECL Detection Systems (Amersham, Buckinghamshire, UK). Densitometric analysis was performed using Image Lab Software (Bio‐Rad, USA).

### Oxygen consumption rate

An assay was performed using the Seahorse XF‐96 Extracellular Flux Analyzer (Seahorse Bioscience, Copenhagen, Denmark) to measure the oxygen consumption rate (OCR) as previously described (Bai *et al*, 2019). Briefly, HK2 cells were initially transfected with *Lonp1* plasmid or shRNA. On the day of the experiment, the cell medium was replaced by Seahorse assay medium, and cells were incubated at 37°C in a CO_2_‐free incubator for 1 h prior to assessing the basal OCR. Inhibitors were prepared in the same medium at a 10× concentration, and the injection ports of the sensors were filled. Half an hour before the experiment, the sensor was placed into the XF‐96 instrument, and calibration was initiated. After calibration, the calibration fluid plate was replaced with the cell plate, and measurements began. Basal oxygen consumption was recorded for 20 min, and OCR measurements were performed over time after the successive addition of mitochondrial inhibitors: (i) Oligomycin (1 μM), which blocks the proton channel of the Fo portion of ATP synthase (Complex V) and thus inhibits ATP synthesis, was employed to determine the ATP‐synthesis coupling efficiency. (ii) The uncoupler carbonyl cyanide 4‐trifluoromethoxy‐phenylhydrazone (FCCP) (2 μM) was added to calculate the spare respiratory capacity. (iii) Finally, a mixture of rotenone (0.5 μM) and antimycin A (0.5 μM), Complex I and Complex III inhibitors, respectively, were used to assess the non‐mitochondrial consumption of O2. The compounds were injected automatically by the XF‐96 instrument into each well. The determined cellular bioenergetics parameters were the following: basal respiration, coupling efficiency, and spare respiratory capacity.

### Analysis of mitchondrial ROS production, mitochondrial membrane potential (MMP), and mitochondria DNA (mtDNA) copy number

Reactive oxygen species production in cells was measured by MitoSOX (cat: M36008, ThermoFisher) as described previously (Yuan *et al*, 2012). The mitochondrial MMP in HK‐2 was determined using the lipophilic cationic probe 5,5′,6,6′‐tetrachloro‐1,1′,3,3′‐tetraethyl‐benzimidazolcarbocyanine iodide (JC‐1, cat: FS1166, FuShen biotechnology, Shanghai, China). JC‐1 dye exhibits potential‐dependent accumulation in mitochondria, indicated by a fluorescence emission shift from green (~529 nm) to red (~590 nm). Consequently, mitochondrial depolarization is indicated by a decrease in the red/green fluorescence intensity ratio. JC‐1 fluorescence levels were analyzed by flow cytometry to quantitate MMP levels. mtDNA was extracted by commercial kit (cat: DP304‐03, TIANGEN, Beijing, China) according to the manufacturer's instructions. Mitochondrial DNA (mtDNA) copy number was detected by qRT‐PCR and calculated through delta–delta Ct method; 18s rRNA was used as the internal control for mice and human respectively.

### Co‐immunoprecipitation

Binding buffer and wash buffer: 50 mM Tris, 150 mM NaCl, 0.25% Tween 20, pH 7.5; elution buffer: 0.1–0.2 M Glycine, 0.25% Tween 20, pH 2.5–3.1; neutralize buffer: 1 M Tris, pH 8.0.

293T cells were transfected with LONP1‐Flag and HMGCS2 plasmids at 70% fusion, and treated with protease inhibitor Bortezomib (Selleck, cat: S1013) for 4 h before cell collection. Cells were lysed with binding buffer supplemented with protease inhibitor cocktail. Whole cell lysate was stored on ice for the following step. Transfer 50 μl protein A/G magnetic bead slurry (Bimake, cat: B23201) to a 1.5 ml tube, and then wash the slurry with 200 μl binding buffer. The magnetic beads were premixed with the whole cell lysate, and were reversed at 4°C for 1–2 h. Discard the beads after treatment. Then whole‐cell lysates were mixed with mouse anti‐flag antibody (20 μg/ml) or normal mouse IgG for 2 h at 4°C. The newly processed beads slurry (50 μl) was subsequently added and mixed with whole‐cell lysates for overnight at 4°C. The second day, the beads were washed three times with wash buffer. Samples were eluted into 50 μl 1 × SDS Loading Buffer (Beyotime, cat: P0015L) and analyzed by western blot.

### GST pull‐down

We used previously purified protein LONP1‐GST synthesized by Gene Universal (Anhui, China) as the bait protein, and the prey protein HMGCS2 was from 293T cell lysates. The experimental procedure was carried out according to the Pierce™ GST Protein Interaction Pull‐Down Kit (ThermoFisher, cat: 21516).

### Immunofluorescence staining

mPTCs were cultured on glass‐bottom cell culture dishes (NEST, China) and co‐transfected with LONP1‐Flag and HMGCS2 plasmids. Then, after several washes with PBS, the cells were fixed with 4% paraformaldehyde for 20 min at room temperature, extensively washed with PBS, permeabilized with PBS + 1% Triton X‐100 for 10 min and blocked with 2% BSA for 1 h. The primary antibodies Flag and HMGCS2 were added to the dish at a 1:100 dilution and incubated in a humid chamber overnight at 4°C; the secondary antibodies were applied after washing with PBST. Then, the cells were viewed under a laser scanning confocal microscope (Zeiss), photographed and recorded.

### Molecular docking

Although the X‐ray structure of human LONP1 catalytic domain was resolved (PDB ID: 2X36) (Garcia‐Nafria *et al*, 2010), the active site in this structure presented a closed conformation. So a homology model of human LONP1 catalytic domain in the active conformation was built based on the crystal structure of the hexameric LonA protease of Meiothermus taiwanensis (PDB ID: 4YPL) (Lin *et al*, 2016) by Prime, and prepared as the receptor for the virtual screening of the TargetMol database and Maybridge‐hitfinder database with 19,826 compounds in total. The compounds were prepared and docked into the receptor at the SP precision by Glide. The docked ligand‐protein complexes in 3D and the 2D ligand‐protein interaction diagrams were presented by Maestro.

### LONP1 protease activity assay

#### Substrate protein‐based protease activity assay

Recombinant human LONP1 carrying a GST‐tag was overproduced and purified from Escherichia coli. Substrate protein based protease activity assay was carried out in cell‐free system containing recombinant human LONP1‐GST (800 nM), recombinant human HMGCS2 protein (60 nM) or recombinant human TFAM‐His (Abcam, cat: ab103784) (60 nM), 25 mM Tris, pH 7.9, 10 mM MgCl_2_, 0.1 mg/ml BSA, 2 mM ATP (the negative control group [NC] was not supplemented with ATP). Samples were incubated at 37°C for 30 min. Enzyme activity of LONP1 is estimated by detecting the remaining protein levels of HMGCS2 or TFAM with immunoblotting. For the drug screening, candidates were incubated with recombinant LONP1‐GST protease and recombinant human TFAM‐His at 37°C for 30 min before adding of ATP.

#### Fluorogenic dipeptide substrate‐based protease activity assay

Purified LONP1‐GST (800 nM) were pre‐incubated in the absence or presence of 84‐B10 in reaction buffer (150 mM NaCl, 50 mM Hepes pH 8.0, 10 mM MgCl_2_) at 37°C for 30 min before dipeptide substrate benzyloxycarbonyl‐alanyl‐alanine amide conjugated with fluorescent dye Rhodamine110 (Z‐AA)_2_‐Rh110 (Anaspec, cat: AS‐60320) (6 μM) and ATP (2 mM) were added. Reactions were carried out at 37°C for 1 h. After cleavage, the colorless and nonfluorescent (Z‐AA)_2_‐Rh110 is hydrolyzed to highly fluorescent Rh110, which exhibits fluorescence that can be detected at excitation/emission = 497 nm/527 nm.

### Surface plasmon resonance (SPR)

The binding affinity of 84‐B10 with Lonp1 were determined using the Biacore T200 SPR biosensor systems with a CM5 sensor chip (GE Healthcare, Chicago, IL). The CM5 chip surface was first activated with 0.1 M N‐hydroxysuccinimide and 0.4 M N‐ethyl‐N′‐(3‐dimethylaminopropyl) carbodiimide at a flow rate of 30 μl/min using PBS supplemented with 0.01% Tween 20 as the running buffer. Next, recombinant human LONP1‐GST was diluted to 20 μg/ml in 10 mM sodium acetate (pH 4.5) and injected on the first flow cell until a density of approximately 1,000 response units (RU) was immobilized. Activated amine groups were quenched with an injection of 1 M ethanolamine (pH 8.0). Then, 84‐B10 was diluted from 10 to 0.3125 μM in PBS/0.01% Tween 20 buffer, and injected to the flow cell at 30 μl/min. The association time was set to 120 s, and the dissociation time was 120 s. Equilibrium constants (KD) were calculated using the “affinity” model in Biacore T200 evaluation software (GE Healthcare).

### Pharmacokinetics and tissue distribution of 84‐B10 in mice

Plasma pharmacokinetic (PK) profiles and tissue distribution of 84‐B10 in C57BL/6J mice (8 weeks old, male) were investigated after a single intraperitoneal injected of 84‐B10 (5 mg/kg). For PK analysis, the blood samples were collected 2, 5, 15, 30 min, 1, 2, 4, 6, 10, 24 h after treatment. For tissue distribution analysis, heart, kidney, liver, lung, brain, small intestine, muscle, and fat tissues were collected 30 min after treatment. Plasma samples were collected by centrifuging the blood at 8,000 rpm for 5 min and tissues (0.2 g) was homogenized with 1 ml saline. The plasma and tissue homogenate were then precipitated with methanol containing ginkgolide J (2 μg/ml) as internal standard. After precipitation and centrifugation for twice, 200 μl of the supernatant was transferred for analysis.

The concentrations of 84‐B10 in bio samples were determined by ExionLC system coupled with 4,500 triple quadrupole mass spectrometers (Sciex, Framingham, MA, USA). A 2 μl aliquot of samples was injected onto the HPLC system using a Phenyl HPLC column (50 × 2.1 mm, 3 μm) (YMC, Kyoto, Japan). The mobile phases A consist of 5 mM ammonium formate in water, and phase B is methanol. Phase A and B were mixed and delivered at a flow rate of 300 μl/min with a gradient program as follows: 0.1 min, 20% B; 0.3 min, 95% B; 1.8 min, 95% B; 1.9 min, 20% B; 3 min, stop. Multiple reaction monitoring (MRM) mode was used for mass detection. The optimized MRM fragmentation transitions for 84‐B10 was *m/z* 472.0 → *m/z* 282.0 (with a collision energy of −30 eV and declustering potential of −120 eV) and for internal standard was *m/z* 423.0 → *m/z* 349.0 (with a collision energy of −40 eV and declustering potential of −130 eV).

### Statistical analysis

Data are expressed as mean ± SEM. Quantifications of images were performed by ImageJ or Image‐Pro Plus (IPP). Statistical analysis was performed with GraphPad Prism (version 7.0, GraphPad Software, SanDiego, CA). A two‐tailed Student's *t*‐test was used to compare differences between two groups. For comparisons among multiple groups, one‐way ANOVA was applied. Pearson correlation analysis was also used. All statistical details regarding *P*‐value and *n* can be found in main and expanded figure legends. We collected data from animal studies in a blinded manner. No animals were excluded from the study. The sample size in each study was based on experience with previous studies employing CKD animals and knockout mice in our lab.

## Author contributions

**Mi Bai:** Data curation; funding acquisition; validation; investigation; visualization; methodology; writing – original draft. **Mengqiu Wu:** Data curation; formal analysis; visualization; methodology; writing – original draft. **Mingzhu Jiang:** Data curation; investigation; visualization. **Jia He:** Data curation; investigation; methodology. **Xu Deng:** Data curation; funding acquisition; investigation; methodology. **Shuang Xu:** Investigation; methodology. **Jiaojiao Fan:** Investigation; methodology. **Mengqiu Miao:** Investigation; methodology. **Ting Wang:** Investigation; methodology. **Yuting Li:** Methodology. **Xiaowen Yu:** Funding acquisition; methodology. **Lin Wang:** Methodology. **Yue Zhang:** Validation; investigation. **Songming Huang:** Funding acquisition; validation; investigation. **Li Yang:** Validation; investigation. **Zhanjun Jia:** Conceptualization; formal analysis; funding acquisition; validation; investigation; writing – review and editing. **Aihua Zhang:** Conceptualization; formal analysis; funding acquisition; validation; investigation; writing – review and editing.

## Disclosure and competing interest statement

The authors declare that they have no conflict of interest.

## For more information

i Kidney Interactive Transcriptomics: http://www.humphreyslab.com/SingleCell/

## Supporting information



Appendix

Expanded View Figures PDF

Table EV1

Table EV2

Source Data for Expanded View and Appendix

PDF+

Source Data for Figure 1

Source Data for Figure 2

Source Data for Figure 3

Source Data for Figure 4

Source Data for Figure 5

Source Data for Figure 6

Source Data for Figure 7

Source Data for Figure 8

## Data Availability

The mass spectrometry proteomics data have been deposited to the ProteomeXchange Consortium via the PRIDE partner repository with the dataset identifier PXD037552 (http://www.ebi.ac.uk/pride/archive/projects/PXD037552).

## References

[emmm202216581-bib-0001] Bahat A , Perlberg S , Melamed‐Book N , Isaac S , Eden A , Lauria I , Langer T , Orly J (2015) Transcriptional activation of LON Gene by a new form of mitochondrial stress: a role for the nuclear respiratory factor 2 in StAR overload response (SOR). Mol Cell Endocrinol 408: 62–72 25724481 10.1016/j.mce.2015.02.022

[emmm202216581-bib-0002] Bai M , Chen H , Ding D , Song R , Lin J , Zhang Y , Guo Y , Chen S , Ding G , Zhang Y *et al* (2019) MicroRNA‐214 promotes chronic kidney disease by disrupting mitochondrial oxidative phosphorylation. Kidney Int 95: 1389–1404 30955870 10.1016/j.kint.2018.12.028

[emmm202216581-bib-0003] Bota DA , Davies KJ (2001) Protein degradation in mitochondria: implications for oxidative stress, aging and disease: a novel etiological classification of mitochondrial proteolytic disorders. Mitochondrion 1: 33–49 16120267 10.1016/s1567-7249(01)00005-8

[emmm202216581-bib-0004] Bota DA , Davies KJ (2002) Lon protease preferentially degrades oxidized mitochondrial aconitase by an ATP‐stimulated mechanism. Nat Cell Biol 4: 674–680 12198491 10.1038/ncb836

[emmm202216581-bib-0005] Bueno M , Lai YC , Romero Y , Brands J , St Croix CM , Kamga C , Corey C , Herazo‐Maya JD , Sembrat J , Lee JS *et al* (2015) PINK1 deficiency impairs mitochondrial homeostasis and promotes lung fibrosis. J Clin Invest 125: 521–538 25562319 10.1172/JCI74942 PMC4319413

[emmm202216581-bib-0006] Cheng CW , Biton M , Haber AL , Gunduz N , Eng G , Gaynor LT , Tripathi S , Calibasi‐Kocal G , Rickelt S , Butty VL *et al* (2019) Ketone body signaling mediates intestinal stem cell homeostasis and adaptation to diet. Cell 178: 1115–1131 31442404 10.1016/j.cell.2019.07.048 PMC6732196

[emmm202216581-bib-0007] Chevalier RL (2016) The proximal tubule is the primary target of injury and progression of kidney disease: role of the glomerulotubular junction. Am J Physiol Renal Physiol 311: F145–F161 27194714 10.1152/ajprenal.00164.2016 PMC4967168

[emmm202216581-bib-0008] Conway BR , O'Sullivan ED , Cairns C , O'Sullivan J , Simpson DJ , Salzano A , Connor K , Ding P , Humphries D , Stewart K *et al* (2020) Kidney single‐cell atlas reveals myeloid heterogeneity in progression and regression of kidney disease. J Am Soc Nephrol 31: 2833–2854 32978267 10.1681/ASN.2020060806 PMC7790206

[emmm202216581-bib-0009] Denby L , Conway B , Hughes J , Cairns C (2020) Gene Expression Omnibus GSE145053 (https://www.ncbi.nlm.nih.gov/geo/query/acc.cgi?acc=GSE145053). [DATASET]

[emmm202216581-bib-0010] Fang L , Li TS , Zhang JZ , Liu ZH , Yang J , Wang BH , Wang YM , Zhou J , Kong LD (2021) Fructose drives mitochondrial metabolic reprogramming in podocytes via Hmgcs2‐stimulated fatty acid degradation. Signal Transduct Target Ther 6: 253 34238920 10.1038/s41392-021-00570-y PMC8266798

[emmm202216581-bib-0011] Garcia‐Nafria J , Ondrovicova G , Blagova E , Levdikov VM , Bauer JA , Suzuki CK , Kutejova E , Wilkinson AJ , Wilson KS (2010) Structure of the catalytic domain of the human mitochondrial Lon protease: proposed relation of oligomer formation and activity. Protein Sci 19: 987–999 20222013 10.1002/pro.376 PMC2868241

[emmm202216581-bib-0012] Geisler CE , Ghimire S , Bogan RL , Renquist BJ (2019) Role of ketone signaling in the hepatic response to fasting. Am J Physiol Gastrointest Liver Physiol 316: G623–G631 30767679 10.1152/ajpgi.00415.2017 PMC6580236

[emmm202216581-bib-0013] Ghosh JC , Seo JH , Agarwal E , Wang Y , Kossenkov AV , Tang HY , Speicher DW , Altieri DC (2019) Akt phosphorylation of mitochondrial Lonp1 protease enables oxidative metabolism and advanced tumor traits. Oncogene 38: 6926–6939 31406245 10.1038/s41388-019-0939-7 PMC6814529

[emmm202216581-bib-0014] Gibellini L , Losi L , De Biasi S , Nasi M , Lo Tartaro D , Pecorini S , Patergnani S , Pinton P , De Gaetano A , Carnevale G *et al* (2018) LonP1 differently modulates mitochondrial function and bioenergetics of primary versus metastatic colon cancer cells. Front Oncol 8: 254 30038898 10.3389/fonc.2018.00254 PMC6046640

[emmm202216581-bib-0015] Gong W , Mao S , Yu J , Song J , Jia Z , Huang S , Zhang A (2016) NLRP3 deletion protects against renal fibrosis and attenuates mitochondrial abnormality in mouse with 5/6 nephrectomy. Am J Physiol Renal Physiol 310: F1081–F1088 26887832 10.1152/ajprenal.00534.2015

[emmm202216581-bib-0016] Gong W , Song J , Liang J , Ma H , Wu W , Zhang Y , Yang L , Huang S , Jia Z , Zhang A (2021) Reduced Lon protease 1 expression in podocytes contributes to the pathogenesis of podocytopathy. Kidney Int 99: 854–869 33181155 10.1016/j.kint.2020.10.025

[emmm202216581-bib-0017] Hamon MP , Bulteau AL , Friguet B (2015) Mitochondrial proteases and protein quality control in ageing and longevity. Ageing Res Rev 23: 56–66 25578288 10.1016/j.arr.2014.12.010

[emmm202216581-bib-0018] Han JY , Kim JS , Son JH (2014) Mitochondrial homeostasis molecules: regulation by a trio of recessive Parkinson's disease genes. Exp Neurobiol 23: 345–351 25548534 10.5607/en.2014.23.4.345 PMC4276805

[emmm202216581-bib-0019] Jadiya P , Tomar D (2020) Mitochondrial protein quality control mechanisms. Genes 11: 563 32443488 10.3390/genes11050563 PMC7290828

[emmm202216581-bib-0020] Jiang M , Bai M , Lei J , Xie Y , Xu S , Jia Z , Zhang A (2020) Mitochondrial dysfunction and the AKI‐to‐CKD transition. Am J Physiol Renal Physiol 319: F1105–F1116 33073587 10.1152/ajprenal.00285.2020

[emmm202216581-bib-0021] Kalvala AK , Yerra VG , Kumar A (2020) LONP1 induction by SRT1720 attenuates mitochondrial dysfunction against high glucose induced neurotoxicity in PC12 cells. Toxicol In Vitro 62: 104695 31639451 10.1016/j.tiv.2019.104695

[emmm202216581-bib-0022] Kang HM , Ahn SH , Choi P , Ko YA , Han SH , Chinga F , Park AS , Tao J , Sharma K , Pullman J *et al* (2015) Defective fatty acid oxidation in renal tubular epithelial cells has a key role in kidney fibrosis development. Nat Med 21: 37–46 25419705 10.1038/nm.3762 PMC4444078

[emmm202216581-bib-0023] Kim JT , Li C , Weiss HL , Zhou Y , Liu C , Wang Q , Evers BM (2019) Regulation of ketogenic enzyme HMGCS2 by Wnt/beta‐catenin/PPARgamma pathway in intestinal cells. Cell 8: 1106 10.3390/cells8091106 PMC6770209 31546785

[emmm202216581-bib-0024] Lee HJ , Chung K , Lee H , Lee K , Lim JH , Song J (2011) Downregulation of mitochondrial lon protease impairs mitochondrial function and causes hepatic insulin resistance in human liver SK‐HEP‐1 cells. Diabetologia 54: 1437–1446 21347624 10.1007/s00125-011-2074-z

[emmm202216581-bib-0025] Leung KC , Tonelli M , James MT (2013) Chronic kidney disease following acute kidney injury‐risk and outcomes. Nat Rev Nephrol 9: 77–85 23247572 10.1038/nrneph.2012.280

[emmm202216581-bib-0026] Lin CC , Su SC , Su MY , Liang PH , Feng CC , Wu SH , Chang CI (2016) Structural insights into the allosteric operation of the Lon AAA+ protease. Structure 24: 667–675 27041592 10.1016/j.str.2016.03.001

[emmm202216581-bib-0027] Liu Y (2011) Cellular and molecular mechanisms of renal fibrosis. Nat Rev Nephrol 7: 684–696 22009250 10.1038/nrneph.2011.149 PMC4520424

[emmm202216581-bib-0028] Liu X , Miao J , Wang C , Zhou S , Chen S , Ren Q , Hong X , Wang Y , Hou FF , Zhou L *et al* (2020) Tubule‐derived exosomes play a central role in fibroblast activation and kidney fibrosis. Kidney Int 97: 1181–1195 32139089 10.1016/j.kint.2019.11.026

[emmm202216581-bib-0029] Lu B (2017) Mitochondrial Lon protease and cancer. Adv Exp Med Biol 1038: 173–182 29178076 10.1007/978-981-10-6674-0_12

[emmm202216581-bib-0030] Lu B , Liu T , Crosby JA , Thomas‐Wohlever J , Lee I , Suzuki CK (2003) The ATP‐dependent Lon protease of *Mus musculus* is a DNA‐binding protein that is functionally conserved between yeast and mammals. Gene 306: 45–55 12657466 10.1016/s0378-1119(03)00403-7

[emmm202216581-bib-0031] Lu B , Lee J , Nie X , Li M , Morozov YI , Venkatesh S , Bogenhagen DF , Temiakov D , Suzuki CK (2013) Phosphorylation of human TFAM in mitochondria impairs DNA binding and promotes degradation by the AAA+ Lon protease. Mol Cell 49: 121–132 23201127 10.1016/j.molcel.2012.10.023 PMC3586414

[emmm202216581-bib-0032] Matsushima Y , Goto Y , Kaguni LS (2010) Mitochondrial Lon protease regulates mitochondrial DNA copy number and transcription by selective degradation of mitochondrial transcription factor A (TFAM). Proc Natl Acad Sci USA 107: 18410–18415 20930118 10.1073/pnas.1008924107 PMC2972957

[emmm202216581-bib-0033] Meng XM , Nikolic‐Paterson DJ , Lan HY (2014) Inflammatory processes in renal fibrosis. Nat Rev Nephrol 10: 493–503 24981817 10.1038/nrneph.2014.114

[emmm202216581-bib-0034] Miguel V , Tituana J , Herrero JI , Herrero L , Serra D , Cuevas‐Delgado P , Barbas C , Rodriguez‐Puyol D , Marquez‐Exposito L , Ruiz‐Ortega M *et al* (2021) Renal tubule Cpt1a overexpression protects from kidney fibrosis by restoring mitochondrial homeostasis. J Clin Invest 131: e140695 33465052 10.1172/JCI140695 PMC7919728

[emmm202216581-bib-0035] Mooli RGR , Ramakrishnan SK (2022) Emerging role of hepatic ketogenesis in fatty liver disease. Front Physiol 13: 946474 35860662 10.3389/fphys.2022.946474 PMC9289363

[emmm202216581-bib-0036] Ngo JK , Pomatto LC , Bota DA , Koop AL , Davies KJ (2011) Impairment of lon‐induced protection against the accumulation of oxidized proteins in senescent wi‐38 fibroblasts. J Gerontol A Biol Sci Med Sci 66: 1178–1185 21868393 10.1093/gerona/glr145 PMC3193527

[emmm202216581-bib-0037] Onat UI , Yildirim AD , Tufanli O , Cimen I , Kocaturk B , Veli Z , Hamid SM , Shimada K , Chen S , Sin J *et al* (2019) Intercepting the lipid‐induced integrated stress response reduces atherosclerosis. J Am Coll Cardiol 73: 1149–1169 30871699 10.1016/j.jacc.2018.12.055 PMC6424590

[emmm202216581-bib-0038] Palikaras K , Tavernarakis N (2014) Mitochondrial homeostasis: the interplay between mitophagy and mitochondrial biogenesis. Exp Gerontol 56: 182–188 24486129 10.1016/j.exger.2014.01.021

[emmm202216581-bib-0039] Puchalska P , Crawford PA (2017) Multi‐dimensional roles of ketone bodies in fuel metabolism, signaling, and therapeutics. Cell Metab 25: 262–284 28178565 10.1016/j.cmet.2016.12.022 PMC5313038

[emmm202216581-bib-0040] Puchalska P , Martin SE , Huang X , Lengfeld JE , Daniel B , Graham MJ , Han X , Nagy L , Patti GJ , Crawford PA (2019) Hepatocyte‐macrophage acetoacetate shuttle protects against tissue fibrosis. Cell Metab 29: 383–398 30449686 10.1016/j.cmet.2018.10.015 PMC6559243

[emmm202216581-bib-0041] Quiros PM , Espanol Y , Acin‐Perez R , Rodriguez F , Barcena C , Watanabe K , Calvo E , Loureiro M , Fernandez‐Garcia MS , Fueyo A *et al* (2014) ATP‐dependent Lon protease controls tumor bioenergetics by reprogramming mitochondrial activity. Cell Rep 8: 542–556 25017063 10.1016/j.celrep.2014.06.018

[emmm202216581-bib-0042] Shi L , Zhao D , Hou C , Peng Y , Liu J , Zhang S , Liu J , Long J (2017) Early interleukin‐6 enhances hepatic ketogenesis in APPSWE/PSEN1dE9 mice via 3‐hydroxy‐3‐methylglutary‐CoA synthase 2 signaling activation by p38/nuclear factor kappaB p65. Neurobiol Aging 56: 115–126 28528772 10.1016/j.neurobiolaging.2017.04.014

[emmm202216581-bib-0043] Smyrnias I , Gray SP , Okonko DO , Sawyer G , Zoccarato A , Catibog N , Lopez B , Gonzalez A , Ravassa S , Diez J *et al* (2019) Cardioprotective effect of the mitochondrial unfolded protein response during chronic pressure overload. J Am Coll Cardiol 73: 1795–1806 30975297 10.1016/j.jacc.2018.12.087 PMC6456800

[emmm202216581-bib-0044] Song J , Herrmann JM , Becker T (2021) Quality control of the mitochondrial proteome. Nat Rev Mol Cell Biol 22: 54–70 33093673 10.1038/s41580-020-00300-2

[emmm202216581-bib-0045] Souza AC , Tsuji T , Baranova IN , Bocharov AV , Wilkins KJ , Street JM , Alvarez‐Prats A , Hu X , Eggerman T , Yuen PS *et al* (2015) TLR4 mutant mice are protected from renal fibrosis and chronic kidney disease progression. Physiol Rep 3: e12558 26416975 10.14814/phy2.12558 PMC4600397

[emmm202216581-bib-0046] Teng H , Wu B , Zhao K , Yang G , Wu L , Wang R (2013) Oxygen‐sensitive mitochondrial accumulation of cystathionine beta‐synthase mediated by Lon protease. Proc Natl Acad Sci USA 110: 12679–12684 23858469 10.1073/pnas.1308487110 PMC3732959

[emmm202216581-bib-0047] Tian Q , Li T , Hou W , Zheng J , Schrum LW , Bonkovsky HL (2011) Lon peptidase 1 (LONP1)‐dependent breakdown of mitochondrial 5‐aminolevulinic acid synthase protein by heme in human liver cells. J Biol Chem 286: 26424–26430 21659532 10.1074/jbc.M110.215772 PMC3143606

[emmm202216581-bib-0048] Tomita I , Kume S , Sugahara S , Osawa N , Yamahara K , Yasuda‐Yamahara M , Takeda N , Chin‐Kanasaki M , Kaneko T , Mayoux E *et al* (2020) SGLT2 inhibition mediates protection from diabetic kidney disease by promoting ketone body‐induced mTORC1 inhibition. Cell Metab 32: 404–419 32726607 10.1016/j.cmet.2020.06.020

[emmm202216581-bib-0049] Venable AH , Lee LE , Feola K , Santoyo J , Broomfield T , Huen SC (2022) Fasting‐induced HMGCS2 expression in the kidney does not contribute to circulating ketones. Am J Physiol Renal Physiol 322: F460–F467 35224990 10.1152/ajprenal.00447.2021 PMC9076412

[emmm202216581-bib-0050] Venkatesh S , Li M , Saito T , Tong M , Rashed E , Mareedu S , Zhai P , Barcena C , Lopez‐Otin C , Yehia G *et al* (2019) Mitochondrial LonP1 protects cardiomyocytes from ischemia/reperfusion injury *in vivo* . J Mol Cell Cardiol 128: 38–50 30625302 10.1016/j.yjmcc.2018.12.017

[emmm202216581-bib-0051] Wan L , Lu J , Fu J , Huang J , Yang Q , Xin B , Chen L , Huo Y , Zhong Y , Guo C (2018) Acetylcholinesterase inhibitor donepezil effects on plasma beta‐hydroxybutyrate levels in the treatment of Alzheimer's disease. Curr Alzheimer Res 15: 917–927 29852870 10.2174/1567205015666180601091818

[emmm202216581-bib-0052] Wan S , Xi M , Zhao HB , Hua W , Liu YL , Zhou YL , Zhuo YJ , Liu ZZ , Cai ZD , Wan YP *et al* (2019) HMGCS2 functions as a tumor suppressor and has a prognostic impact in prostate cancer. Pathol Res Pract 215: 152464 31176575 10.1016/j.prp.2019.152464

[emmm202216581-bib-0053] Wang Q , Zhou Y , Rychahou P , Fan TW , Lane AN , Weiss HL , Evers BM (2017) Ketogenesis contributes to intestinal cell differentiation. Cell Death Differ 24: 458–468 27935584 10.1038/cdd.2016.142 PMC5344206

[emmm202216581-bib-0054] Wang P , Deng J , Dong J , Liu J , Bigio EH , Mesulam M , Wang T , Sun L , Wang L , Lee AY *et al* (2019a) TDP‐43 induces mitochondrial damage and activates the mitochondrial unfolded protein response. PLoS Genet 15: e1007947 31100073 10.1371/journal.pgen.1007947 PMC6524796

[emmm202216581-bib-0055] Wang YH , Liu CL , Chiu WC , Twu YC , Liao YJ (2019b) HMGCS2 mediates ketone production and regulates the proliferation and metastasis of hepatocellular carcinoma. Cancer 11: 1876 10.3390/cancers11121876 PMC6966636 31779269

[emmm202216581-bib-0056] Wang L , Bi X , Han J (2020a) Silencing of peroxisome proliferator‐activated receptor‐alpha alleviates myocardial injury in diabetic cardiomyopathy by downregulating 3‐hydroxy‐3‐methylglutaryl‐coenzyme A synthase 2 expression. IUBMB Life 72: 1997–2009 32734614 10.1002/iub.2337

[emmm202216581-bib-0057] Wang YH , Suk FM , Liao YJ (2020b) Loss of HMGCS2 enhances lipogenesis and attenuates the protective effect of the ketogenic diet in liver cancer. Cancer 12: 1797 10.3390/cancers12071797 PMC7408319 32635582

[emmm202216581-bib-0058] Webster AC , Nagler EV , Morton RL , Masson P (2017) Chronic kidney disease. Lancet 389: 1238–1252 27887750 10.1016/S0140-6736(16)32064-5

[emmm202216581-bib-0059] Yi‐An K (2014) BioStudies E‐MTAB‐2502 (https://www.ebi.ac.uk/biostudies/arrayexpress/studies/E‐MTAB‐2502). [DATASET]

[emmm202216581-bib-0060] Yuan Y , Huang S , Wang W , Wang Y , Zhang P , Zhu C , Ding G , Liu B , Yang T , Zhang A (2012) Activation of peroxisome proliferator‐activated receptor‐gamma coactivator 1alpha ameliorates mitochondrial dysfunction and protects podocytes from aldosterone‐induced injury. Kidney Int 82: 771–789 22648295 10.1038/ki.2012.188

[emmm202216581-bib-0061] Zhang D , Yang H , Kong X , Wang K , Mao X , Yan X , Wang Y , Liu S , Zhang X , Li J *et al* (2011) Proteomics analysis reveals diabetic kidney as a ketogenic organ in type 2 diabetes. Am J Physiol Endocrinol Metab 300: E287–E295 20959534 10.1152/ajpendo.00308.2010

[emmm202216581-bib-0062] Zhang L , Wang F , Wang L , Wang W , Liu B , Liu J , Chen M , He Q , Liao Y , Yu X *et al* (2012) Prevalence of chronic kidney disease in China: a cross‐sectional survey. Lancet 379: 815–822 22386035 10.1016/S0140-6736(12)60033-6

[emmm202216581-bib-0063] Zou K , Hu Y , Li M , Wang H , Zhang Y , Huang L , Xie Y , Li S , Dai X , Xu W *et al* (2019) Potential role of HMGCS2 in tumor angiogenesis in colorectal cancer and its potential use as a diagnostic marker. Can J Gastroenterol Hepatol 2019: 8348967 31355161 10.1155/2019/8348967 PMC6634068

