# Supplementary material for: LONP1 targets HMGCS2 to protect mitochondrial function and attenuate chronic kidney disease
Source: EMBO Mol Med. 2023 Jan 11;15(2):e16581. doi: 10.15252/emmm.202216581 (PMC9906428; doi:10.15252/emmm.202216581)
Supplement: Supplementary file 1 — Appendix [file EMMM-15-e16581-s001.pdf]

LONP1 Targets HMGCS2 to Protect Mitochondrial Function and Attenuate Chronic Kidney Disease

Mi Bai [1,2,3*], Mengqiu Wu[1,2,3*], Mingzhu Jiang [1,2*], Jia He [1,2], Xu Deng [1,2], Shuang Xu [1,2], Jiaojiao Fan [1,2], Mengqiu Miao [1,2], Ting Wang [1,2], Yuting Li [1,2], Xiaowen Yu[1,2,3], Lin Wang[5], Yue Zhang [1,2], Songming Huang [1,2], Li Yang[4], Zhanjun Jia [1,2, 3], and Aihua Zhang [1,2, 3]

Table of Contents

Appendix Figure S1-3 with their legends

Appendix Table S1

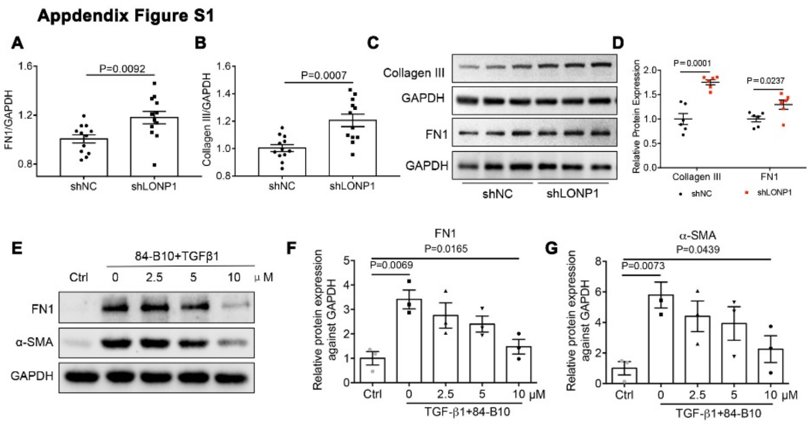

Appendix Figure S1. The effect of tubular LONP1 and 84-B10 on the activation of NRK-49F cells.

A, B qRT-PCR analysis of FN1 and Collagen III transcript levels in NRK-49F cells. After interfering LONP1 with shRNA in mPTC for 24 h, the supernatant was collected to stimulate NRK-49F cells for 24 h. Two independent experiments were carried out (n=12 in each group, biological replicates).

C, D Representative immunoblot and densitometric analysis of FN1 and Collagen III in NRK-49F cells treated as indicated. Two independent experiments were carried out (n=6 in each group, biological replicates).

E-G Representative immunoblot and densitometric analysis of FN1 and α-SMA in NRK-49F cells treated as indicated. Three independent experiments were carried out and quantification of the abundance of these proteins is shown in panel (n=3 in each group, biological replicates).

Data information: Data are presented as mean ± SEM. Student's t-test.

Appdendix Figure S2

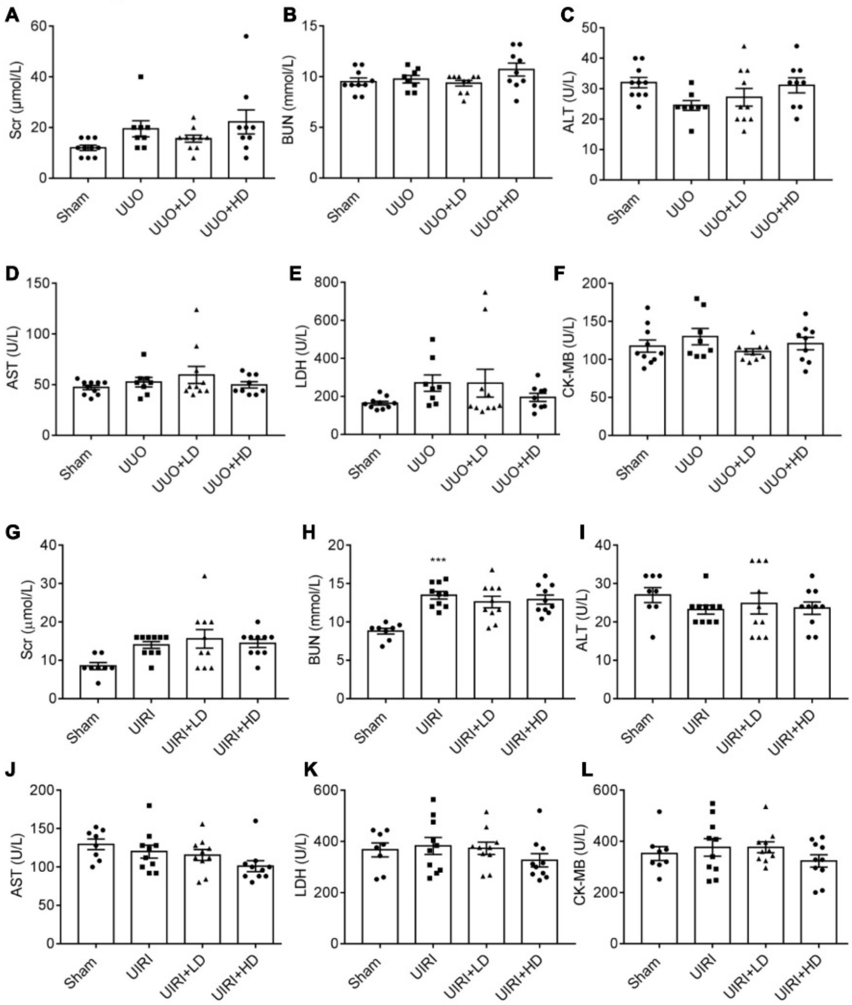

Appendix Figure S2. The therapeutic doses of 84-B10 had no obvious renal, hepatic, heart and systemic toxicity.

A-F The mice were pre-treated with low dose (0.5 mg/kg) or high dose (7.5 mg/kg) of 84-B10 before UUO surgery and then treated daily for 7 consecutive days and sacrificed. Serum concentrations of Scr (A), BUN (B), ALT (C), AST (D), LDH (E) and CK-MB (F) were detected (n=9-10 in each group, biological replicates).

G-L The mice were treated with low dose (2.5 mg/kg) or high dose (5 mg/kg) of 84-B10 3 days after UIRI surgery and then treated daily for 19 consecutive days and sacrificed. Serum concentrations of Scr (G), BUN (H), ALT (I), AST (J), LDH (K) and CK-MB (L) were detected (n=8 in Sham group, n=10 in the other three groups, biological replicates).

Data information: Data are presented as mean ± SEM.

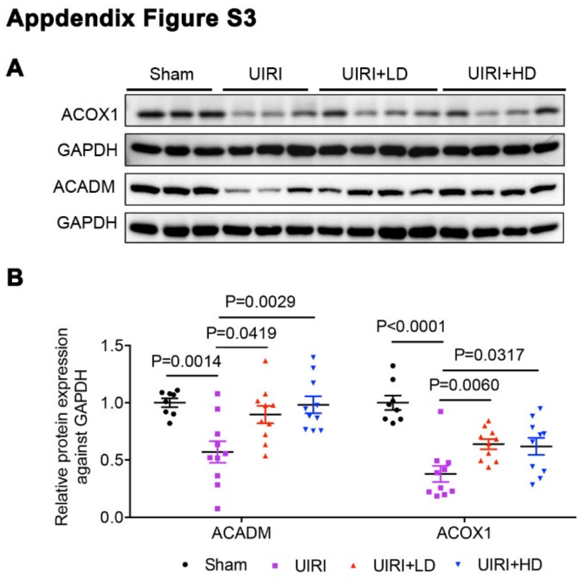

Appendix Figure S3. The effect of 84-B10 on fatty acid β-oxidation (FAO) in UIRI model.

A Representative Western blot images of ACADM and ACOX1 in UIRI model following 84-B10 treatment.

B Densitometric analysis of ACADM and ACOX1 protein levels in UIRI model following 84-B10 treatment (n=8 in Sham group, n=10 in the other three groups, biological replicates).

Data information: Data are presented as mean ± SEM. Student's t-test.

Appendix Table S1. The sequences of the mitochondrial gene's primers.

Primer Name	Primer Sequence 5 ' -3 '
mtNd1-F	ACACTTATTACAACCCAAGAACACAT
mtNd1-R	TCATATTATGGCTATGGGTCAGG
mtNd2-F	CCATCAACTCAATCTCACTTCTATG
mtNd2-R	GAATCCTGTTAGTGGTGGAAGG
mtNd4-F	GCTTACGCCAAACAGAT
mtNd4-R	TAGGCAGAATAGGAGTGAT
mtNd4l-F	GCCATCTACCTTCTTCA
mtNd4l-R	TAGGGCTAGTCCTACAGC
mtNd5-F	GCCAACAACATATTTCAACTTTTC
mtNd5-R	ACCATCATCCAATTAGTAGAAAGGA
mtNd6-F	GGGAGATTGGTTGATGTA
mtNd6-R	ATACCCGCAAACAAAGAT
mtCo1-F	CAGACCGCAACCTAAACACA
mtCo1-R	TTCTGGGTGCCCAAAGAAT
mtCo2-F	GCCGACTAAATCAAGCAACA
mtCo2-R	CAATGGGCATAAAGCTATGG
mtCo3-F	CGTGAAGGAACCTACCAAGG
mtCo3-R	ATTCCTGTTGGAGGTCAGCA
mtAtp6-F	CCATAAATCTAAGTATAGCCATTCCAC
mtAtp6-R	AGCTTTTTAGTTTGTGTCGGAAG
mtAtp8-F	ACATTCCCACTGGCACC
mtAtp8-R	GGGGTAATGAATGAGGC
