# Supplementary material for: LONP1 targets HMGCS2 to protect mitochondrial function and attenuate chronic kidney disease
Source: EMBO Mol Med. 2023 Jan 11;15(2):e16581. doi: 10.15252/emmm.202216581 (PMC9906428; doi:10.15252/emmm.202216581)
Supplement: Supplementary file 2 — Expanded View Figures PDF [file EMMM-15-e16581-s011.pdf]

Expanded View Figures

Figure EV1. LONP1 was down-regulated in CKD kidneys based on the analysis of online datasets and proximal tubular-specific overexpression of *Lonp1* alleviated the reduction of mitochondrial genes expression and protected mitochondrial morphology in UUO model.

A *Lonp1* expression from online human RNA sequencing data (Data ref: Yi-An, 2014). $N = 20$ in Ctl group an $n = 19$ in CKD group (biological replicates).
B *Lonp1* expression from online mouse UUO model RNA sequencing data (Data ref: Denby *et al*, 2020). $N = 4$ in each group (biological replicates).
C The mRNA expression of *Lonp1* in our UUO models ($n = 5$–7, biological replicates).
D *Lonp1* expression from kidney single cell datasets Gene Atlas of Reversible Unilateral Ureteric Obstruction Model (http://www.ruuo-kidney-gene-atlas.com/).
E, F Pearson correlation analysis of LONP1 and BUN and Serum Creatinine in CKD patients ($n = 30$).
G Western blot and densitometric analysis for the expression of LONP1 of proximal tubular cells isolated from WT and cKI mice ($n = 3$, biological replicates).
H, I qRT-PCR analysis of mitochondrial genes in WT and cKI mice after UUO ($n = 8$ in each group, biological replicates).
J Transmission electron microscopy images of intact renal tubule cells and mitochondria in WT and cKI mice after UUO. Scale bar: 5 μm, 2 μm and 500 nm.

Data information: Data are presented as mean ± SEM. Student's *t*-test. In the boxplot of B, the central band represents the median line; the boxes represent range between 25 and 75%; the whiskers represent range within the 1.5 IQR (Inter-Quartile Range).
Source data are available online for this figure.

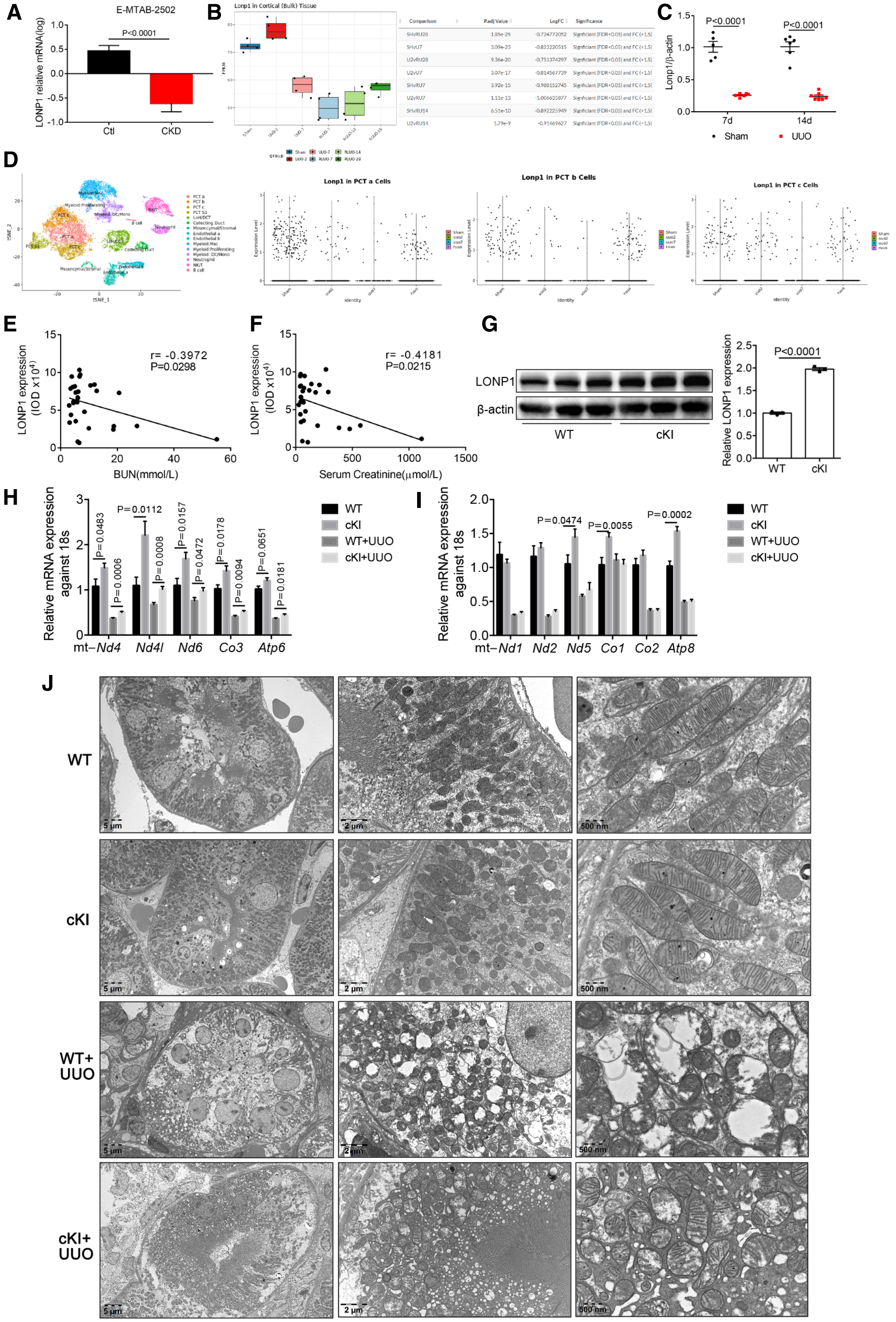

Figure EV1.

 © 2023 The Authors

Figure EV2. Validation of proximal tubular-specific deletion of *Lonp1* (cKO), physiological indexes of aged cKO mice, and mitochondrial morphology in UUO model.

A LONP1 immunofluorescence staining of primary renal tubular epithelial cells (PTECs) isolated from WT and cKO mice. Scale bar: 20 μm.
B Western blot analysis for the expression of LONP1 in medulla, glomerular and PTECs.
C–G Body weight (n = 8 or 10), blood pressure (BP, n = 5 or 6), serum creatinine (Scr, n = 8 or 10), blood urea nitrogen (BUN, n = 8 or 10) and Masson's trichrome staining in elderly WT and cKO mice (18 months). Scale bar: 50 μm.
H Transmission electron microscopy images of the mitochondria in tubular cells in WT and cKO mice after UUO model. Scale bar: 1 μm.

Data information: Data are presented as mean ± SEM. Student's t-test.
Source data are available online for this figure.

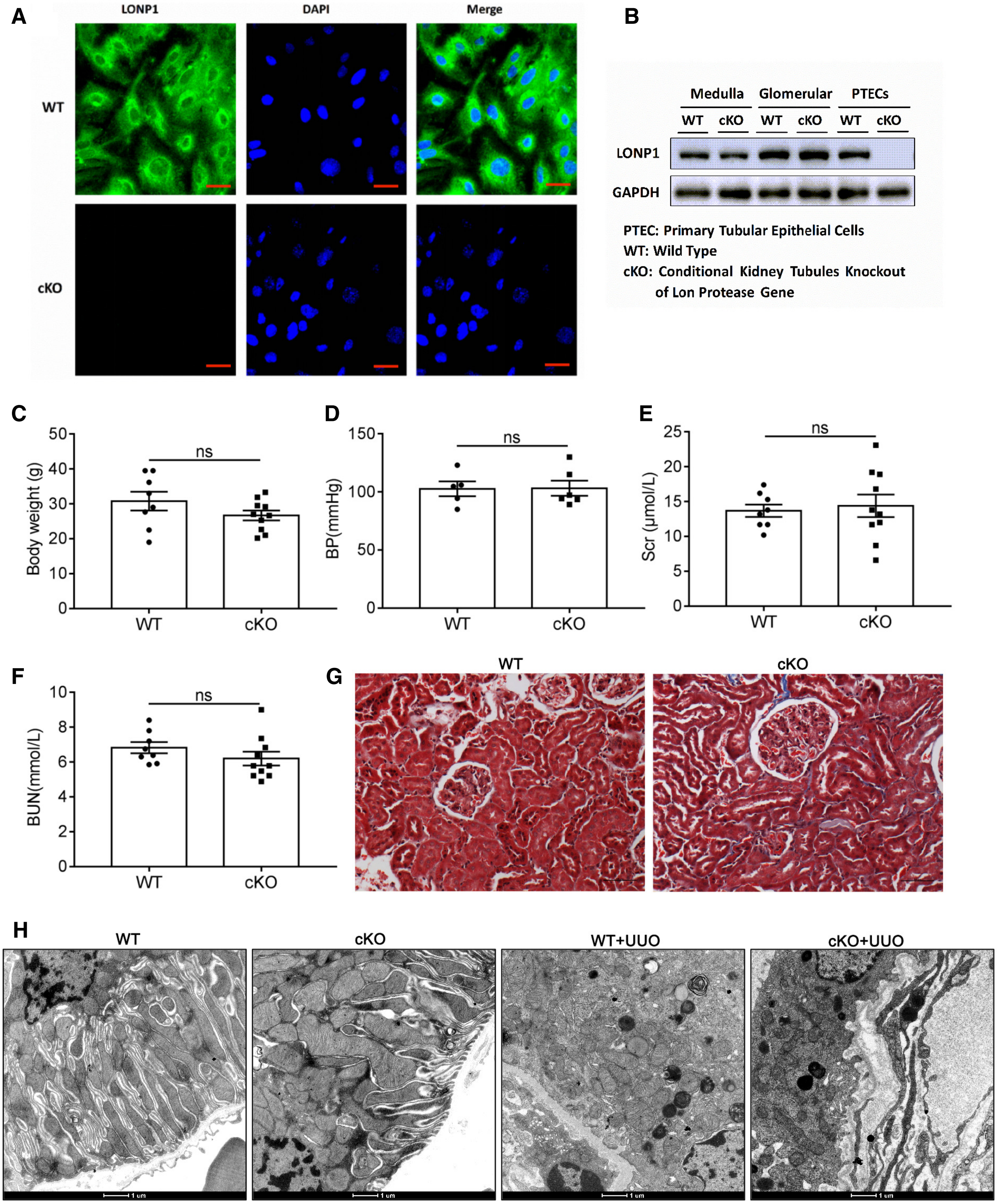

Figure EV2.

 © 2023 The Authors

Figure EV3. Proximal tubular-specific overexpression or deletion of *Lonp1* mitigated or aggravated renal fibrosis and mitochondrial morphology damage in 5/6Nx model and the protective effect of LONP1 in primary proximal tubular cells.

- A, B Sirius red staining and fibrotic area statistics of WT and cKI mice in 5/6Nx model. Scale bar: 50 μm ($n = 5$ in WT groups, $n = 6$ in cKI groups, biological replicates).
- C, D Sirius red staining and fibrotic area statistics of WT and cKO mice in 5/6Nx model. Scale bar: 50 μM ($n = 3$–6, biological replicates).
- E Transmission electron microscopy images of the mitochondria in tubular cells in WT and cKO mice after 5/6Nx model. Scale bar: 1 μm.
- F Western blot and densitometric analysis for the expression of LONP1 after transfected with vector or *Lonp1* plasmid.
- G, H Western blot and densitometric analysis for the expression of LONP1 after transfected with vector or Lonp1 shRNA ($n = 3$, biological replicates).
- I, J The proximal tubular cells were isolated from WT and Lonp1 cKI mice. The primary cells were treated with TGF-β1 for 24 h. Western blot and densitometric analysis for the expression of LONP1, FN1 and Collagen III ($n = 3$ in each group).
- K Quantification of mitochondrial ROS production ($n = 3$, biological replicates). The primary cells were treated with TGF-β1 for 4 h.

Data information: In (H), data are presented as mean ± SEM. Student's *t*-test. In (B, D, J, K), data are presented as mean ± SEM. One-way ANOVA.
Source data are available online for this figure.

© 2023 The Authors

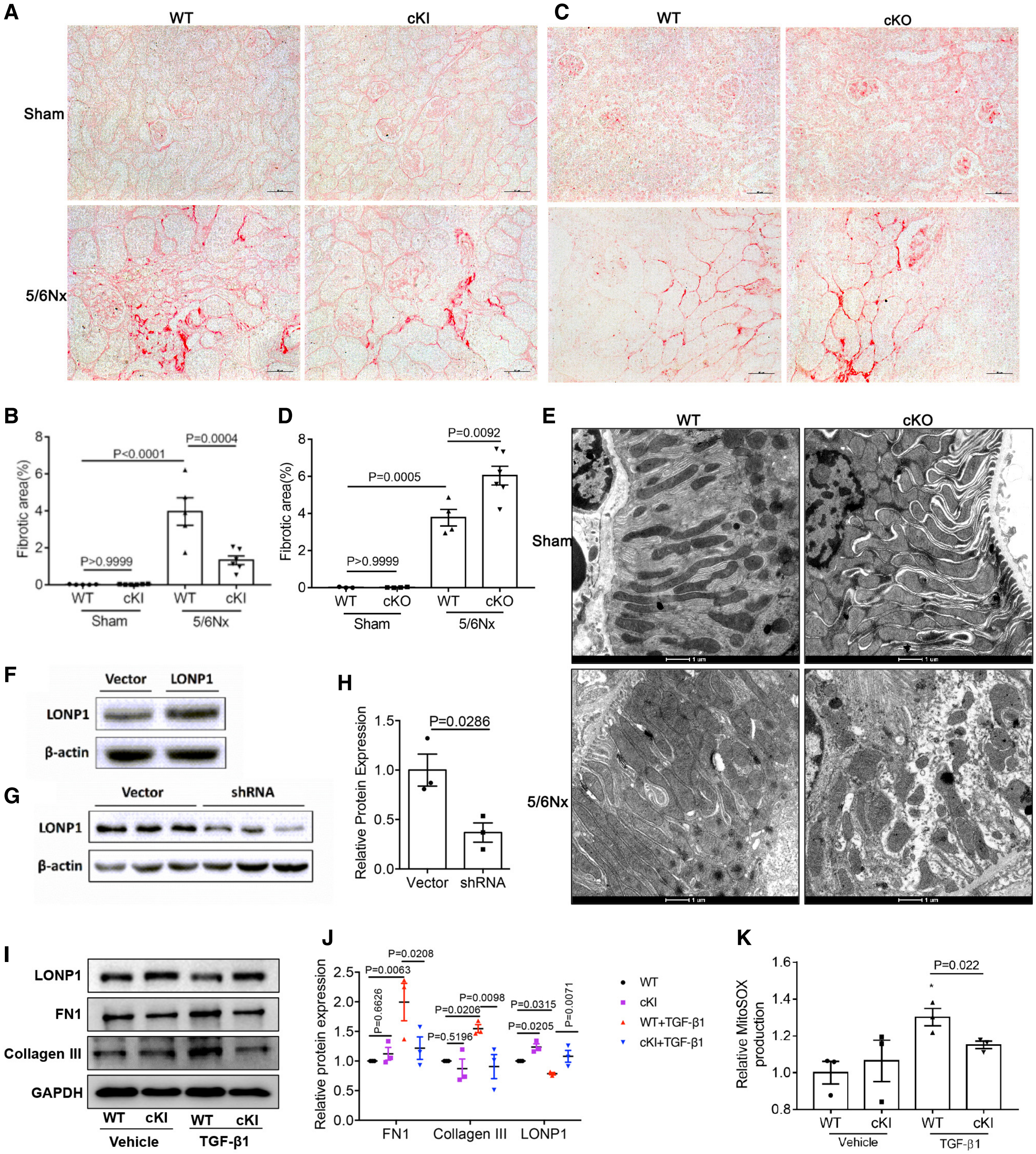

Figure EV3.

© 2023 The Authors

Figure EV4. HMGCS2 aggravated TGF-β1-induced mitochondrial dysfunction and fibrotic response in mPTCs and primary proximal tubular cells.

- A The expression of known substrates of LONP1 and HMGCS2 in our proteomics ($n = 3$, biological replicates). Compared with Hmgcs2, there was little difference in their expression in WT and Lonp1 cKO groups.
- B Western blot showing the intramitochondrial localization of HMGCS2 in mPTC cells after transfected with Vector or *Hmgcs2* plasmids ($n = 2$, biological replicates).
- C Quantification of mitochondrial ROS production in mPTC cells after transfected with Vector or *Hmgcs2* plasmids following TGF-β1 treatment ($n = 4$ in each group, biological replicates).
- D Quantitation of mitochondrial membrane potential ($\Delta\Psi m$) by JC-1 staining in mPTC cells after transfected with Vector or *Hmgcs2* plasmids following TGF-β1 treatment ($n = 4$ in each group, biological replicates).
- E qRT-PCR analysis of FN1, Collagen III and α-SMA in mPTC cells after transfected with Vector or *Hmgcs2* plasmids following TGF-β1 treatment ($n = 4$ or 5, biological replicates).
- F, G Western blot and densitometric analysis for the expression of FN1 and Collagen III in mPTC cells after transfected with Vector or *Hmgcs2* plasmids following TGF-β1 treatment ($n = 3$ in each group, biological replicates).
- H qRT-PCR analysis of FN1 and Collagen I in mPTC cells after transfected with shNC or sh*Hmgcs2* plasmids following TGF-β1 treatment ($n = 6$ in each group, biological replicates).
- I, J Western blot and densitometric analysis for the expression of FN1 and Collagen III in mPTC cells after transfected with shNC or sh*Hmgcs2* plasmids following TGF-β1 treatment ($n = 3$ in each group, biological replicates).
- K The proximal tubular cells were isolated from C57BL/6J mice and infected with HMGCS2-lentivirus (pLVX-Puro-mHmgcs2-HA) or control lentivirus (vector). Western blot and densitometric analysis for the expression of HA ($n = 3$ in each group, biological replicates).
- L, M The infected primary cells were treated with TGF-β1 for 24 h after infected with HMGCS2-lentivirus or control lentivirus. Western blot and densitometric analysis for the expression of FN1 and Collagen III. Three independent experiments were carried out and quantification of the abundance of these proteins is shown in panel ($n = 3$ in each group).
- N The infected primary cells were treated with TGF-β1 for 4 h. Quantification of mitochondrial ROS production ($n = 4$ in each group, biological replicates).

Data information: In (K), data are presented as mean ± SEM. Student's *t*-test. In (C–E, G, H, J, M, N), data are presented as mean ± SEM. One-way ANOVA.
Source data are available online for this figure.

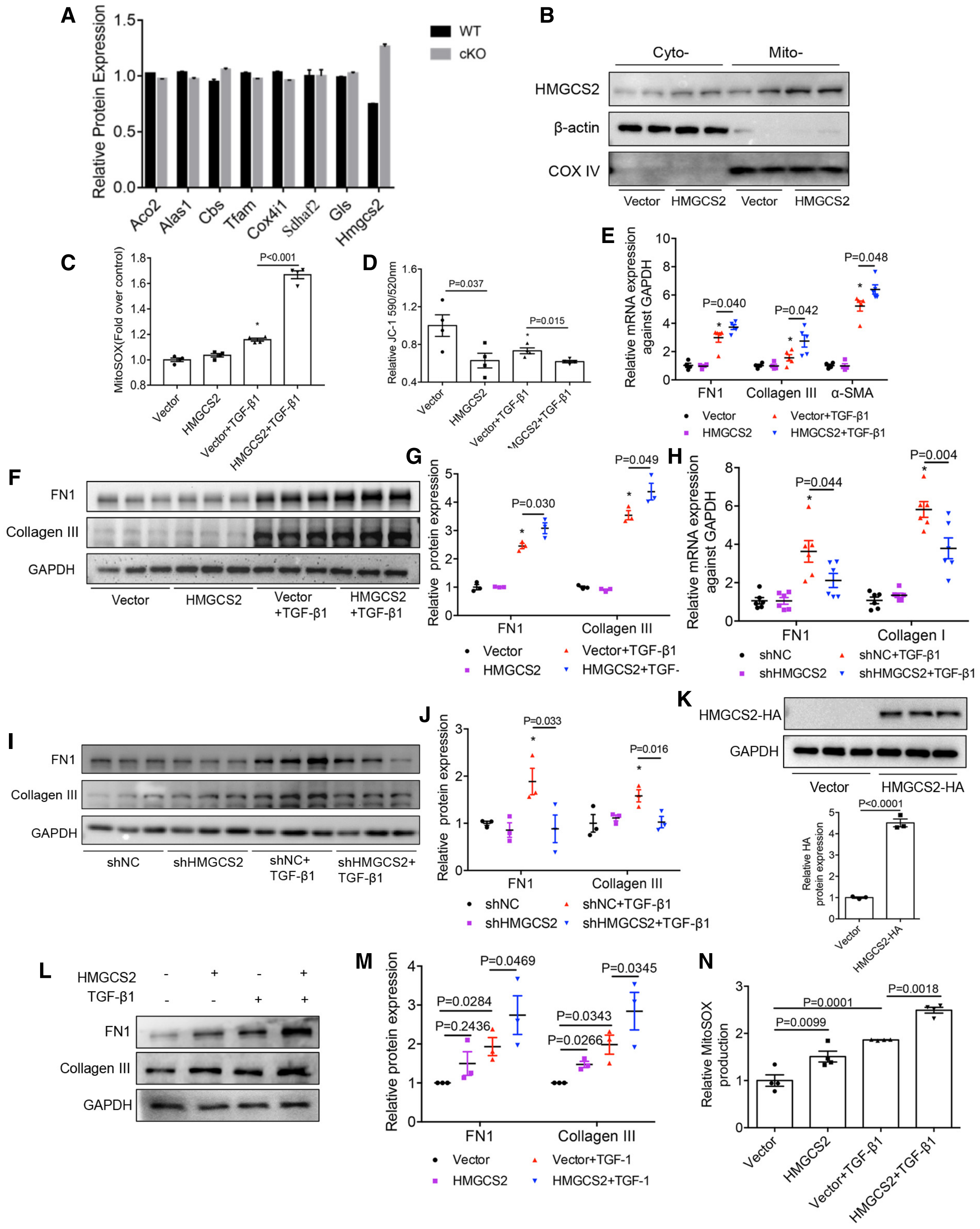

Figure EV4.

 © 2023 The Authors

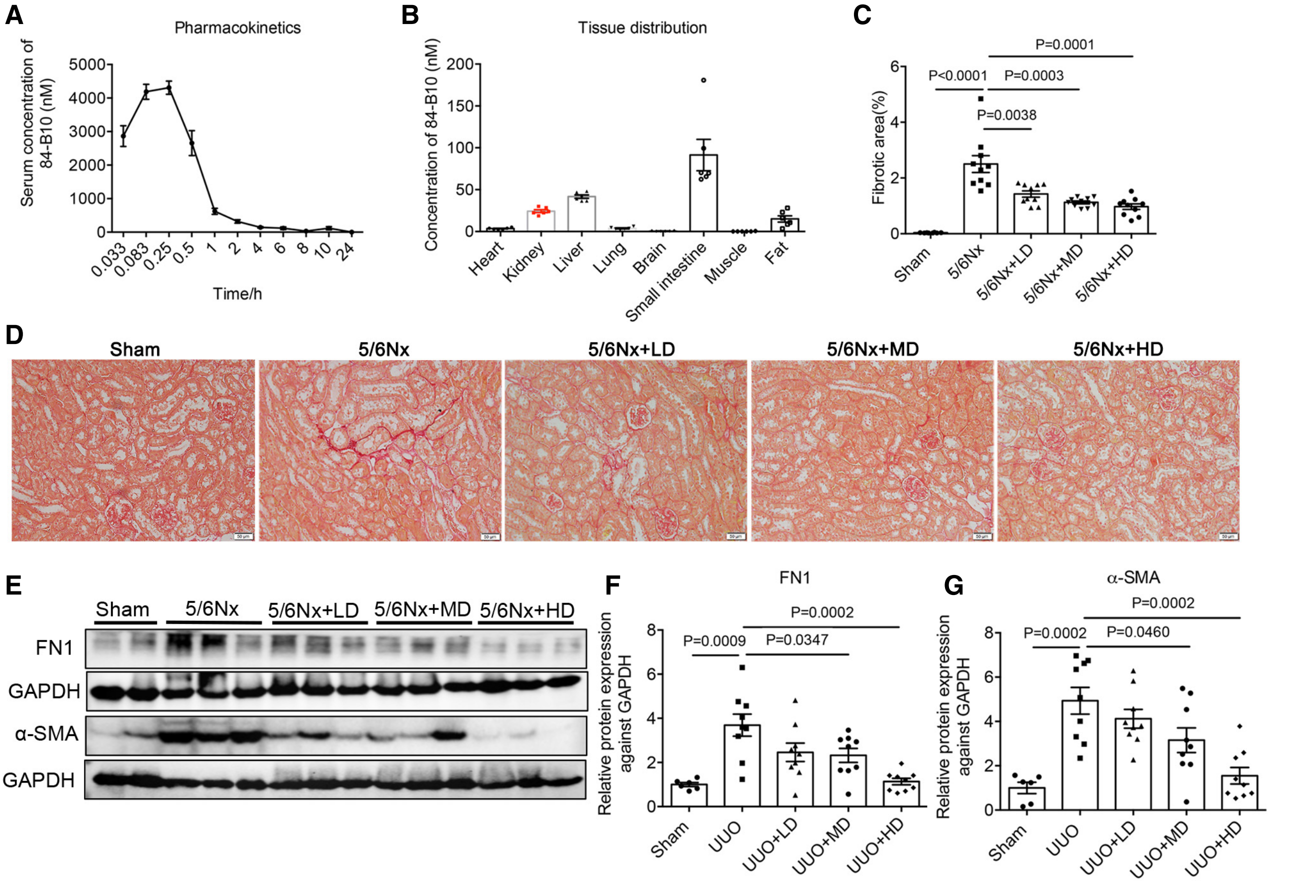

Figure EV5. Pharmacokinetics characteristic of 84-B10 and its anti-fibrosis effect in 5/6Nx mice.

A Plasma-concentration versus time profiles of 84-B10 in mice ($n = 6$, biological replicates).

B Tissue distribution of 84-B10 in mice 30 min after injection ($n = 6$, biological replicates).

C, D Sirius red staining and fibrotic area statistics of 5/6Nx mice treated with LONP1 activator 84-B10. Scale bar: 50 μm ($n = 8$ in WT group, $n = 10$ in other three groups, biological replicates).

E–G Western blot and densitometric analysis for the expression of FN1 and α-SMA in 5/6Nx mice treated with 84-B10 ($n = 6$ in WT group, $n = 9$ in other three groups, biological replicates).

Data information: Data are presented as mean ± SEM. Student's *t*-test.
Source data are available online for this figure.
