# Supplementary material for: LONP1 targets HMGCS2 to protect mitochondrial function and attenuate chronic kidney disease
Source: EMBO Mol Med. 2023 Jan 11;15(2):e16581. doi: 10.15252/emmm.202216581 (PMC9906428; doi:10.15252/emmm.202216581)
Supplement: Supplementary file 3 — Table EV1 [file EMMM-15-e16581-s009.docx]

**Table EV1. The basic information and diagnosis of renal biopsy specimens**

| **Sex** | **Age (yr)** | **Proteinuria**  **(g/24 h)** | **Pathological diagnosis** | **Serum Creatinine(μmol/L)** | **BUN**  **（mmol/L）** |
| --- | --- | --- | --- | --- | --- |
| Girl | 11.7 | 3.65 | endocapillary proliferative glomerulonephritis | 119 | 12.73 |
| Boy | 10.3 | 0.832 | HSPN IIa | 26 | 3.02 |
| Boy | 8.3 | 6.53 | IgAN (Lee III) | 138 | 11.95 |
| Girl | 7.0 | 4.26 | thrombotic microangiopathy | 287 | 5.33 |
| Boy | 13.2 | 2.69 | HSPN IIIa | 43 | 6.92 |
| Girl | 12.0 | 2.73 | IgAN (Lee II) | 39 | 3.92 |
| Boy | 13.4 | 1.17 | IgAN (Lee IV) | 39 | 4.33 |
| Boy | 8.5 | 1.72 | HSPN IIIa | 266 | 6.65 |
| Boy | 7.1 | 1.02 | IgAN (Lee III) | 52 | 4.50 |
| Boy | 4.9 | 2.50 | IgAN (Lee III) | 61 | 6.21 |
| Boy | 13.0 | 0.00 | IgAN (Lee II) | 45 | 5.00 |
| Boy | 9.2 | 4.57 | IgAN (Lee IV) | 85 | 8.40 |
| Boy | 4.9 | 1.71 | IgAN | 193 | 18.77 |
| Boy | 12.8 | 3.10 | IgAN (Lee IV) | 46 | 6.07 |
| Boy | 9.8 | 2.31 | IgAN (Lee III) | 78 | 6.68 |
| Boy | 9.4 | 1.64 | IgAN (Lee V) | 45 | 6.01 |
| Girl | 5.6 | 1.19 | FSGS | 40 | 3.65 |
| Girl | 12.6 | 1.56 | C3 nephritis | 72 | 3.50 |
| Girl | 10.0 | 3.85 | LN IV-G | 209 | 10.55 |
| Boy | 5.6 | 4.59 | IgAN (Lee V) | 106 | 6.55 |
| Boy | 6.7 | 3.56 | Sclerosing glomerulonephritis | 382 | 9.95 |
| Boy | 3.8 | 2.07 | Sclerosing glomerulonephritis | 58 | 6.03 |
| Girl | 7.8 | 0.31 | Diffuse Interstitial Nephritis | 1111 | 55.10 |
| Boy | 13.6 | 0.56 | IgAN (Lee IV) | 175 | 20.62 |
| Boy | 10.2 | 4.38 | IgAN (Lee V) | 46 | 3.18 |
| Girl | 6.6 | 5.75 | IgAN (Lee IV) | 69.7 | 5.30 |
| Girl | 6.7 | 6.23 | IgAN (Lee V) | 476 | 18.62 |
| Boy | 11.9 | 1.43 | IgAN (Lee IV) | 68 | 4.50 |
| Boy | 3.6 | 2.09 | Sclerosing glomerulonephritis | 570 | 26.95 |
| Girl | 2.6 | 1.12 | FSGS | 44 | 5.85 |

HSPN: Henoch-Schonlein Purpura nephropathy; IgAN: IgA nephropathy; FSGS: Focal Segmental Glomerulosclerosis; LN: Lupus Nephritis.
