# Supplementary material for: LONP1 targets HMGCS2 to protect mitochondrial function and attenuate chronic kidney disease
Source: EMBO Mol Med. 2023 Jan 11;15(2):e16581. doi: 10.15252/emmm.202216581 (PMC9906428; doi:10.15252/emmm.202216581)
Supplement: Supplementary file 4 — Table EV2 [file EMMM-15-e16581-s008.docx]

**Table EV2.** **The sequences of the primers used in the study.**

| Primer Name | Primer Sequence 5＇-3＇ |
| --- | --- |
| hFN1-F | CGGTGGCTGTCAGTCAAAG |
| hFN1-R | AAACCTCGGCTTCCTCCATAA |
| hCollagenI-F | GAGGGCCAAGACGAAGACATC |
| hCollagenI-R | CAGATCACGTCATCGCACAAC |
| hα -SMA-F | CTATGAGGGCTATGCCTTGCC |
| hα-SMA-R | GCTCAGCAGTAGTAACGAAGGA |
| hCollagenIV-F | GGGATGCTGTTGAAAGGTGAA |
| hCollagenIV-R | GGTGGTCCGGTAAATCCTGG |
| hCollagenIII-F | GCCAAATATGTGTCTGTGACTCA |
| hCollagenIII-R | GGGCGAGTAGGAGCAGTTG |
| hVimentin-F | AGTCCACTGAGTACCGGAGAC |
| hVimentin-R | CATTTCACGCATCTGGCGTTC |
| hGAPDH-F | AAGTGGTCGTTGAGGGCAATG |
| hGAPDH-R | CTGGGCTACACTGAGCACC |
| mFN1-F | ATGTGGACCCCTCCTGATAGT |
| mFN1-R | GCCCAGTGATTTCAGCAAAGG |
| mα-SMA-F | CCCAGACATCAGGGAGTAATGG |
| mα-SMA-R | TCTATCGGATACTTCAGCGTCA |
| mCollagenI-F | TAAGGGTCCCCAATGGTGAGA |
| mCollagenI-R | GGGTCCCTCGACTCCTACAT |
| mCollagenIII-F | CAGGACCTAAGGGCGAAGATG |
| mCollagenIII-R | TCCGGGCATACCCCGTATC |
| mVimentin-F | CGGCTGCGAGAGAAATTGC |
| mVimentin-F | CCACTTTCCGTTCAAGGTCAAG |
| mPeriostin-F | CACGGCATGGTTATTCCTTCA |
| mPeriostin-R | TCAGGACACGGTCAATGACAT |
| mLonp1-F | TCCGACTTGCACAGCCCTA |
| mLonp1-R | GCGAATGTTCCCGTATGGTAG |
| mGAPDH-F | AGGTCGGTGTGAACGGATTTG |
| mGAPDH-R | TGTAGACCATGTAGTTGAGGTCA |
| mtDNA-F | TTTTATCTGCATCTGAGTTTAATCCTGT |
| mtDNA-R | CCACTTCATCTTACCATTTATTATCGC |
| 18S rRNA-F | TTCGGAACTGAGGCCATGATT |
| 18S rRNA-R | TTTCGCTCTGGTCCGTCTTG |
