# Supplementary figures and images for: LONP1 targets HMGCS2 to protect mitochondrial function and attenuate chronic kidney disease
Source: EMBO Mol Med. 2023 Jan 11;15(2):e16581. doi: 10.15252/emmm.202216581 (PMC9906428; doi:10.15252/emmm.202216581)

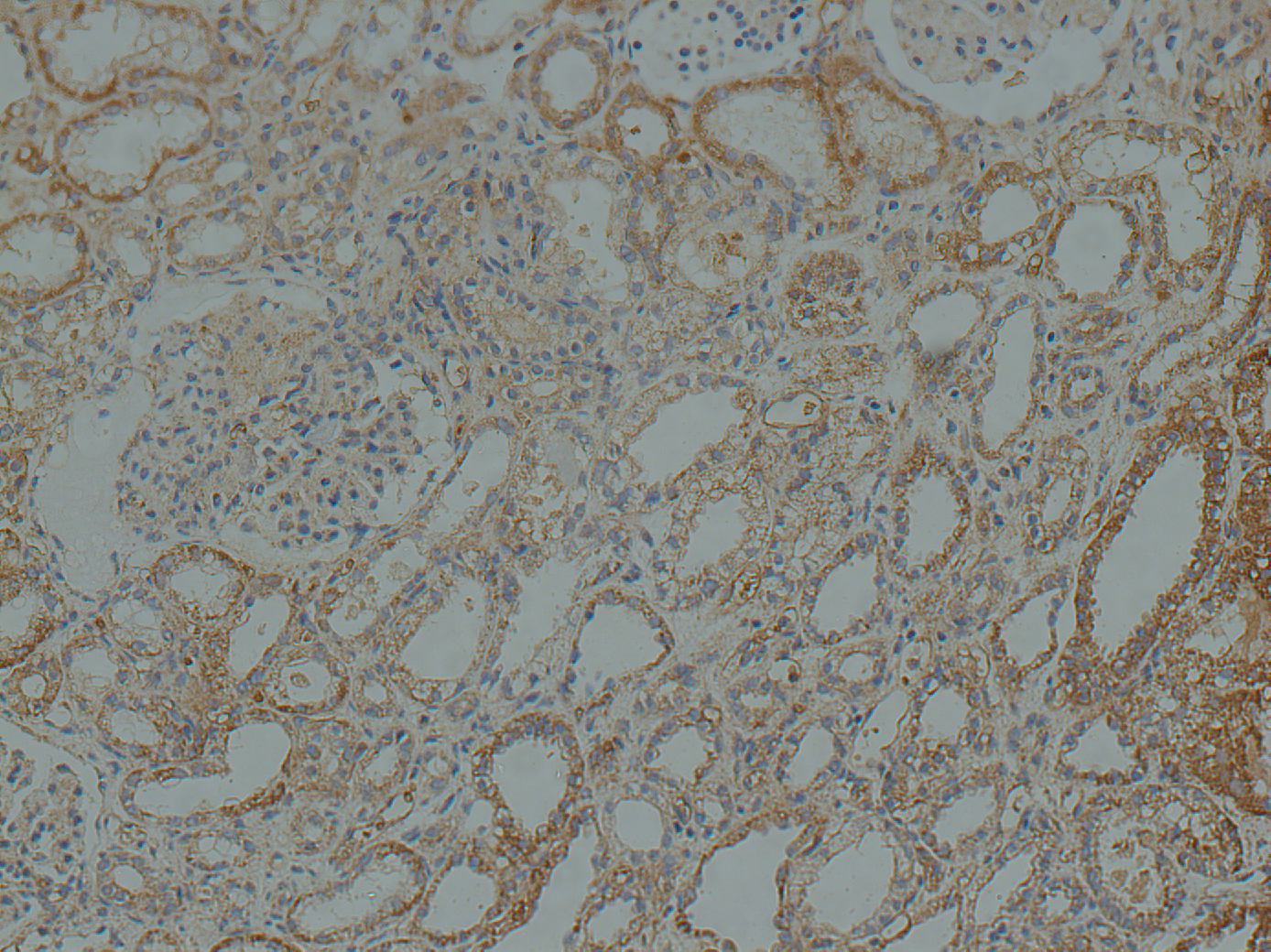

Supplement: Supplementary file 7 — Source Data for Figure 1 [file EMMM-15-e16581-s004.zip › Figure 1/1A-C/Mild.tif]

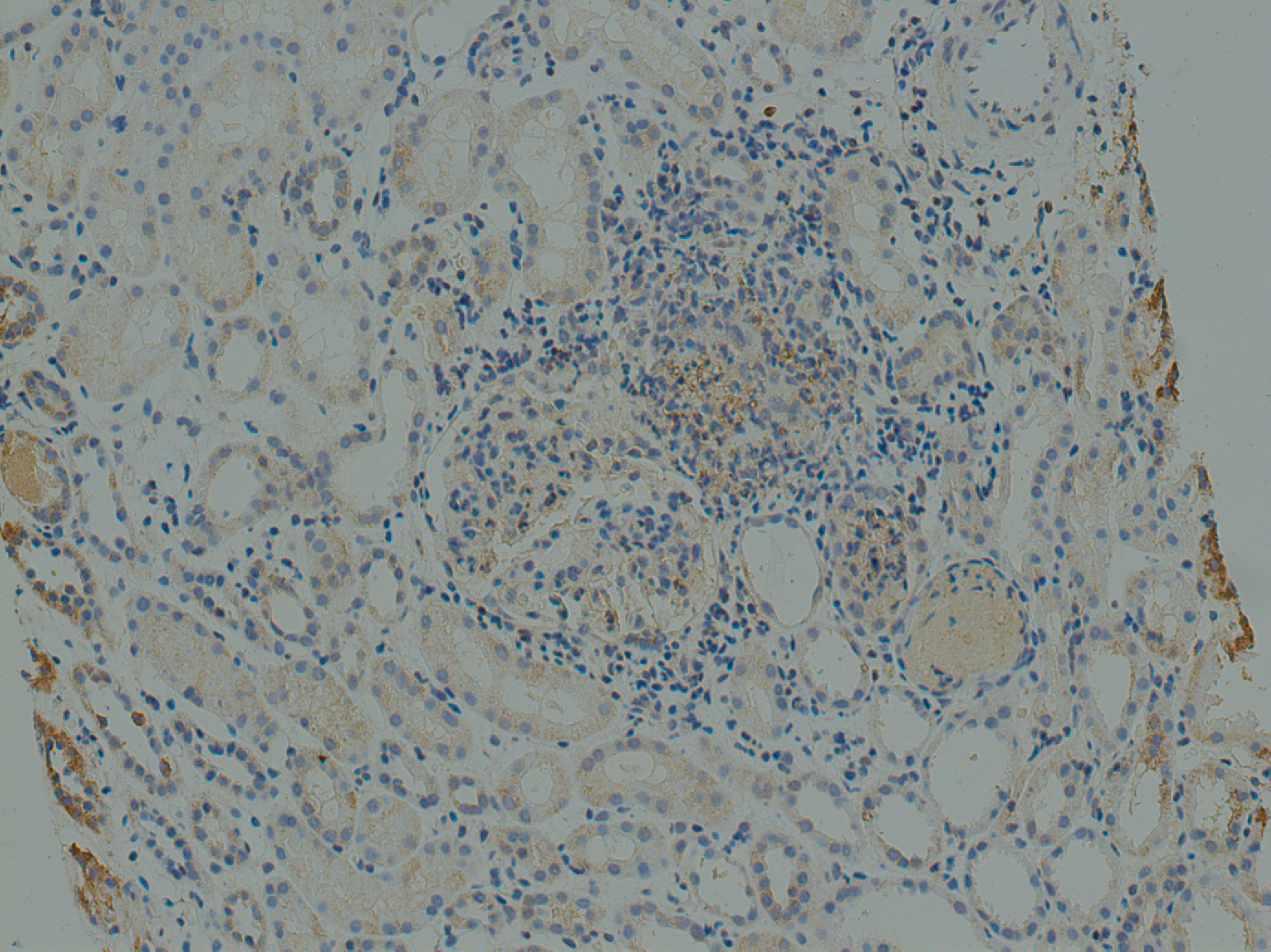

Supplement: Supplementary file 7 — Source Data for Figure 1 [file EMMM-15-e16581-s004.zip › Figure 1/1A-C/Moderate.tif]

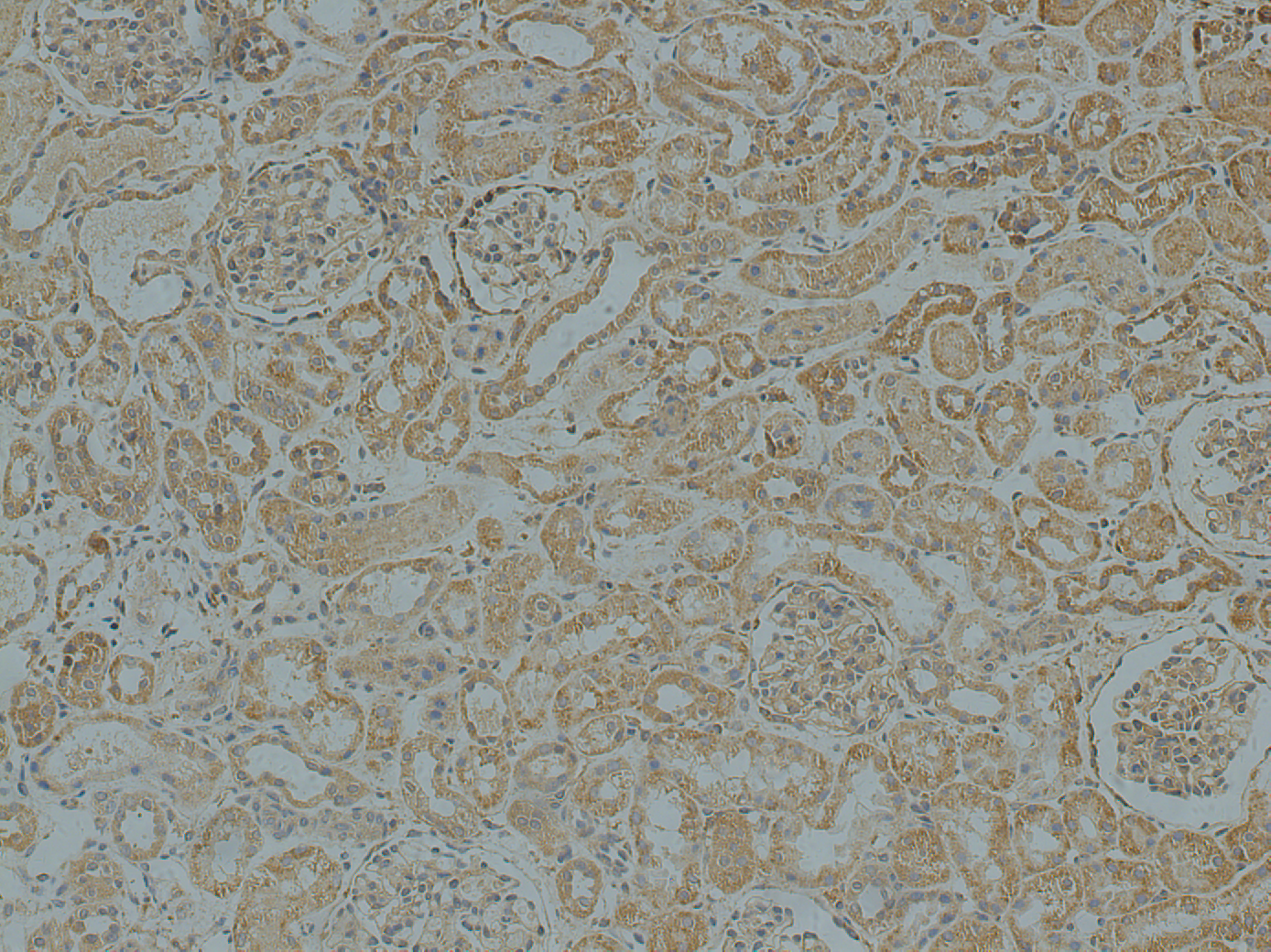

Supplement: Supplementary file 7 — Source Data for Figure 1 [file EMMM-15-e16581-s004.zip › Figure 1/1A-C/Normal.tif]

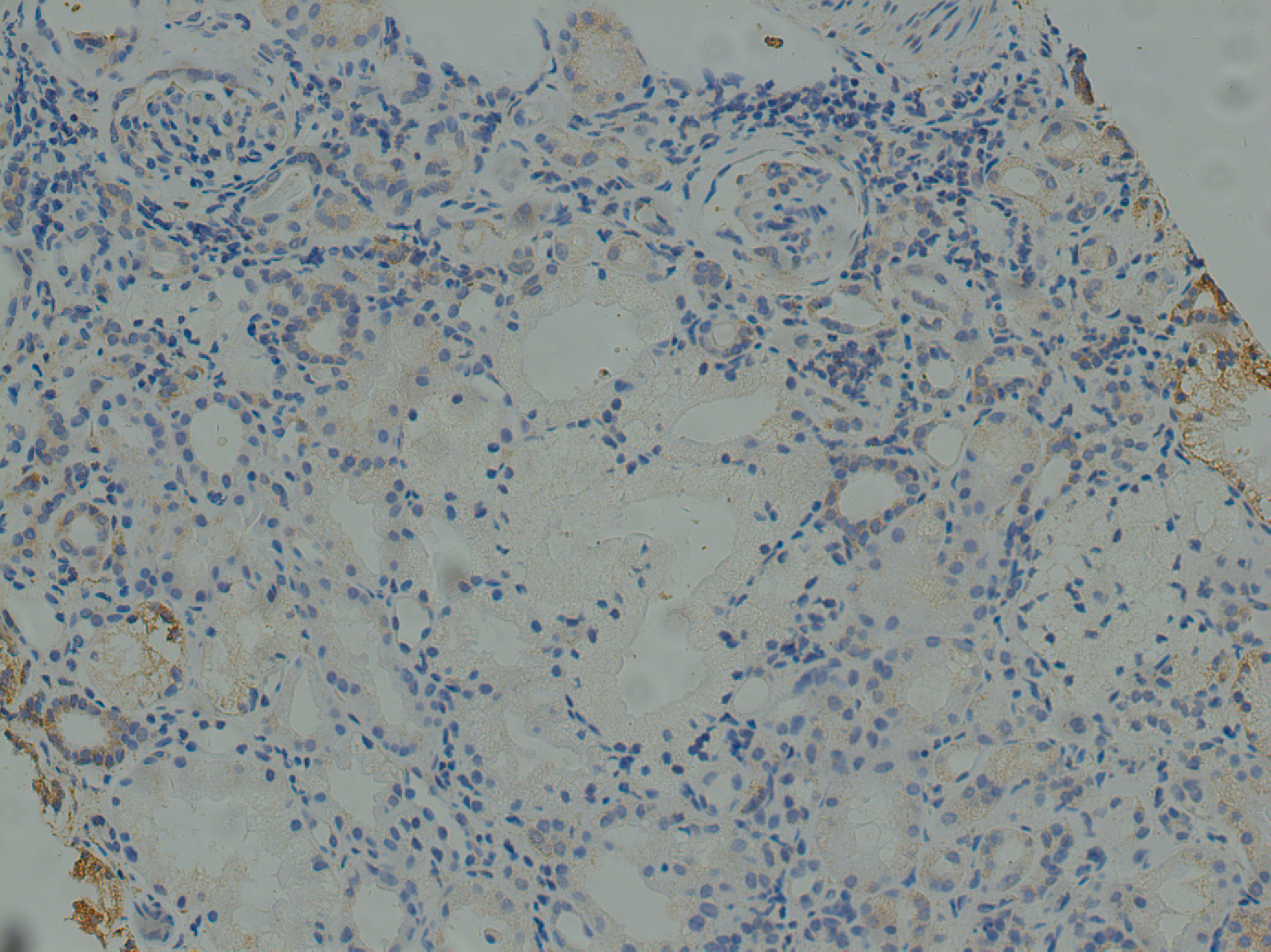

Supplement: Supplementary file 7 — Source Data for Figure 1 [file EMMM-15-e16581-s004.zip › Figure 1/1A-C/Severe.tif]

Fig 1D

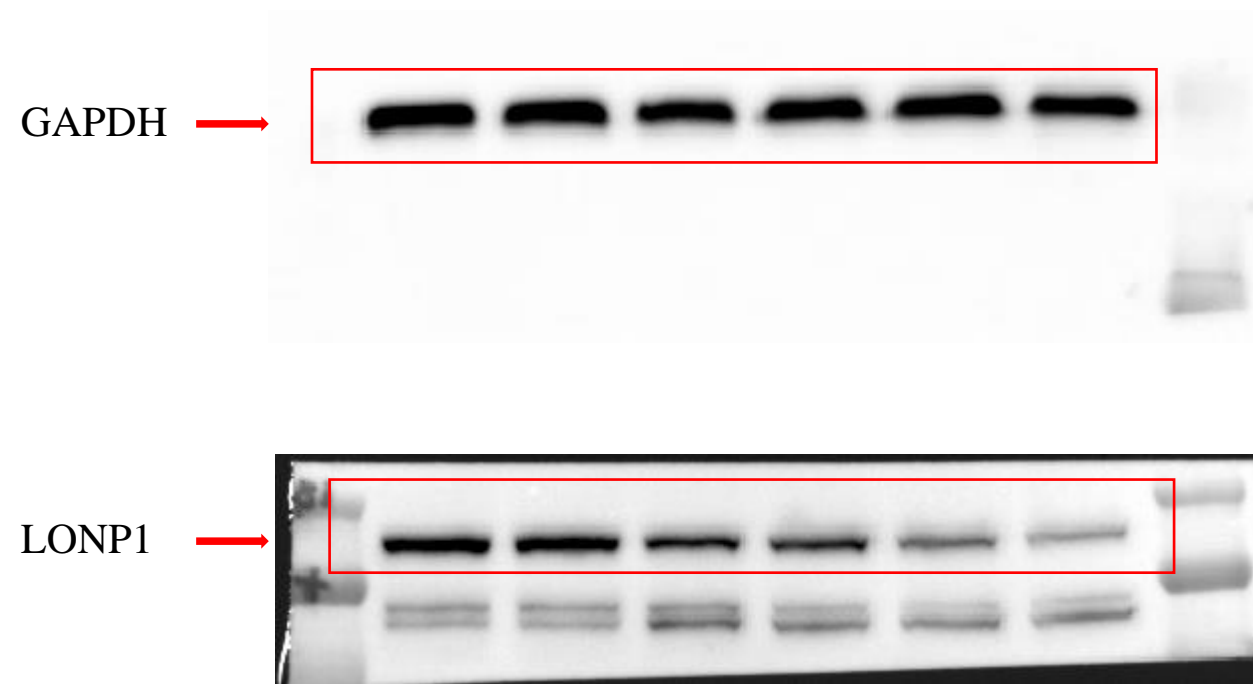

Supplement: Supplementary file 7 — Source Data for Figure 1 [file EMMM-15-e16581-s004.zip › Figure 1/1D-E/western gel.pdf]

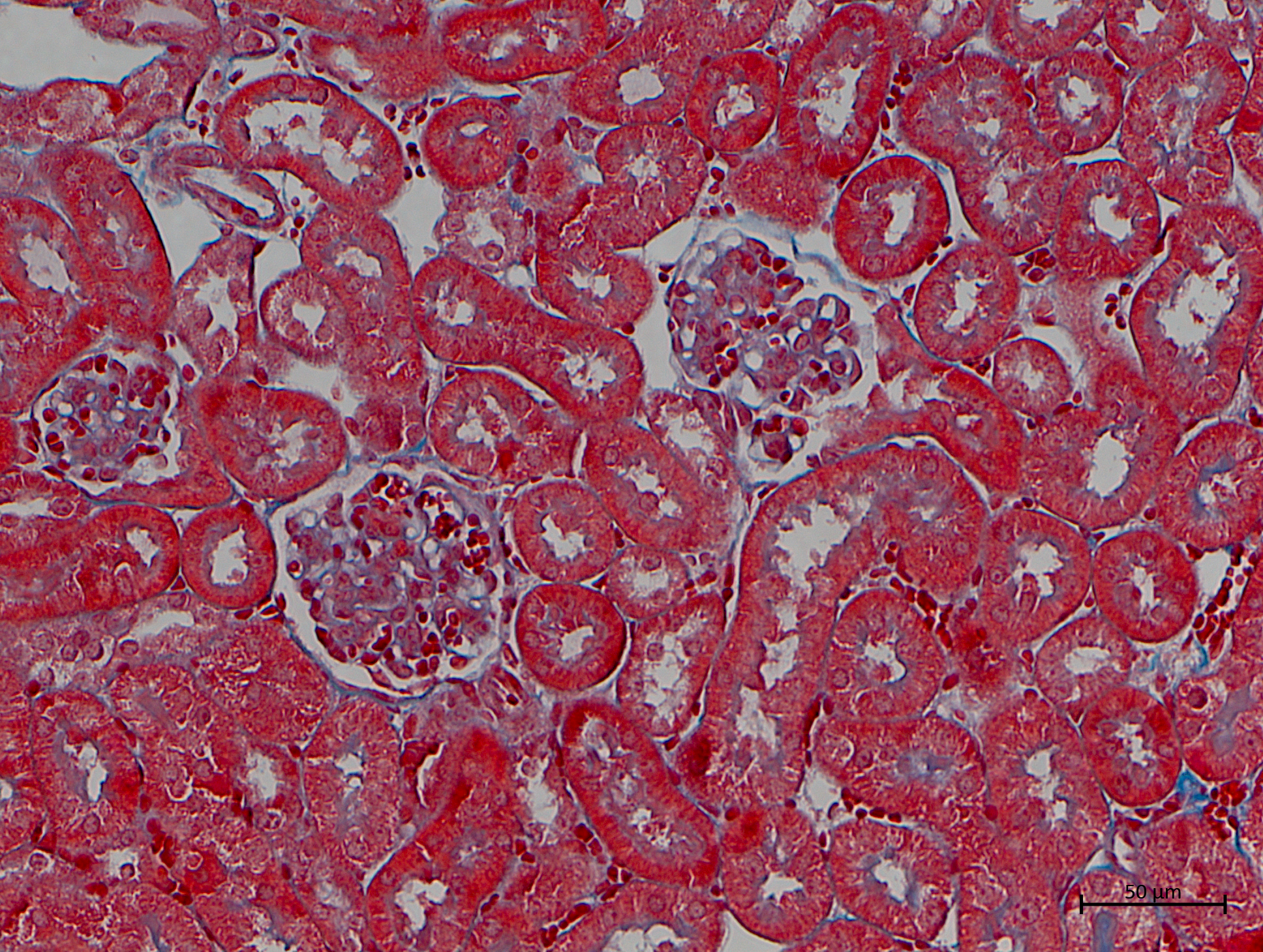

Supplement: Supplementary file 7 — Source Data for Figure 1 [file EMMM-15-e16581-s004.zip › Figure 1/1F/Sham WT.tif]

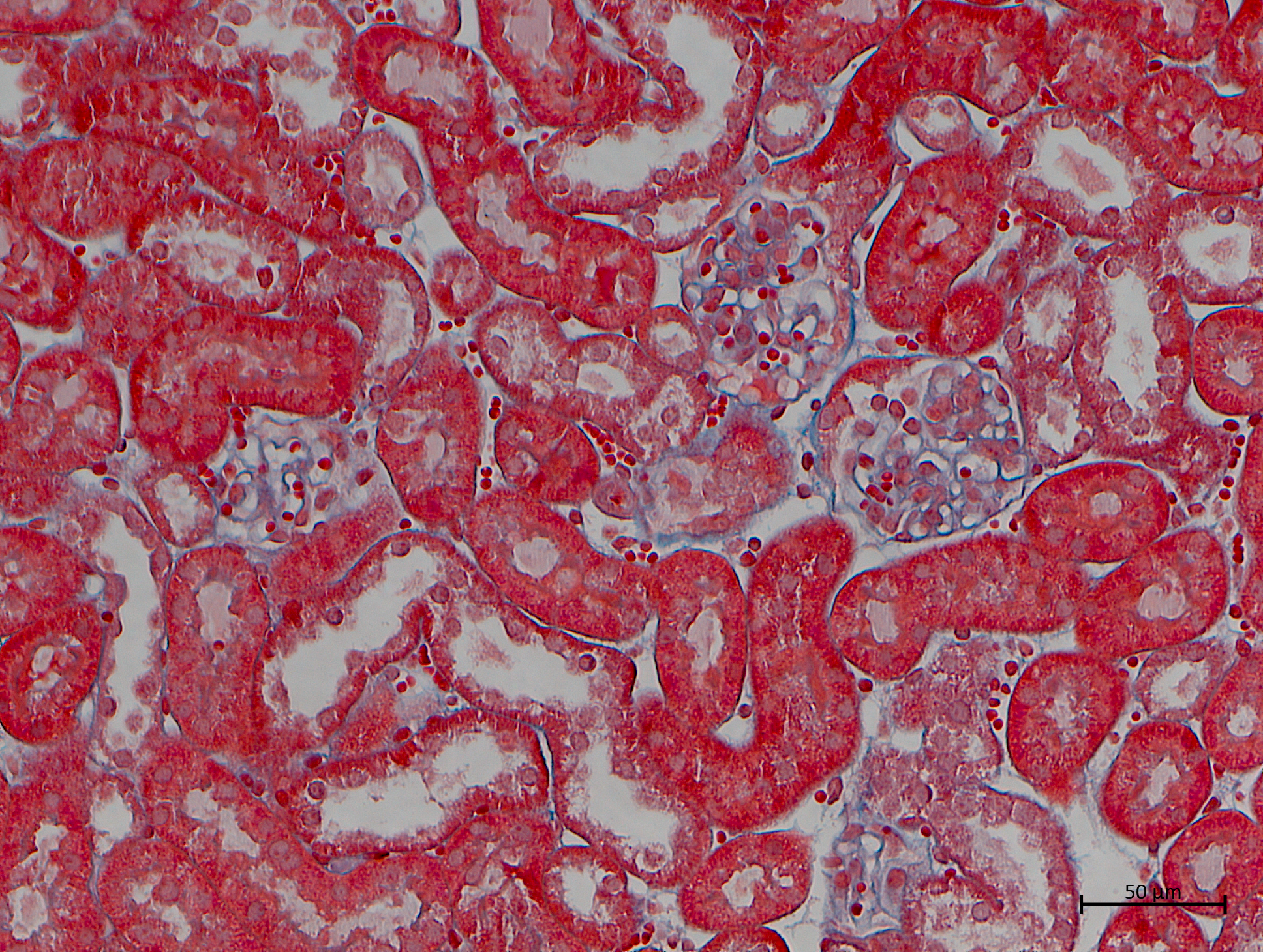

Supplement: Supplementary file 7 — Source Data for Figure 1 [file EMMM-15-e16581-s004.zip › Figure 1/1F/Sham cKI.tif]

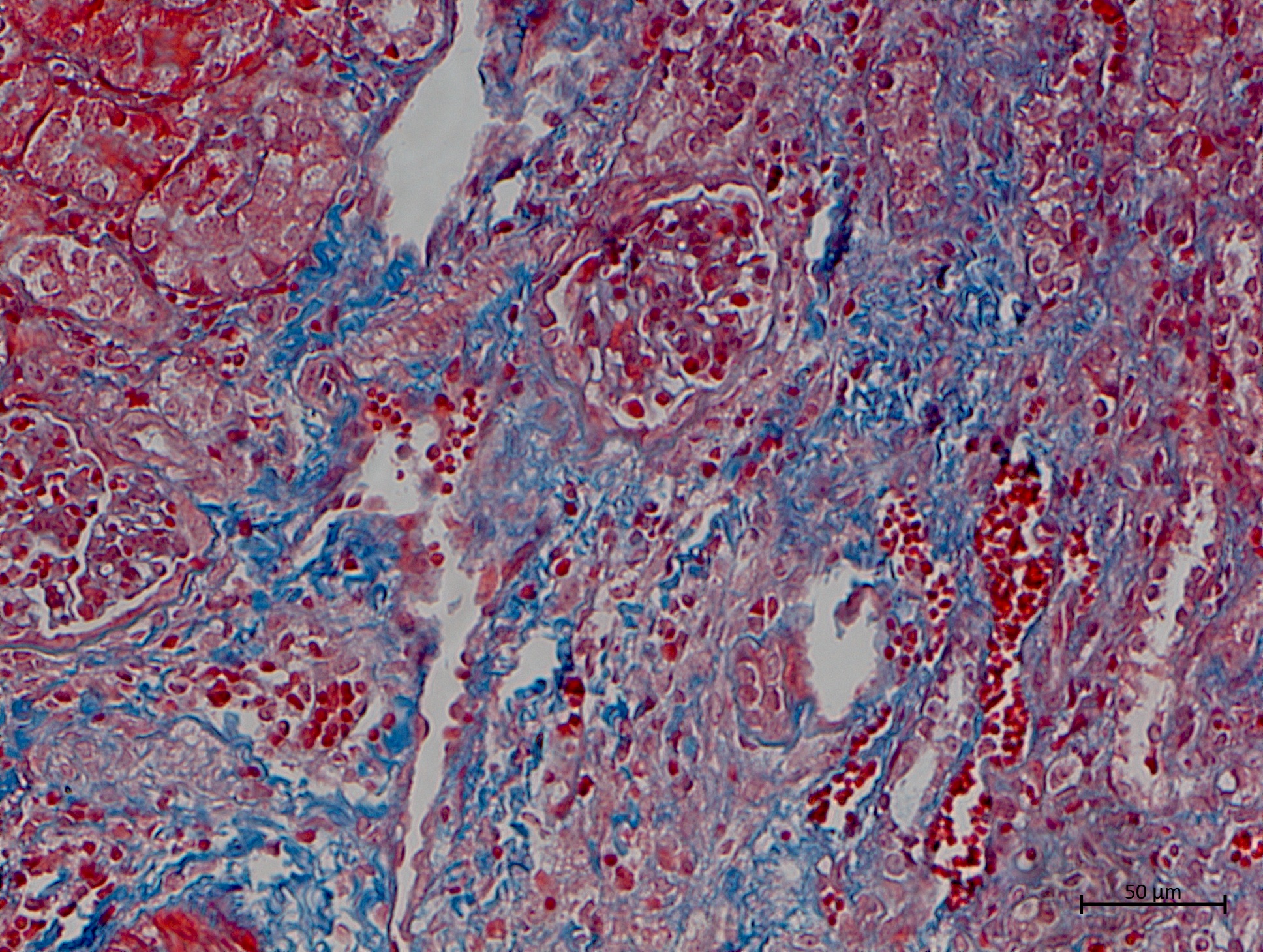

Supplement: Supplementary file 7 — Source Data for Figure 1 [file EMMM-15-e16581-s004.zip › Figure 1/1F/UUO WT.tif]

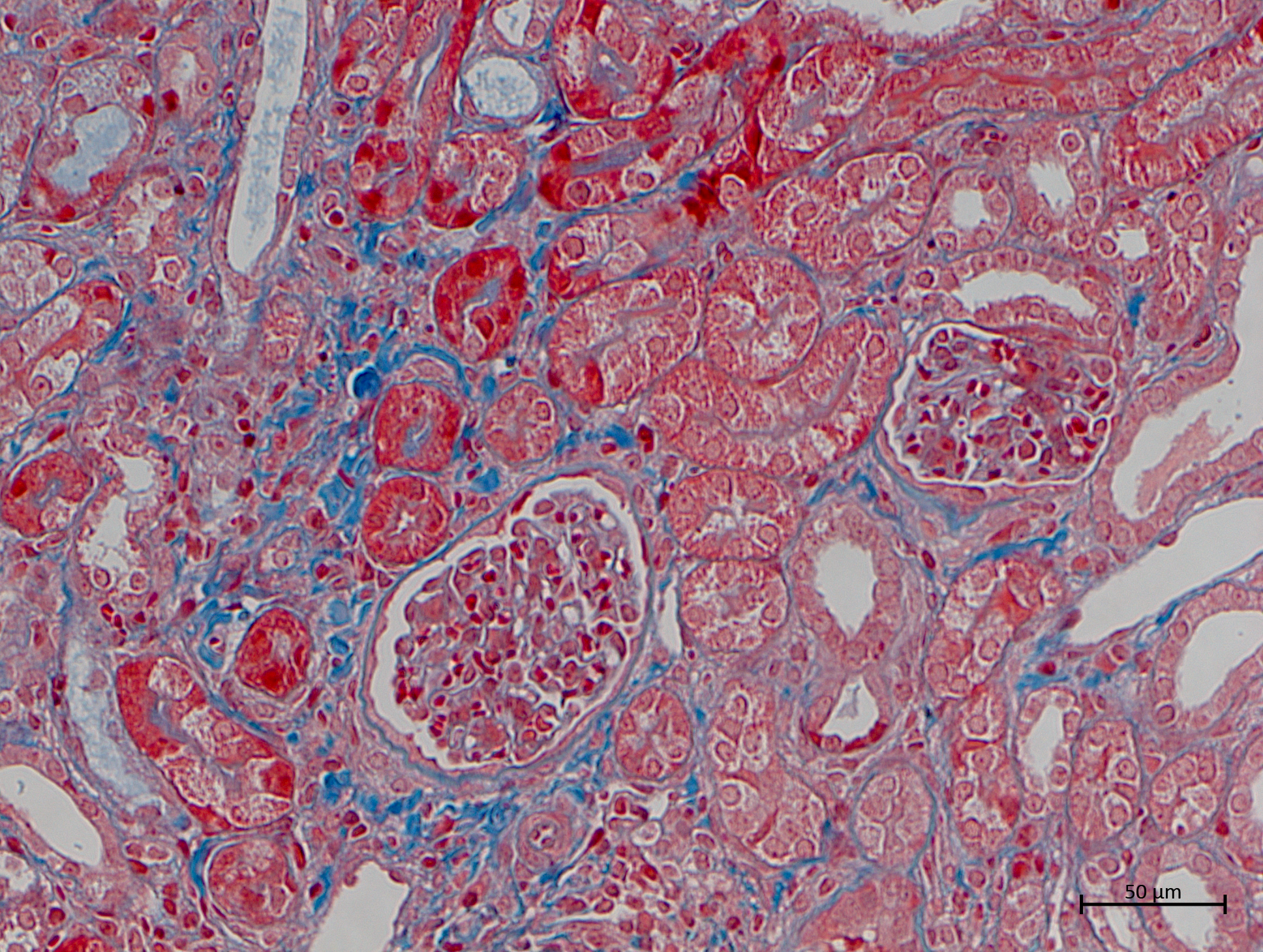

Supplement: Supplementary file 7 — Source Data for Figure 1 [file EMMM-15-e16581-s004.zip › Figure 1/1F/UUO cKI.tif]

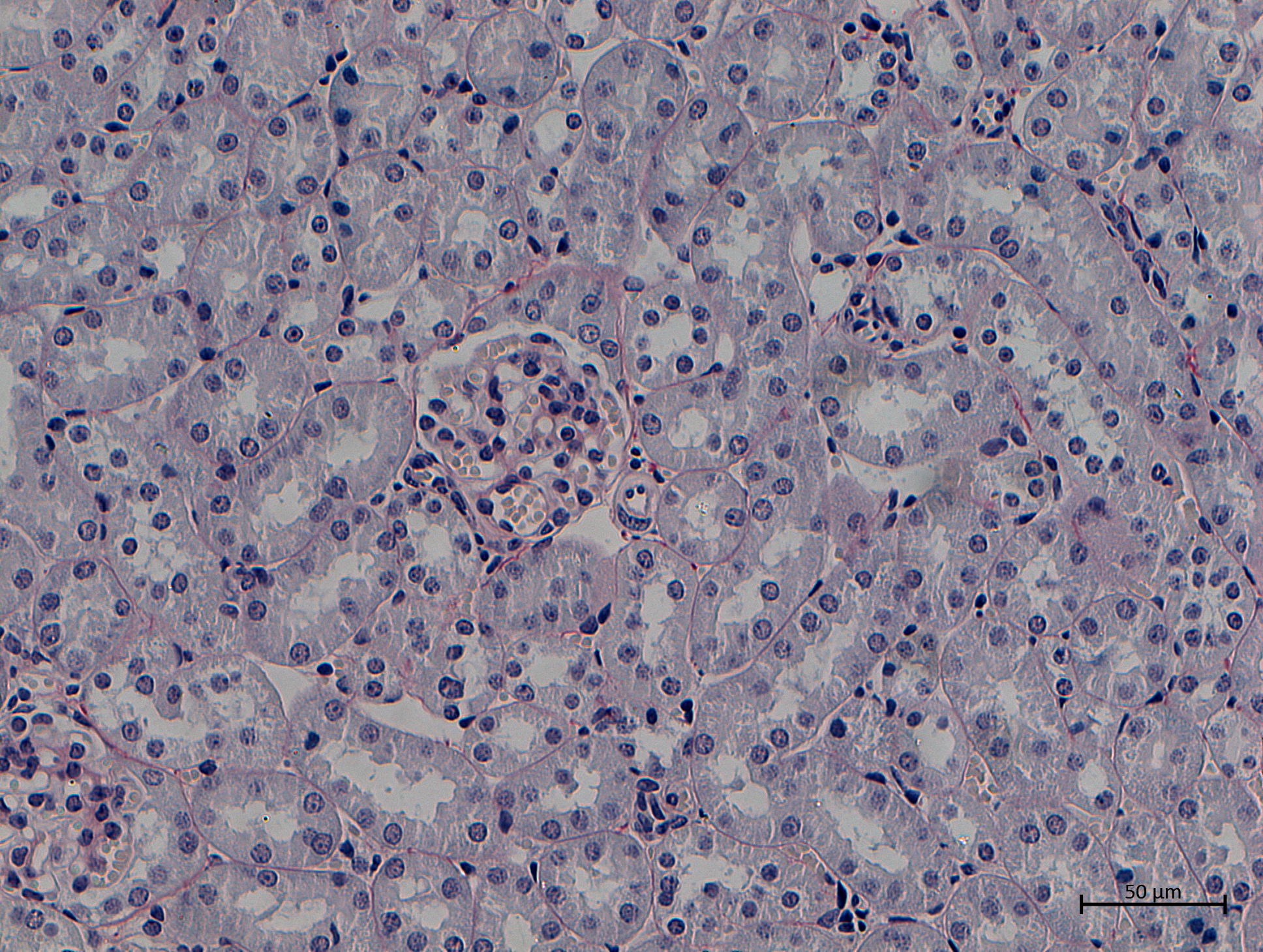

Supplement: Supplementary file 7 — Source Data for Figure 1 [file EMMM-15-e16581-s004.zip › Figure 1/1G-H/Sham WT.tif]

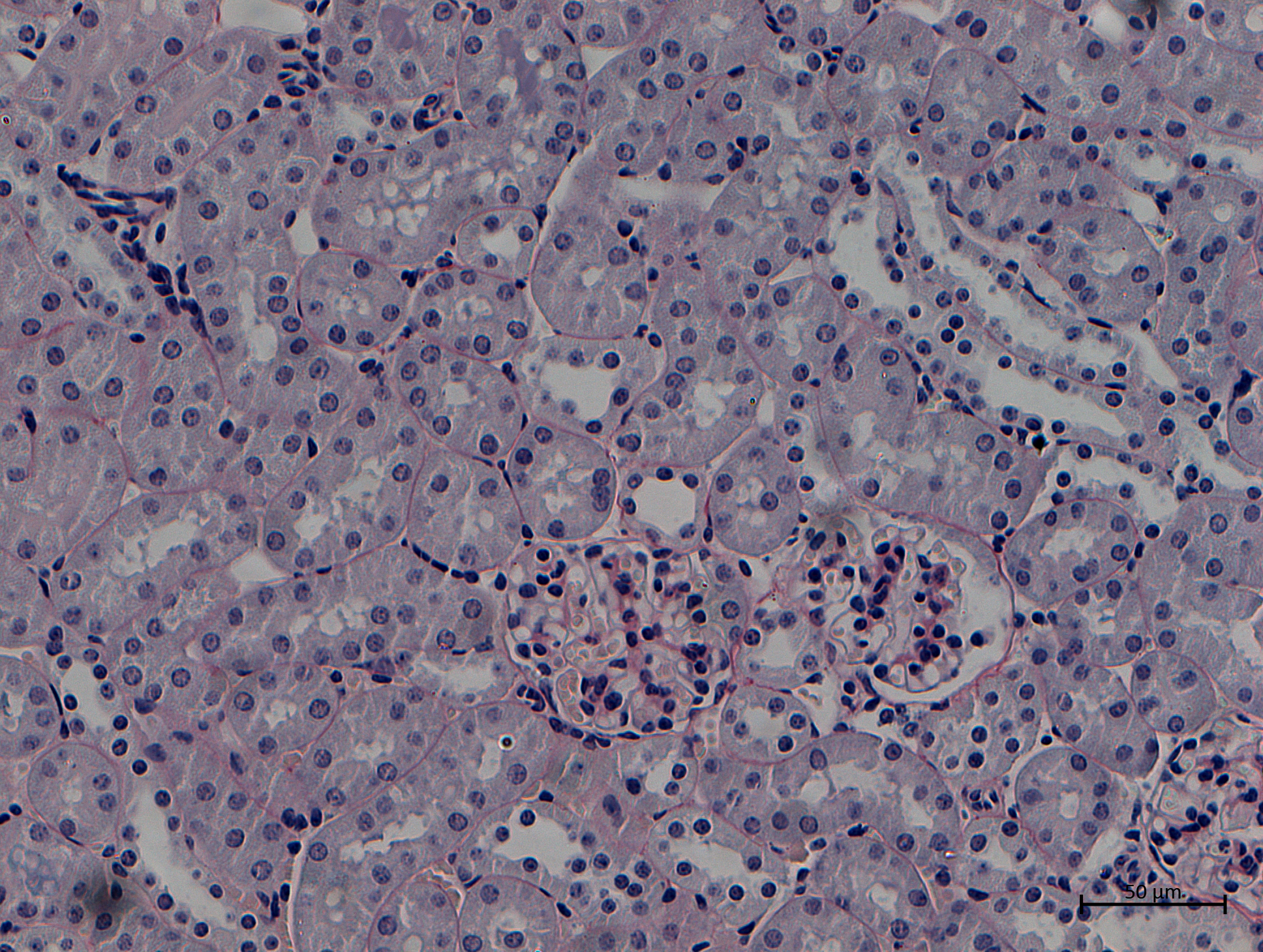

Supplement: Supplementary file 7 — Source Data for Figure 1 [file EMMM-15-e16581-s004.zip › Figure 1/1G-H/Sham cKI.tif]

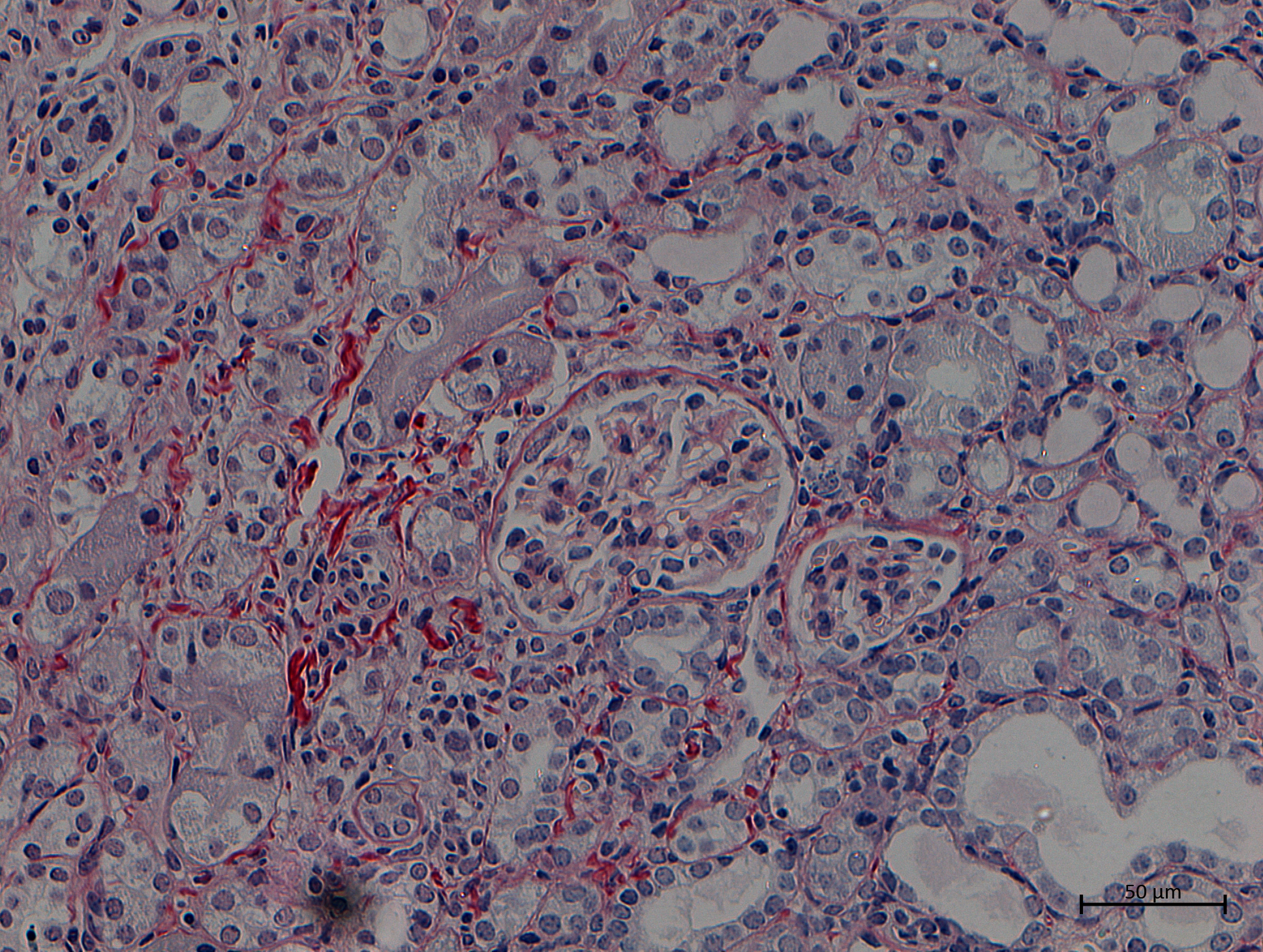

Supplement: Supplementary file 7 — Source Data for Figure 1 [file EMMM-15-e16581-s004.zip › Figure 1/1G-H/UUO CKI.tif]

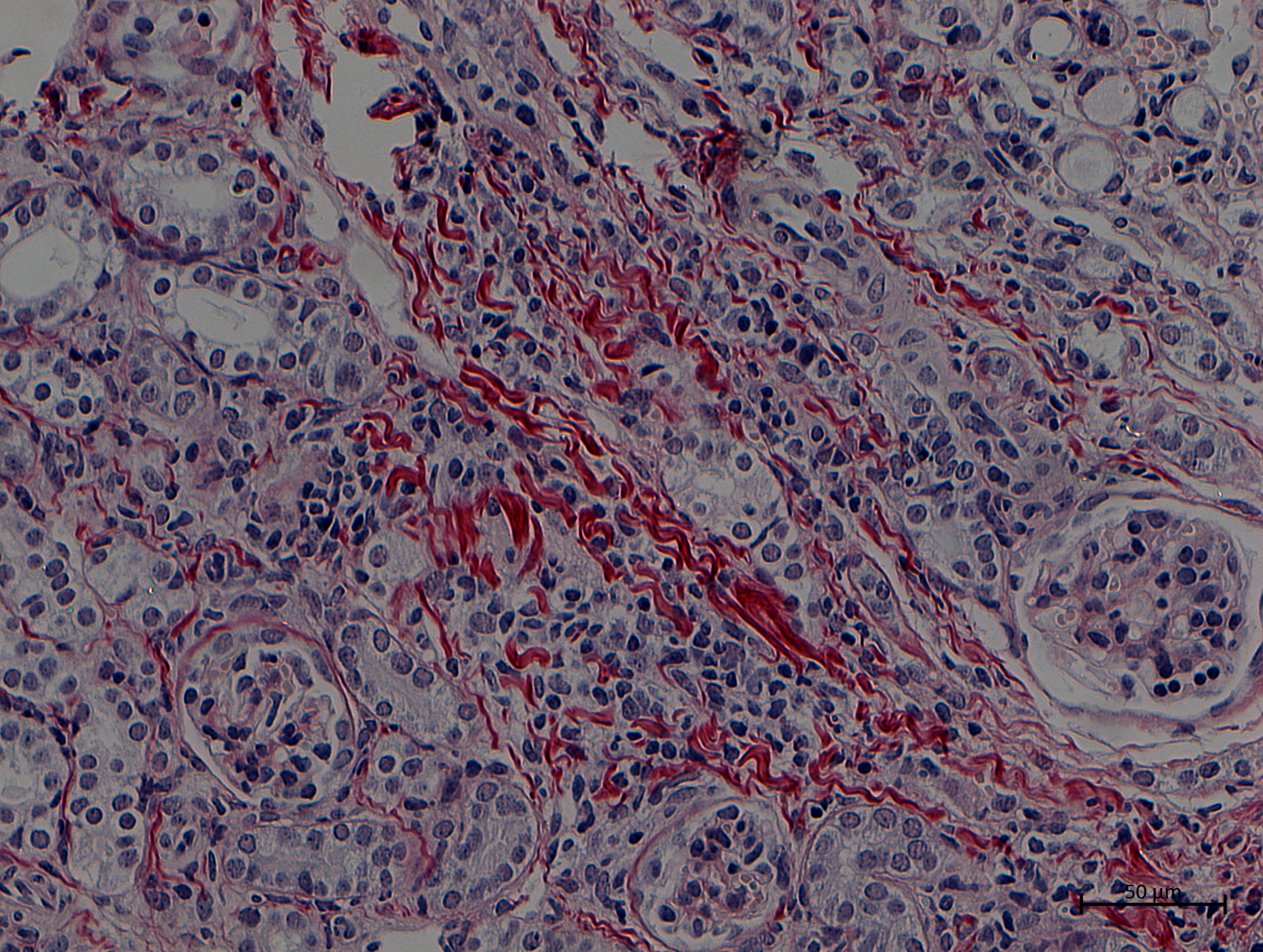

Supplement: Supplementary file 7 — Source Data for Figure 1 [file EMMM-15-e16581-s004.zip › Figure 1/1G-H/UUO WT.tif]

Fig 1J

FN1

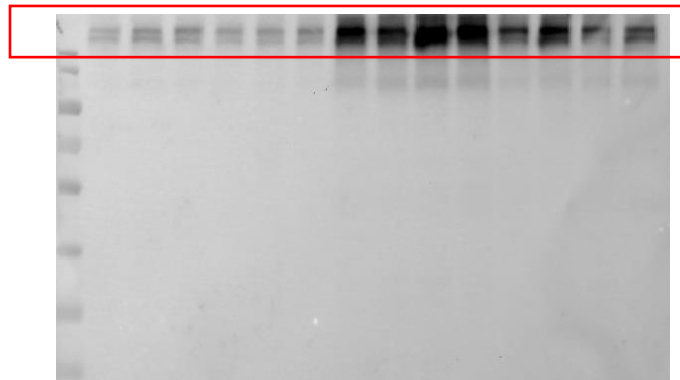

Collagen III

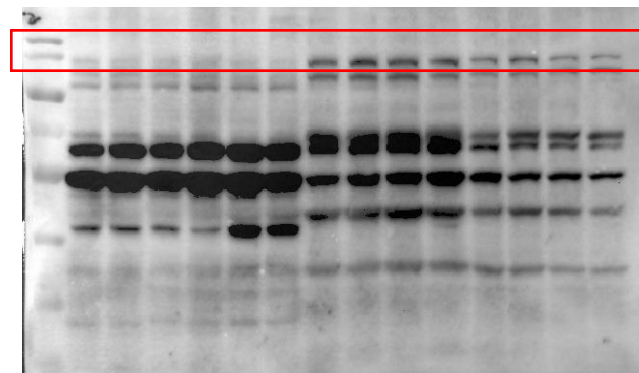

Collagen I

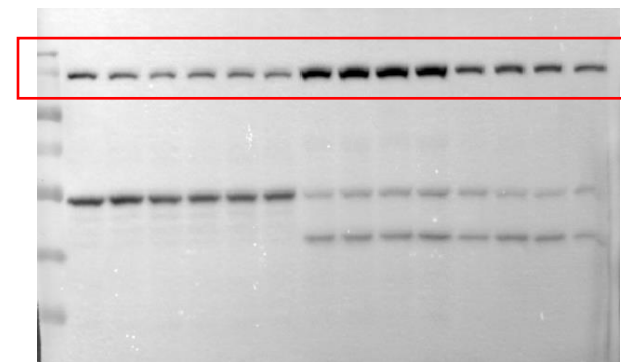

GAPDH

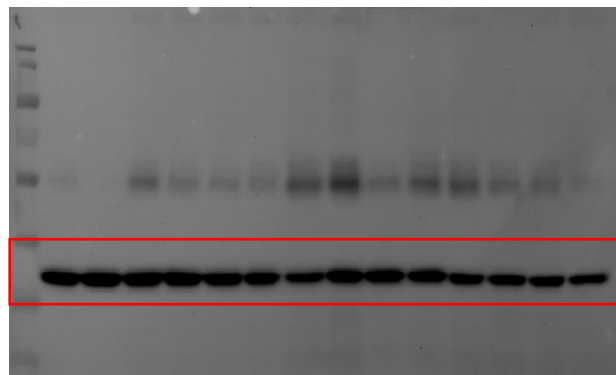

TGF- $\beta$ 1

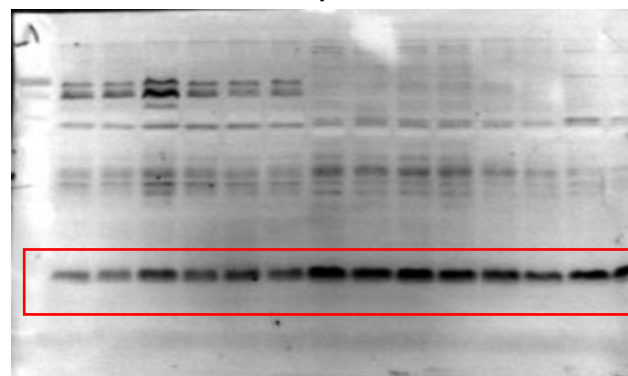

GAPDH

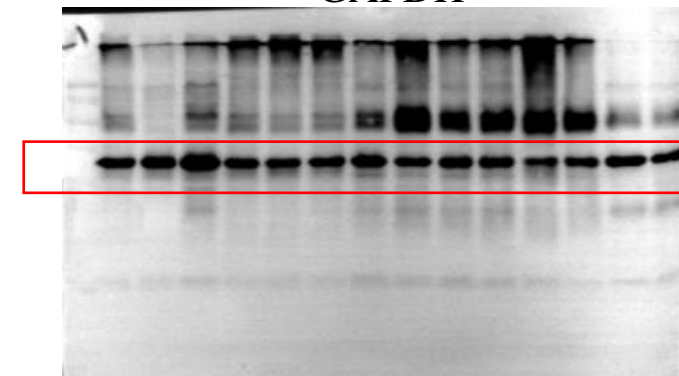

Supplement: Supplementary file 7 — Source Data for Figure 1 [file EMMM-15-e16581-s004.zip › Figure 1/1J/western gel.pdf]

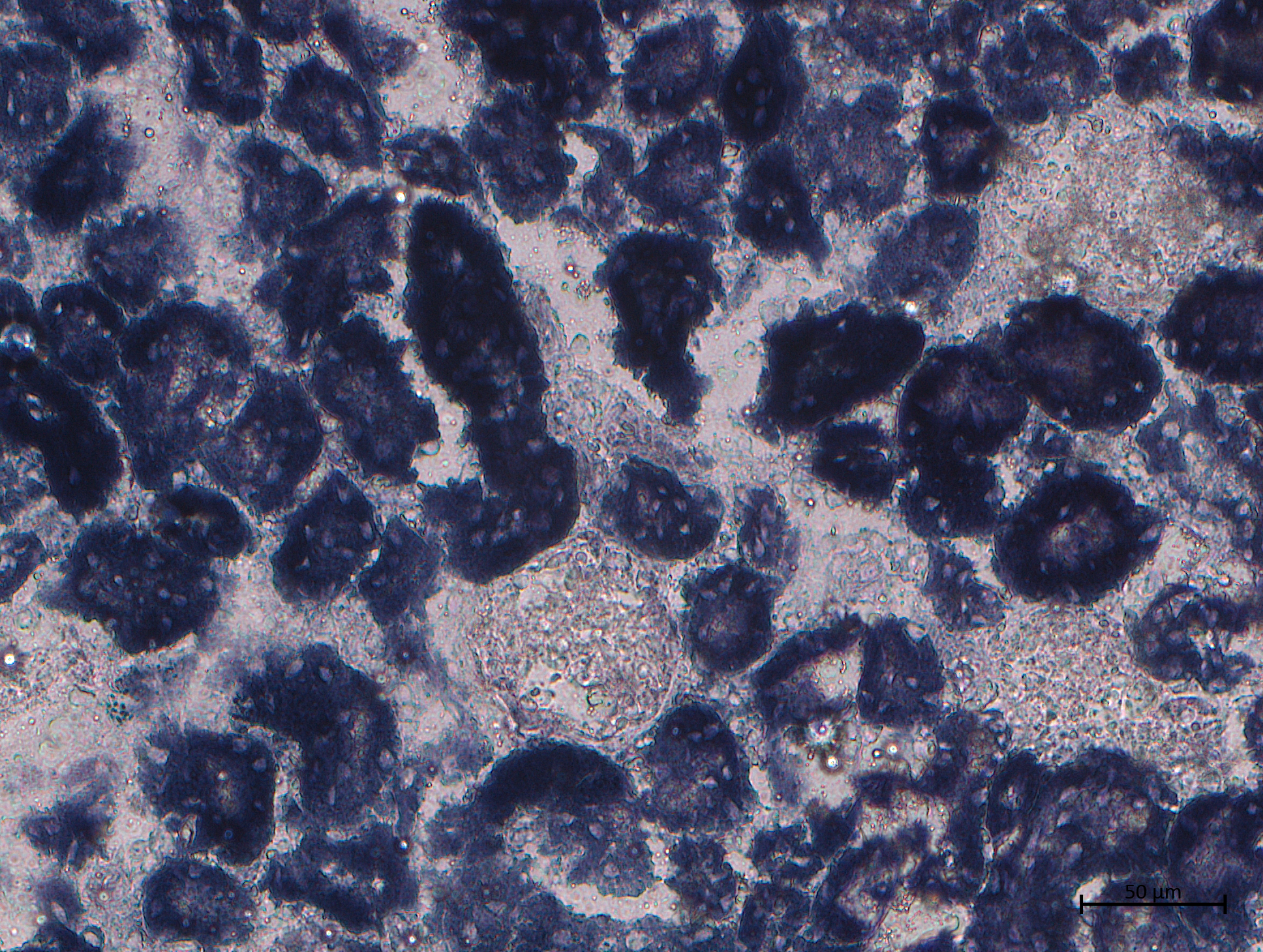

Supplement: Supplementary file 7 — Source Data for Figure 1 [file EMMM-15-e16581-s004.zip › Figure 1/1M/Sham WT.tif]

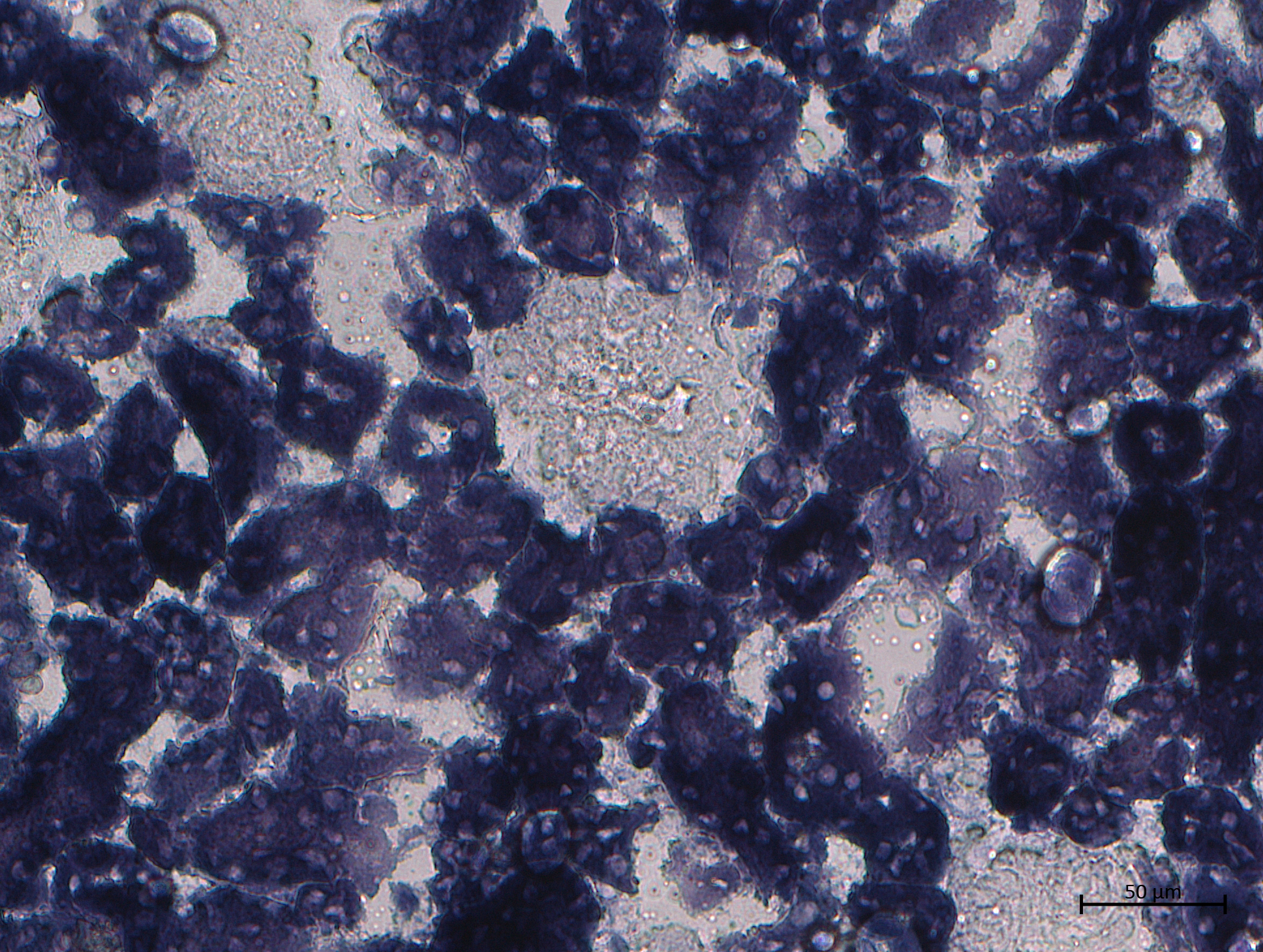

Supplement: Supplementary file 7 — Source Data for Figure 1 [file EMMM-15-e16581-s004.zip › Figure 1/1M/Sham cKI.tif]

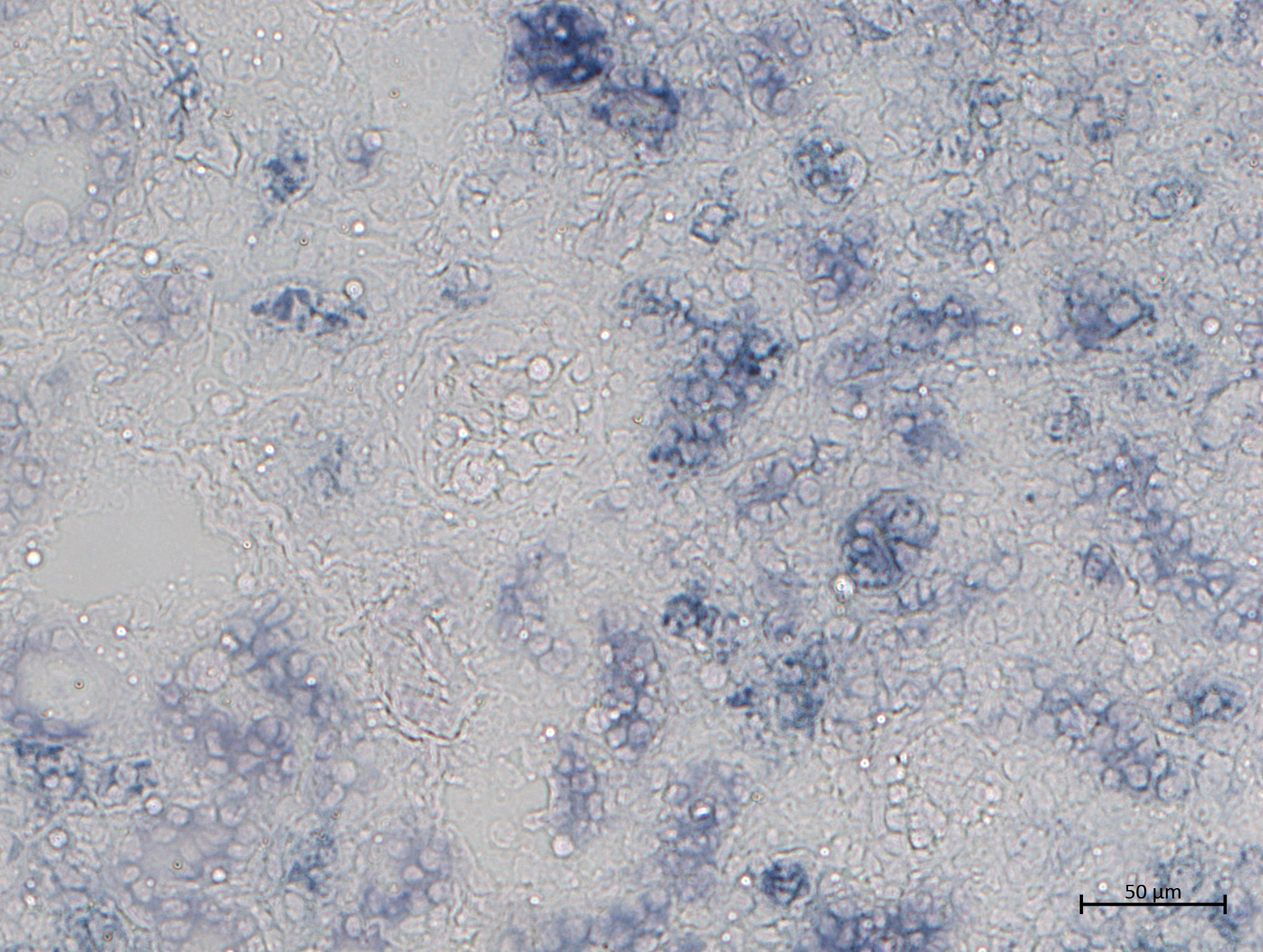

Supplement: Supplementary file 7 — Source Data for Figure 1 [file EMMM-15-e16581-s004.zip › Figure 1/1M/UUO WT.tif]

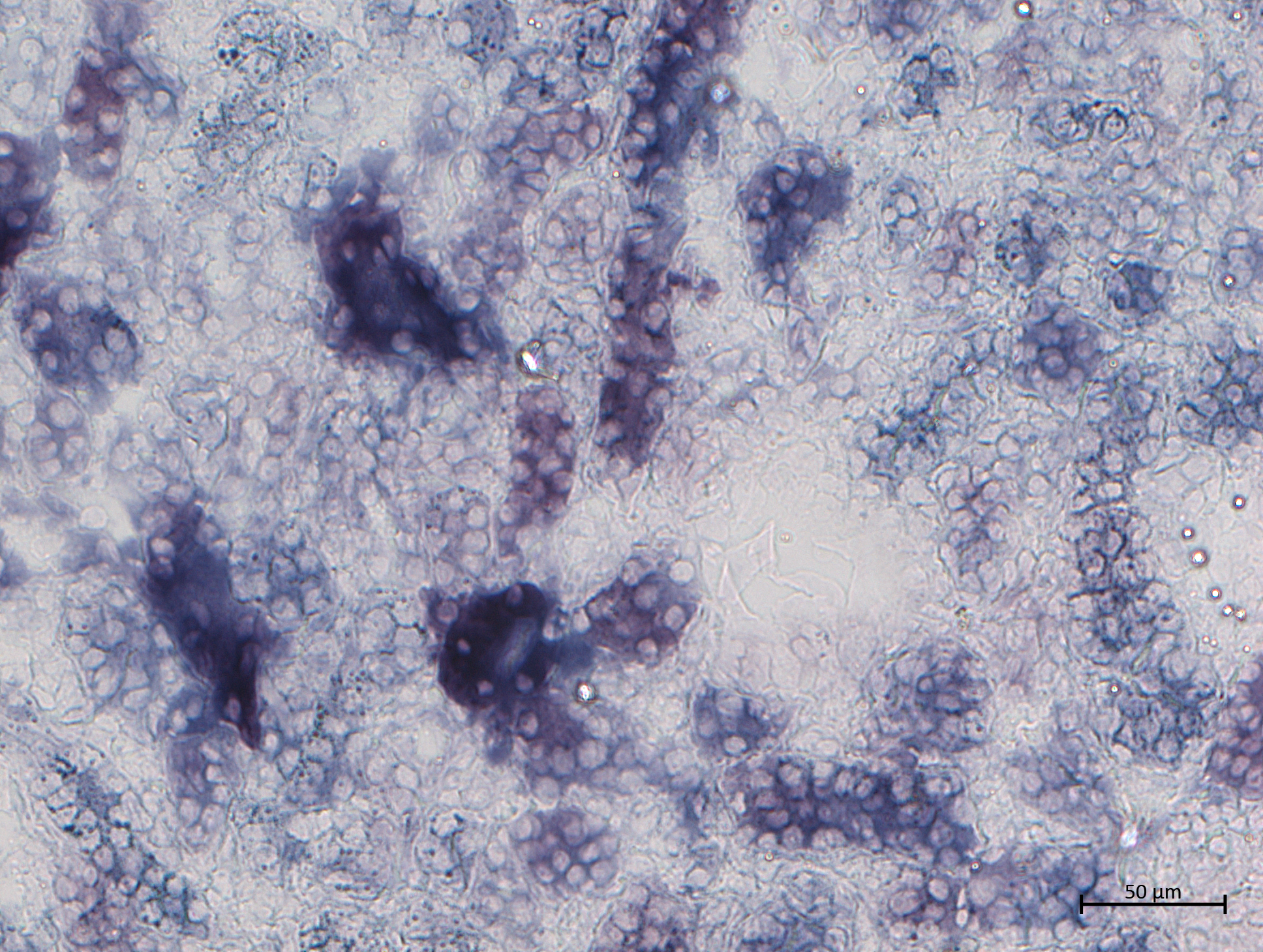

Supplement: Supplementary file 7 — Source Data for Figure 1 [file EMMM-15-e16581-s004.zip › Figure 1/1M/UUO cKI.tif]

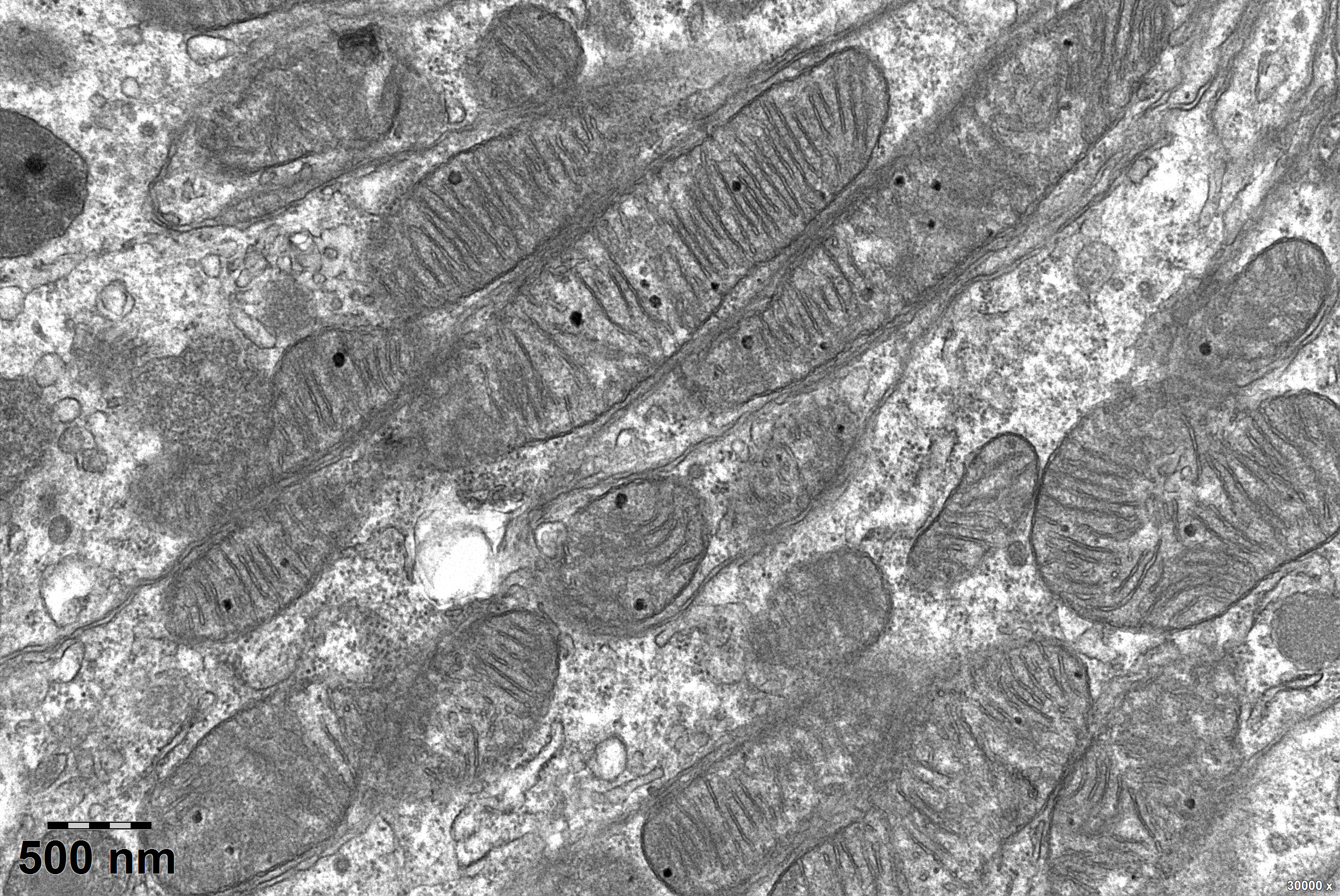

Supplement: Supplementary file 7 — Source Data for Figure 1 [file EMMM-15-e16581-s004.zip › Figure 1/1N/Sham WT.tif]

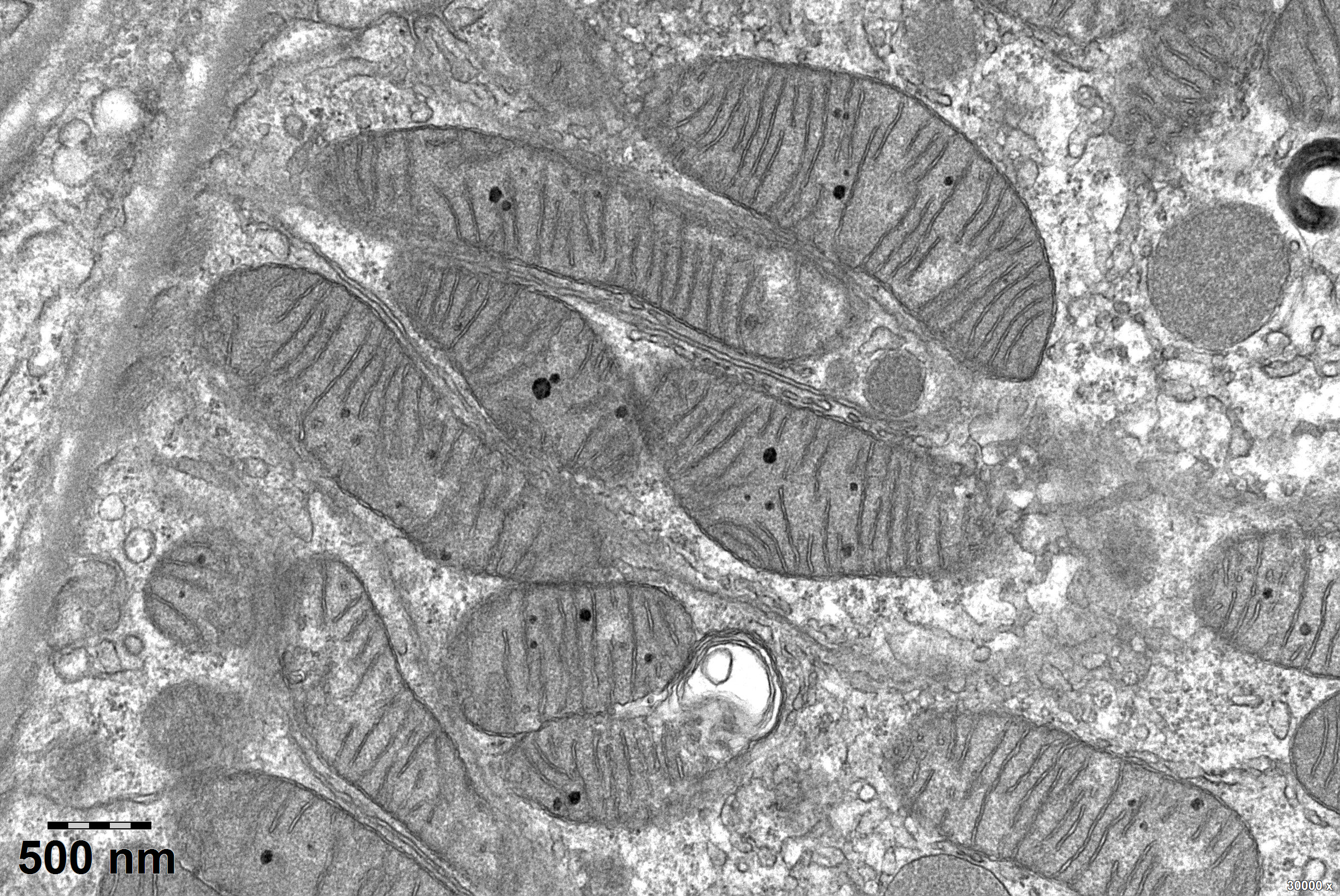

Supplement: Supplementary file 7 — Source Data for Figure 1 [file EMMM-15-e16581-s004.zip › Figure 1/1N/Sham cKI.tif]

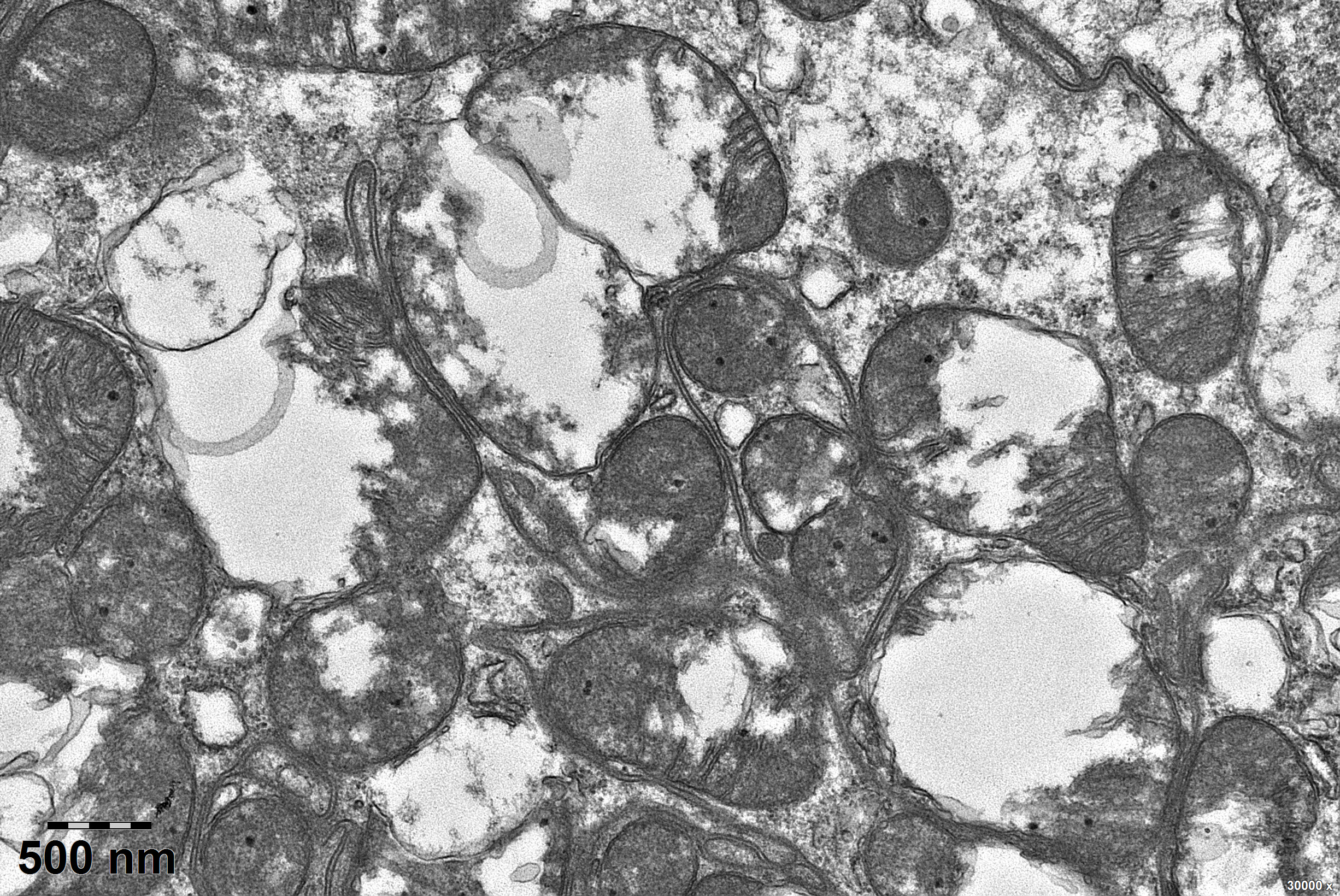

Supplement: Supplementary file 7 — Source Data for Figure 1 [file EMMM-15-e16581-s004.zip › Figure 1/1N/UUO WT.tif]

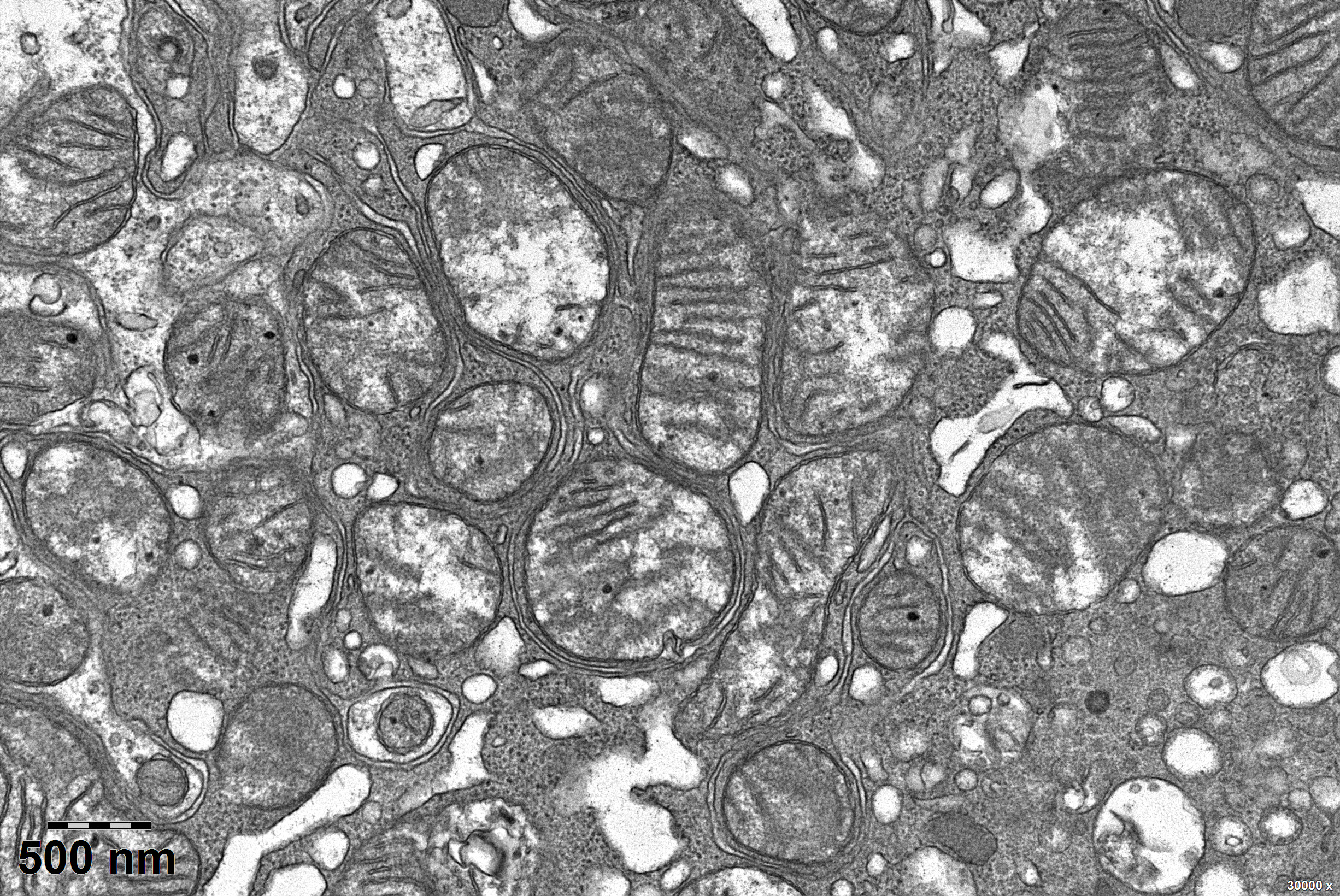

Supplement: Supplementary file 7 — Source Data for Figure 1 [file EMMM-15-e16581-s004.zip › Figure 1/1N/UUO cKI.tif]

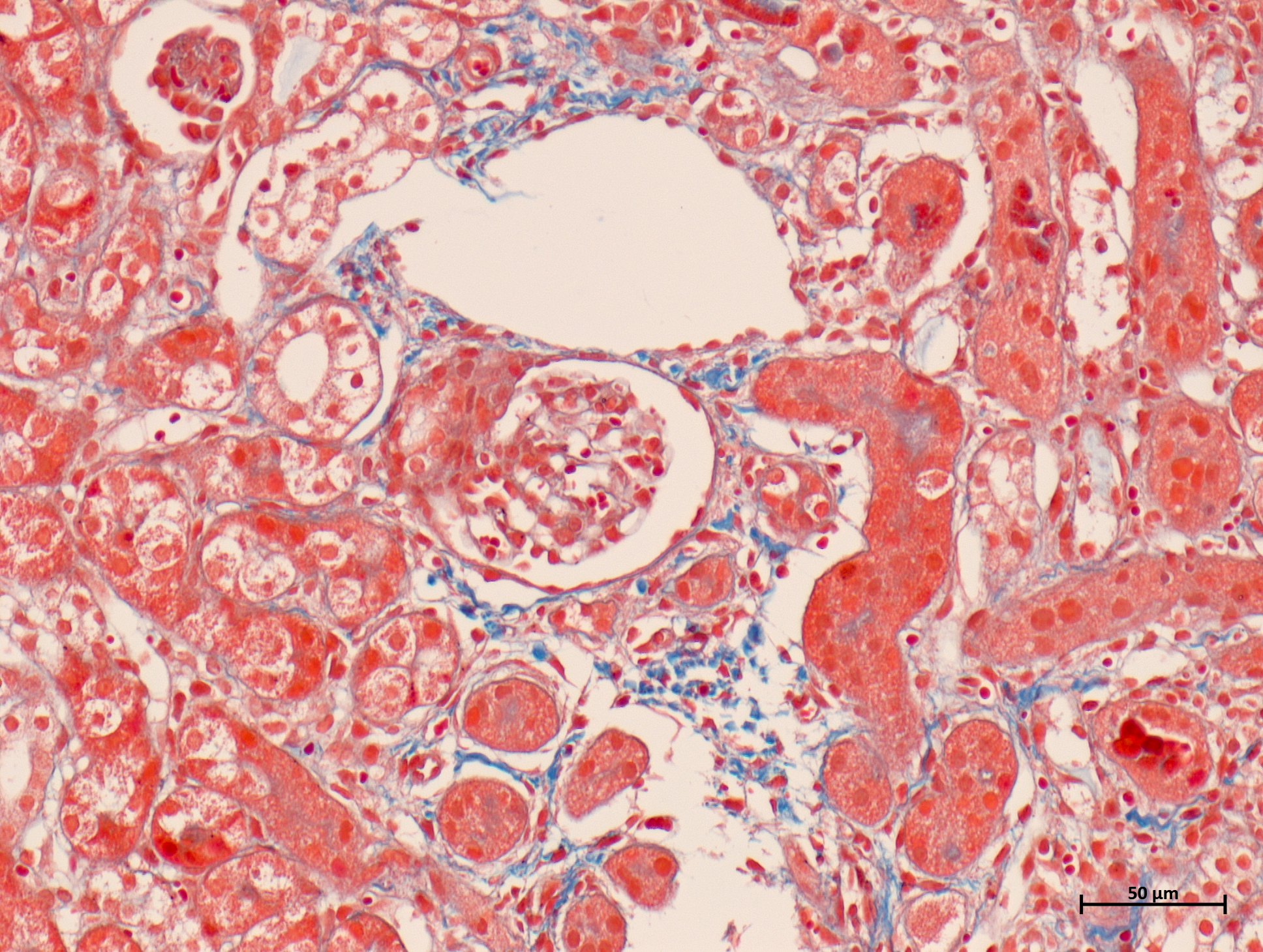

Supplement: Supplementary file 8 — Source Data for Figure 2 [file EMMM-15-e16581-s015.zip › Figure 2/2A/WT+UUO.tif]

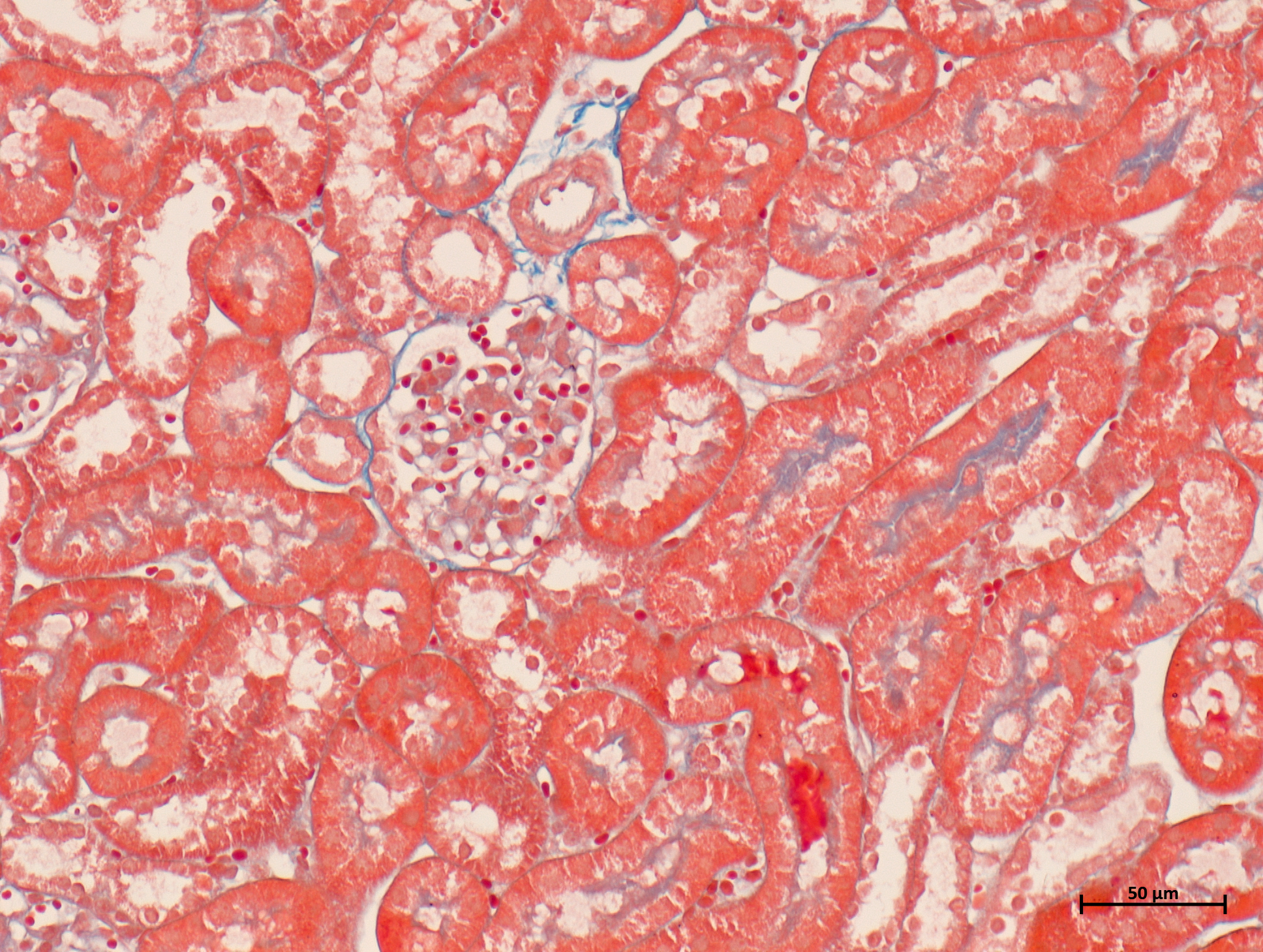

Supplement: Supplementary file 8 — Source Data for Figure 2 [file EMMM-15-e16581-s015.zip › Figure 2/2A/WT.tif]

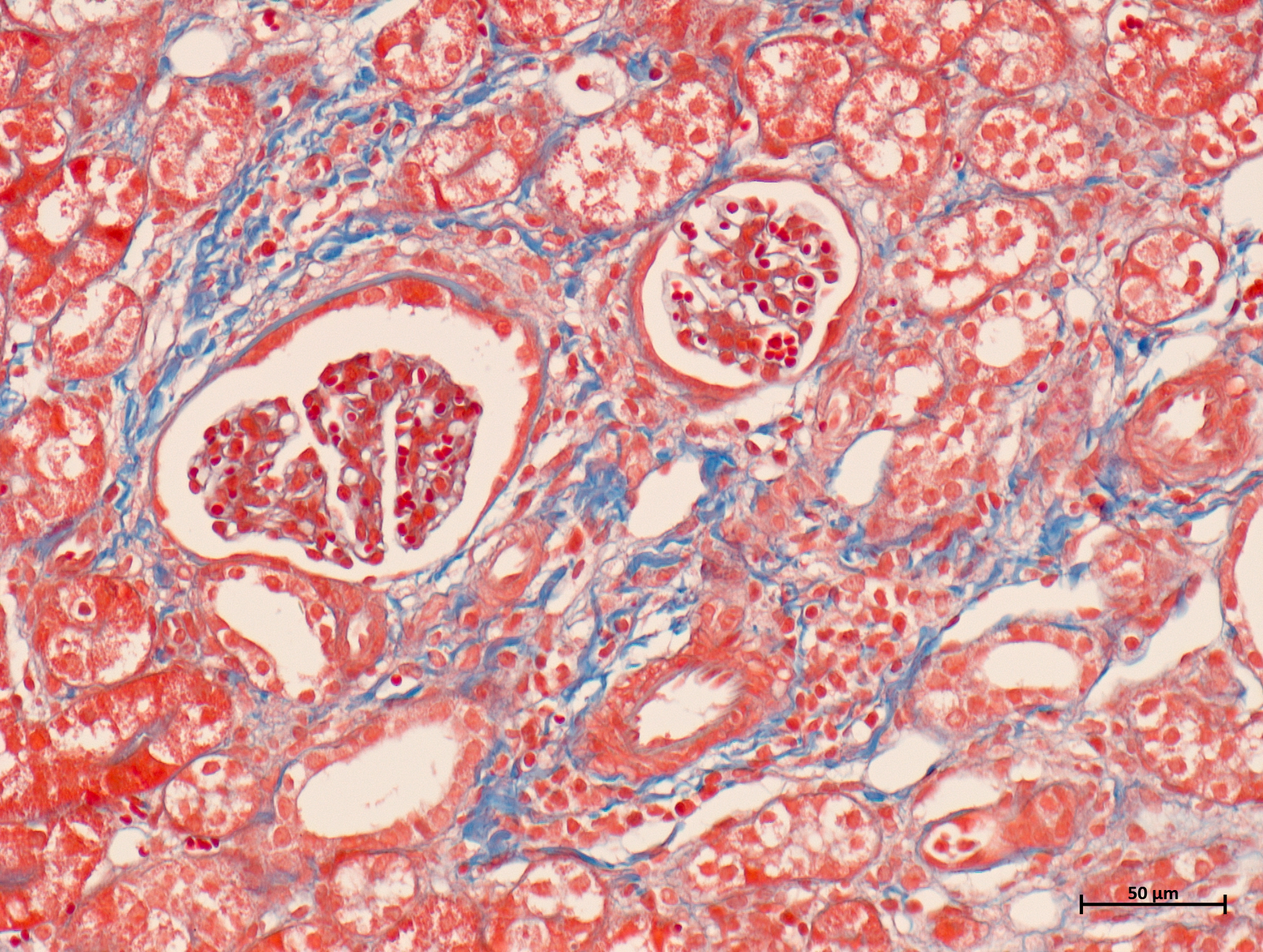

Supplement: Supplementary file 8 — Source Data for Figure 2 [file EMMM-15-e16581-s015.zip › Figure 2/2A/cKO+UUO.tif]

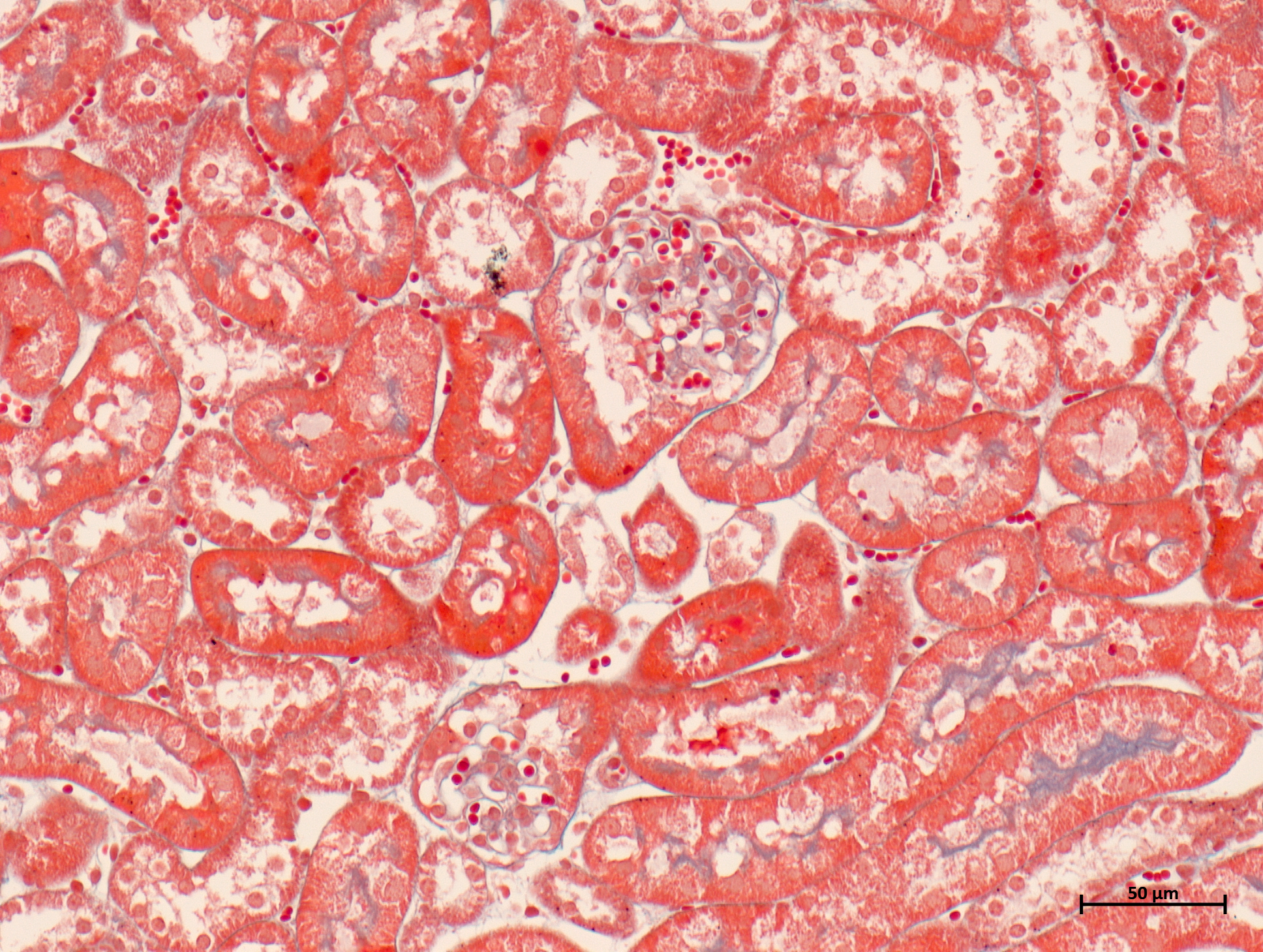

Supplement: Supplementary file 8 — Source Data for Figure 2 [file EMMM-15-e16581-s015.zip › Figure 2/2A/cKO.tif]

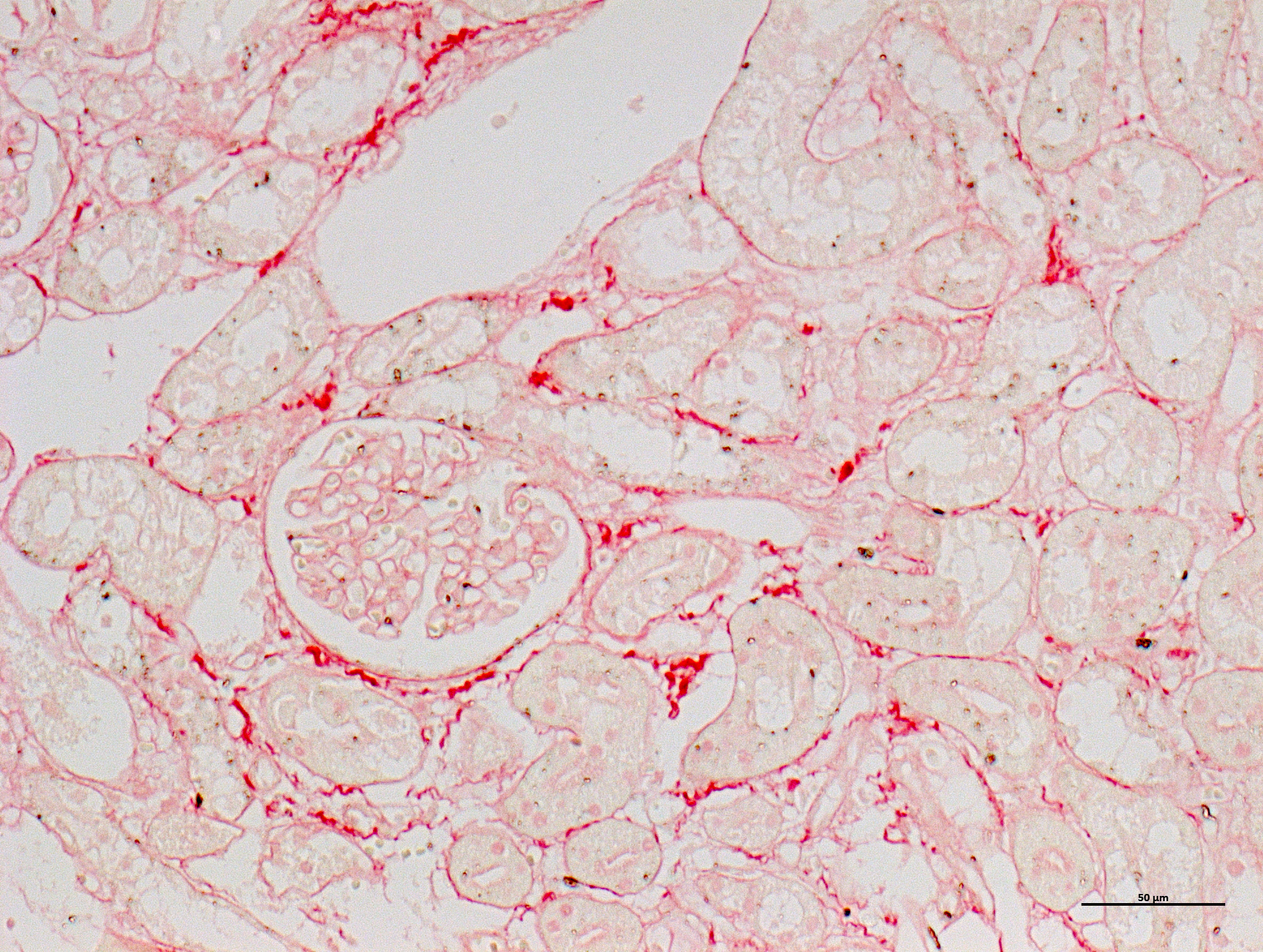

Supplement: Supplementary file 8 — Source Data for Figure 2 [file EMMM-15-e16581-s015.zip › Figure 2/2B-C/WT+UUO.tif]

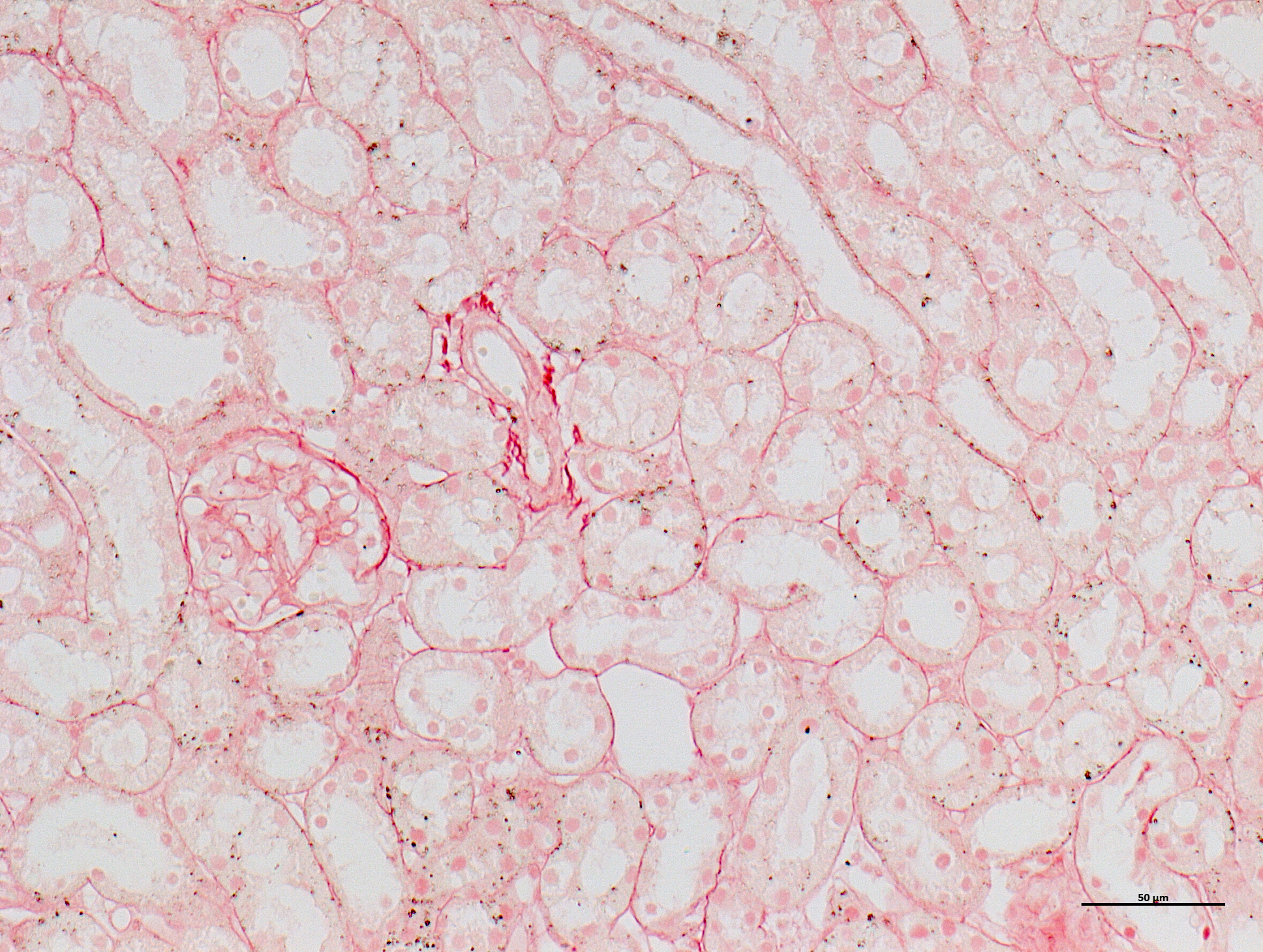

Supplement: Supplementary file 8 — Source Data for Figure 2 [file EMMM-15-e16581-s015.zip › Figure 2/2B-C/WT.tif]

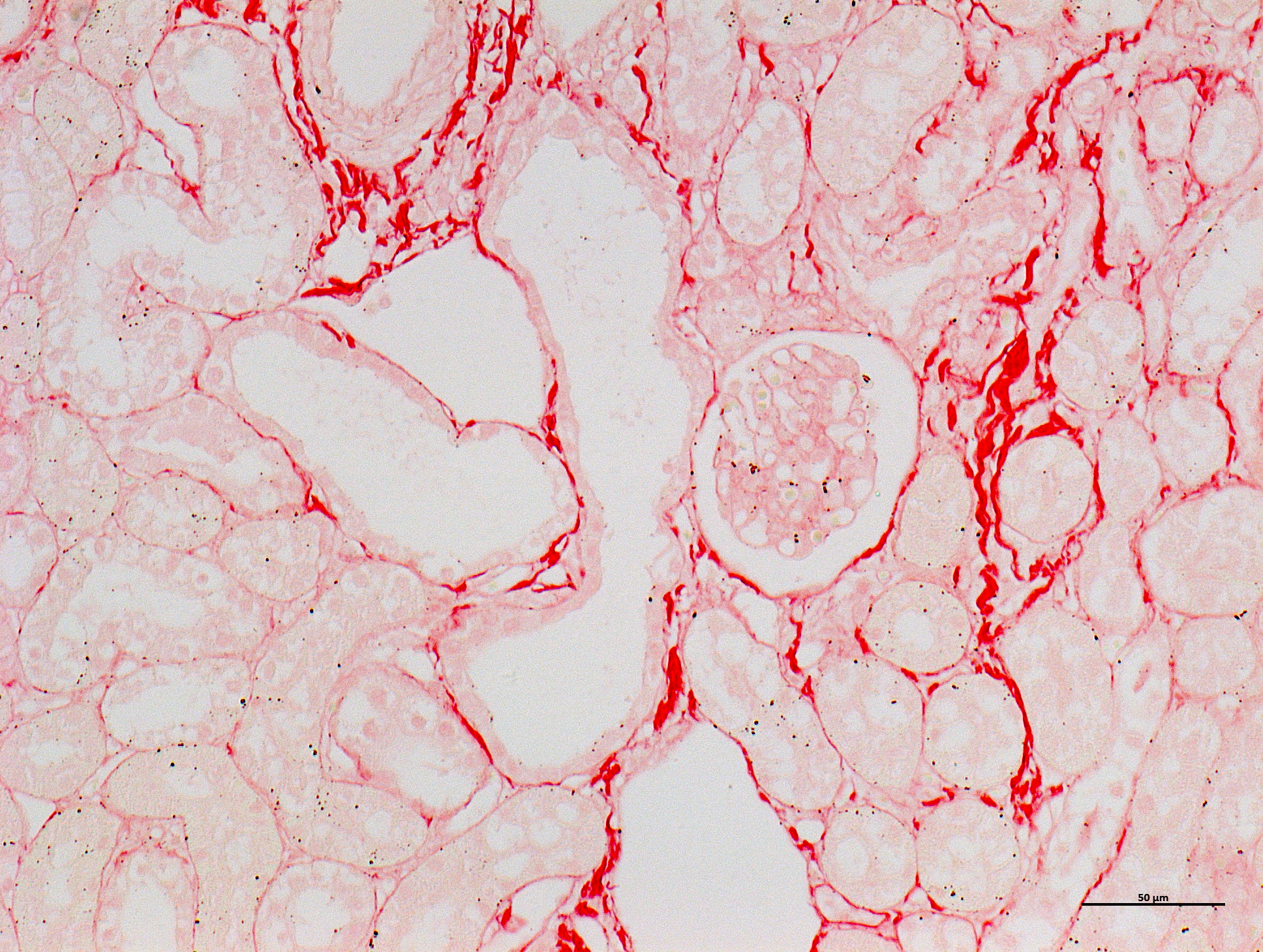

Supplement: Supplementary file 8 — Source Data for Figure 2 [file EMMM-15-e16581-s015.zip › Figure 2/2B-C/cKO+UUO.tif]

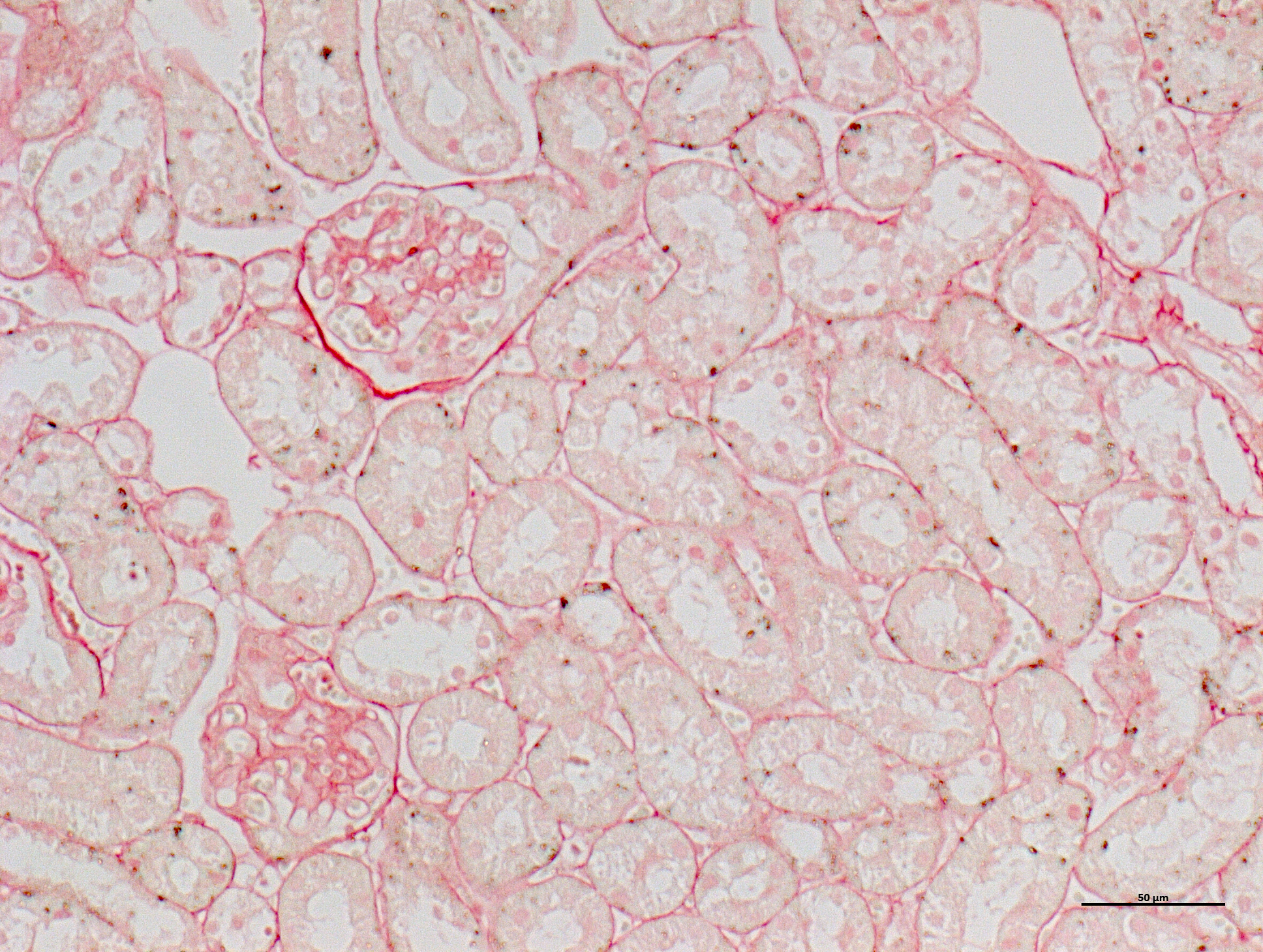

Supplement: Supplementary file 8 — Source Data for Figure 2 [file EMMM-15-e16581-s015.zip › Figure 2/2B-C/cKO.tif]

Fig 2D

FN1

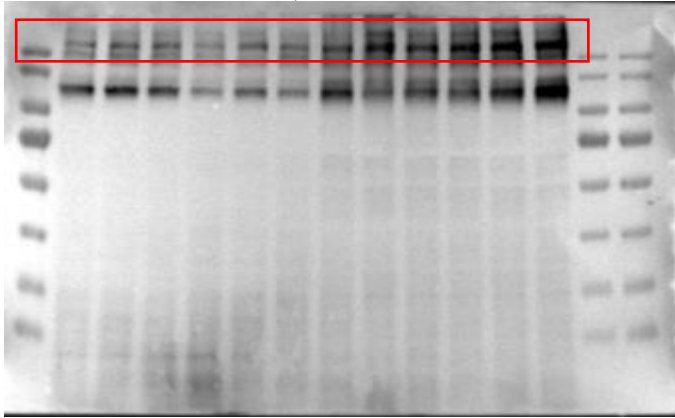

GAPDH

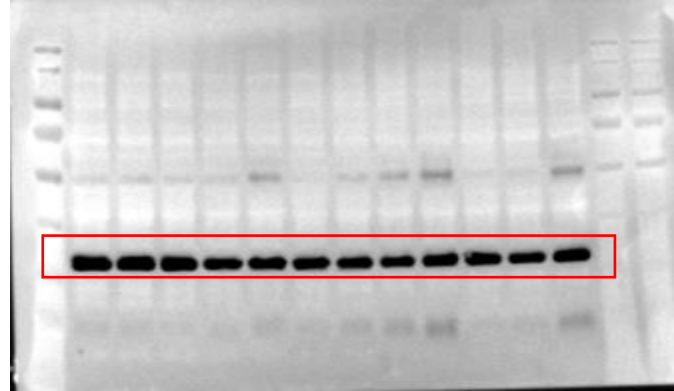

Collagen III

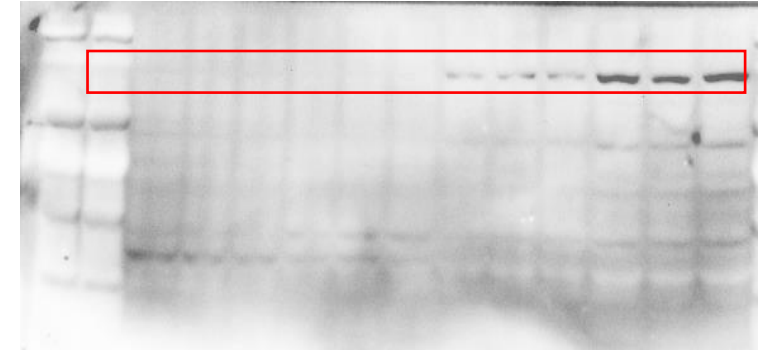

Collagen I

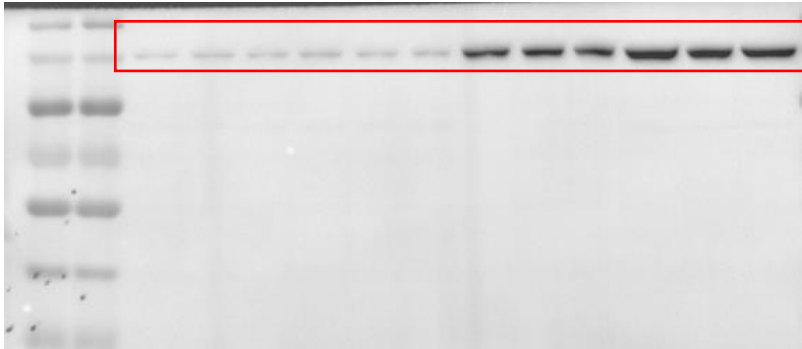

$\alpha$ -SMA

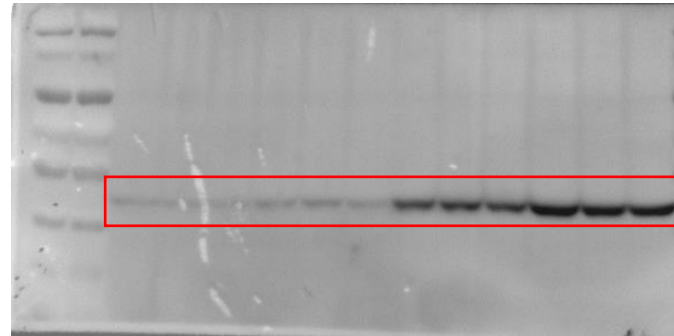

GAPDH

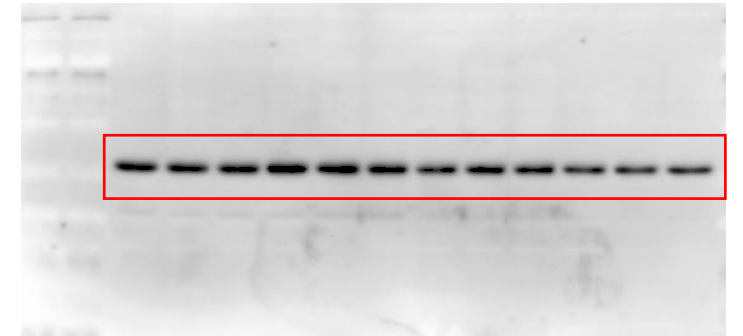

Supplement: Supplementary file 8 — Source Data for Figure 2 [file EMMM-15-e16581-s015.zip › Figure 2/2D-E/western gel.pdf]

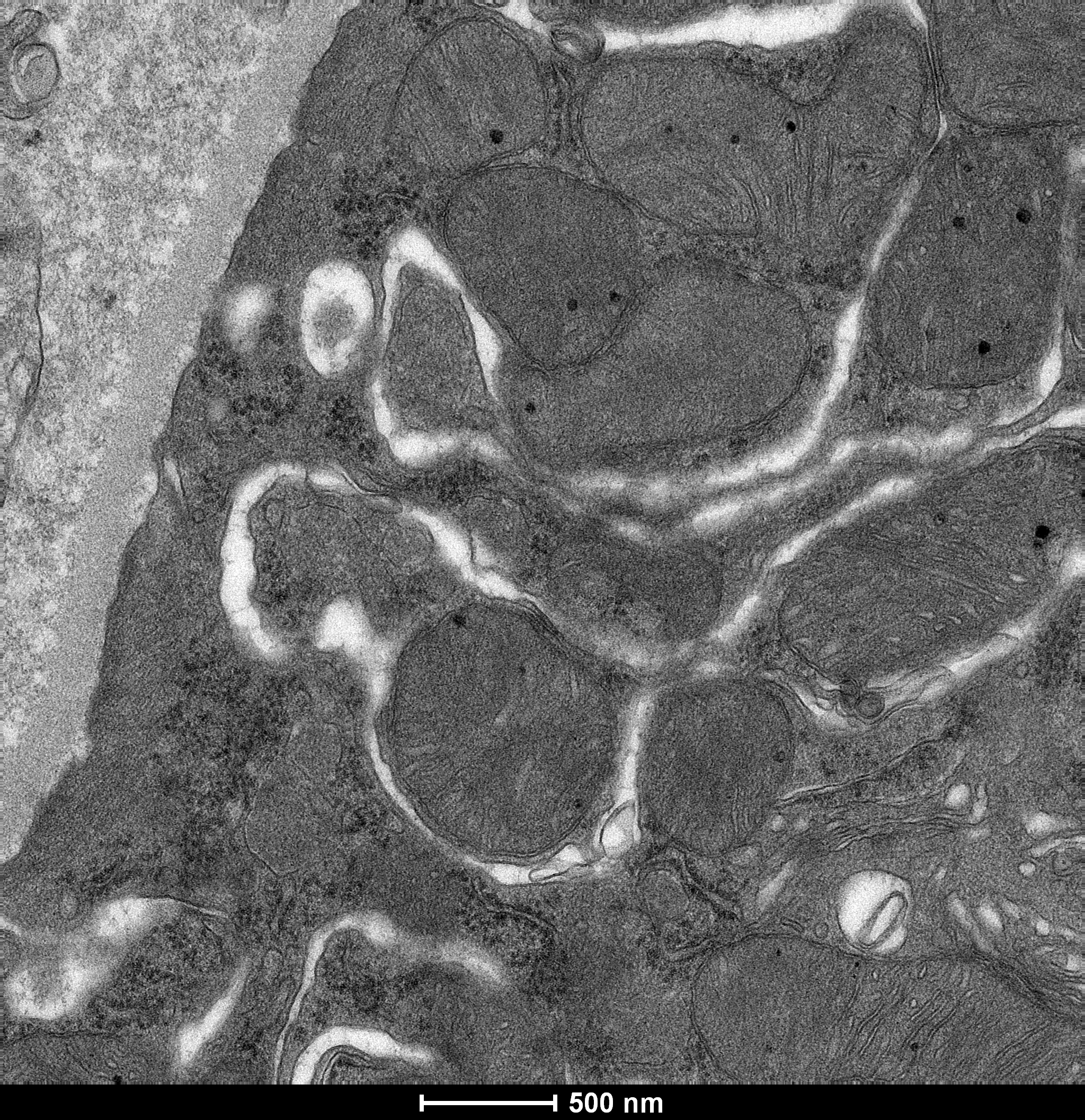

Supplement: Supplementary file 8 — Source Data for Figure 2 [file EMMM-15-e16581-s015.zip › Figure 2/2I/WT+UUO.tif]

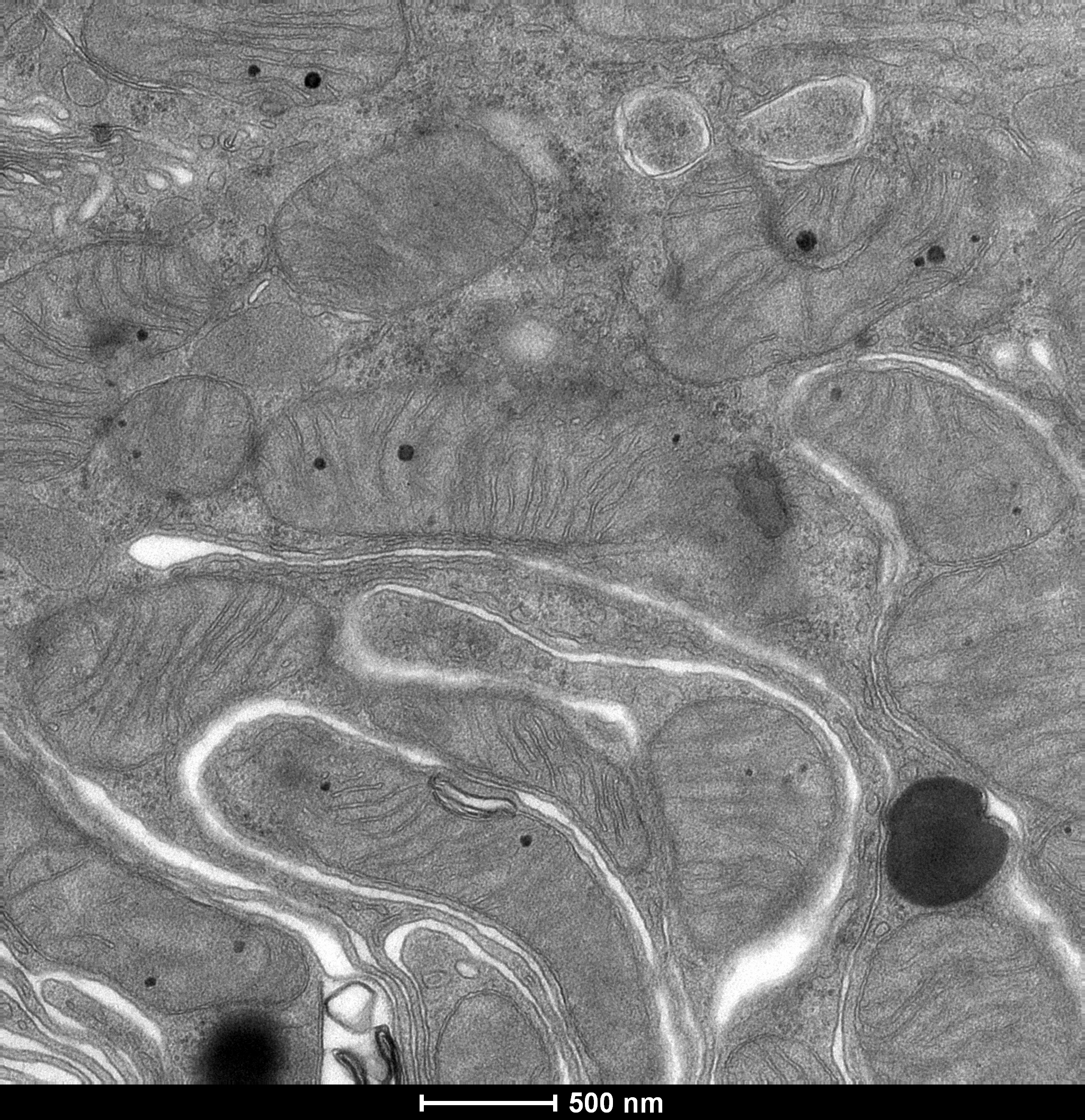

Supplement: Supplementary file 8 — Source Data for Figure 2 [file EMMM-15-e16581-s015.zip › Figure 2/2I/WT.tif]

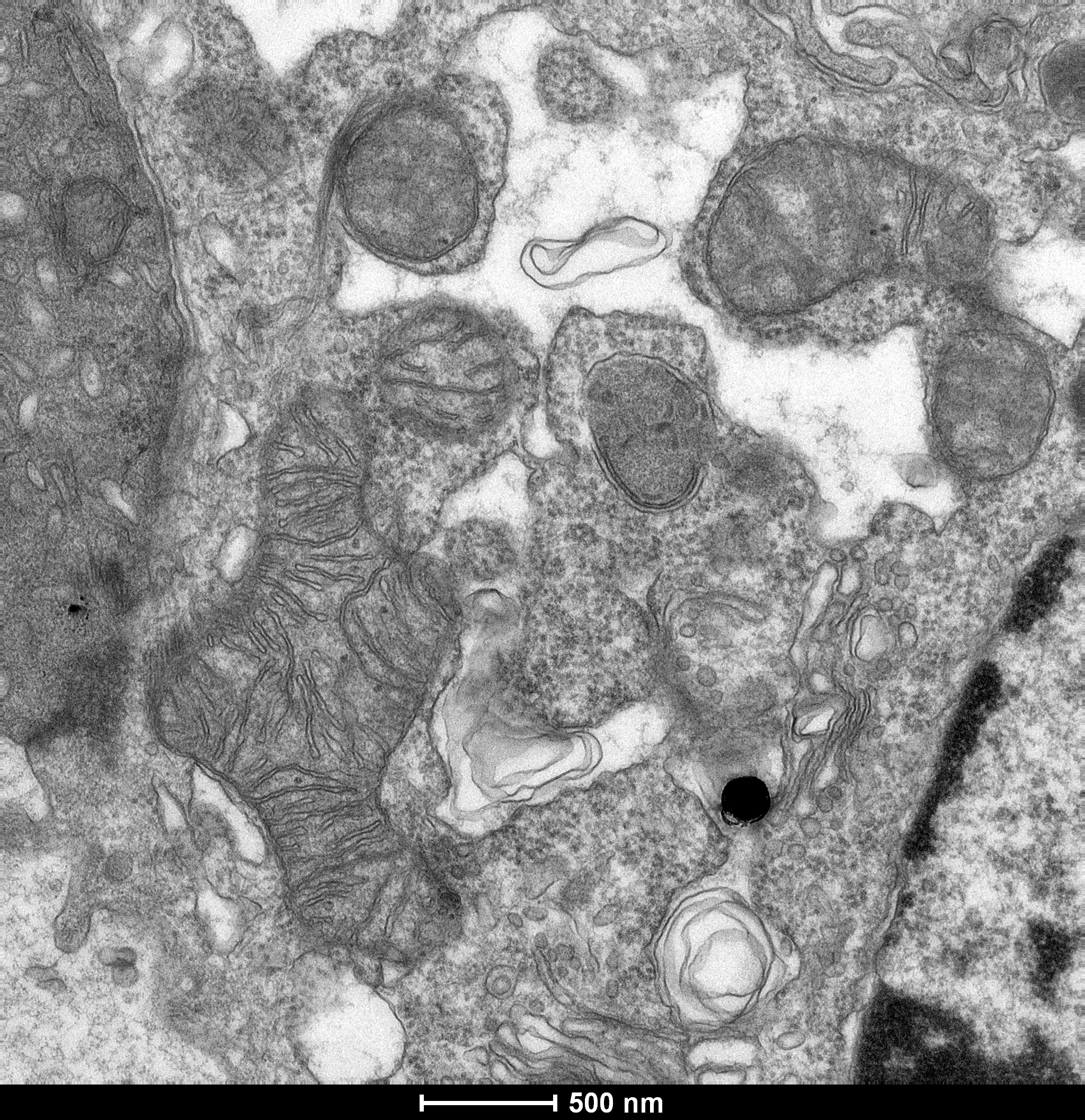

Supplement: Supplementary file 8 — Source Data for Figure 2 [file EMMM-15-e16581-s015.zip › Figure 2/2I/cKO+UUO.tif]

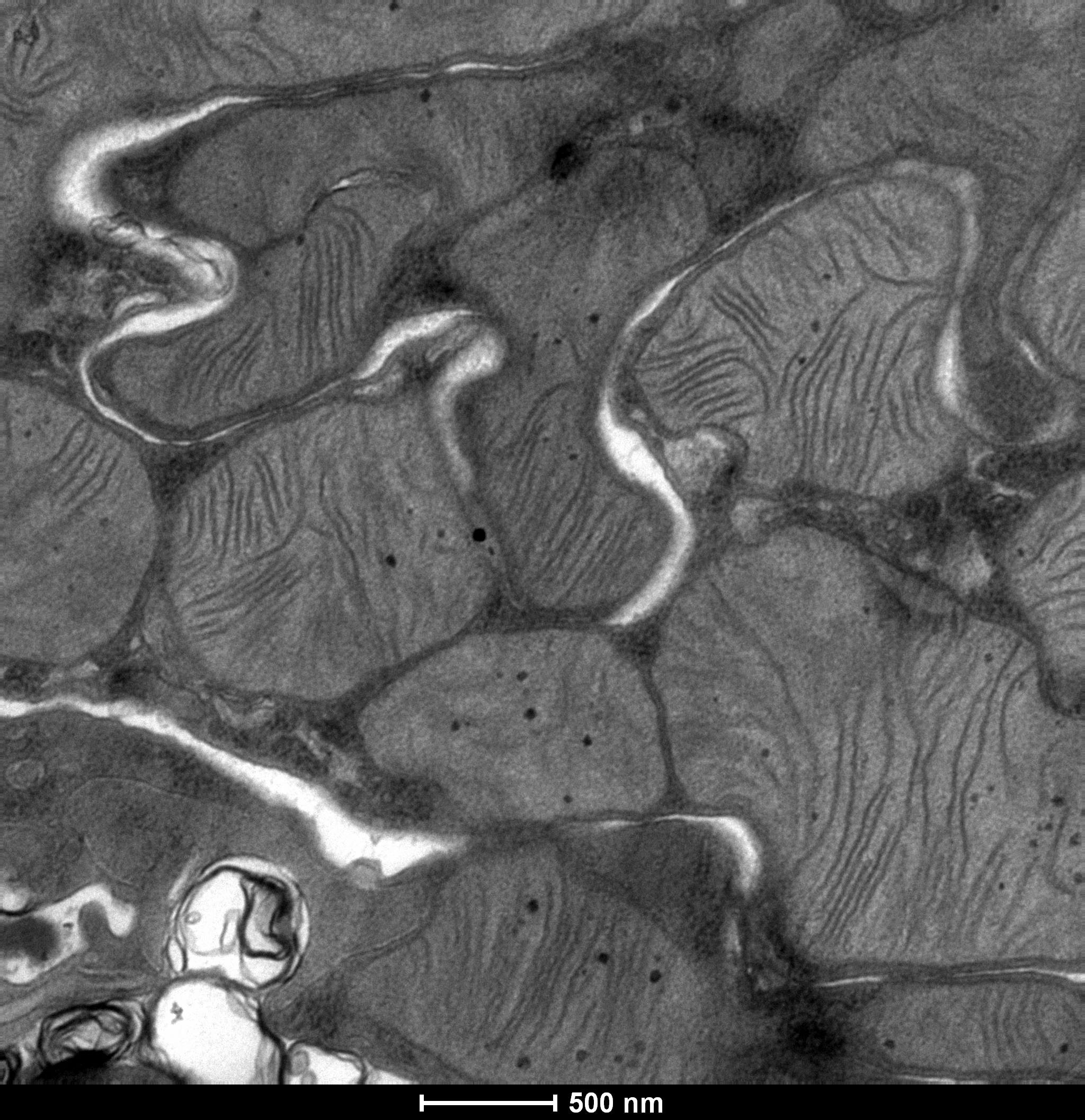

Supplement: Supplementary file 8 — Source Data for Figure 2 [file EMMM-15-e16581-s015.zip › Figure 2/2I/cKO.tif]

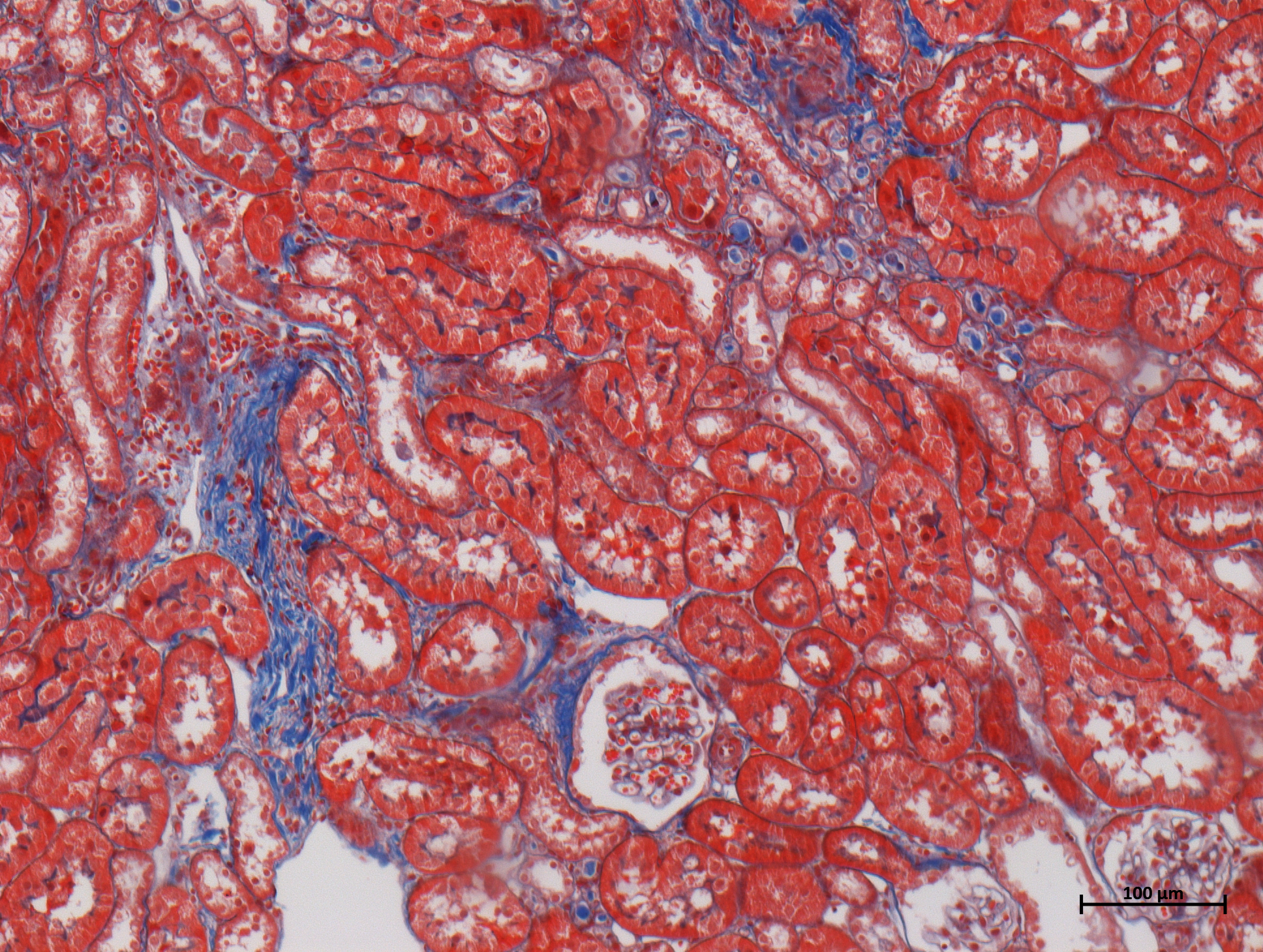

Supplement: Supplementary file 9 — Source Data for Figure 3 [file EMMM-15-e16581-s010.zip › Figure 3/3C/56Nx WT.tif]

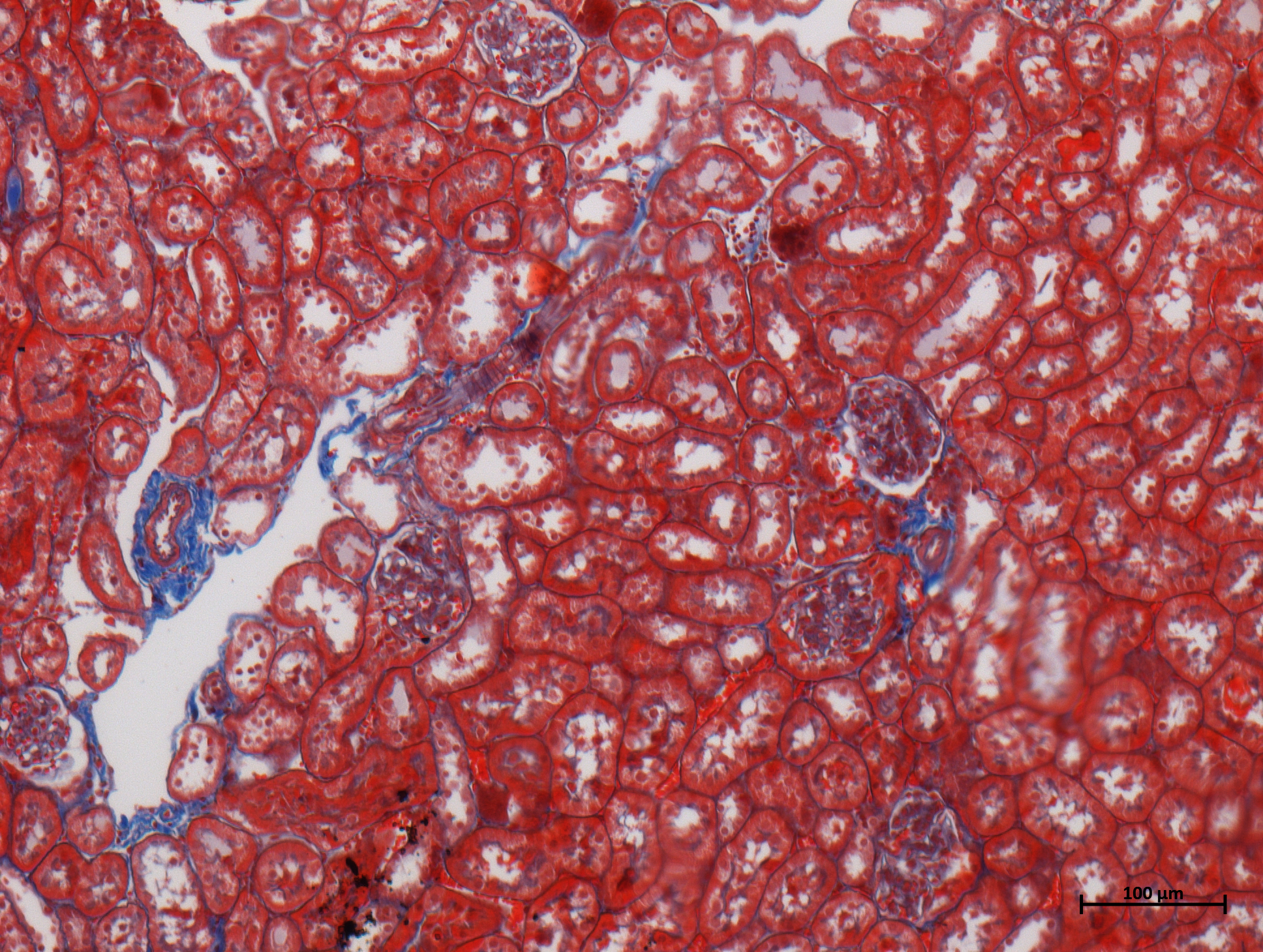

Supplement: Supplementary file 9 — Source Data for Figure 3 [file EMMM-15-e16581-s010.zip › Figure 3/3C/56Nx cKI.tif]

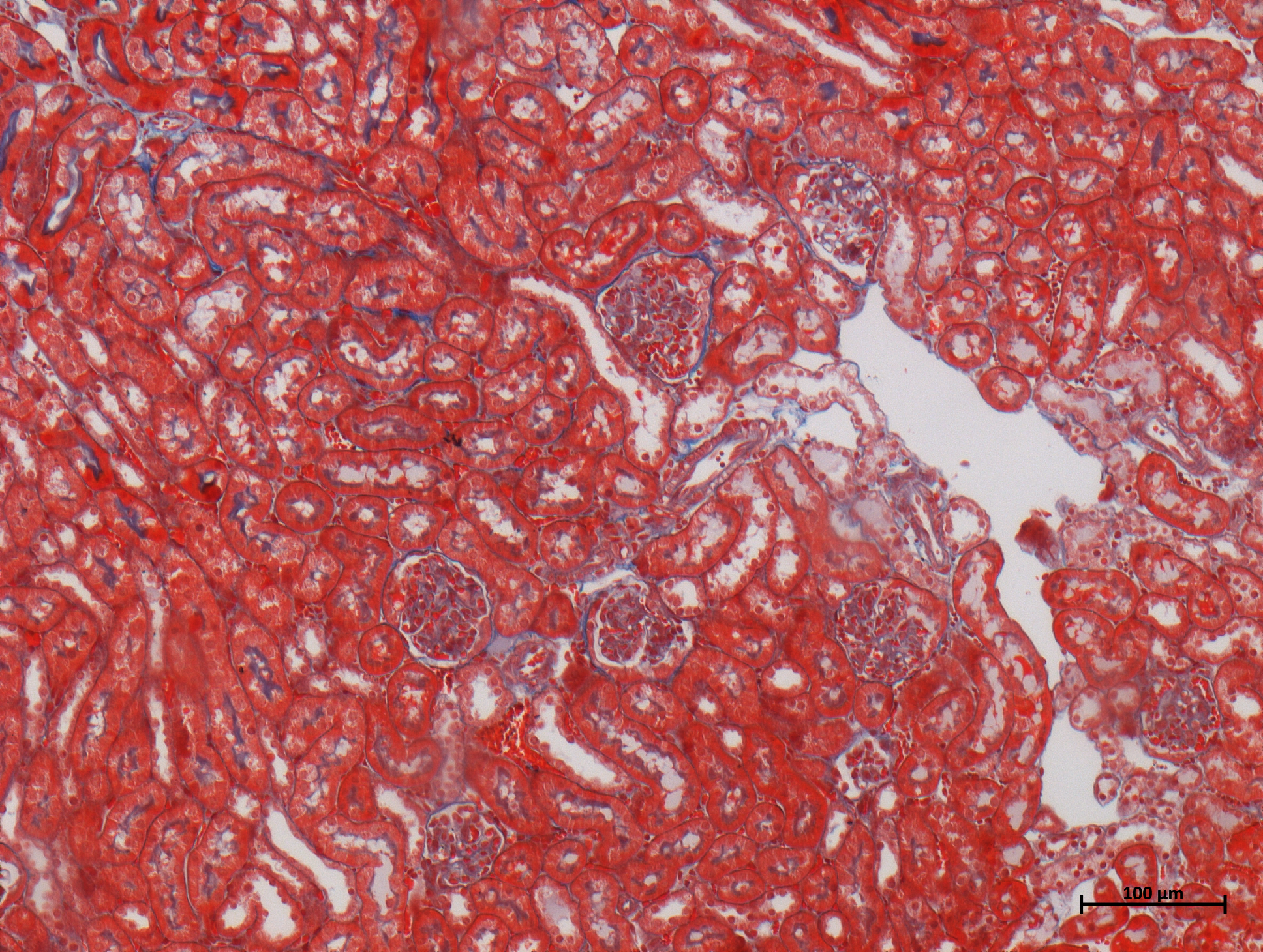

Supplement: Supplementary file 9 — Source Data for Figure 3 [file EMMM-15-e16581-s010.zip › Figure 3/3C/Sham WT.tif]

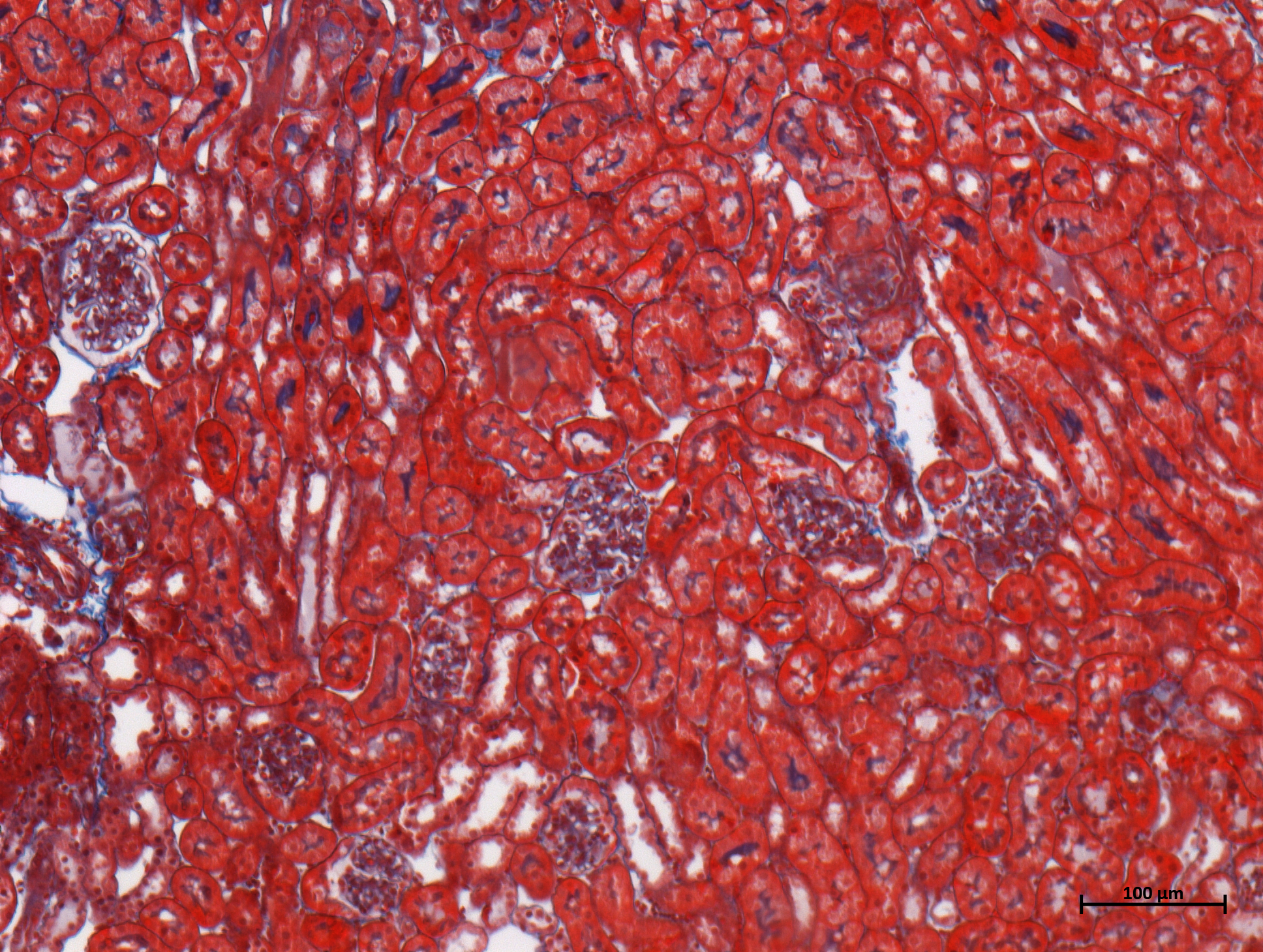

Supplement: Supplementary file 9 — Source Data for Figure 3 [file EMMM-15-e16581-s010.zip › Figure 3/3C/Sham cKI.tif]

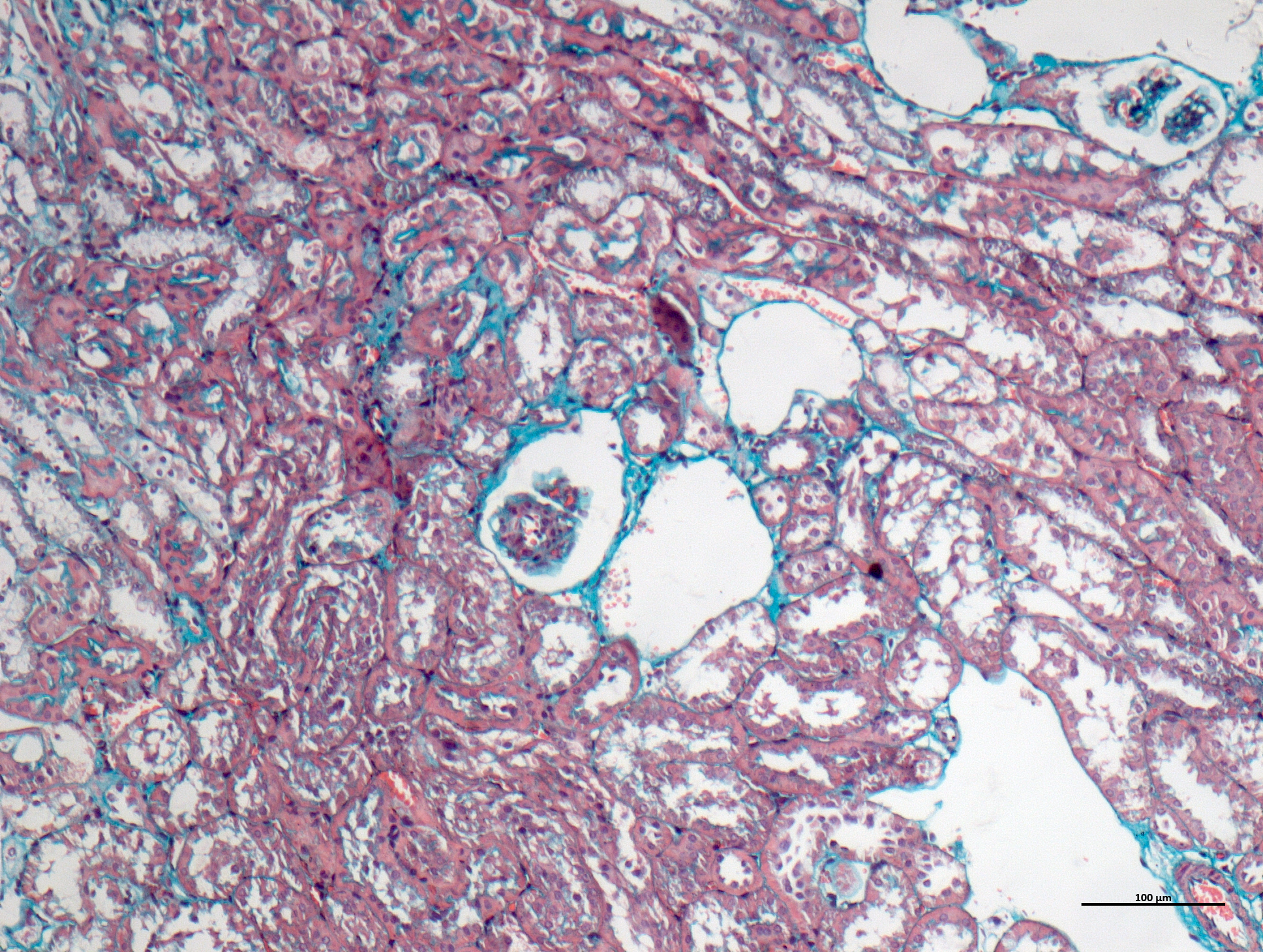

Supplement: Supplementary file 9 — Source Data for Figure 3 [file EMMM-15-e16581-s010.zip › Figure 3/3G/56Nx WT.tif]

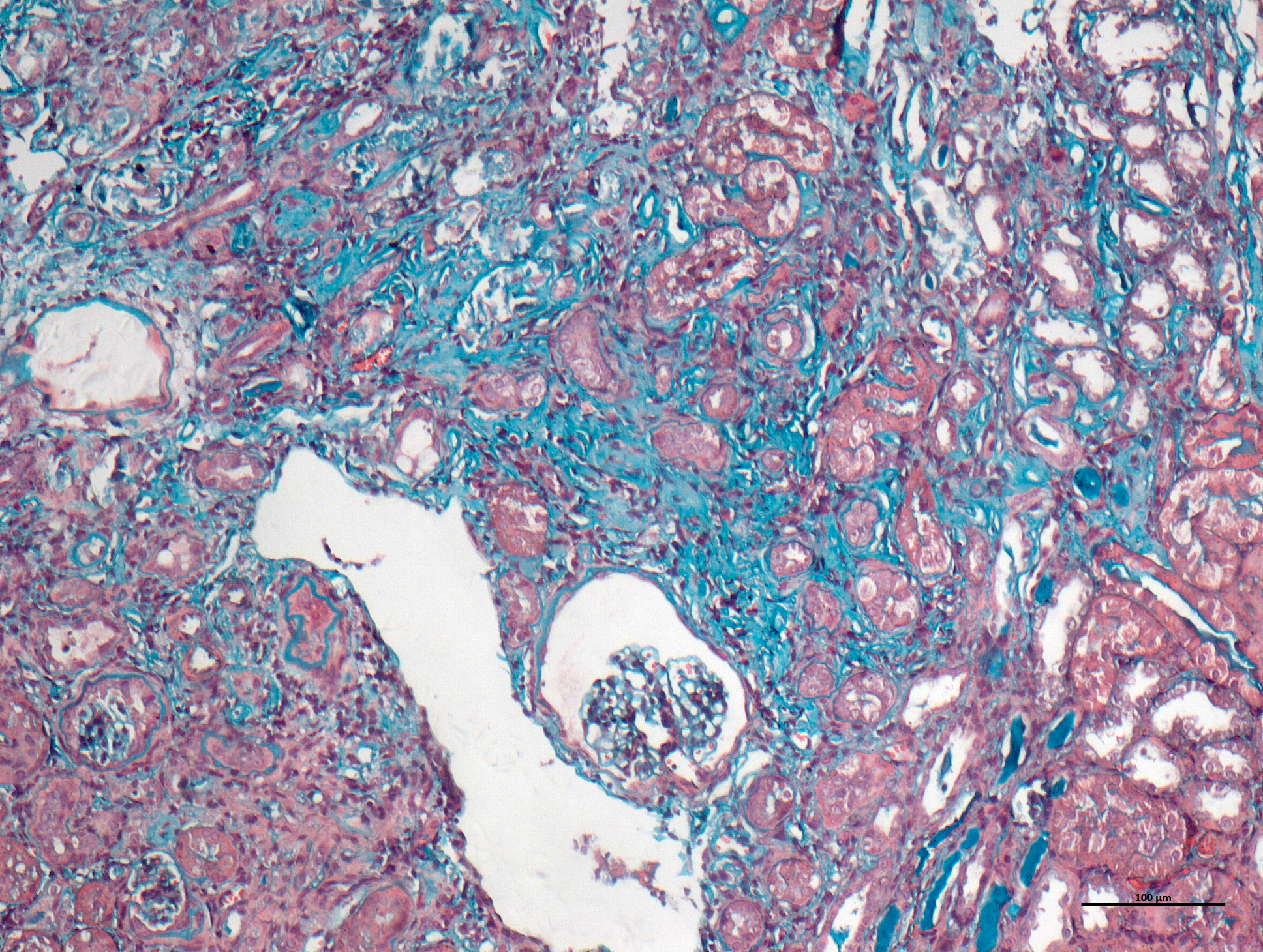

Supplement: Supplementary file 9 — Source Data for Figure 3 [file EMMM-15-e16581-s010.zip › Figure 3/3G/56Nx cKO.tif]

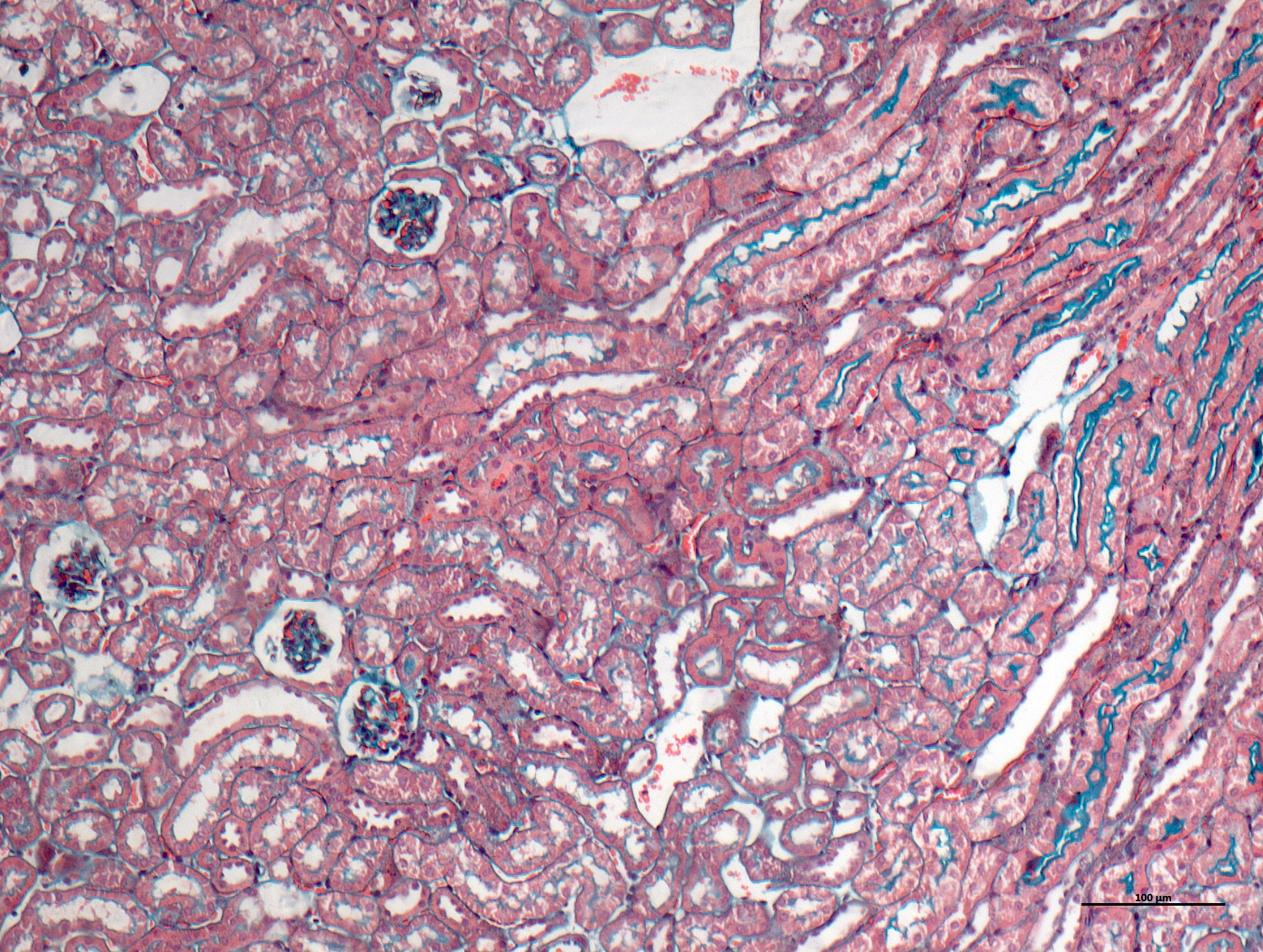

Supplement: Supplementary file 9 — Source Data for Figure 3 [file EMMM-15-e16581-s010.zip › Figure 3/3G/Sham WT.tif]

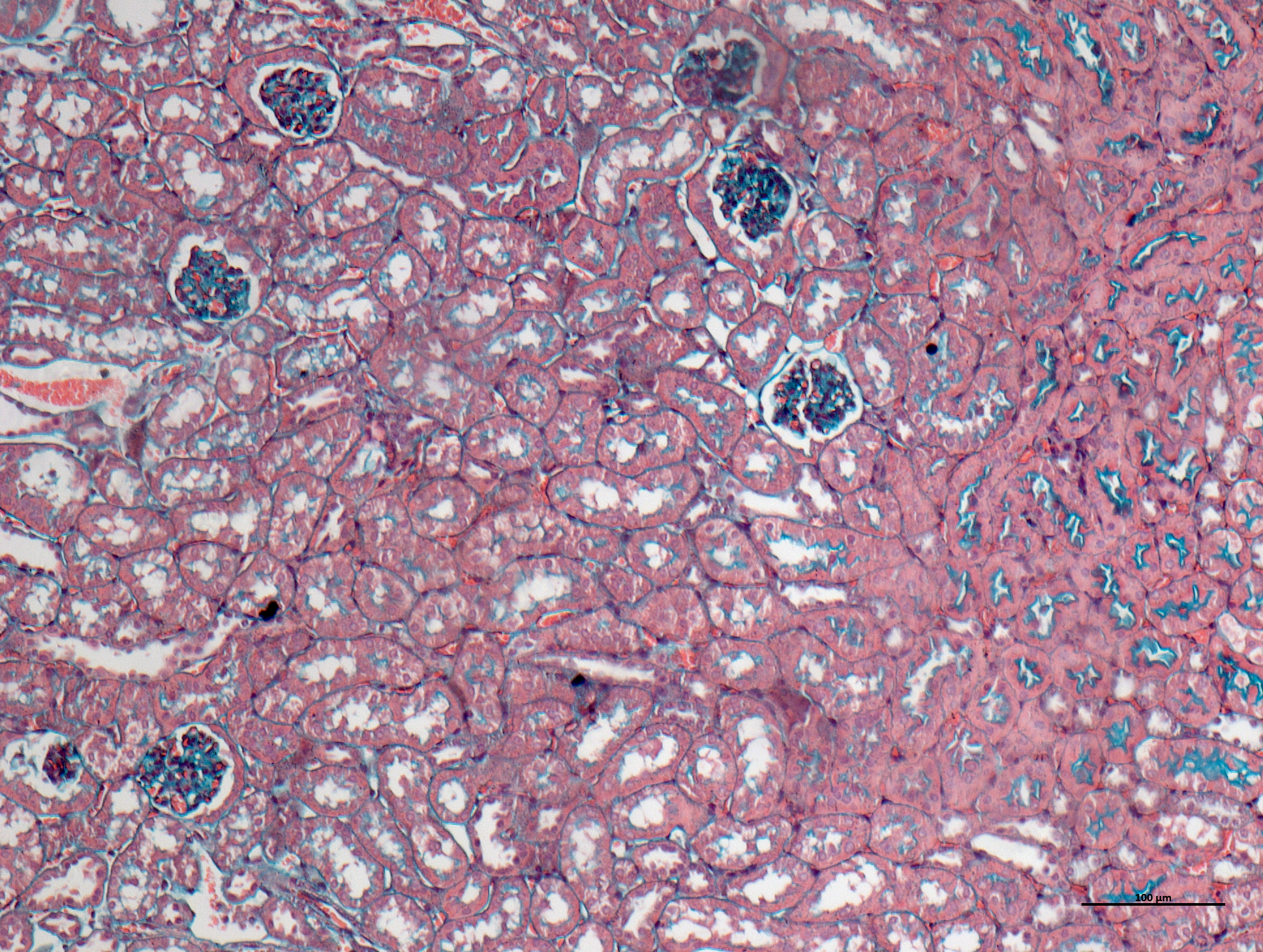

Supplement: Supplementary file 9 — Source Data for Figure 3 [file EMMM-15-e16581-s010.zip › Figure 3/3G/Sham cKO.tif]

Fig 3I

Collagen I

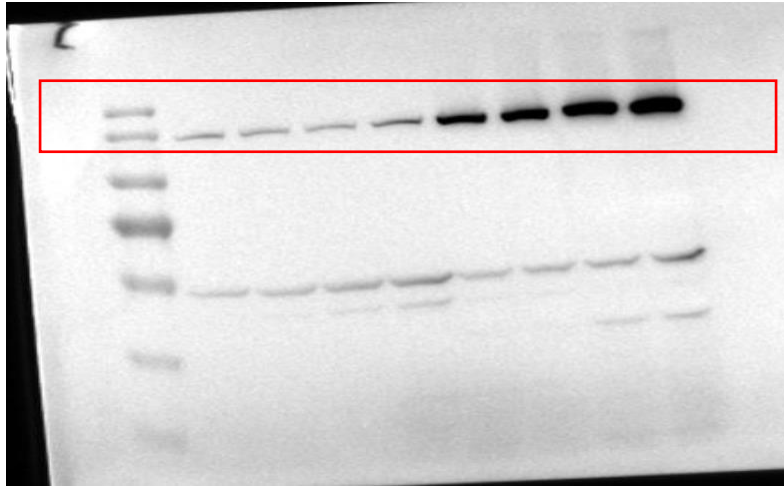

GAPDH

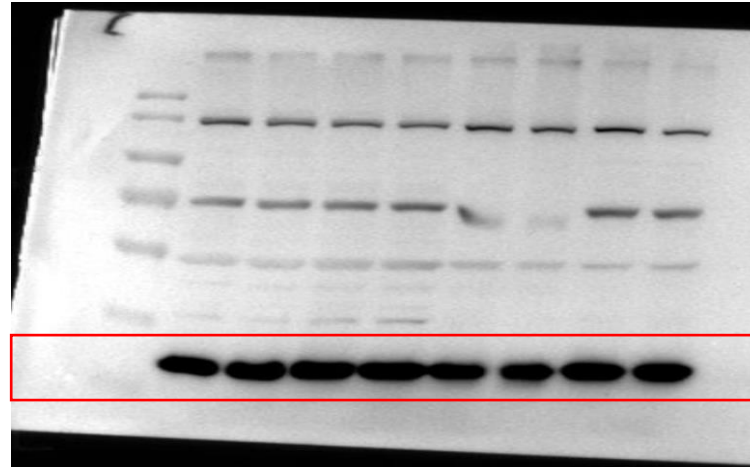

$\alpha$ -SMA

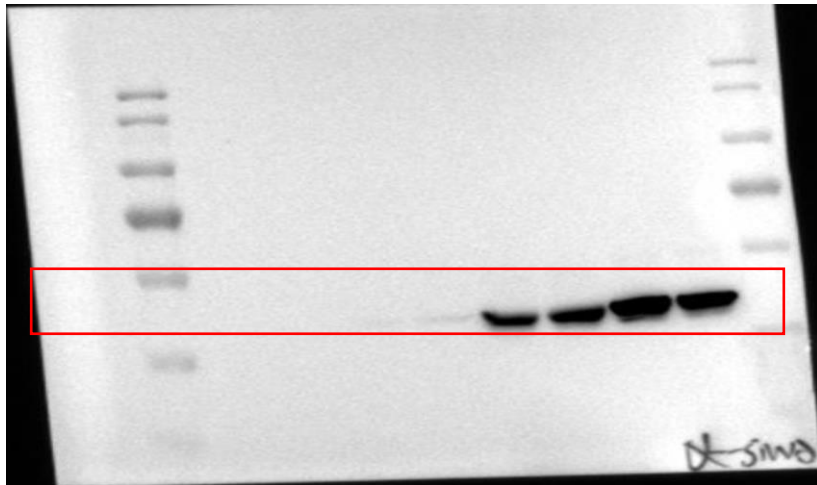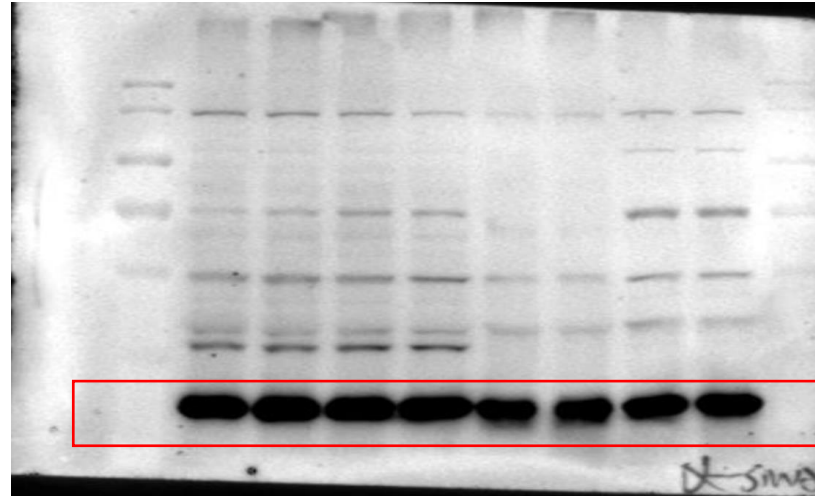

Supplement: Supplementary file 9 — Source Data for Figure 3 [file EMMM-15-e16581-s010.zip › Figure 3/3I-J/western gel.pdf]

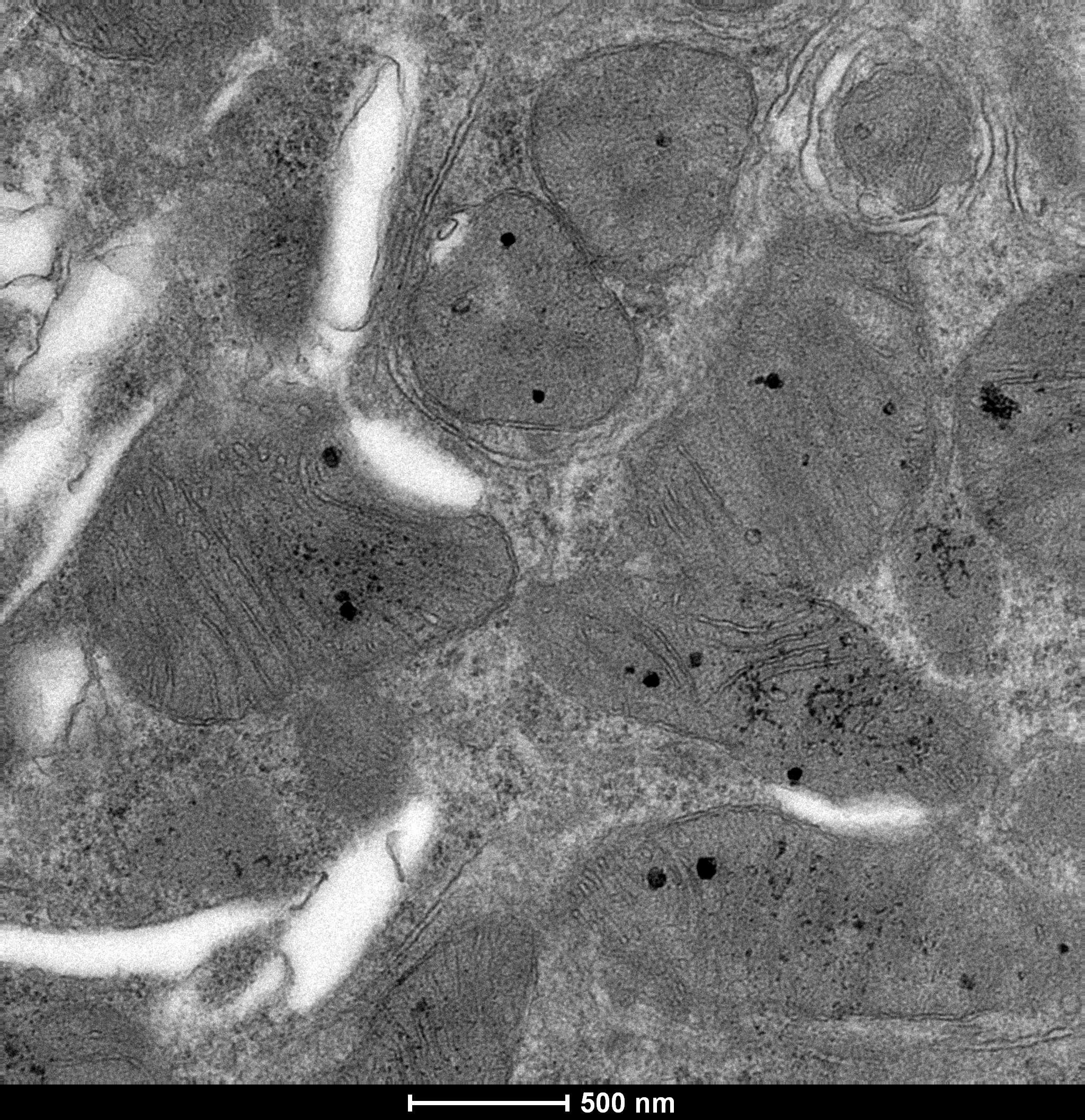

Supplement: Supplementary file 9 — Source Data for Figure 3 [file EMMM-15-e16581-s010.zip › Figure 3/3L/WT+56Nx.tif]

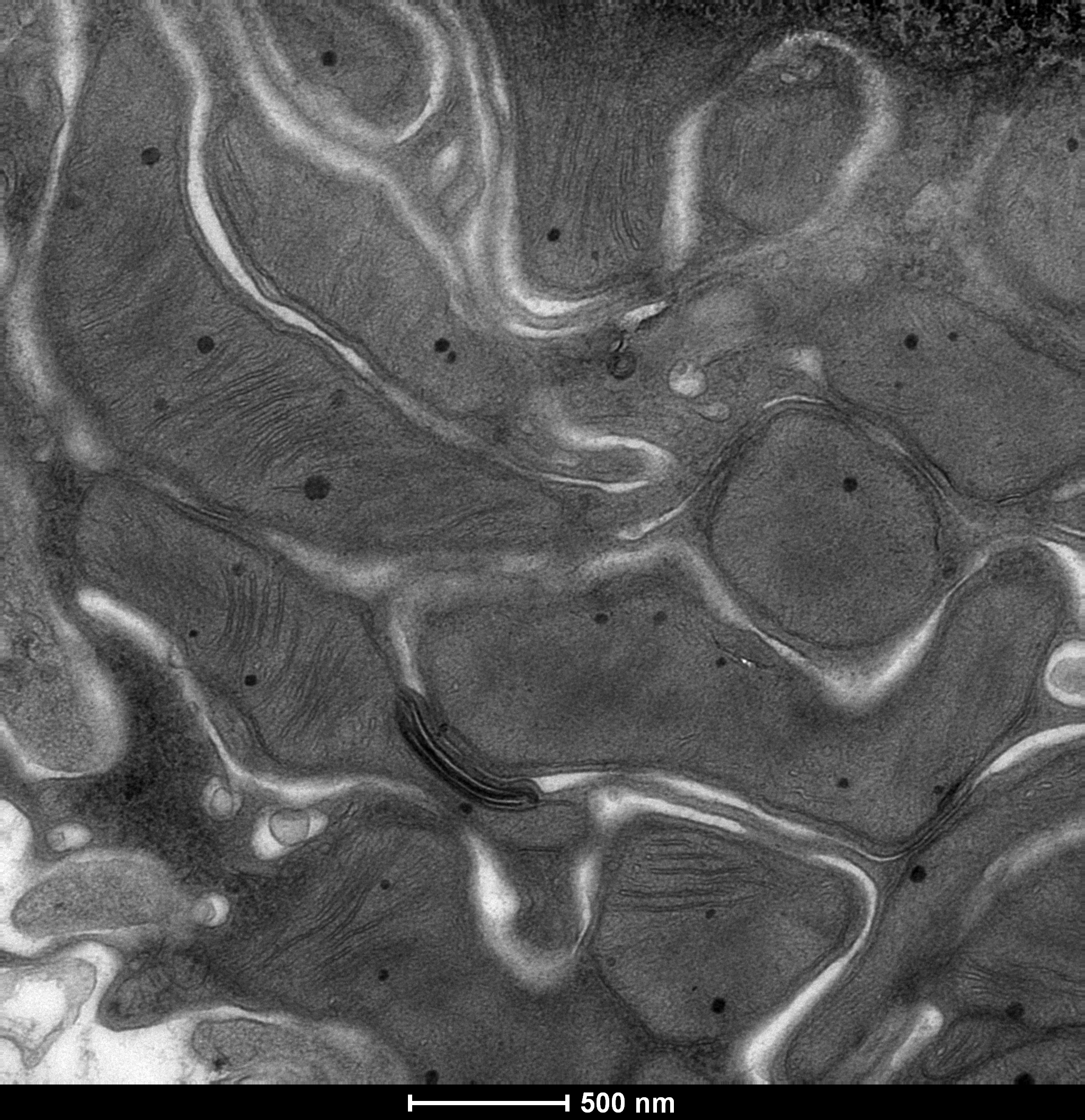

Supplement: Supplementary file 9 — Source Data for Figure 3 [file EMMM-15-e16581-s010.zip › Figure 3/3L/WT+Sham.tif]

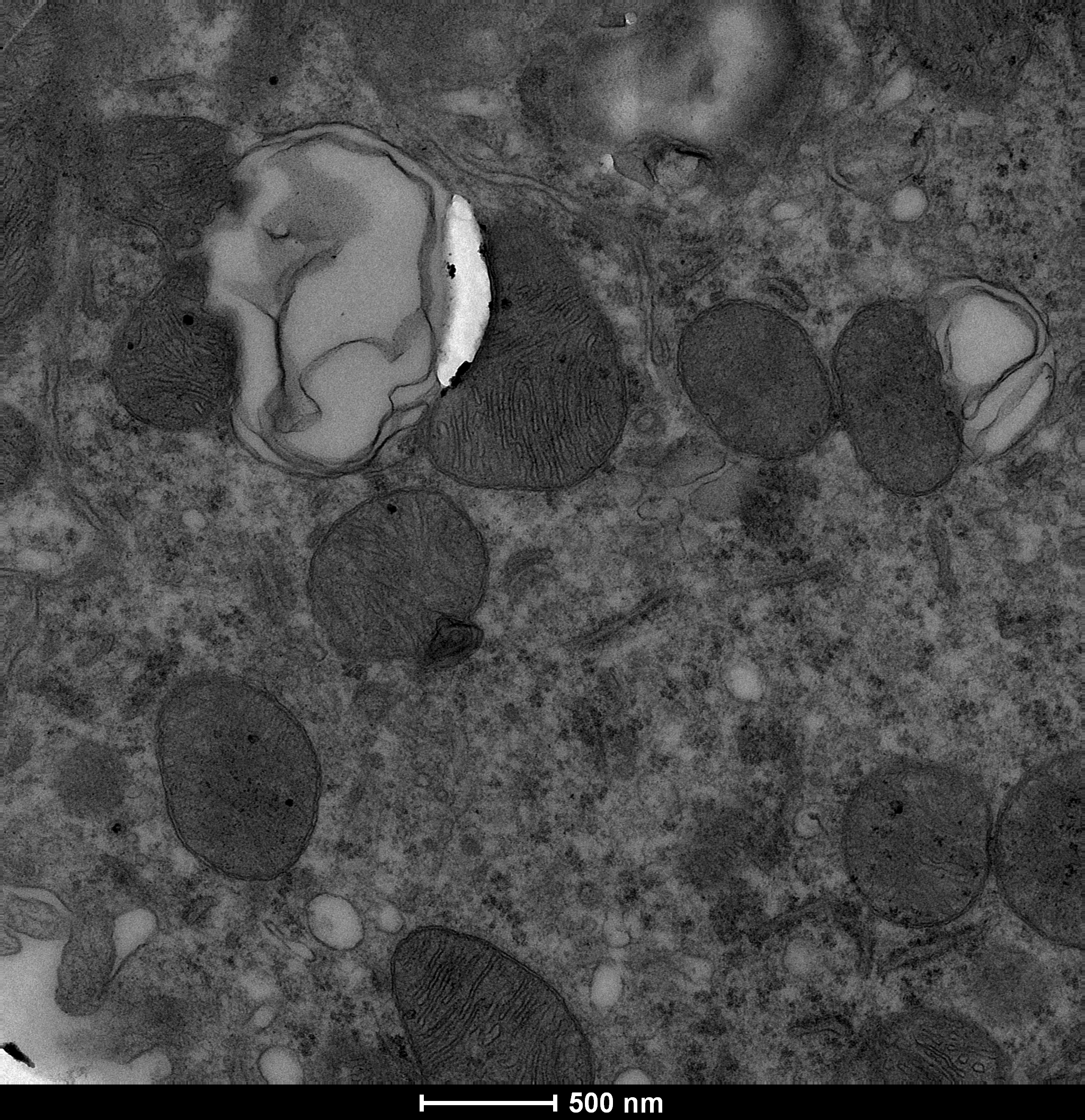

Supplement: Supplementary file 9 — Source Data for Figure 3 [file EMMM-15-e16581-s010.zip › Figure 3/3L/cKO+56Nx.tif]

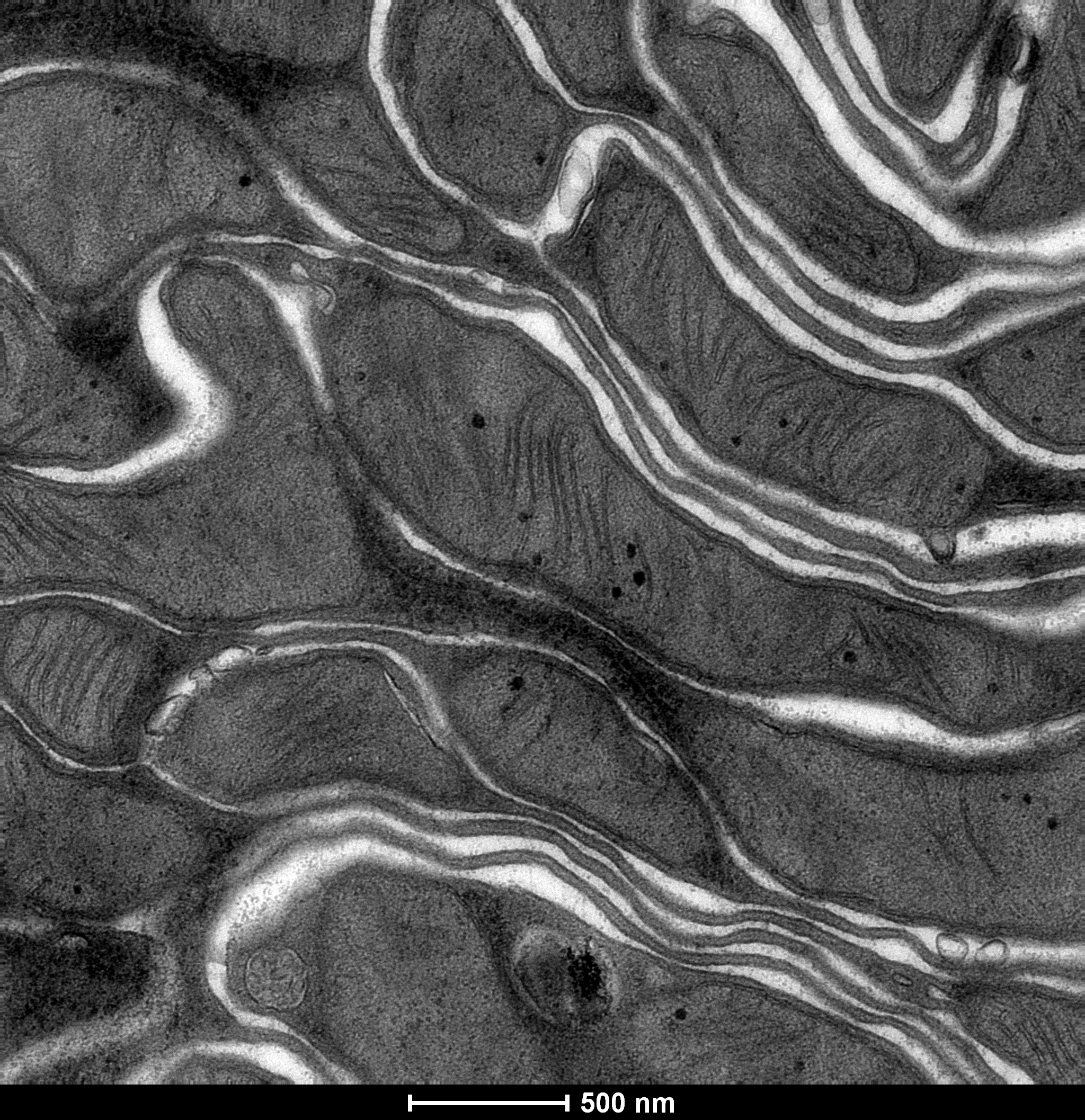

Supplement: Supplementary file 9 — Source Data for Figure 3 [file EMMM-15-e16581-s010.zip › Figure 3/3L/cKO+Sham.tif]

Fig 4E

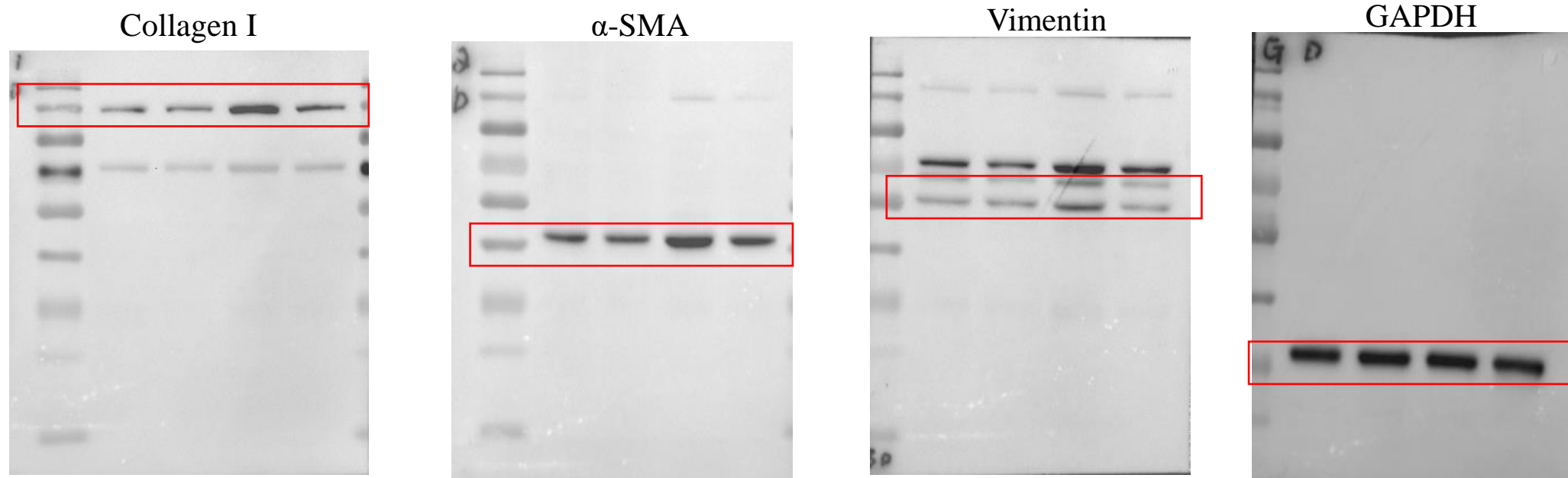

Supplement: Supplementary file 10 — Source Data for Figure 4 [file EMMM-15-e16581-s007.zip › Figure 4/4E-F/western gel.pdf]

Fig 4K

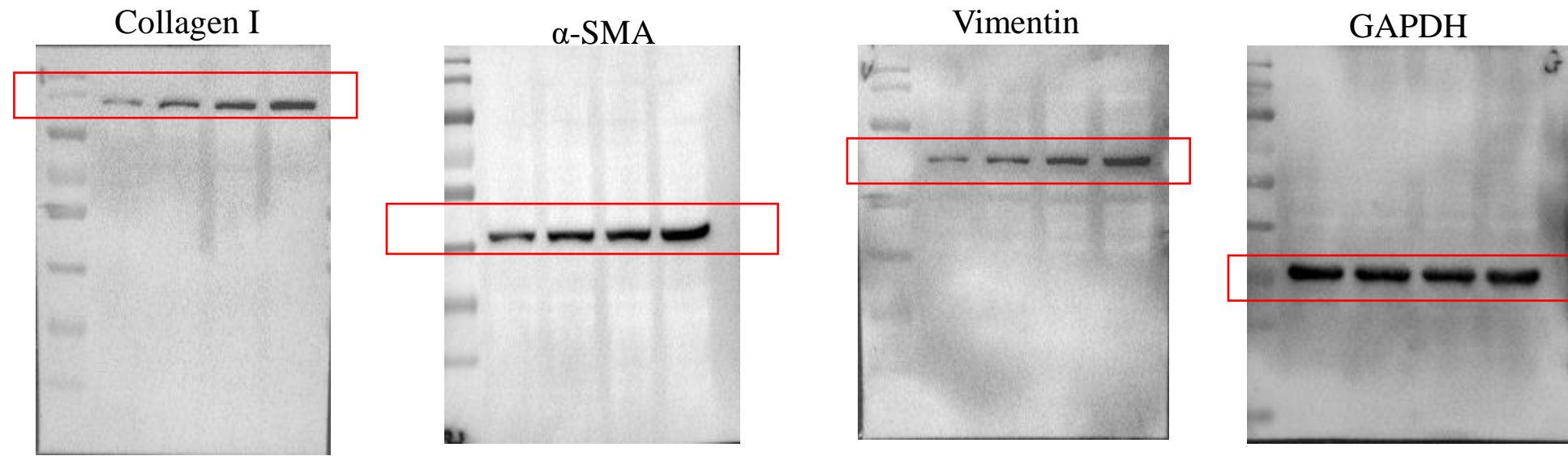

Supplement: Supplementary file 10 — Source Data for Figure 4 [file EMMM-15-e16581-s007.zip › Figure 4/4K-L/western gel.pdf]

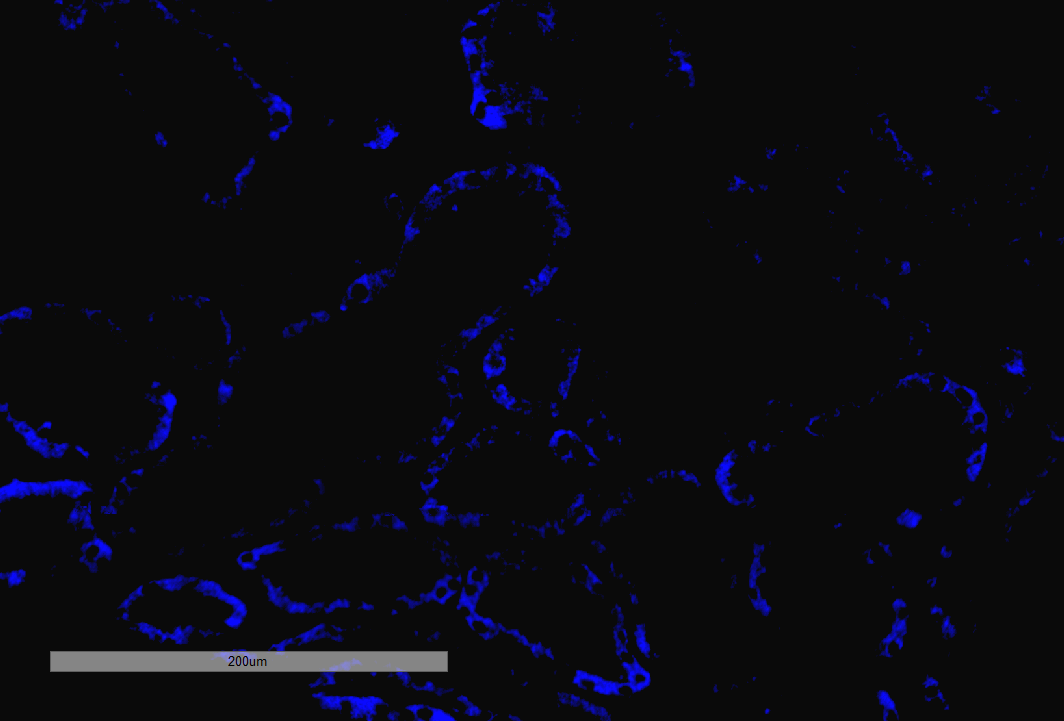

Supplement: Supplementary file 11 — Source Data for Figure 5 [file EMMM-15-e16581-s013.zip › Figure 5/5G/Human Kidney/AIF.tif]

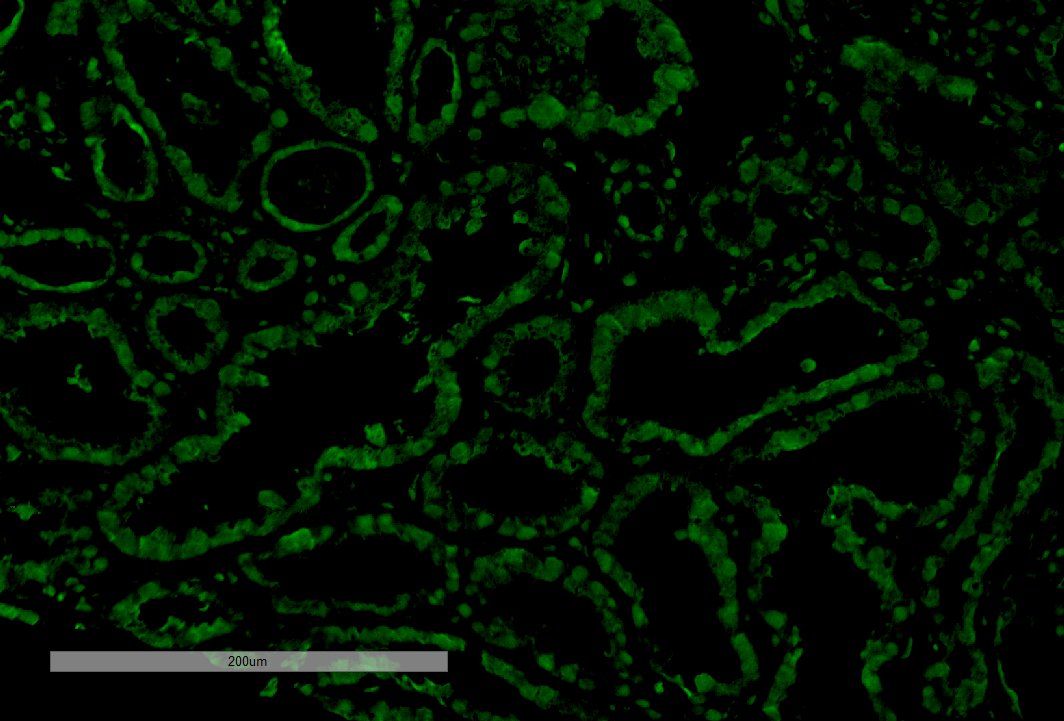

Supplement: Supplementary file 11 — Source Data for Figure 5 [file EMMM-15-e16581-s013.zip › Figure 5/5G/Human Kidney/HMGCS2.tif]

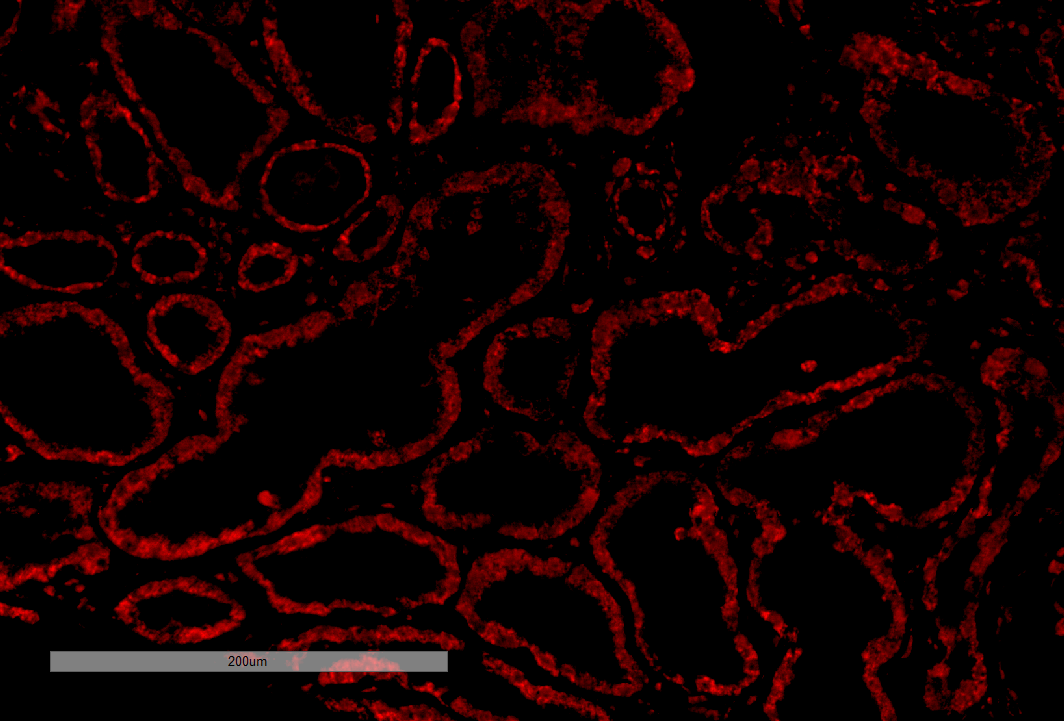

Supplement: Supplementary file 11 — Source Data for Figure 5 [file EMMM-15-e16581-s013.zip › Figure 5/5G/Human Kidney/LONP1.tif]

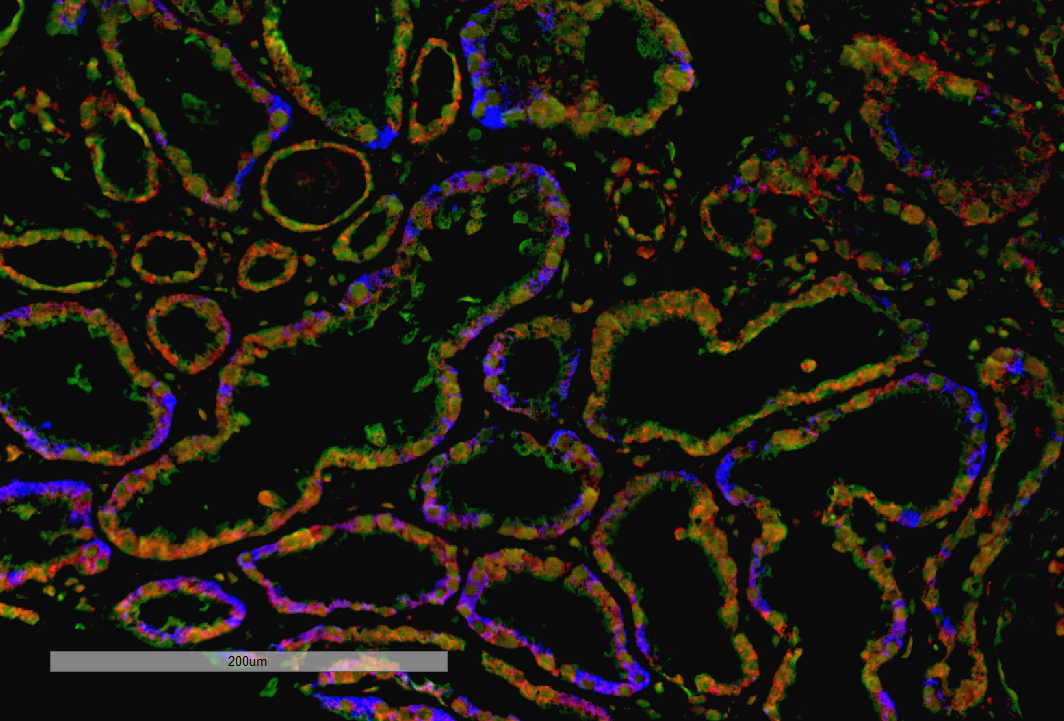

Supplement: Supplementary file 11 — Source Data for Figure 5 [file EMMM-15-e16581-s013.zip › Figure 5/5G/Human Kidney/Merge.tif]

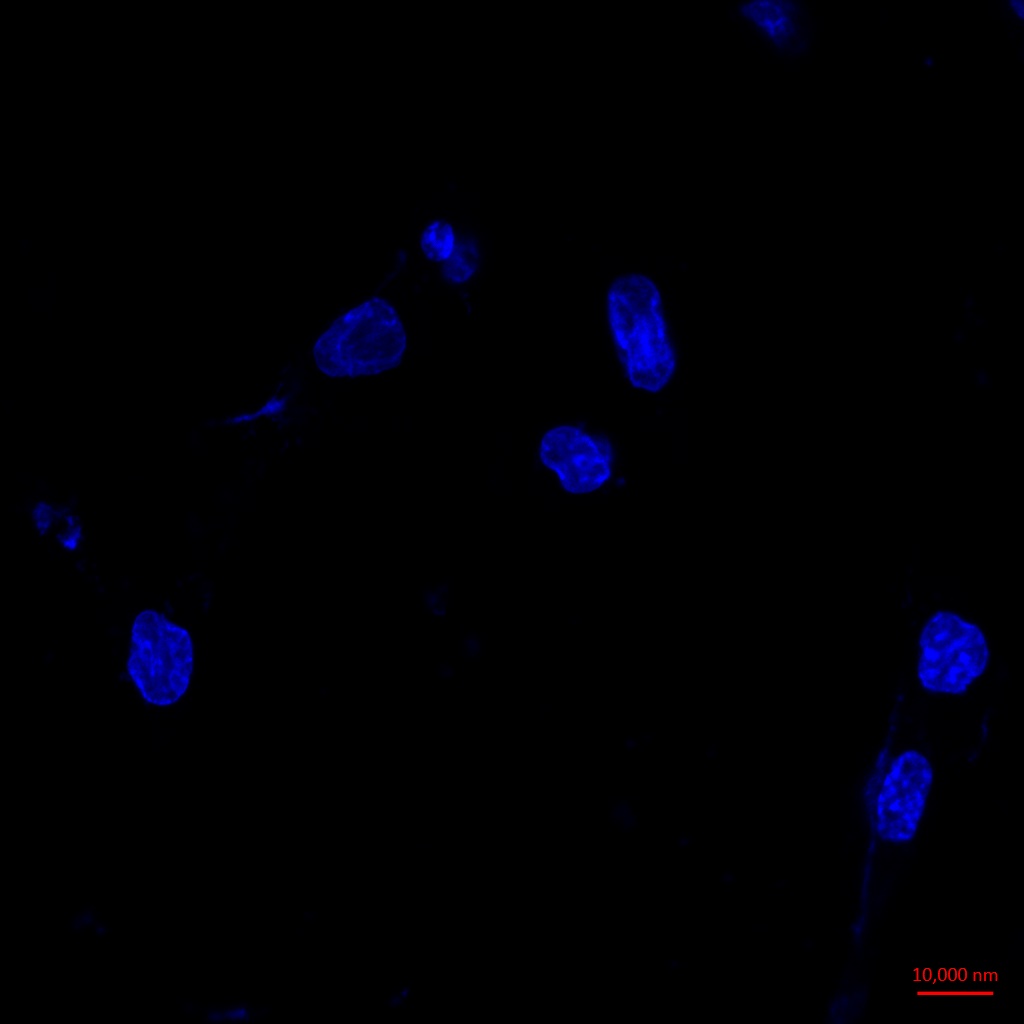

Supplement: Supplementary file 11 — Source Data for Figure 5 [file EMMM-15-e16581-s013.zip › Figure 5/5G/mPTC/DAPI.jpg]

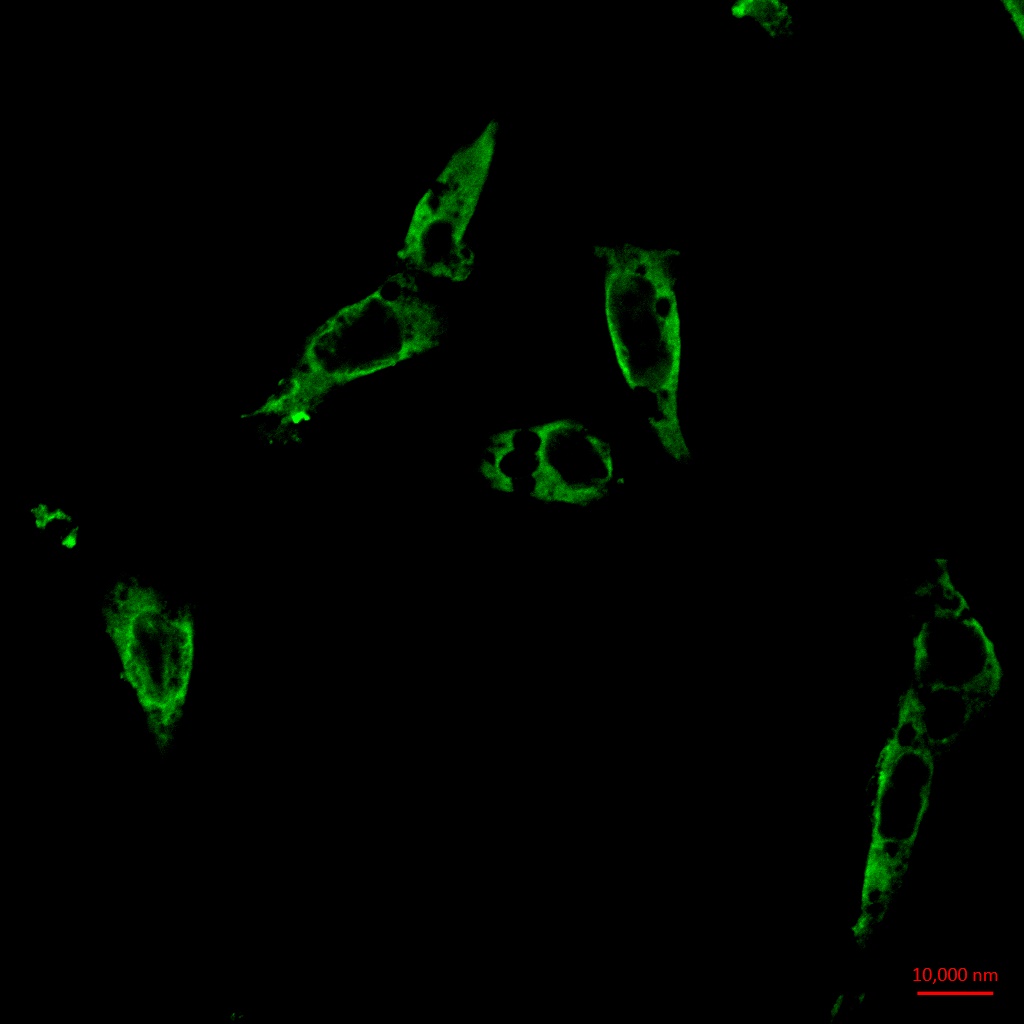

Supplement: Supplementary file 11 — Source Data for Figure 5 [file EMMM-15-e16581-s013.zip › Figure 5/5G/mPTC/HMGCS2.jpg]

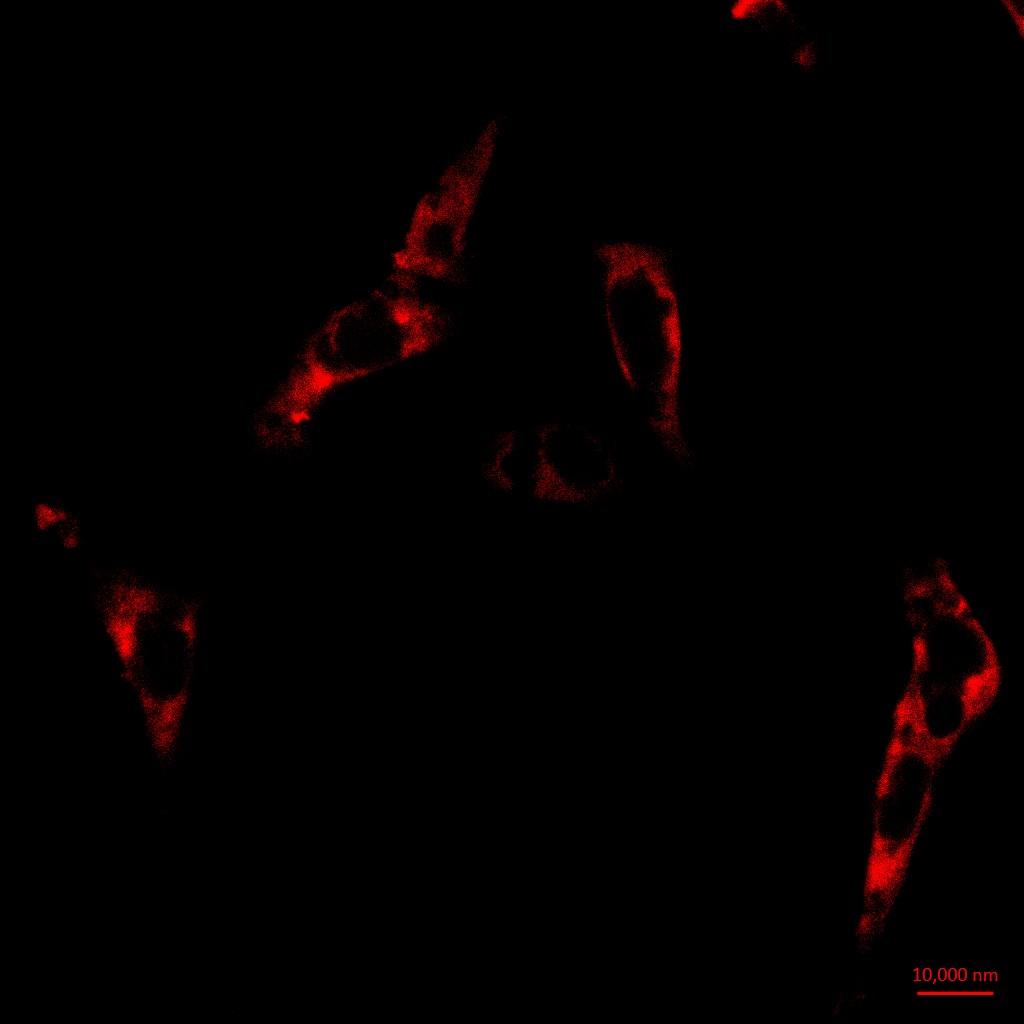

Supplement: Supplementary file 11 — Source Data for Figure 5 [file EMMM-15-e16581-s013.zip › Figure 5/5G/mPTC/LONP1-Flag.jpg]

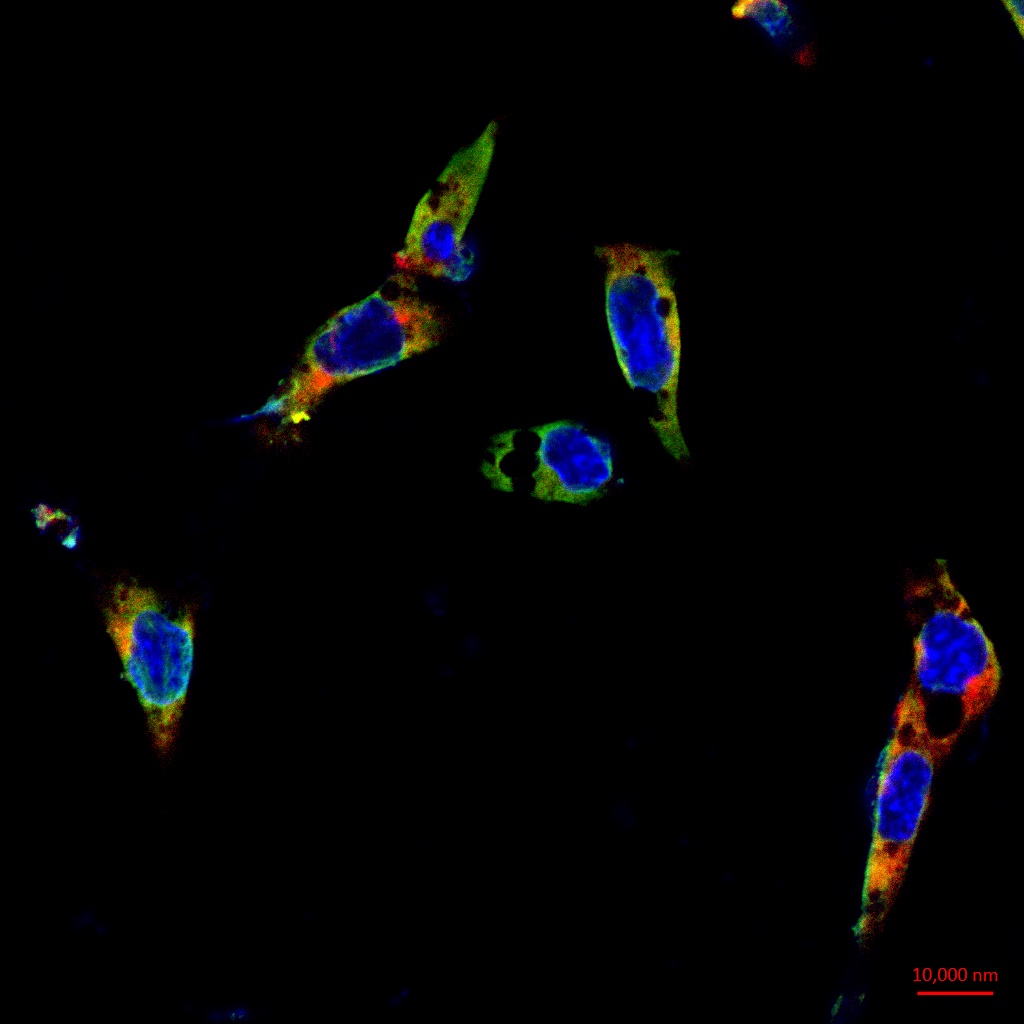

Supplement: Supplementary file 11 — Source Data for Figure 5 [file EMMM-15-e16581-s013.zip › Figure 5/5G/mPTC/Merge.jpg]

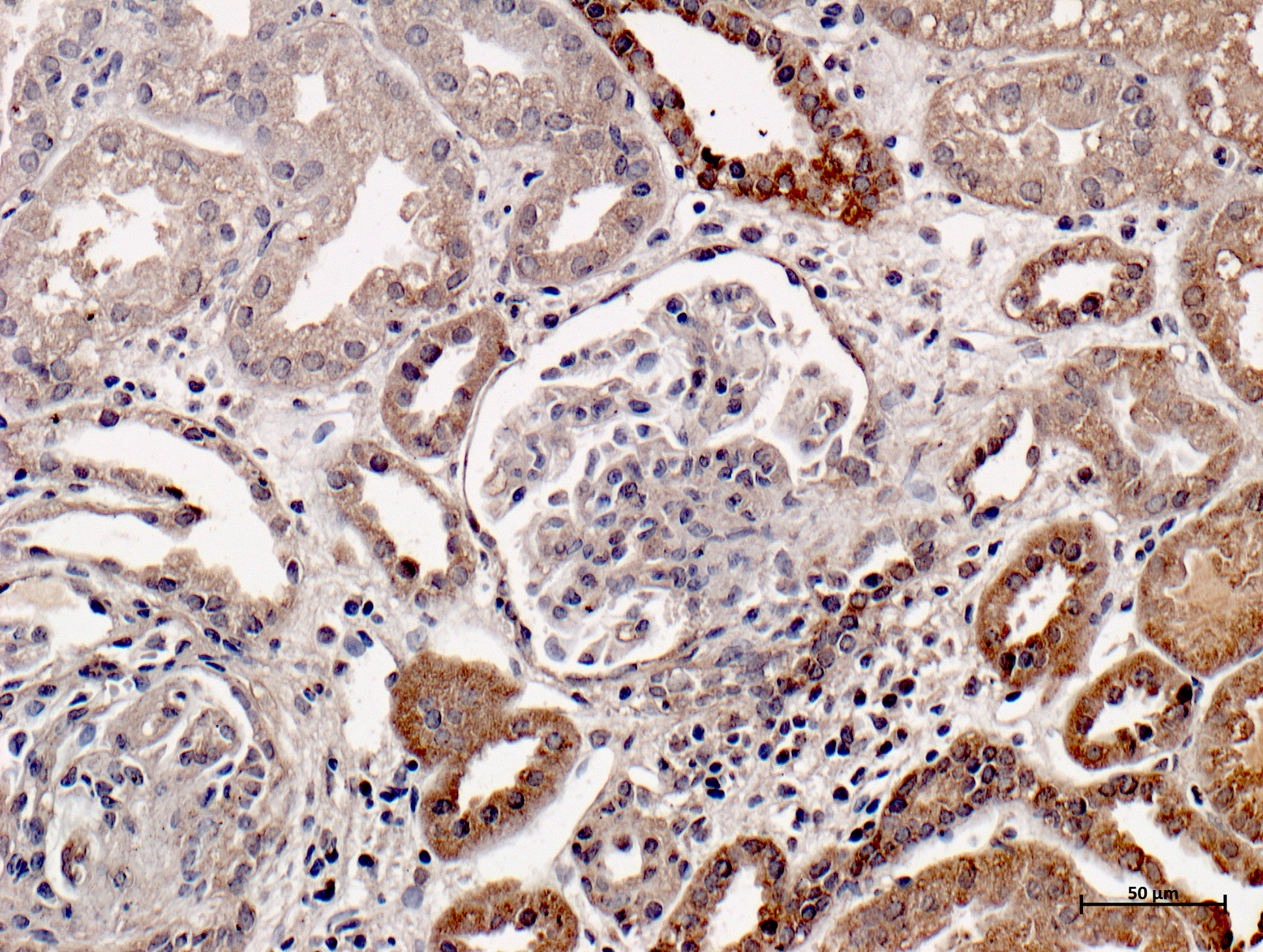

Supplement: Supplementary file 12 — Source Data for Figure 6 [file EMMM-15-e16581-s005.zip › Figure 6/6A-C/CKD.tif]

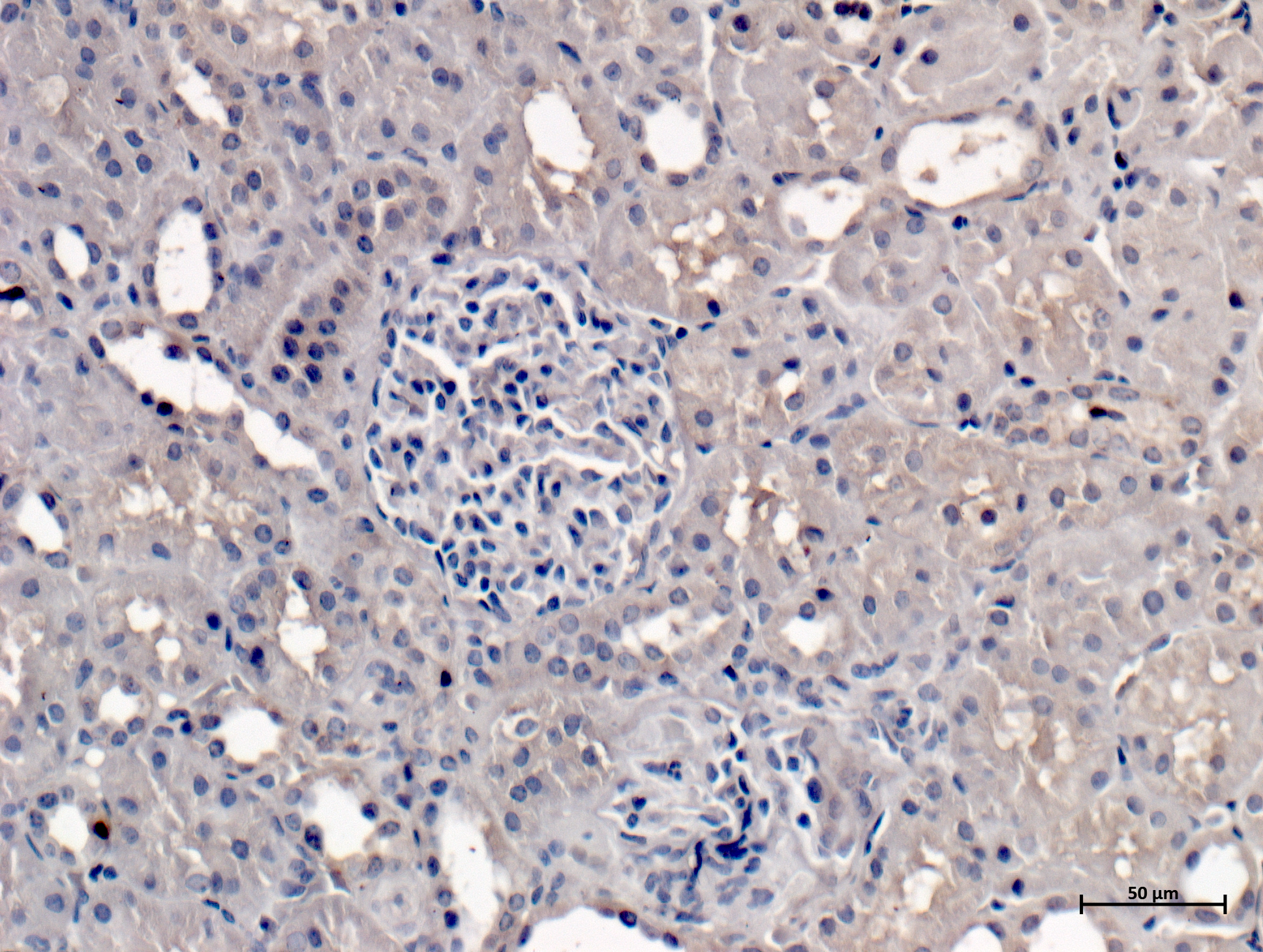

Supplement: Supplementary file 12 — Source Data for Figure 6 [file EMMM-15-e16581-s005.zip › Figure 6/6A-C/Control.tif]

Fig 6D

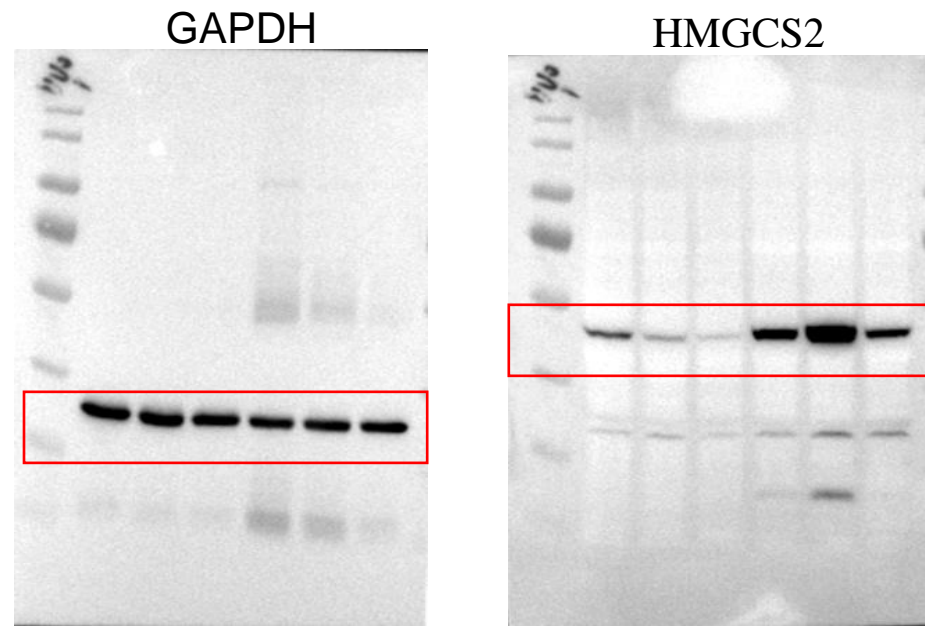

Fig 6F

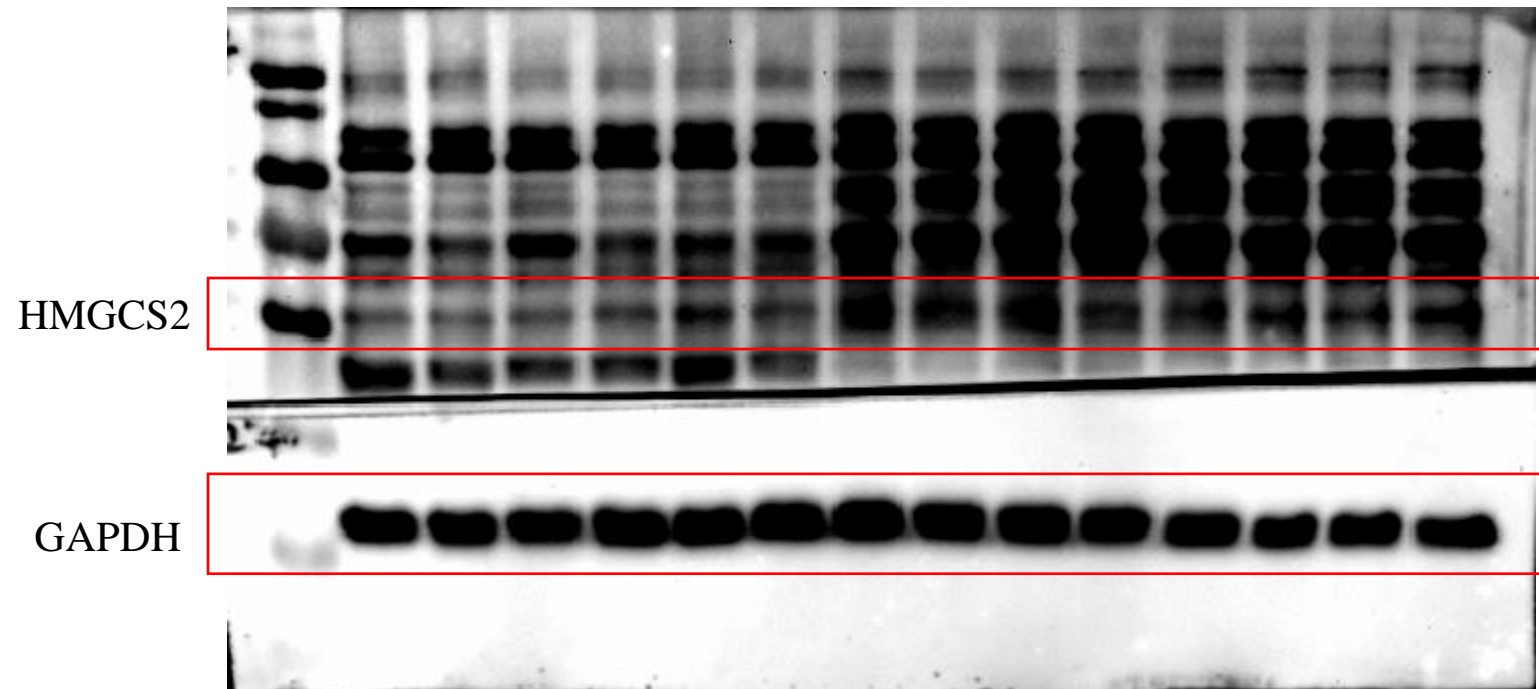

Supplement: Supplementary file 12 — Source Data for Figure 6 [file EMMM-15-e16581-s005.zip › Figure 6/6D-G/western gel.pdf]

Fig 6I

GAPDH

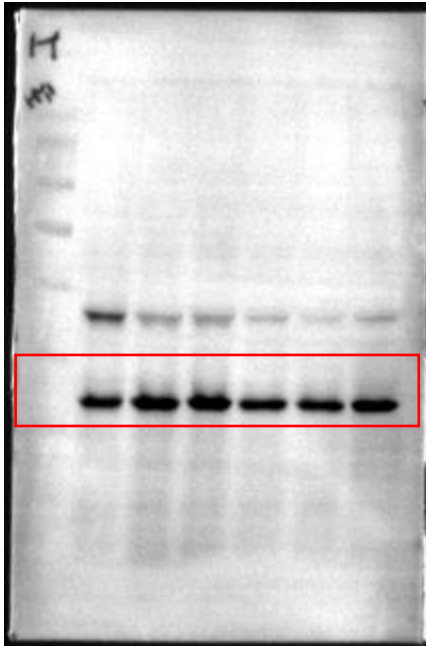

HMGCS2

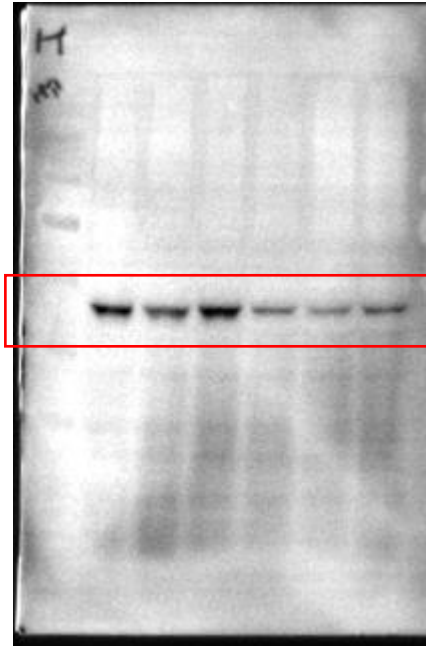

Supplement: Supplementary file 12 — Source Data for Figure 6 [file EMMM-15-e16581-s005.zip › Figure 6/6I/western gel.pdf]

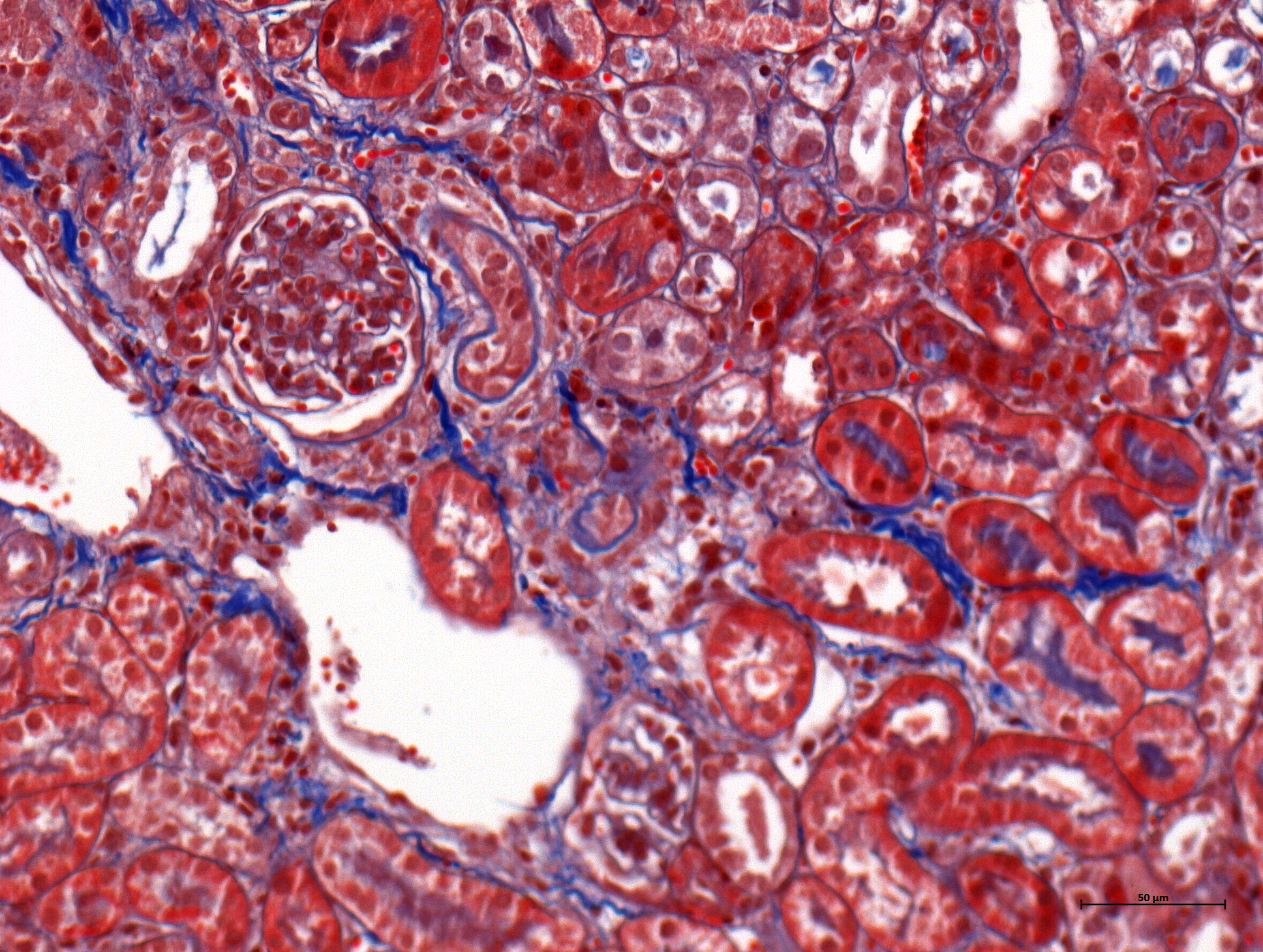

Supplement: Supplementary file 12 — Source Data for Figure 6 [file EMMM-15-e16581-s005.zip › Figure 6/6K/Hmgcs2+UUO.tif]

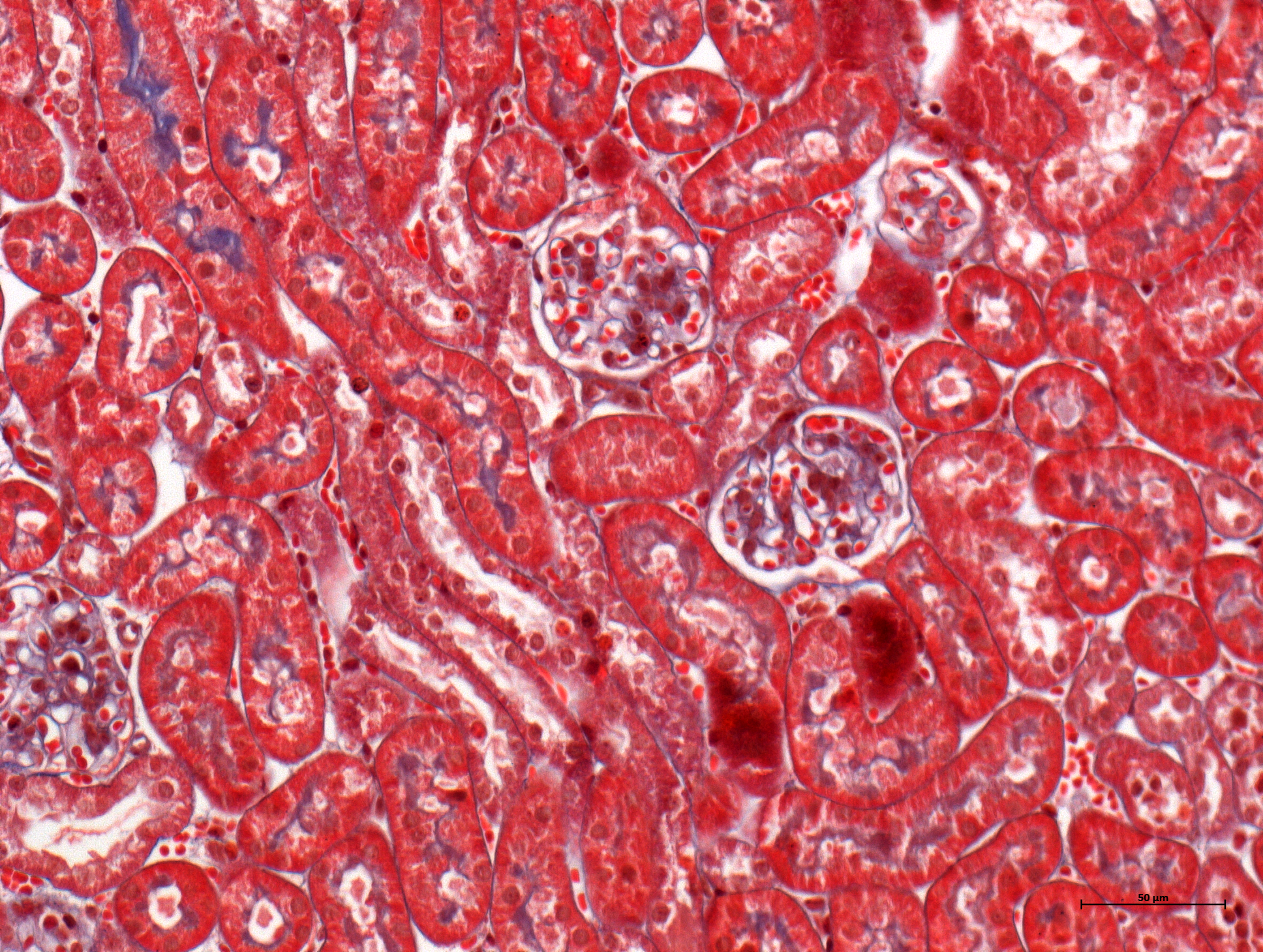

Supplement: Supplementary file 12 — Source Data for Figure 6 [file EMMM-15-e16581-s005.zip › Figure 6/6K/Hmgcs2.tif]

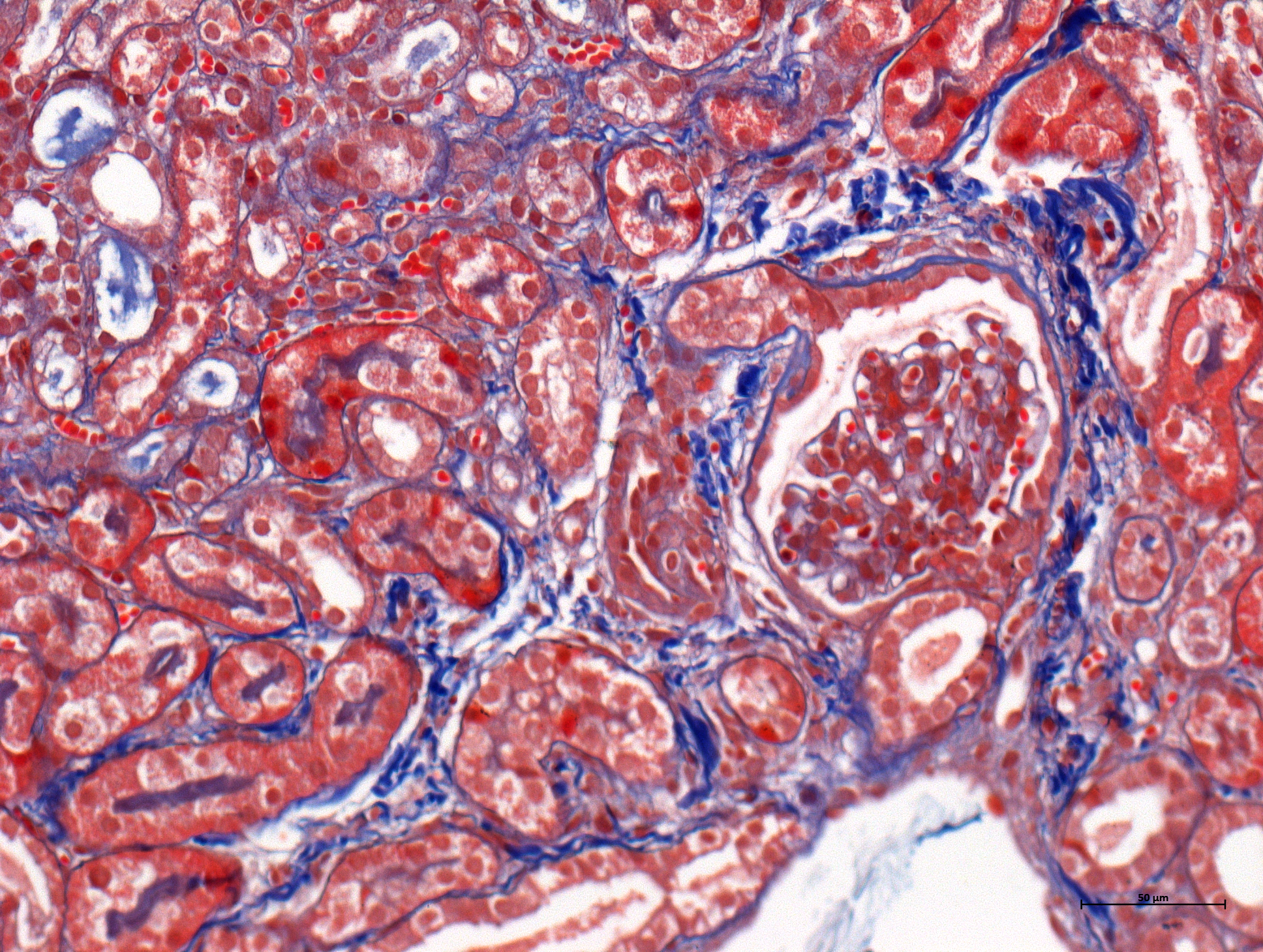

Supplement: Supplementary file 12 — Source Data for Figure 6 [file EMMM-15-e16581-s005.zip › Figure 6/6K/WT+UUO.tif]

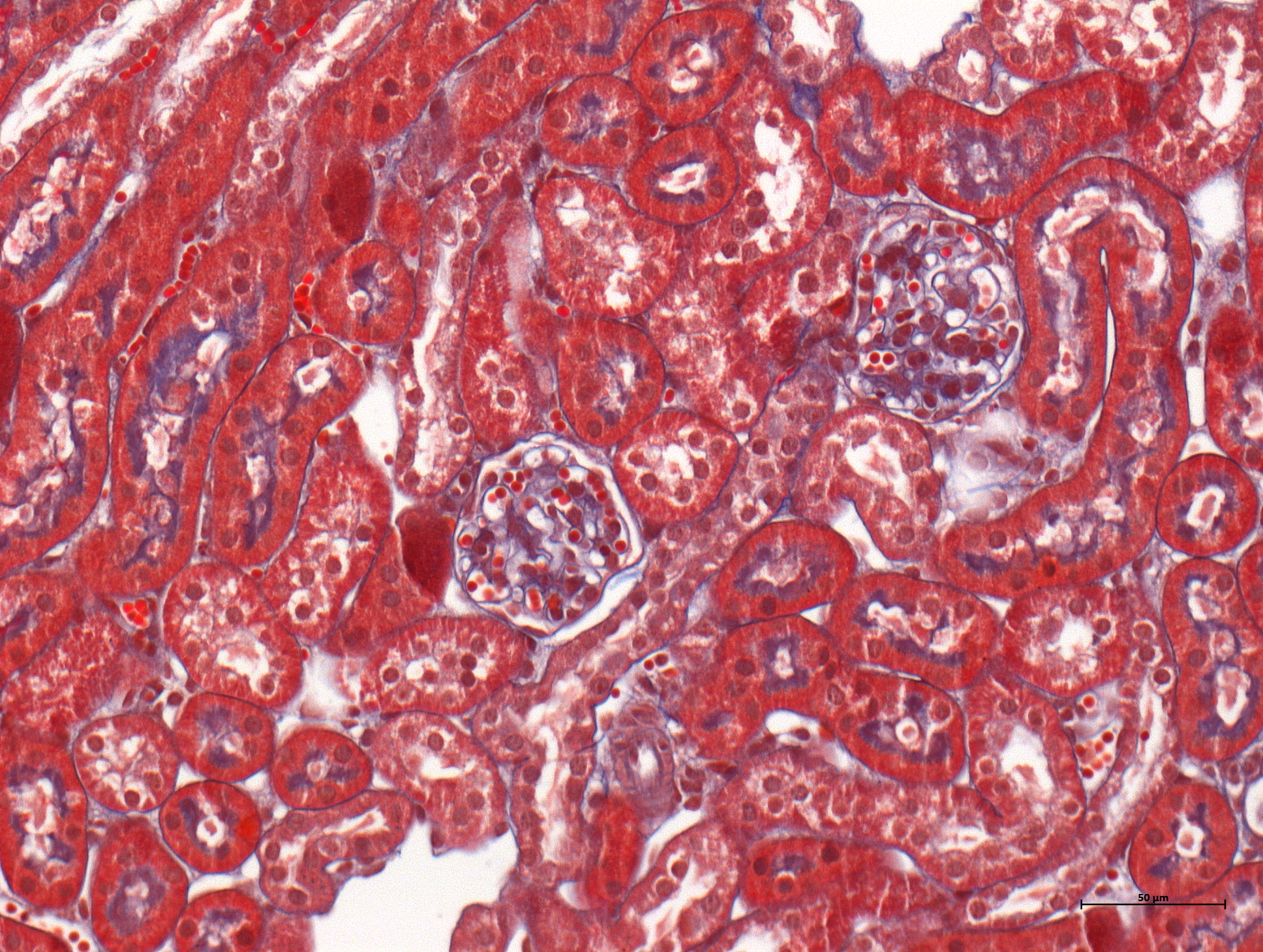

Supplement: Supplementary file 12 — Source Data for Figure 6 [file EMMM-15-e16581-s005.zip › Figure 6/6K/WT.tif]

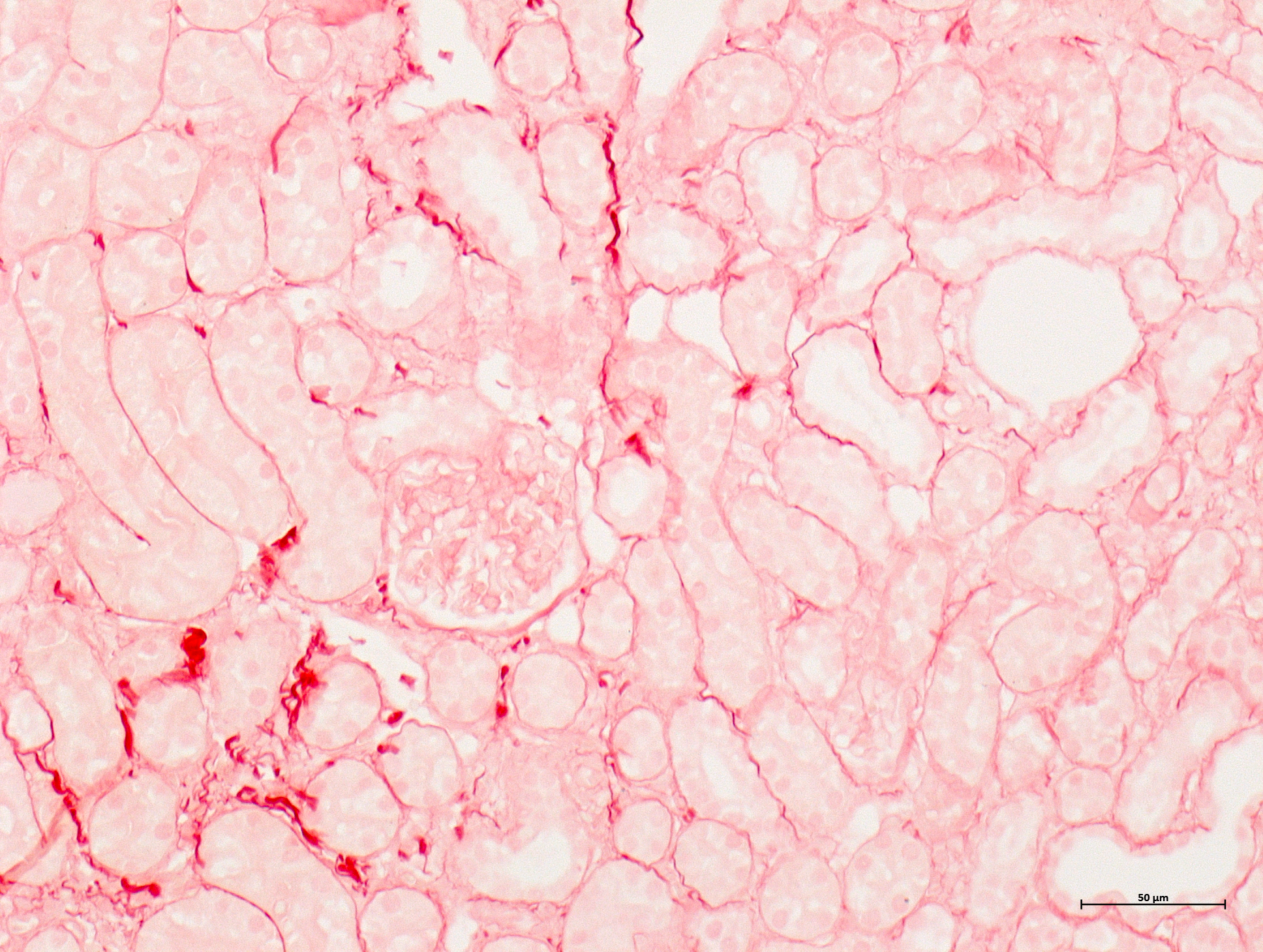

Supplement: Supplementary file 12 — Source Data for Figure 6 [file EMMM-15-e16581-s005.zip › Figure 6/6L J/Hmgcs2+UUO.tif]

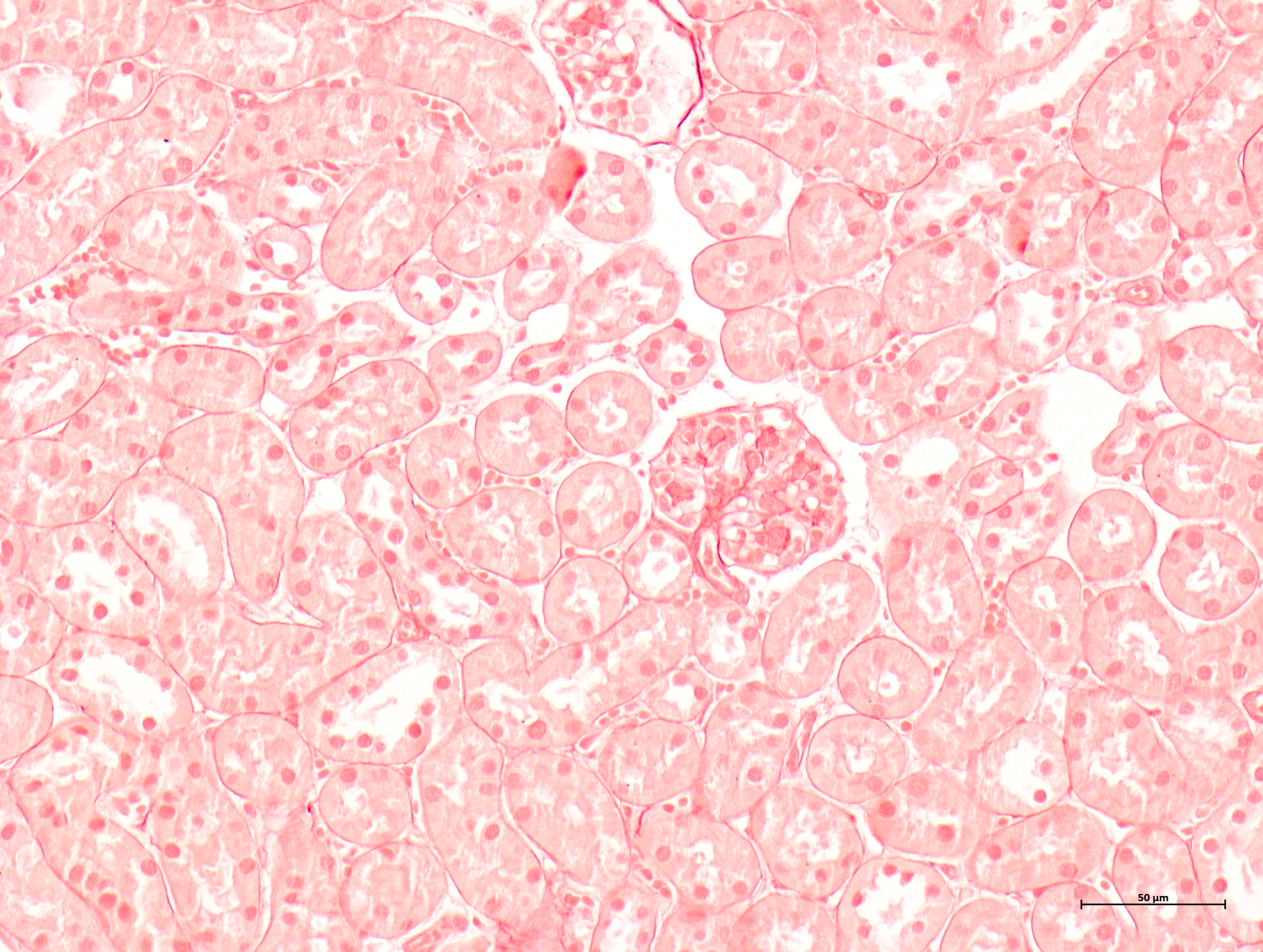

Supplement: Supplementary file 12 — Source Data for Figure 6 [file EMMM-15-e16581-s005.zip › Figure 6/6L J/Hmgcs2.tif]

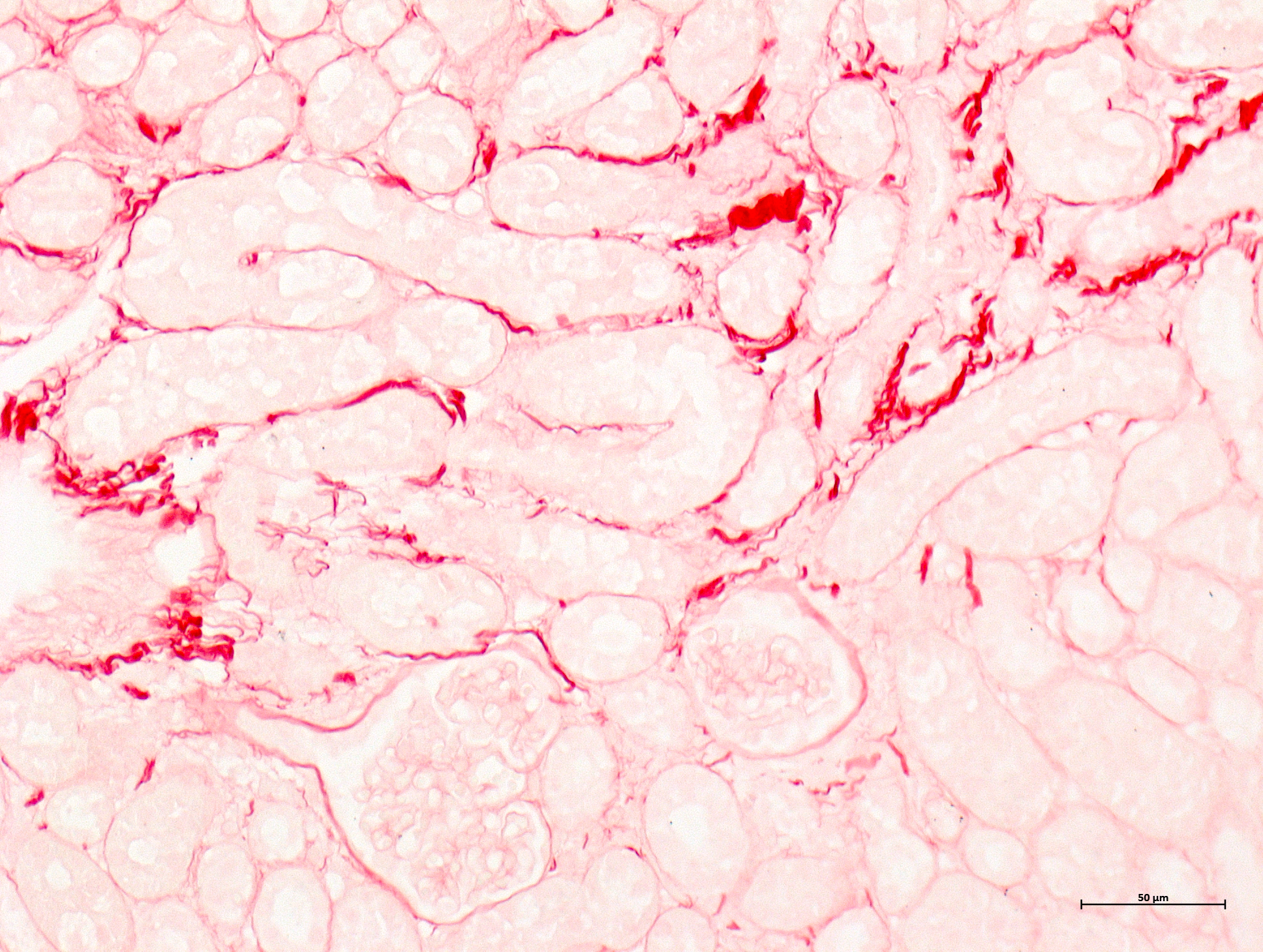

Supplement: Supplementary file 12 — Source Data for Figure 6 [file EMMM-15-e16581-s005.zip › Figure 6/6L J/WT+UUO.tif]

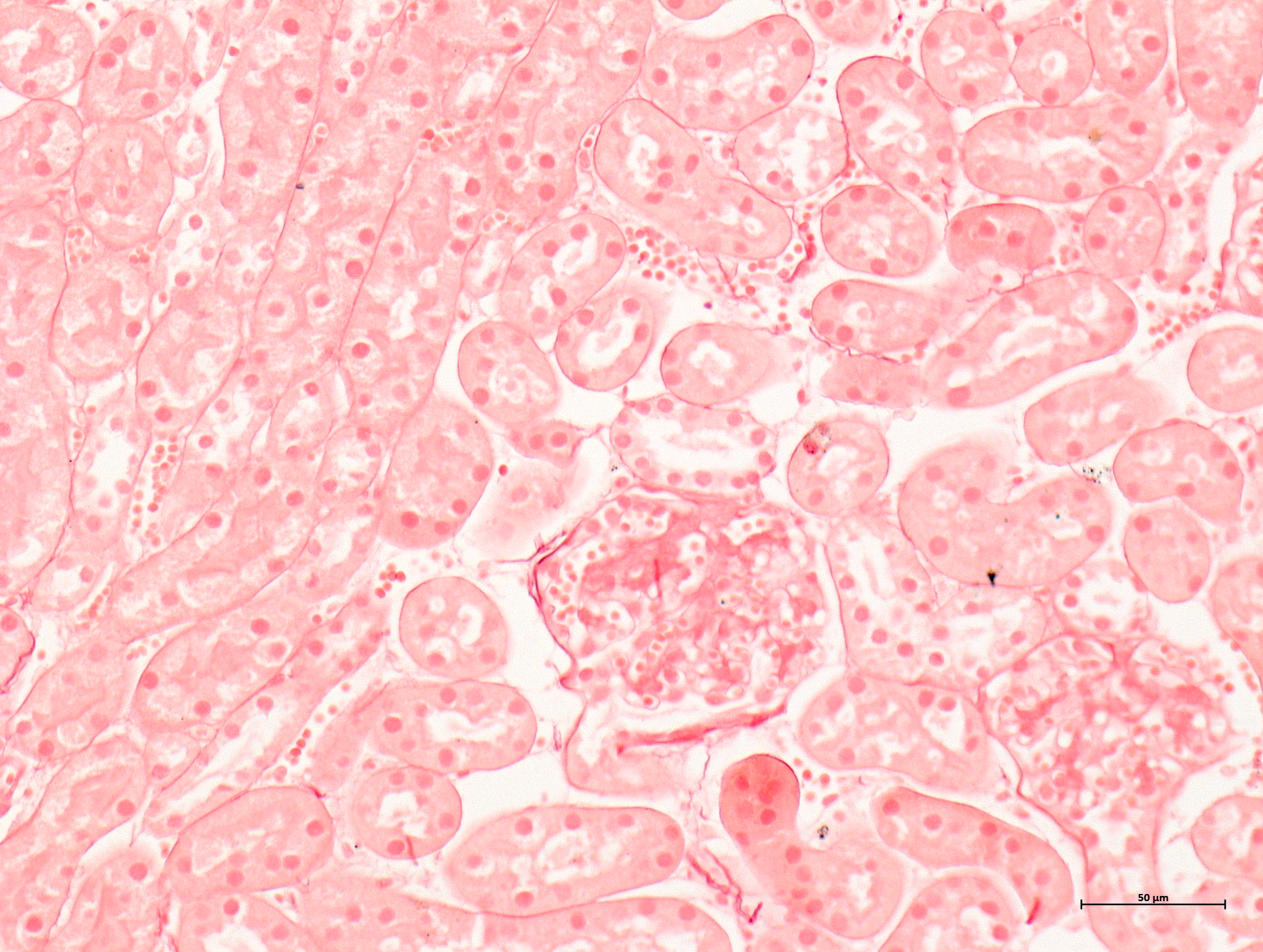

Supplement: Supplementary file 12 — Source Data for Figure 6 [file EMMM-15-e16581-s005.zip › Figure 6/6L J/WT.tif]

Fig 6M

FN1

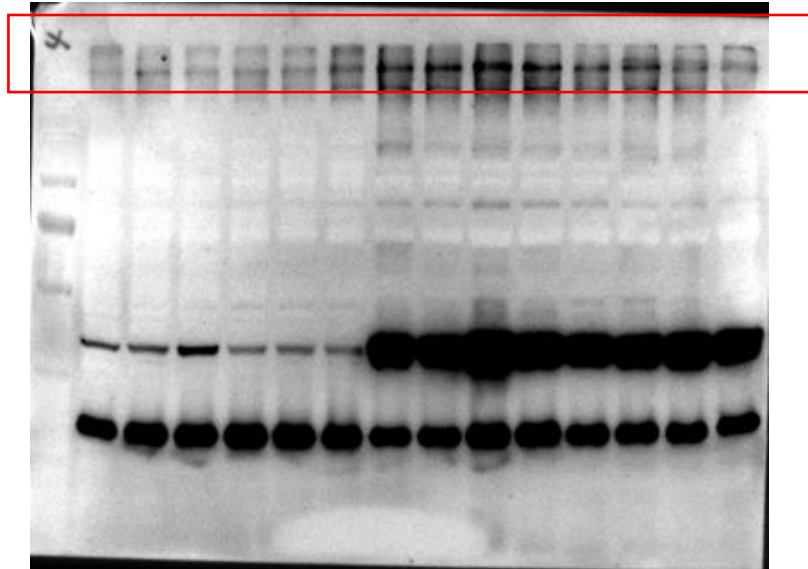

$\alpha$ -SMA

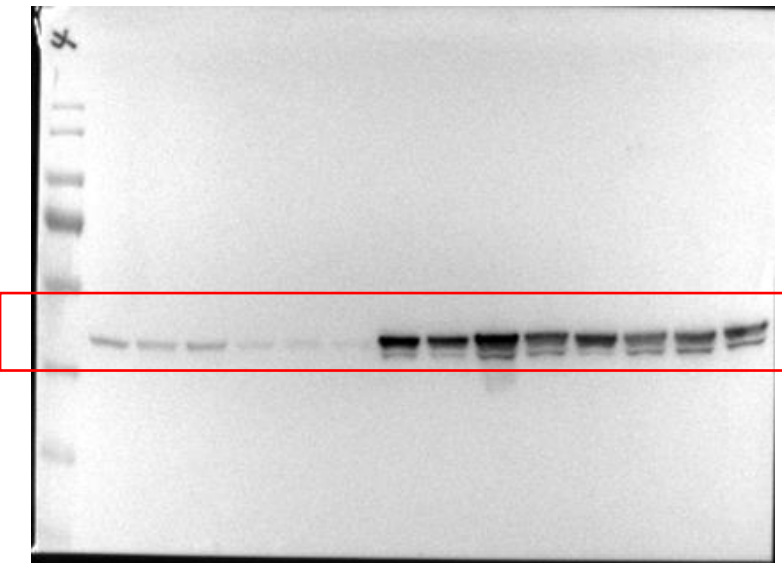

GAPDH

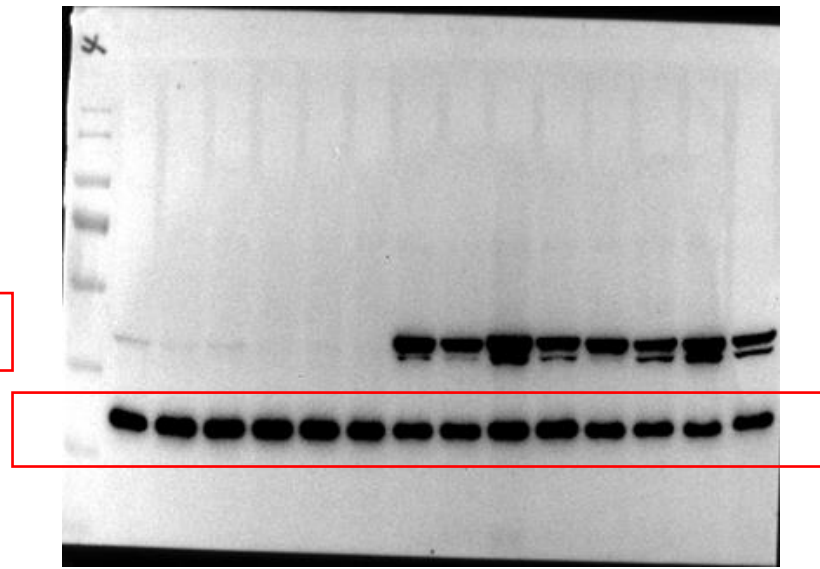

Collagen III

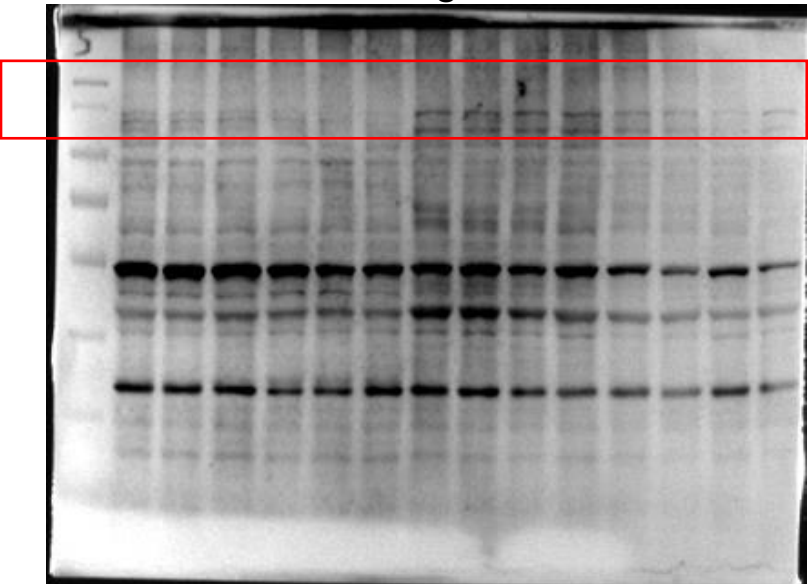

GAPDH

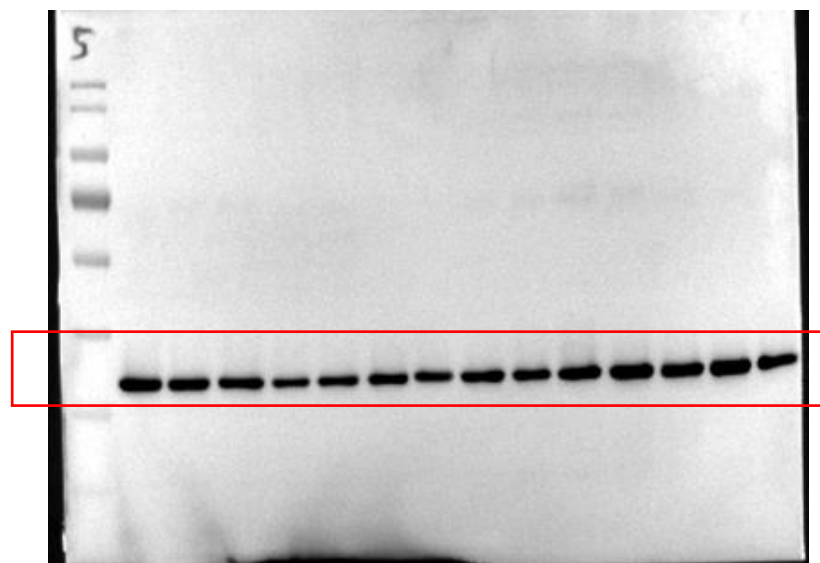

Supplement: Supplementary file 12 — Source Data for Figure 6 [file EMMM-15-e16581-s005.zip › Figure 6/6M-N/western gel.pdf]

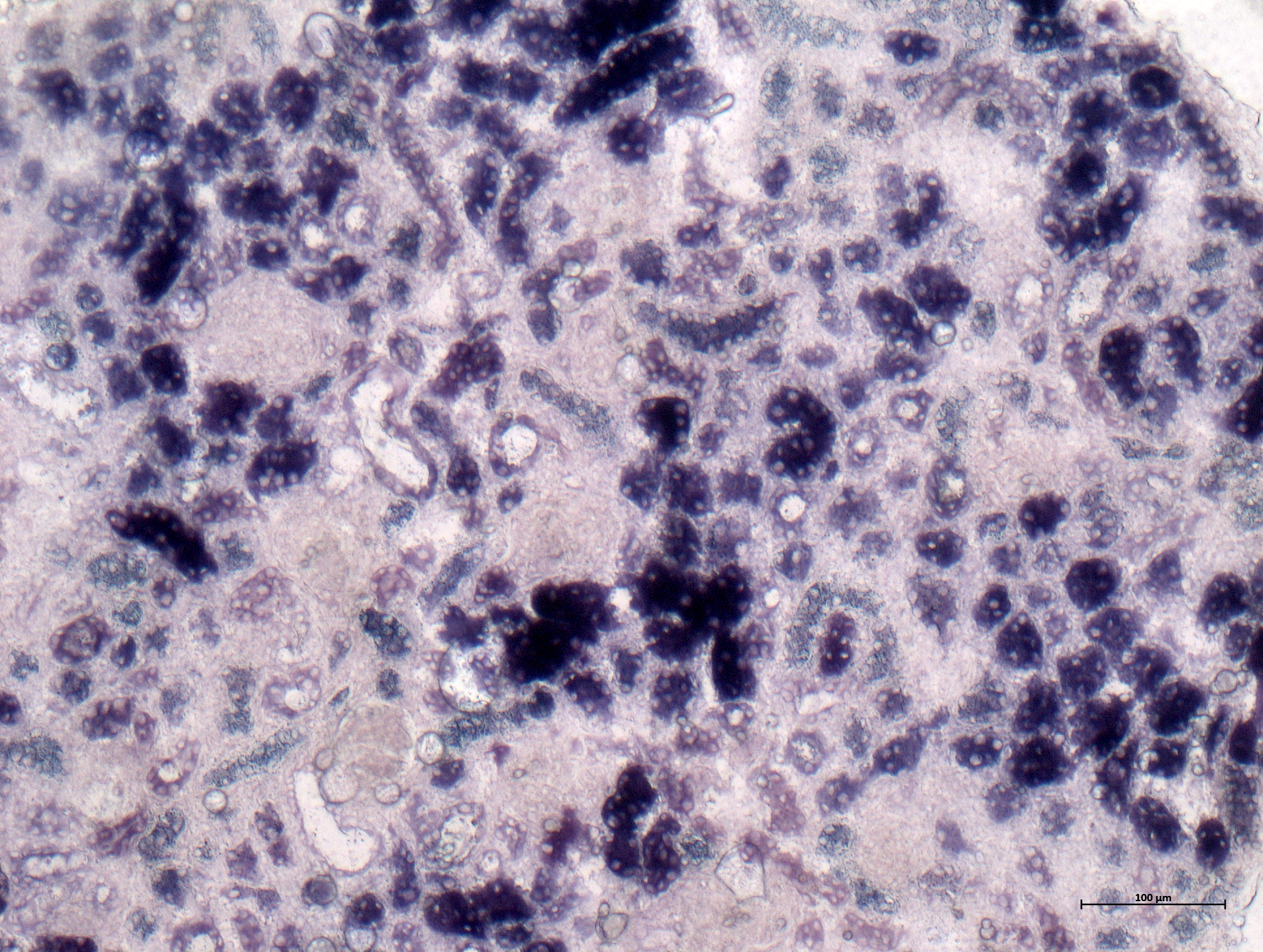

Supplement: Supplementary file 12 — Source Data for Figure 6 [file EMMM-15-e16581-s005.zip › Figure 6/6P/Hmgcs2+UUO.tif]

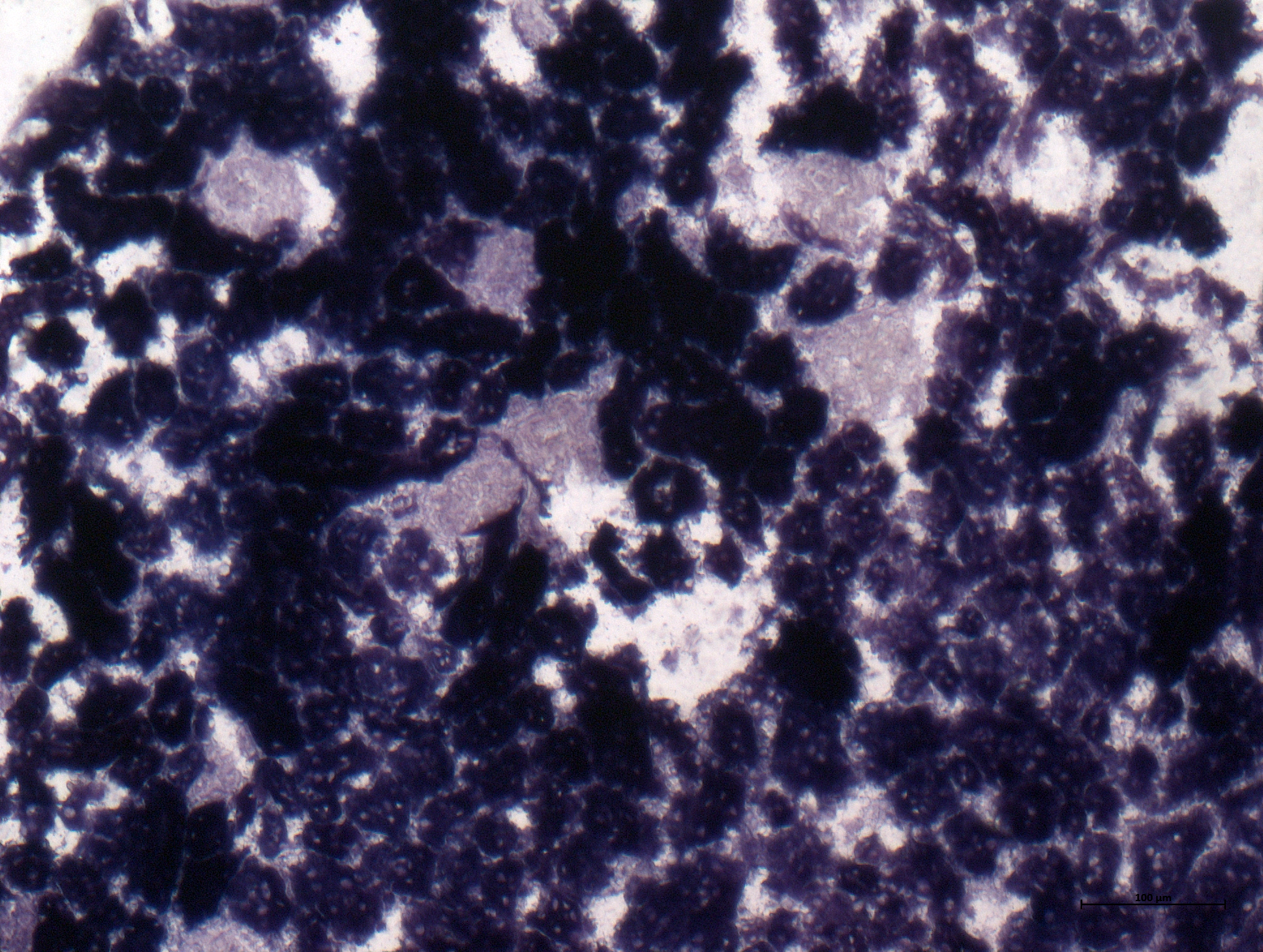

Supplement: Supplementary file 12 — Source Data for Figure 6 [file EMMM-15-e16581-s005.zip › Figure 6/6P/Hmgcs2.tif]

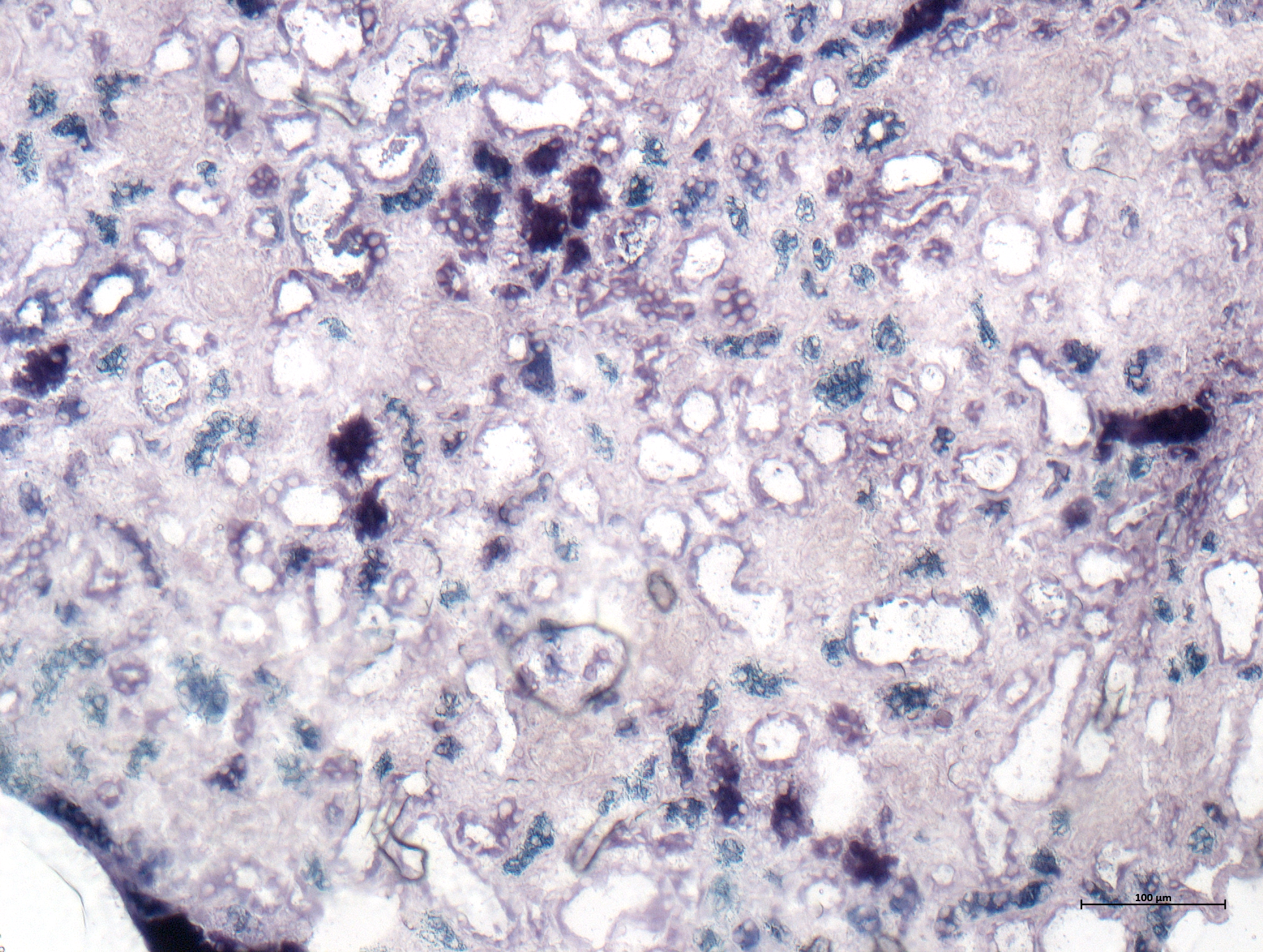

Supplement: Supplementary file 12 — Source Data for Figure 6 [file EMMM-15-e16581-s005.zip › Figure 6/6P/WT+UUO.tif]

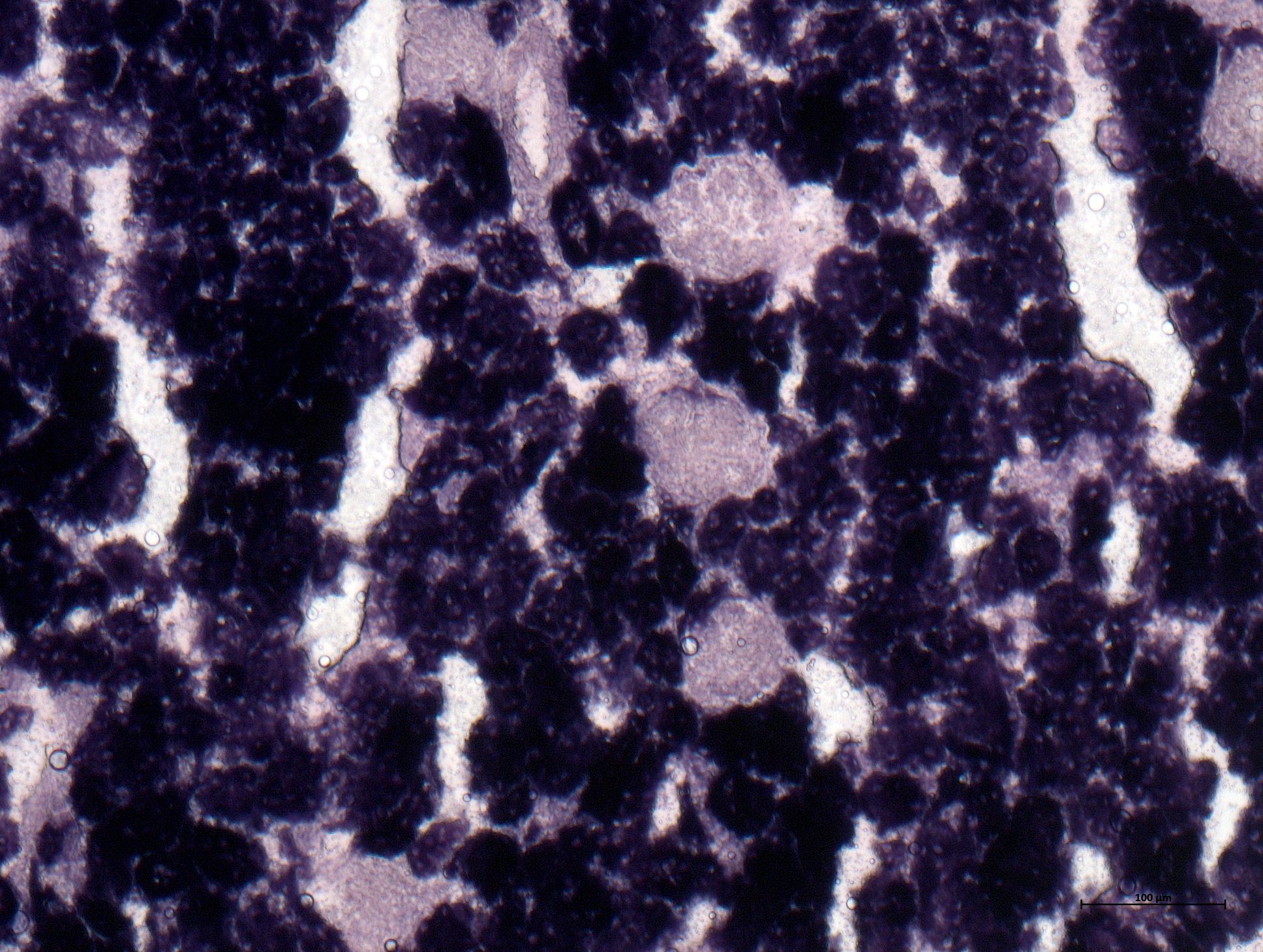

Supplement: Supplementary file 12 — Source Data for Figure 6 [file EMMM-15-e16581-s005.zip › Figure 6/6P/WT.tif]

Fig 7A

TFAM-His

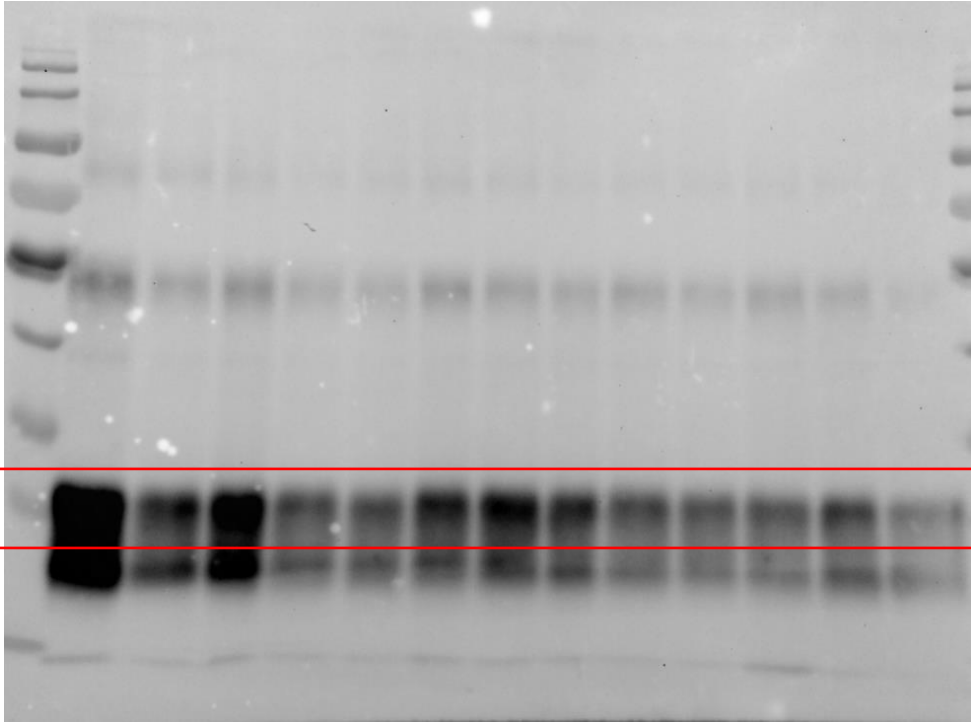

LONP1-GST

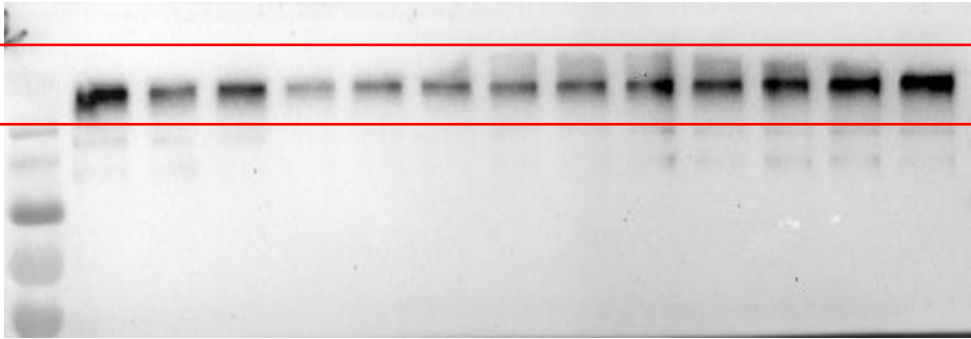

Supplement: Supplementary file 13 — Source Data for Figure 7 [file EMMM-15-e16581-s012.zip › Figure 7/7A/western gel.pdf]

Fig 7F

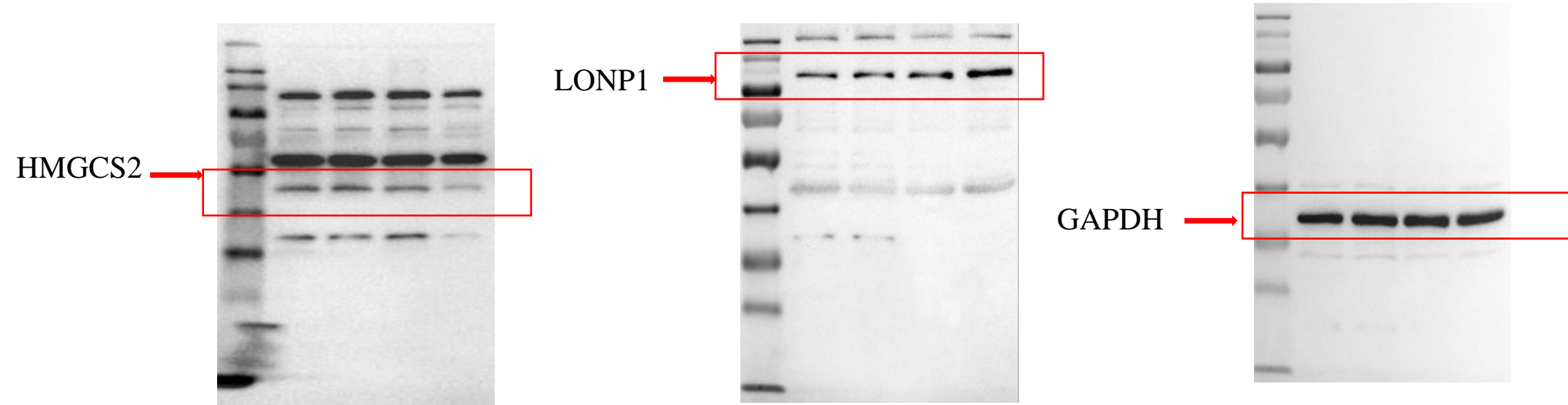

Supplement: Supplementary file 13 — Source Data for Figure 7 [file EMMM-15-e16581-s012.zip › Figure 7/7F/western gel.pdf]

Fig 7G

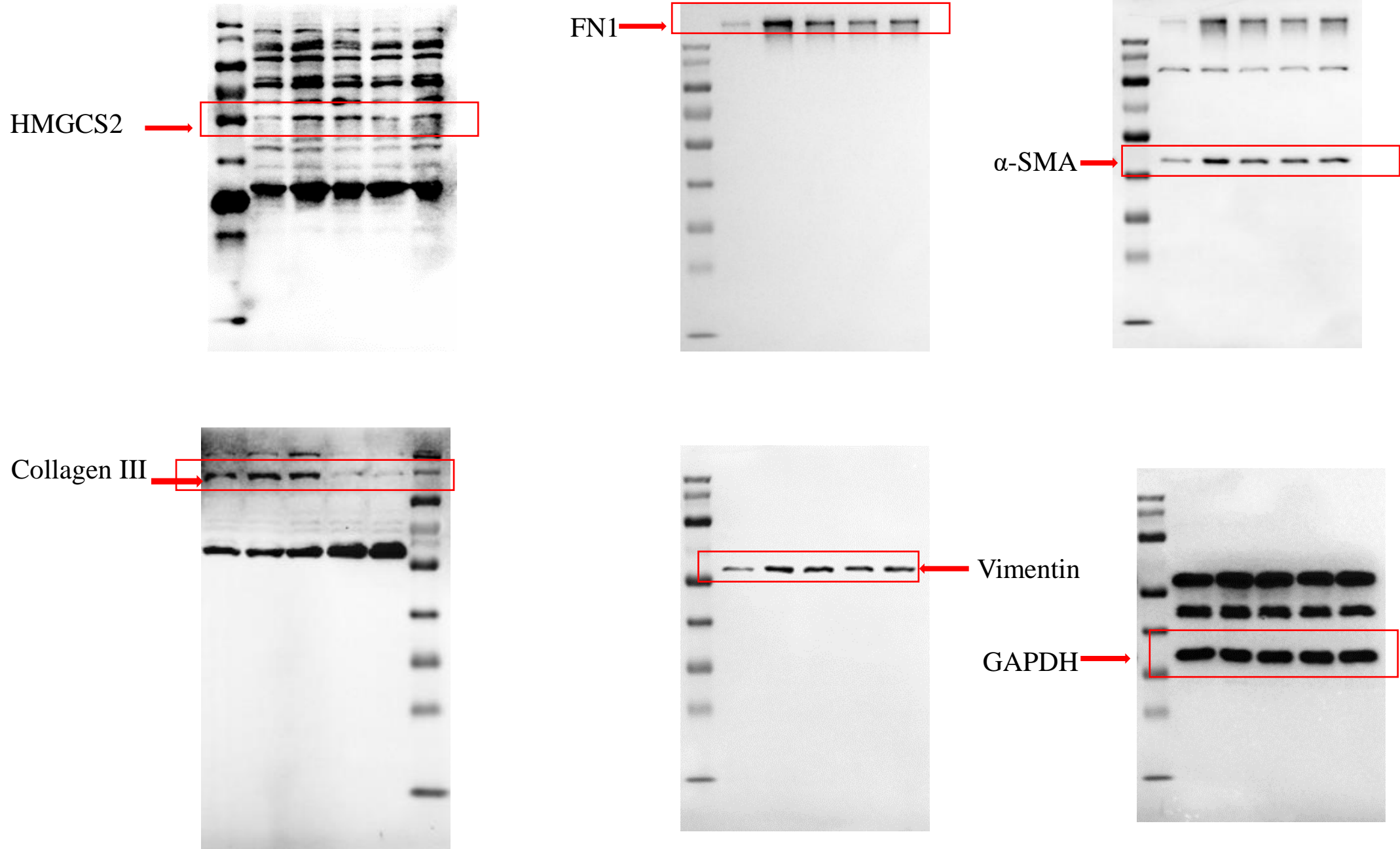

Supplement: Supplementary file 13 — Source Data for Figure 7 [file EMMM-15-e16581-s012.zip › Figure 7/7G-H/western gel.pdf]

Fig 7J

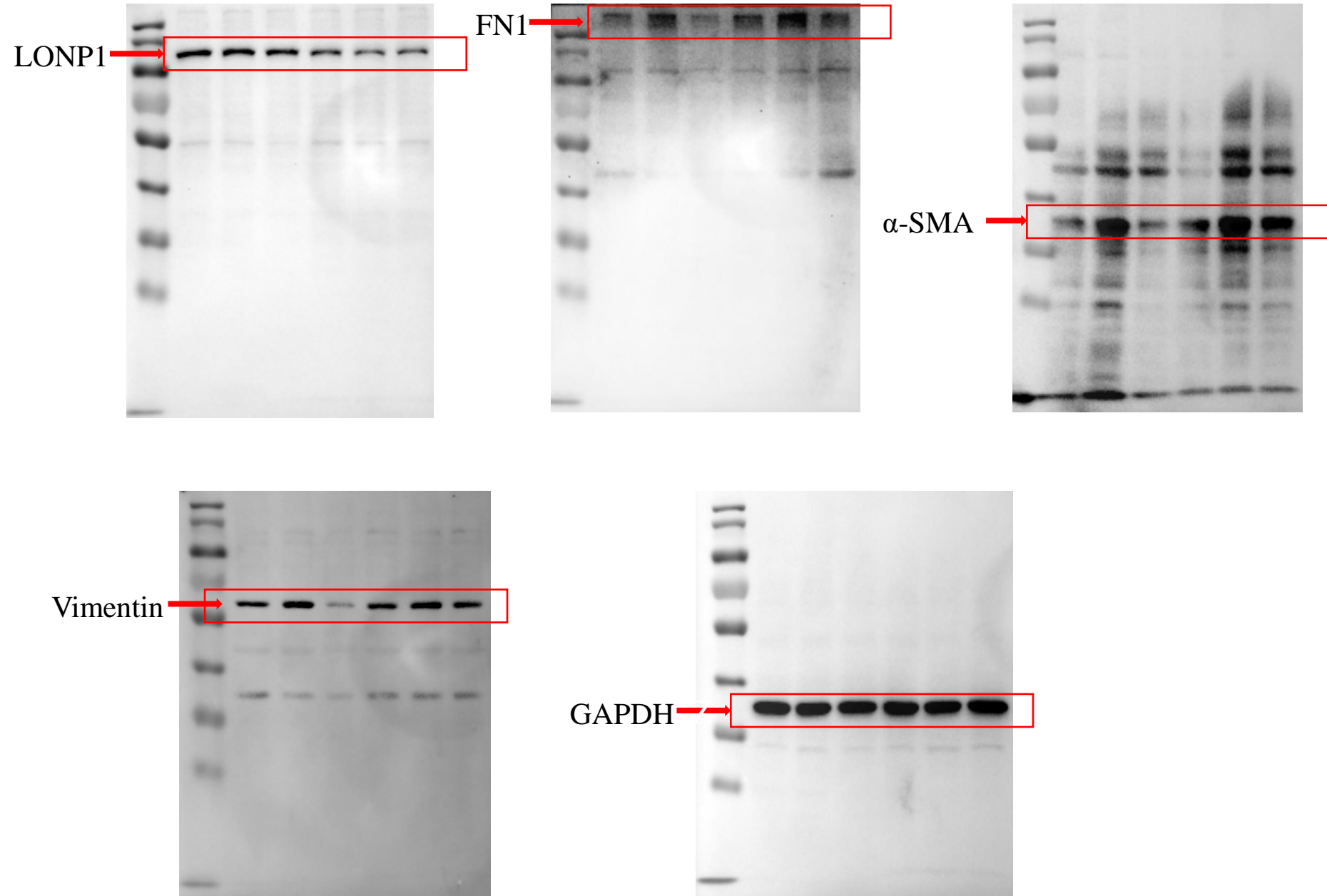

Supplement: Supplementary file 13 — Source Data for Figure 7 [file EMMM-15-e16581-s012.zip › Figure 7/7J-N/western gel.pdf]

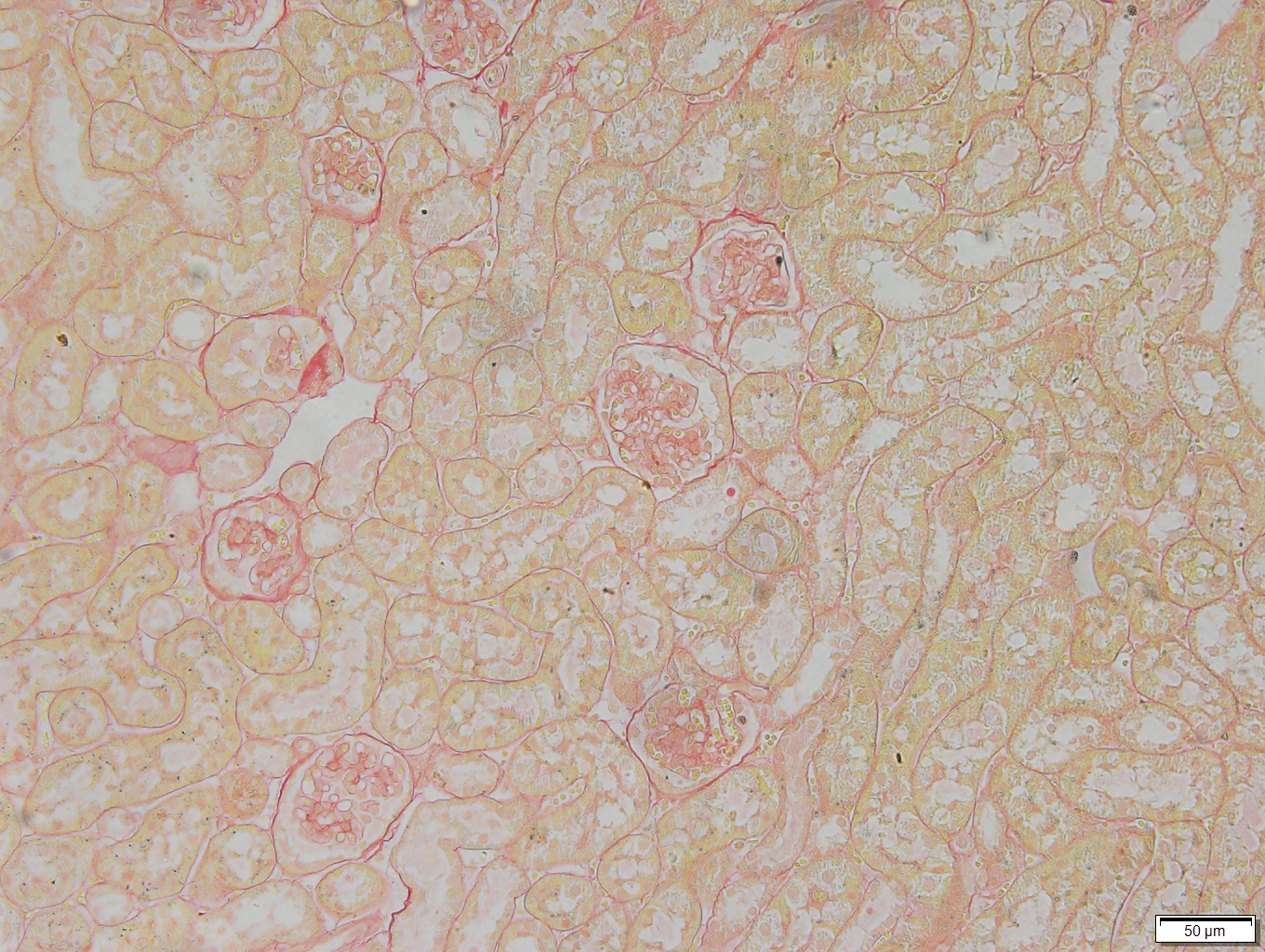

Supplement: Supplementary file 14 — Source Data for Figure 8 [file EMMM-15-e16581-s006.zip › Figure 8/8A-B/Sham.tif]

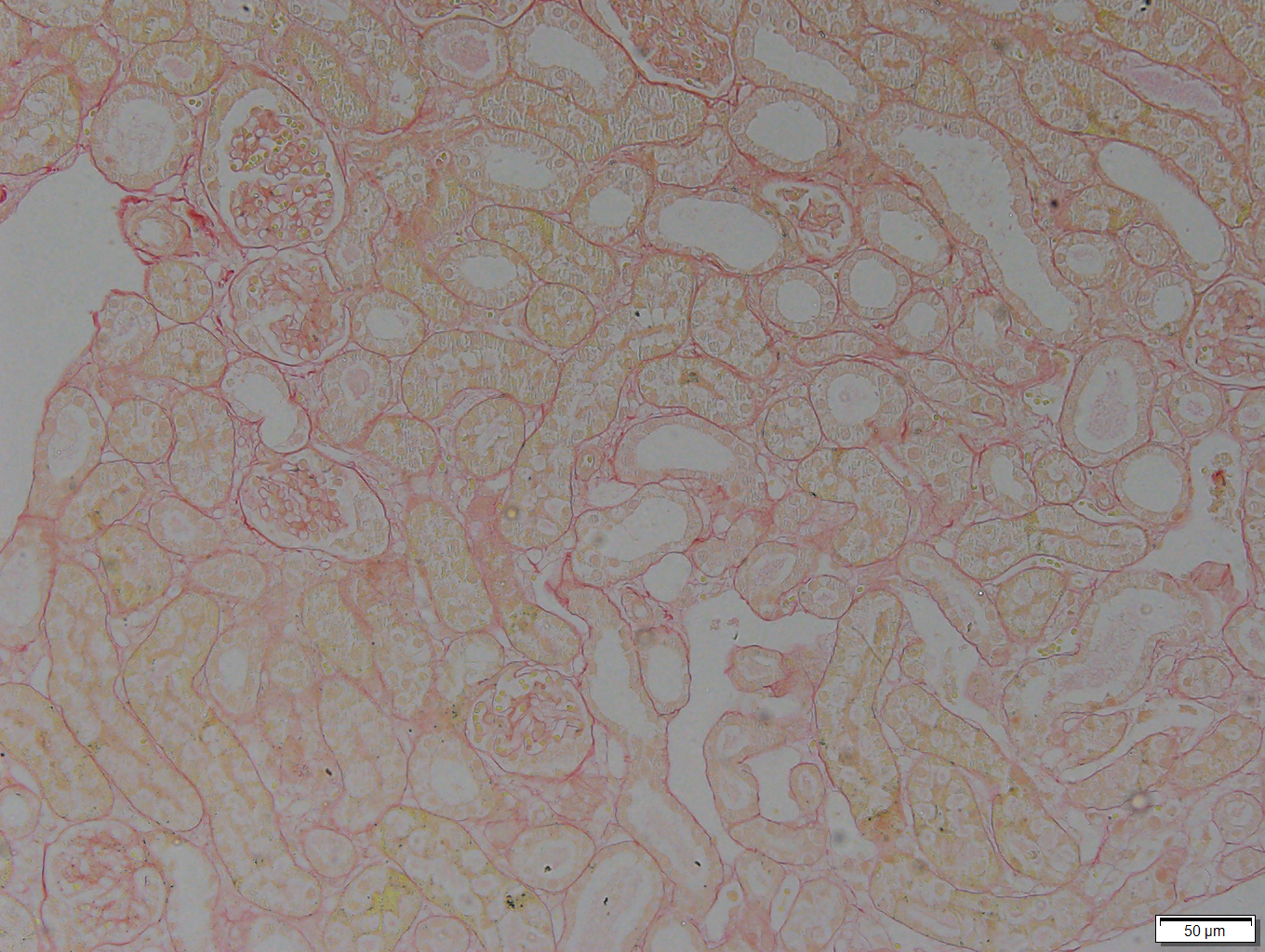

Supplement: Supplementary file 14 — Source Data for Figure 8 [file EMMM-15-e16581-s006.zip › Figure 8/8A-B/UUO+HD.tif]

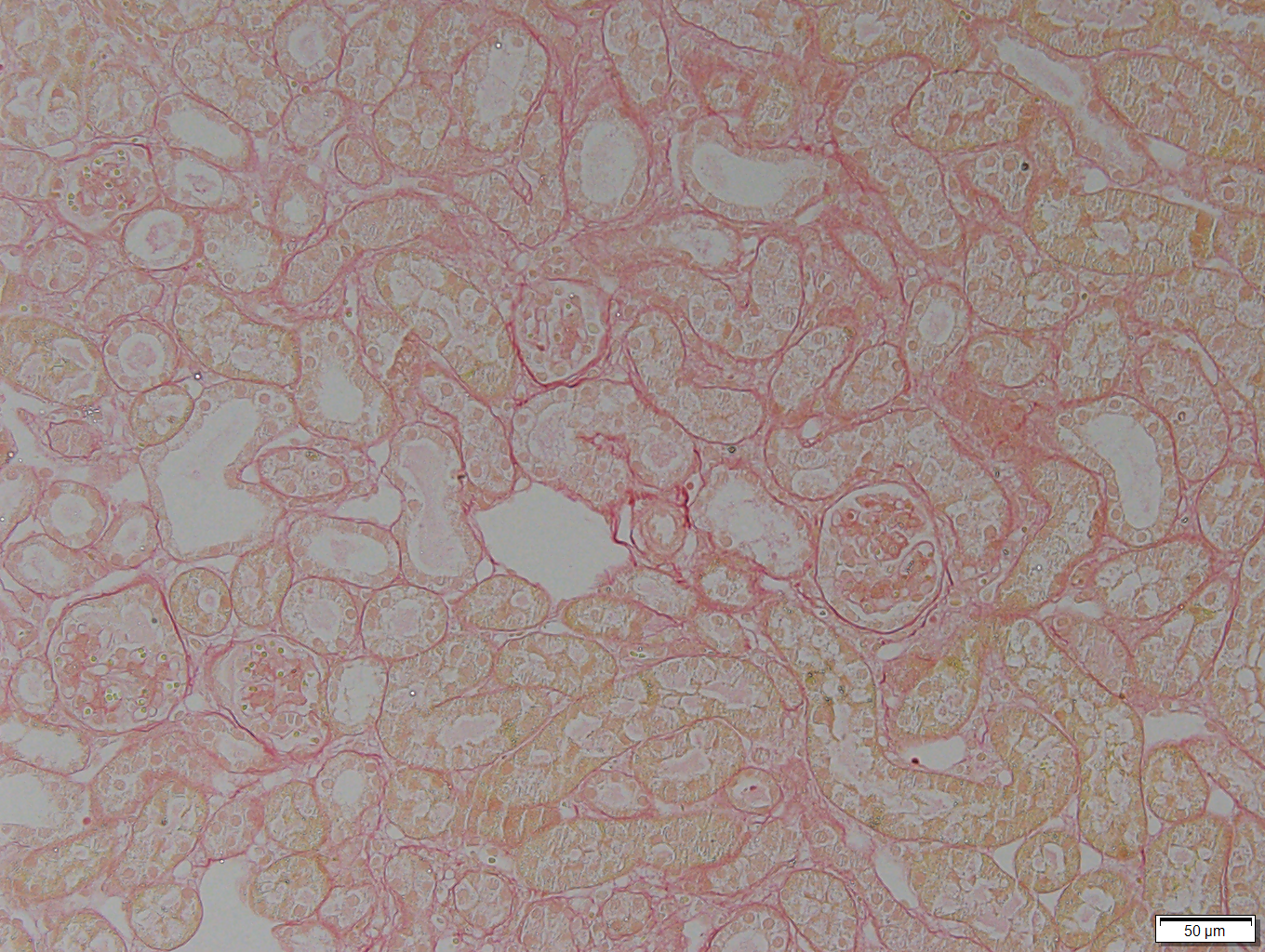

Supplement: Supplementary file 14 — Source Data for Figure 8 [file EMMM-15-e16581-s006.zip › Figure 8/8A-B/UUO+LD.tif]

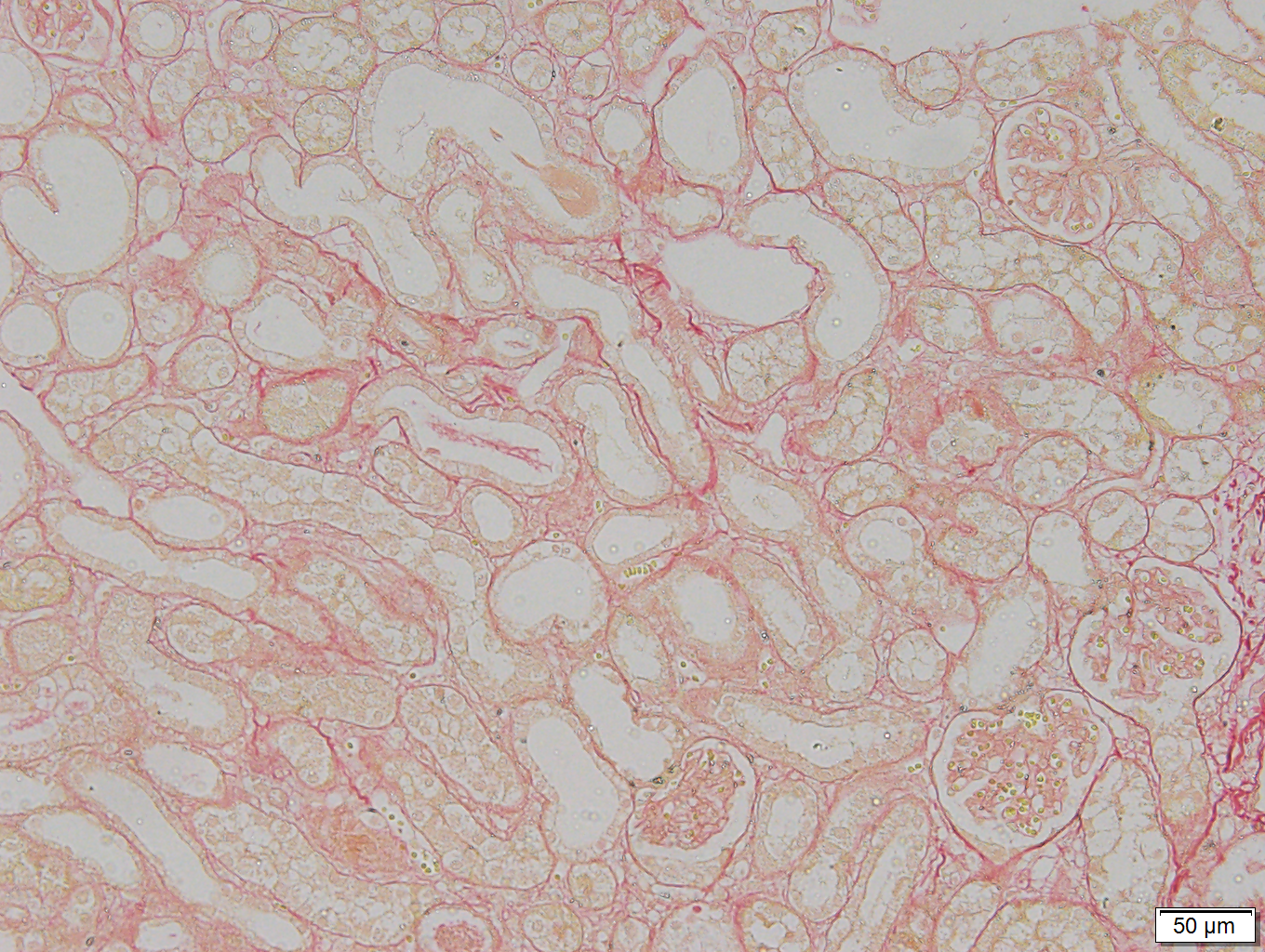

Supplement: Supplementary file 14 — Source Data for Figure 8 [file EMMM-15-e16581-s006.zip › Figure 8/8A-B/UUO.tif]

Fig 8C

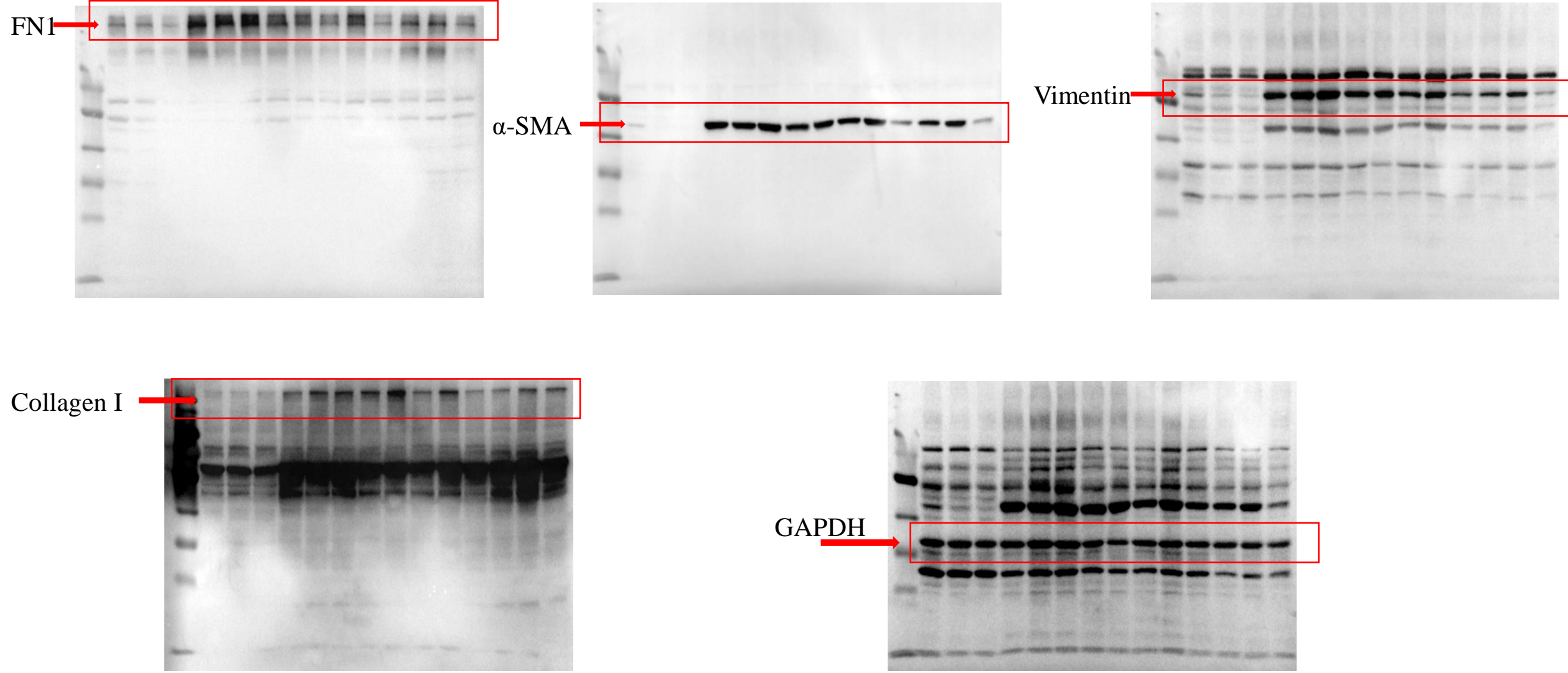

Supplement: Supplementary file 14 — Source Data for Figure 8 [file EMMM-15-e16581-s006.zip › Figure 8/8C-D/western gel.pdf]

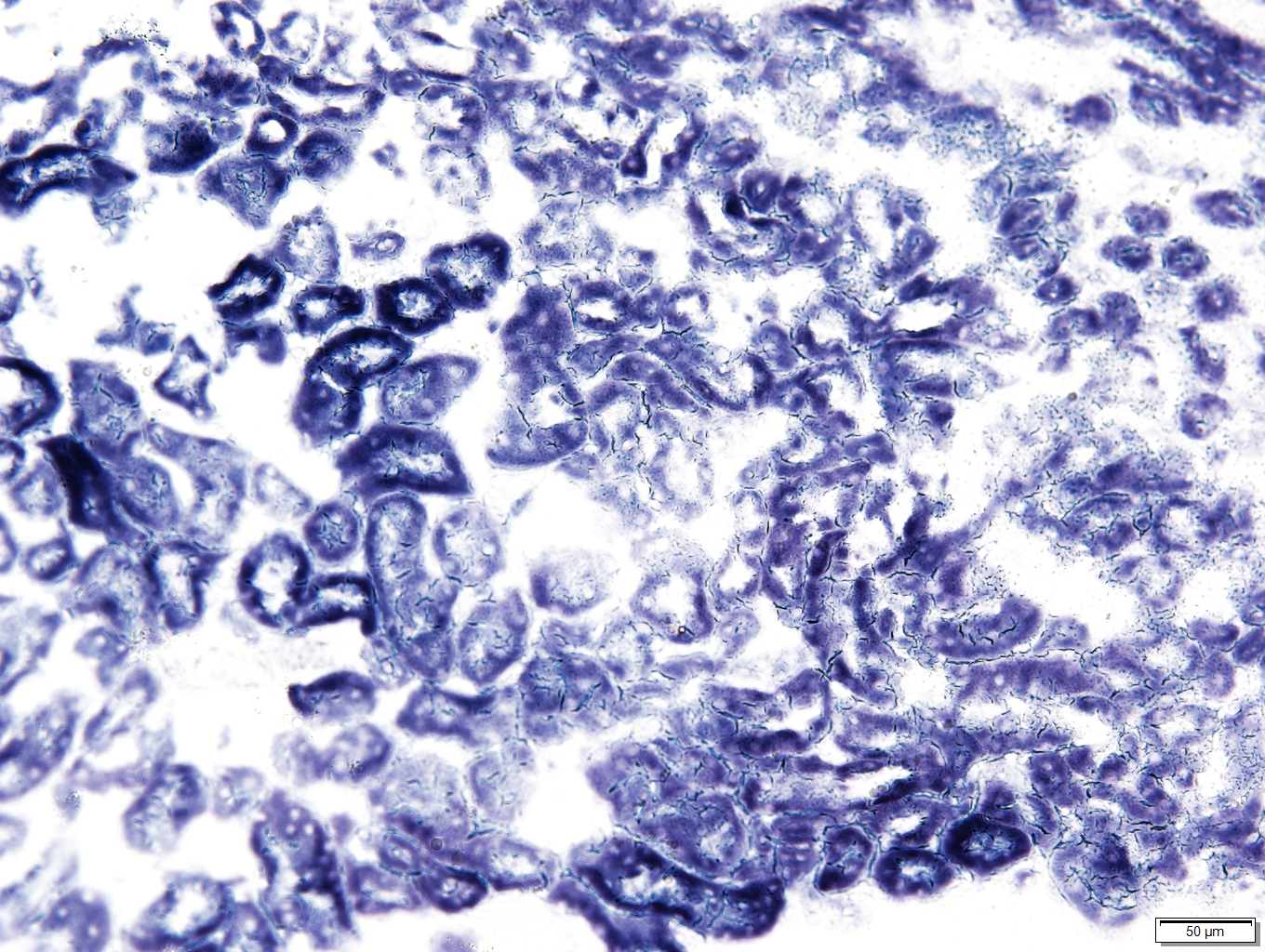

Supplement: Supplementary file 14 — Source Data for Figure 8 [file EMMM-15-e16581-s006.zip › Figure 8/8F/Sham.tif]

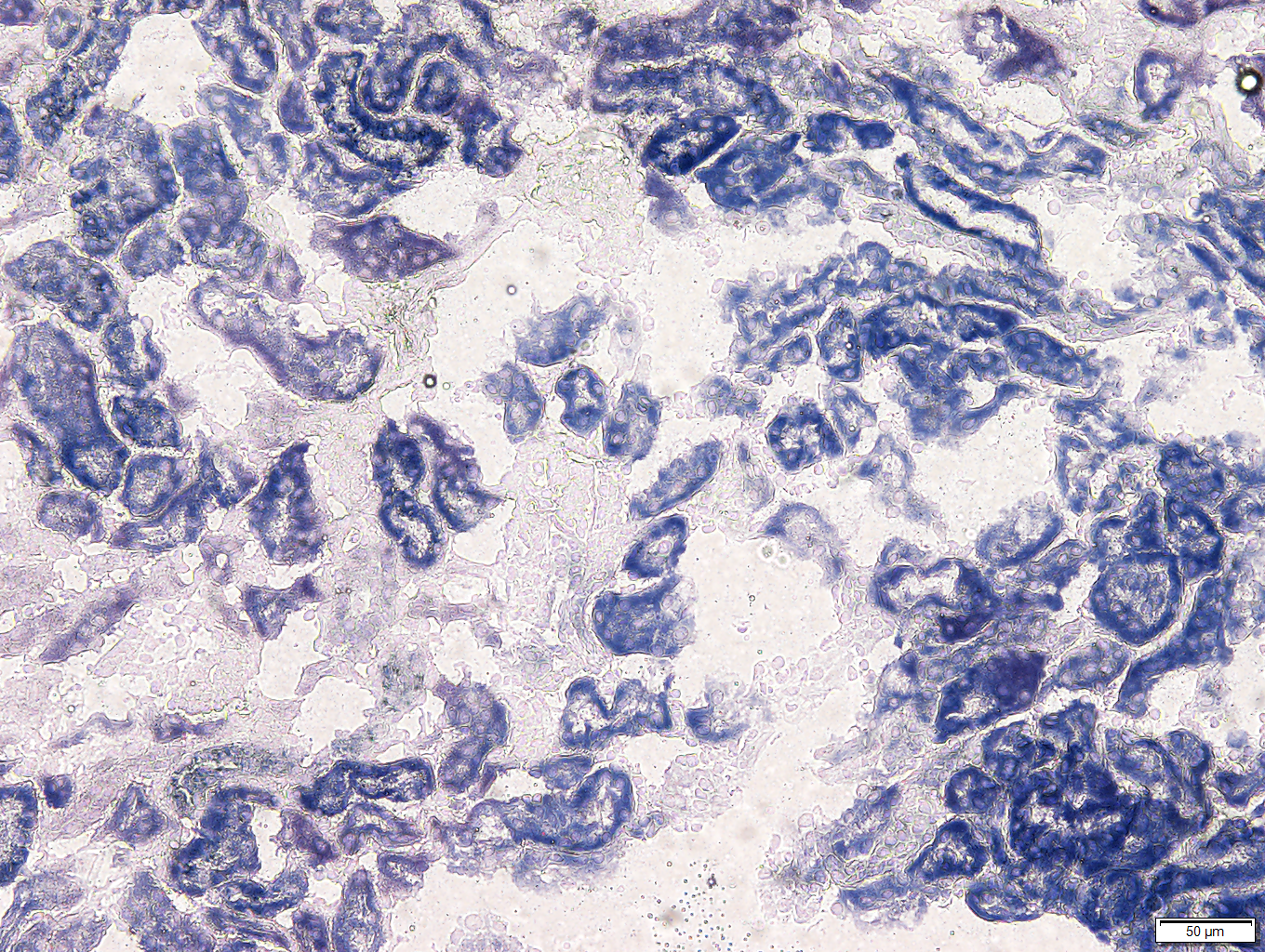

Supplement: Supplementary file 14 — Source Data for Figure 8 [file EMMM-15-e16581-s006.zip › Figure 8/8F/UUO+HD.tif]

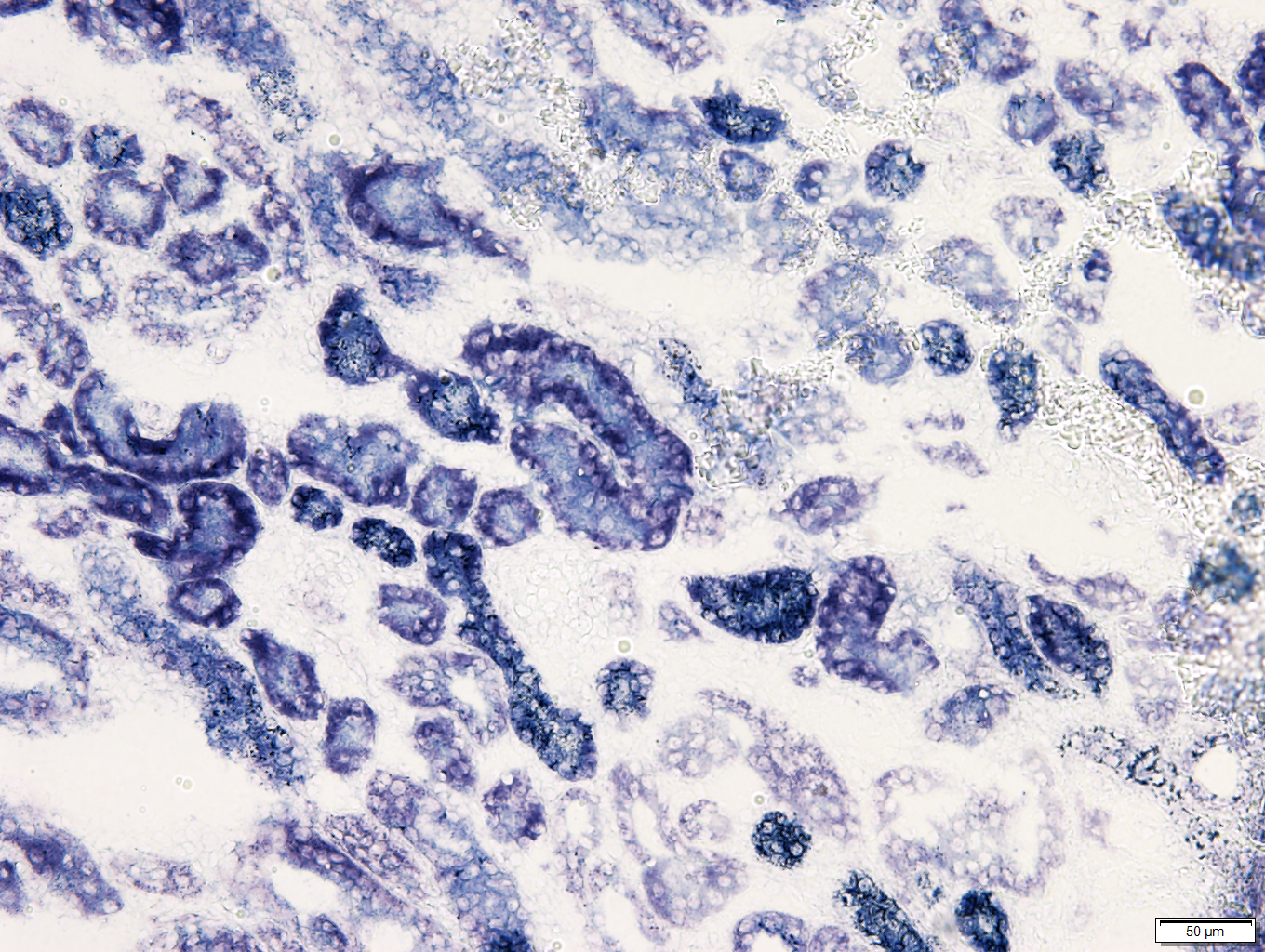

Supplement: Supplementary file 14 — Source Data for Figure 8 [file EMMM-15-e16581-s006.zip › Figure 8/8F/UUO+LD.tif]

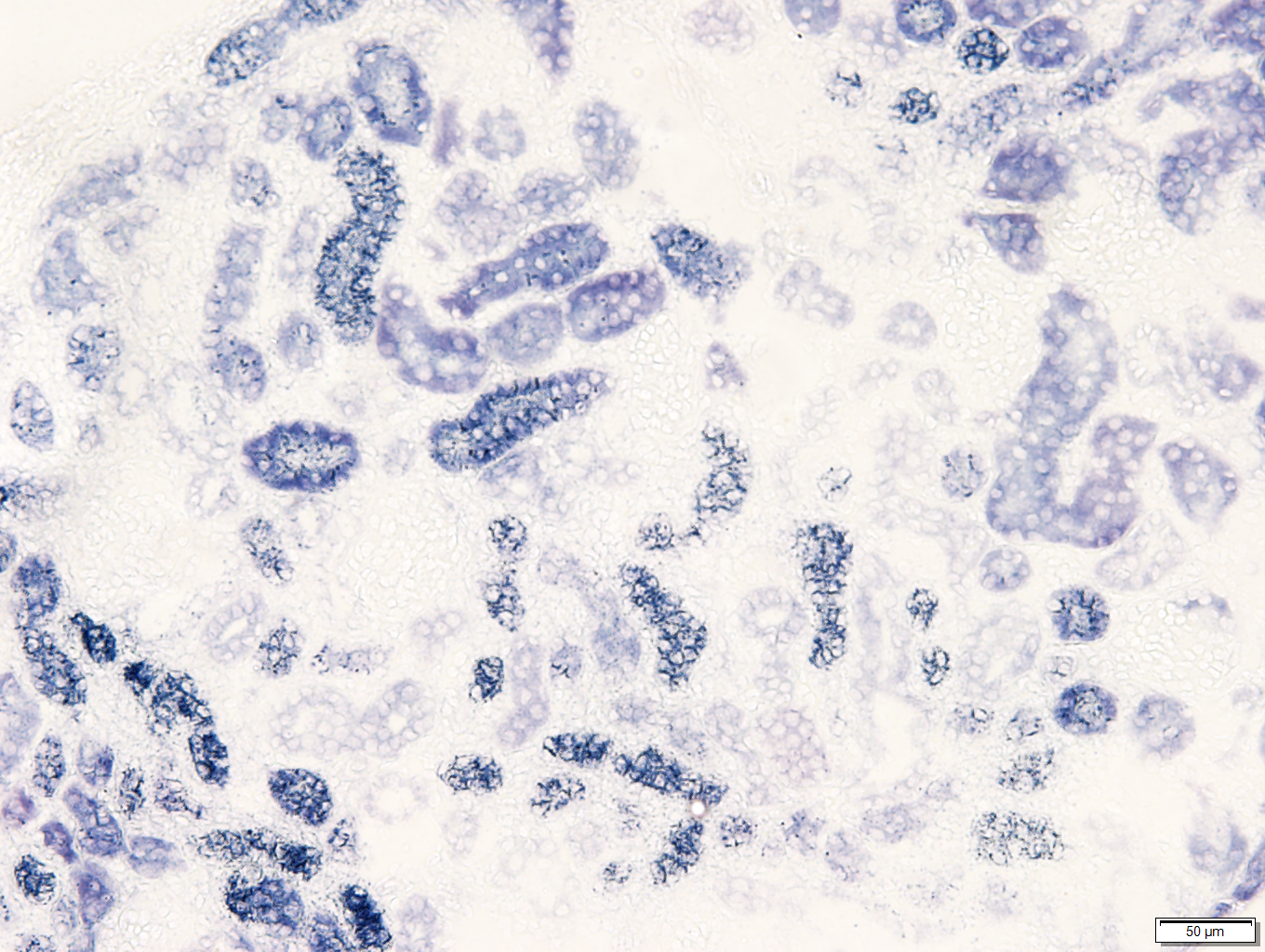

Supplement: Supplementary file 14 — Source Data for Figure 8 [file EMMM-15-e16581-s006.zip › Figure 8/8F/UUO.tif]

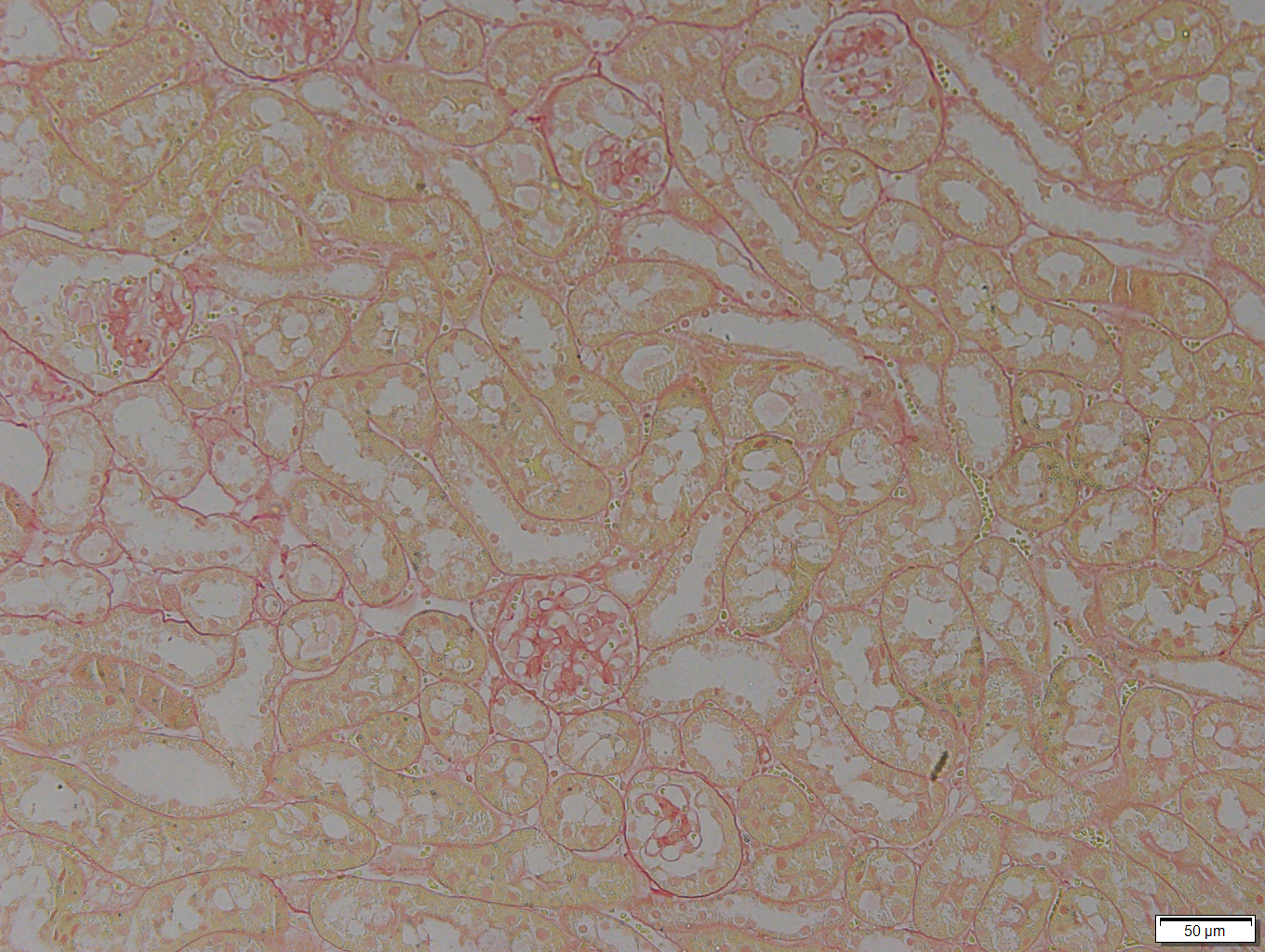

Supplement: Supplementary file 14 — Source Data for Figure 8 [file EMMM-15-e16581-s006.zip › Figure 8/8G-H/Sham.tif]

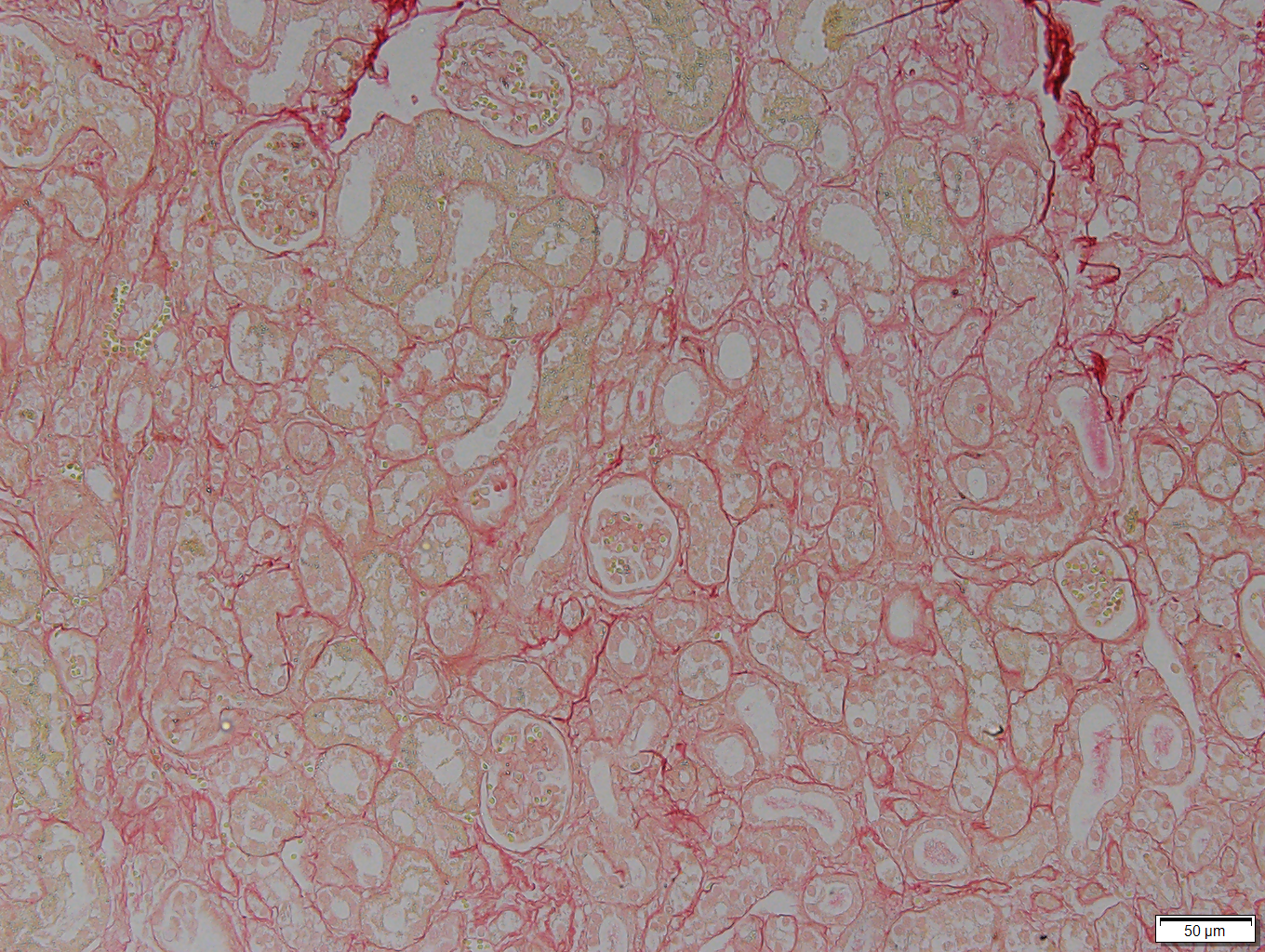

Supplement: Supplementary file 14 — Source Data for Figure 8 [file EMMM-15-e16581-s006.zip › Figure 8/8G-H/UIRI+HD.tif]

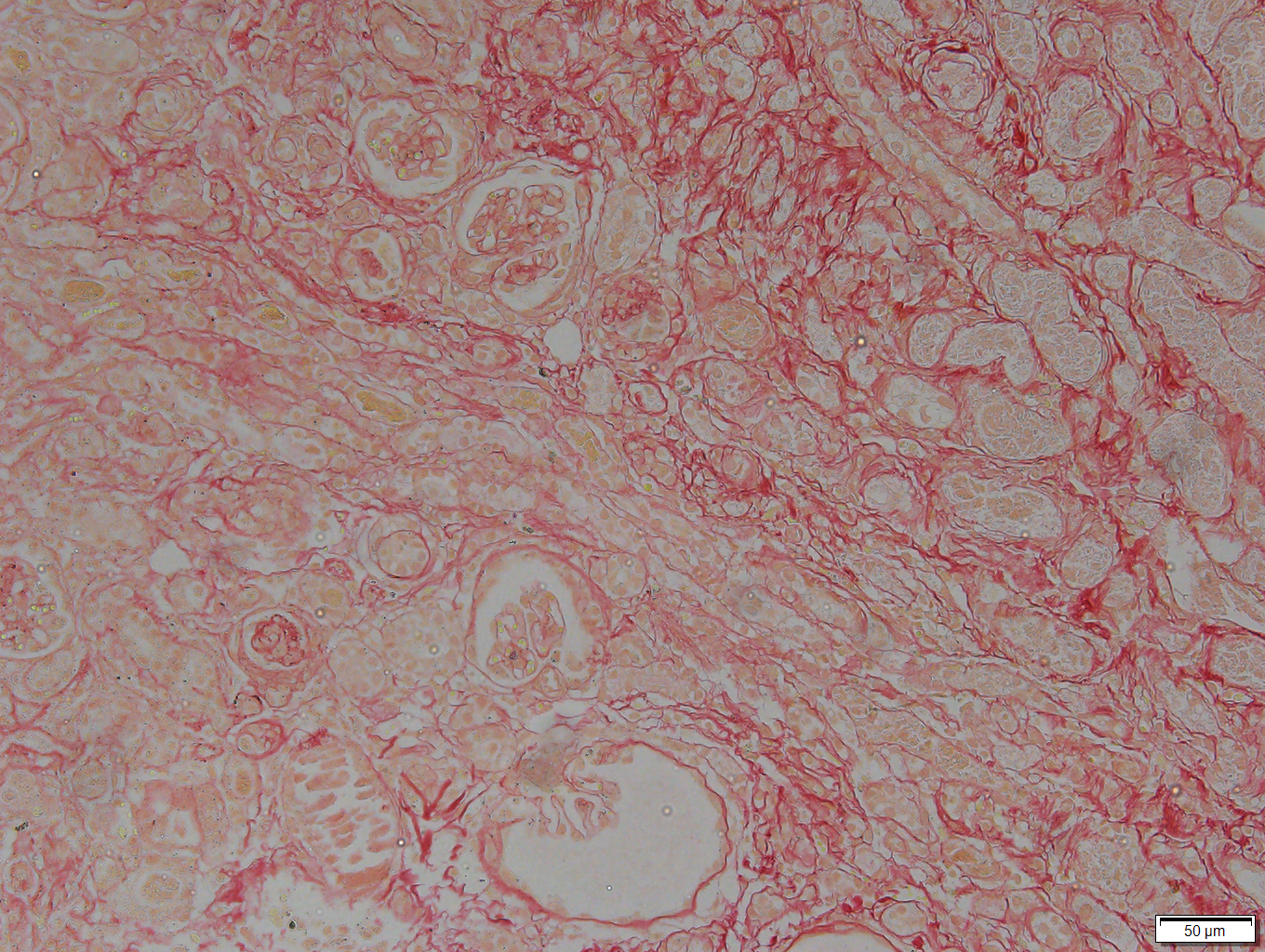

Supplement: Supplementary file 14 — Source Data for Figure 8 [file EMMM-15-e16581-s006.zip › Figure 8/8G-H/UIRI+LD.tif]

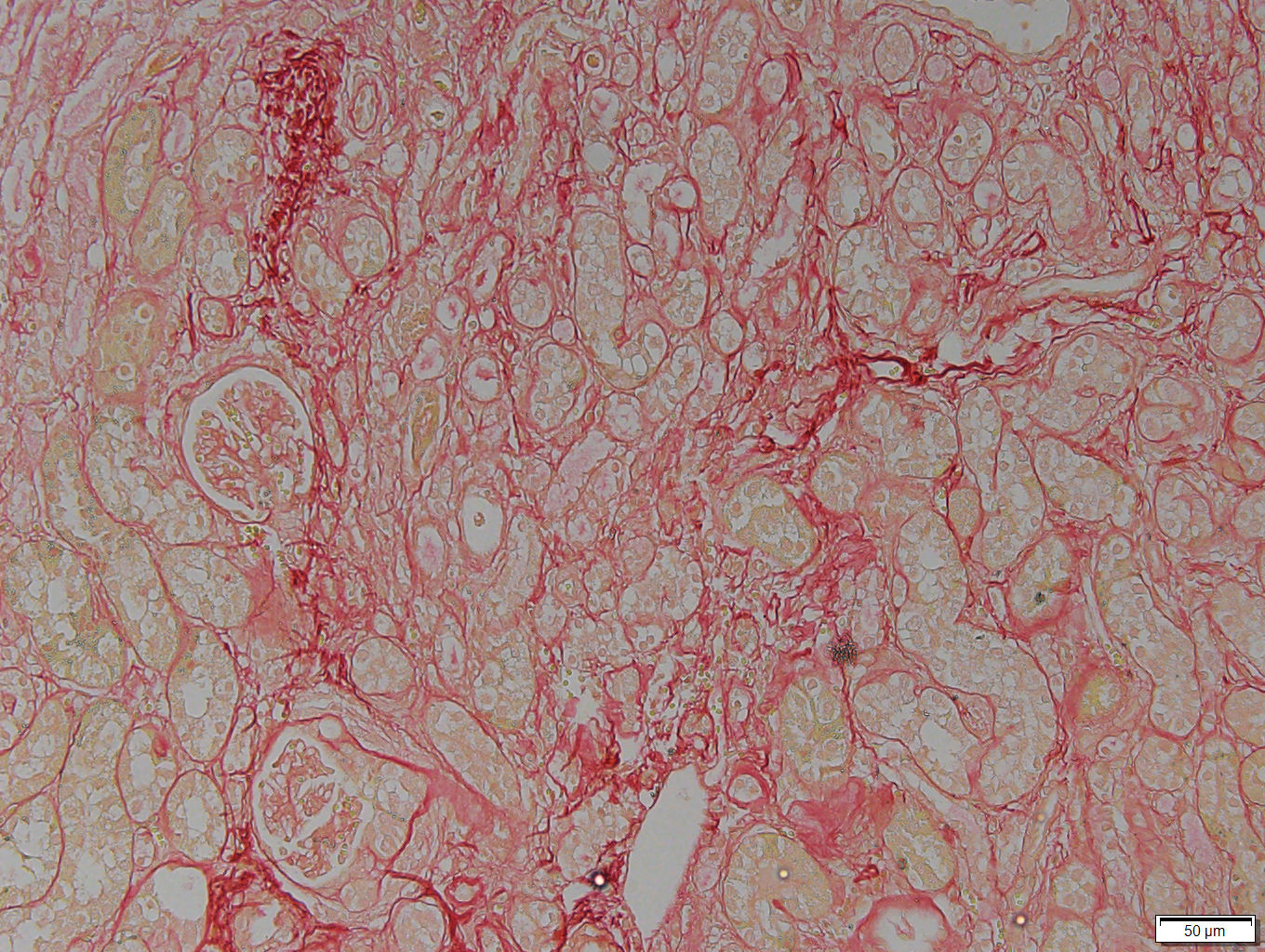

Supplement: Supplementary file 14 — Source Data for Figure 8 [file EMMM-15-e16581-s006.zip › Figure 8/8G-H/UIRI.tif]

Fig 8I

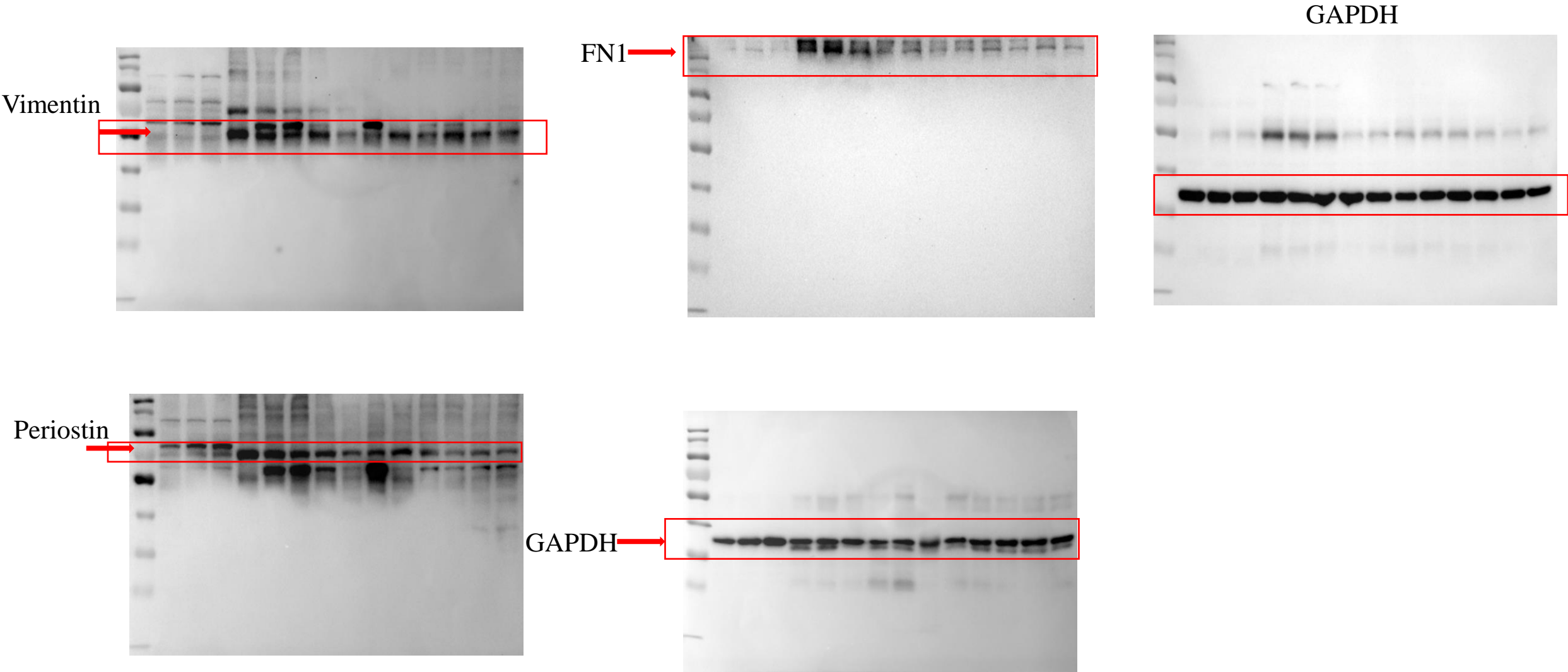

Supplement: Supplementary file 14 — Source Data for Figure 8 [file EMMM-15-e16581-s006.zip › Figure 8/8I-J/western gel.pdf]
