# Supplementary material for: LONP1 targets HMGCS2 to protect mitochondrial function and attenuate chronic kidney disease
Source: EMBO Mol Med. 2023 Jan 11;15(2):e16581. doi: 10.15252/emmm.202216581 (PMC9906428; doi:10.15252/emmm.202216581)

Fig 5C

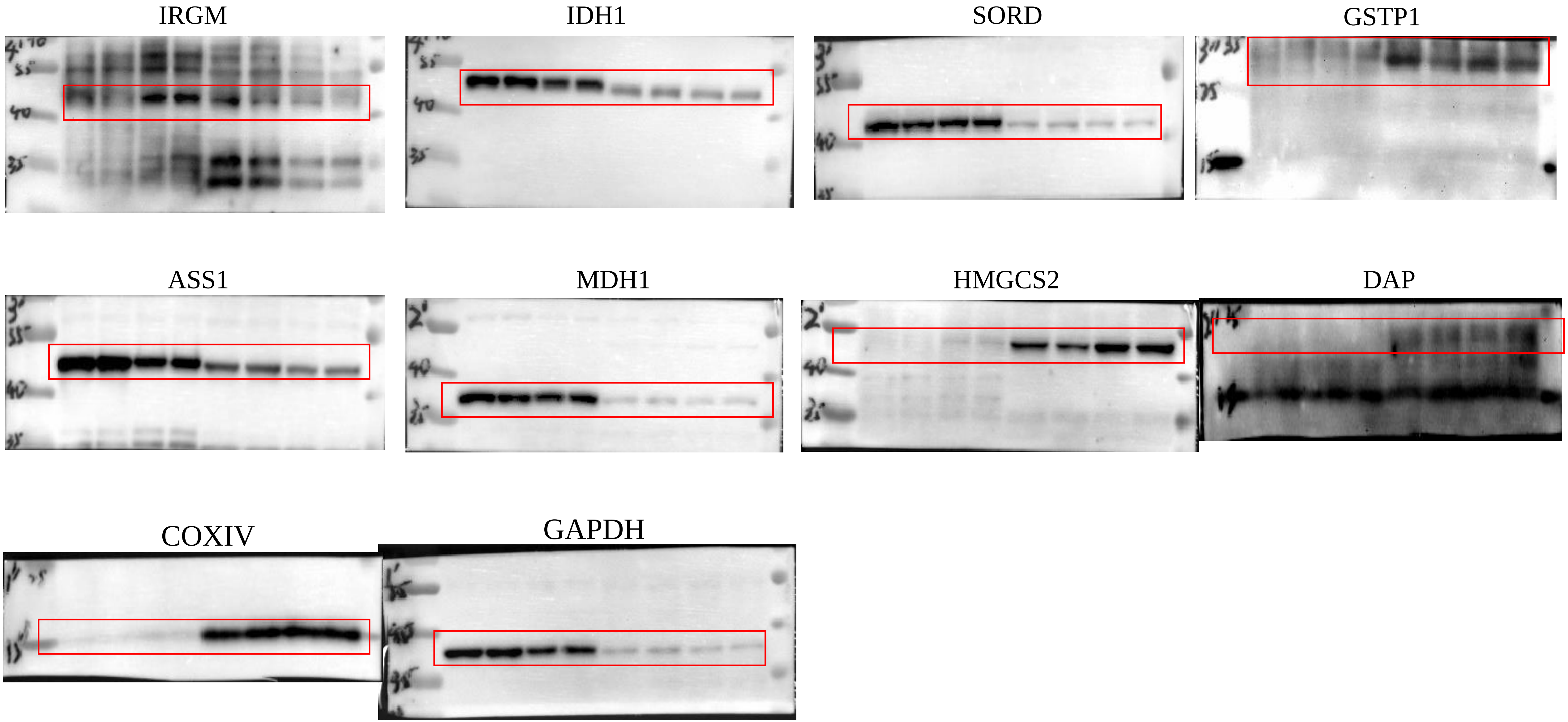

Fig 5D

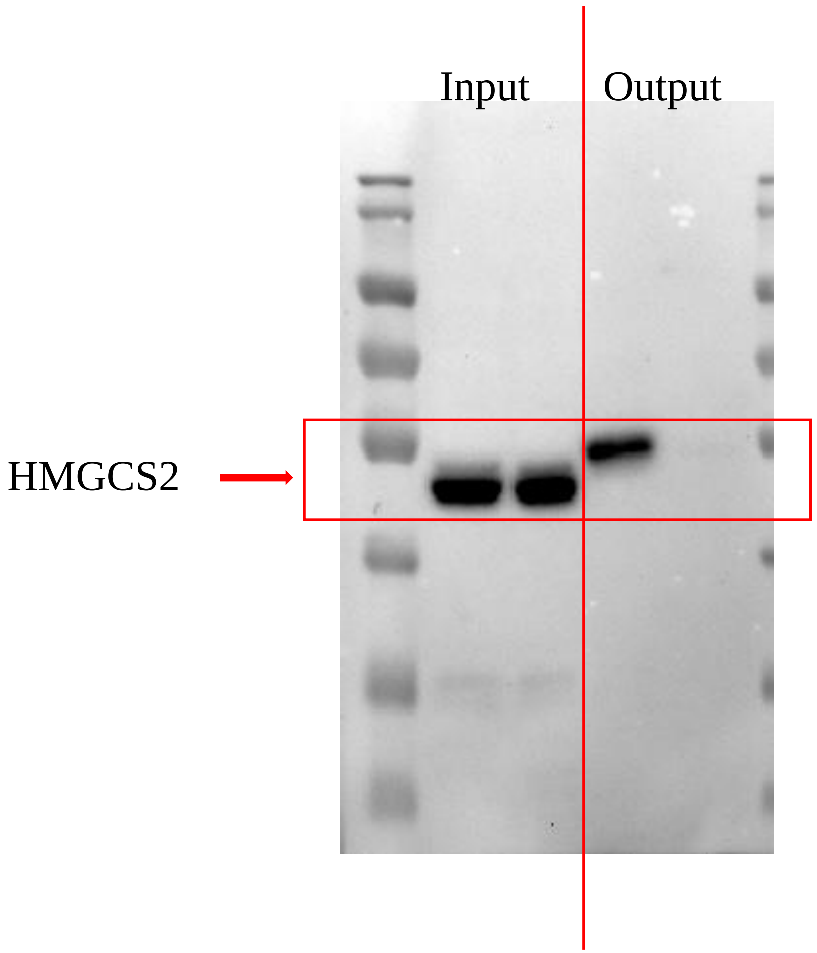


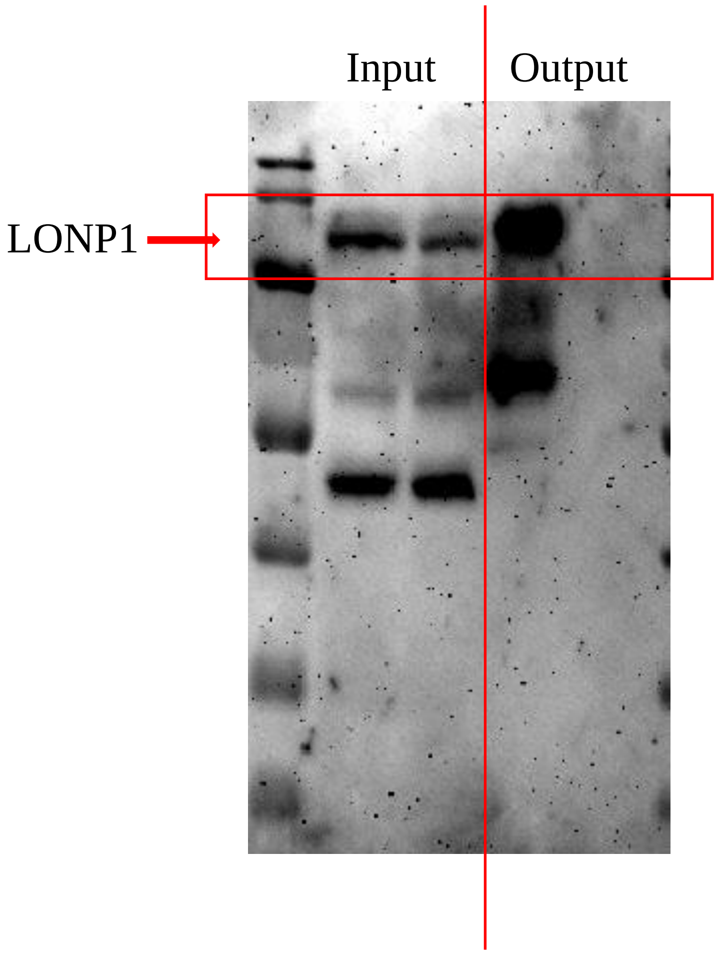

Fig 5E

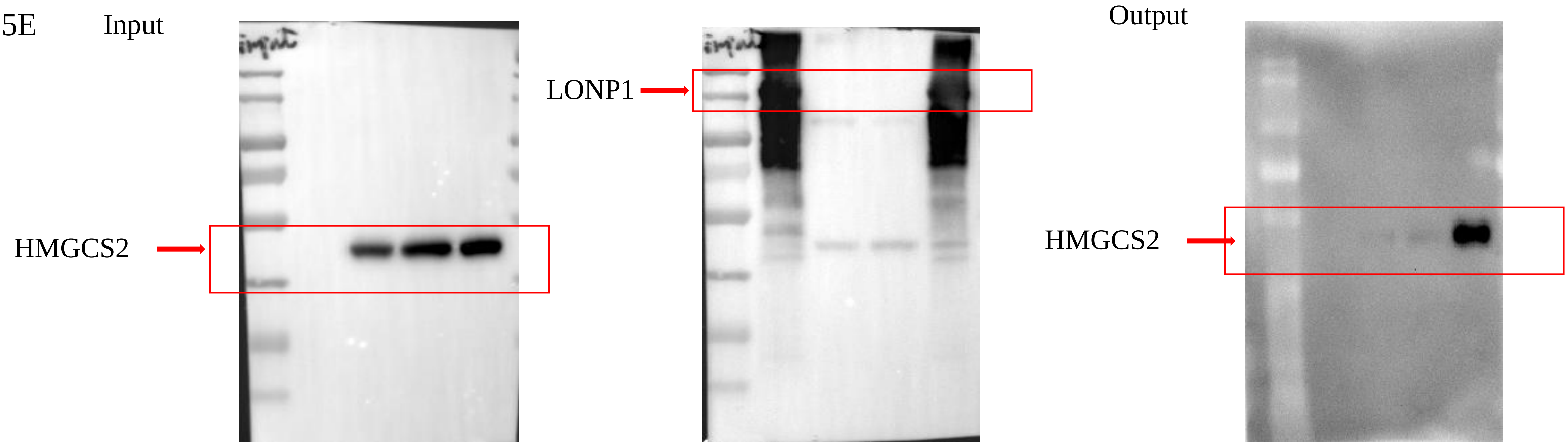


Fig 5F

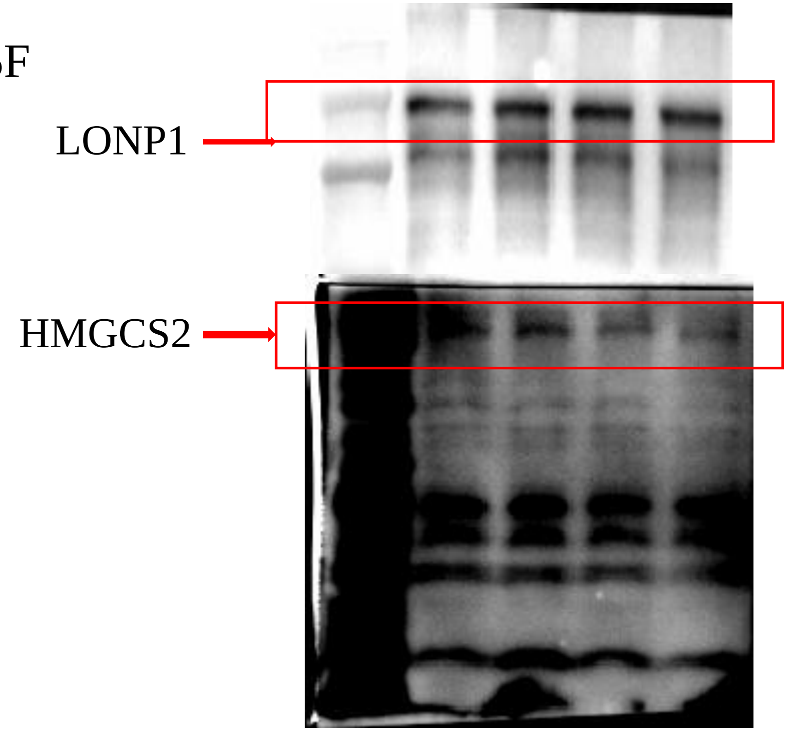

Supplement: Supplementary file 11 — Source Data for Figure 5 [file EMMM-15-e16581-s013.zip › Figure 5/5C-5F/western gel.pdf]
