# Supplementary material for: LONP1 targets HMGCS2 to protect mitochondrial function and attenuate chronic kidney disease
Source: EMBO Mol Med. 2023 Jan 11;15(2):e16581. doi: 10.15252/emmm.202216581 (PMC9906428; doi:10.15252/emmm.202216581)
Supplement: Supplementary file 15 [file EMMM-15-e16581-s002.pdf]

EMBO Molecular Medicine

LONP1 Targets HMGCS2 to Protect Mitochondrial Function and Attenuate Chronic Kidney Disease

Mi Bai, Mengqiu Wu, Mingzhu Jiang, Jia He, Xu Deng, Shuang Xu, Jiaojiao Fan, Mengqiu Miao, Ting Wang, Yuting Li, Xiaowen Yu, Lin Wang, Yue Zhang, Songming Huang, Li Yang, Zhanjun Jia, and Aihua Zhang
DOI: 10.15252/emmm.202216581

Corresponding authors: Aihua Zhang (zhaihua@njmu.edu.cn), Zhanjun Jia (jiazj72@hotmail.com)

Review Timeline:

Submission Date:	21st Jul 22
Editorial Decision:	18th Aug 22
Revision Received:	16th Nov 22
Editorial Decision:	7th Dec 22
Revision Received:	12th Dec 22
Accepted:	13th Dec 22

Editor: Zeljko Durdevic

Transaction Report:

(Note: With the exception of the correction of typographical or spelling errors that could be a source of ambiguity, letters and reports are not edited. Depending on transfer agreements, referee reports obtained elsewhere may or may not be included in this compilation. Referee reports are anonymous unless the Referee chooses to sign their reports.)

18th Aug 2022

Dear Dr. Zhang,

Thank you for the submission of your manuscript to EMBO Molecular Medicine. We have now received feedback from the two reviewers who agreed to evaluate your manuscript. As you will see from the reports, both referees recognize potential interest of the study but also raise serious concerns that should be addressed in a major revision. During our cross-commenting session it became clear that physical interaction between 84-B10 and LONP1 should be experimentally shown e.g., by performing Surface Plasmon Resonance.

Further consideration of a revision that addresses reviewers' concerns in full will entail a second round of review. EMBO Molecular Medicine encourages a single round of revision only and therefore, acceptance or rejection of the manuscript will depend on the completeness of your responses included in the next, final version of the manuscript. For this reason, and to save you from any frustrations in the end, I would strongly advise against returning an incomplete revision.

We would welcome the submission of a revised version within three months for further consideration. Please let us know if you require longer to complete the revision.

Please use this link to login to the manuscript system and submit your revision: https://embomolmed.msubmit.net/cgi-bin/main.plex

I look forward to receiving your revised manuscript.

Yours sincerely,

Zeljko Durdevic

Zeljko Durdevic
Editor
EMBO Molecular Medicine

***** Reviewer's comments *****

Referee #1 (Comments on Novelty/Model System for Author):

Injecting 2ml of plasmid DNA into a 25 g mouse via the tail vein within 10 seconds may lead to ethical concerns

Referee #1 (Remarks for Author):

In this study, data are presented to suggest a mechanistic pathway involving tubular cell metabolism, transforming growth factor beta 1 (TGF b1) signaling and kidney fibrosis. The results and conclusions support the notion that preservation of renal tubular cell mitochondrial function can block or slow the prognosis of renal fibrosis. Although there is no striking conceptual advance in this study, it demonstrated, using both genetic and pharmacologic approaches, a new pathway which links LONP1 to HMGCS2 regulating the fibrotic gene expression in tubular cells. Considering that the major contributor to kidney scaring is renal fibroblast, the authors should test the pathway in these cells to support the conclusion. In addition, it would be helpful if more unbiased approaches, (e.g., bulk and single cell transcriptome, metabolomics) were used to support the phenotypes they observed; this would provide a stronger rationale for their experimental proposal.
SPECIFIC COMMENTS

1. Figure1: Does downregulation of LONP1 occur at the transcriptional level, or is it due to loss of tubules? Transcriptome analyses, especially at single cell resolution, are needed to confirm these observations. The authors can refer to available datasets, such as E-MTAB-2502 (Nature Medicine Vol.21, pages37-46, 2015) and GSE145053 (JASN Vol. 31, Issue 12, pages 2833-2854,2020). To fully assess the impact of LONP1 on renal metabolism, the authors should profile the changes of mitochondrial genes in tubular cells.
2. Figure 2: EM fields of view are notoriously heterogeneous and selective. Please show additional, low magnification images including an intact tubular cell.
3. Figure 3: In 5/6 nephrectomy, fibrosis usually companied with glomerular hyper filtration and sclerosis. Please show a low magnification picture to exclude the edge fibrosis area caused by chopping. Please show low magnification images of tubular mitochondria.
Supplemental Figure S3A: the authors mentioned in Method: "injected 2ml of the plasmids to the mice through the tail vein via high-throughput within 10 seconds". At week 8, the mice weighed approximately 25 g and had a blood volume of approximately 5.6% of their body weight (based on the JAX laboratory database), so the total blood volume of the experimental mice was approximately 1.5 ml. Did this protocol cause any problem for mice?
4. Figure 4: the authors showed that LONP1 overexpression in HK2 cells increased the spare respiratory capacity upon TGFb1 treatment. It tends to stand alone as descriptive observations that are not clearly linked mechanistically with suppression of FN, Col, SMA mRNA. Did overexpression of LONP1 also suppress TGFb1 production in vivo?
5. Figure 5: Can the authors provide a list of known substrates of LONP1? How many substrate proteins are present in the proteomics results? Metabolomic profiling or targeted metabolome analysis should be added here to corroborate the results of proteomics.
6. Figure 6: interveinal delivery of DNA plasmid usually fail to archive the desired results; coupled with the problematic "tail vein hyperbaric injection" protocol, this reviewer suggests to perform bioluminescence imaging to verify the distribution of DNA plasmids.
7. Figure 7: What is the half-life and distribution of 84-B10 in the mouse body?
In current mouse models of kidney fibrosis, production of matrix proteins by damaged tubular cells is limited. Whereas, TGF-beta1 stimulated the activation/transformation of interstitial fibroblasts contributing largely to matrix production and fibrosis. Relevant key experiments should be repeated using fibroblasts.
8. Figure 8: did 84-B10 decrease beta-oxidation in tubules of both Sham and UIRI mice?
9. Please proofread the entire article carefully as some sentences are misleading, for example: "To ascertain the role of LONP1

in CKD, we generated proximal tubule Lonp1 conditional knock-in mice (cKI) that had approximately two-fold more proximal tubular cells, compared with those of the WT mice (Supplemental Figure S1A and B).
"Firstly, we confirmed EGFP protein expression by fluorescence microscopy were mainly in renal tubules after being overexpressed in the tail vein (Supplemental Figure S3A)".

Referee #2 (Remarks for Author):

In this study, the authors investigated the role of Lon protease 1 (LONP1), a major mitochondrial protease, in CKD. By using human biopsies from patients suffering from CKD, and the UUO and 5/6Nx models of experimental nephropathy, as well as in vitro experiments in tubular cells, they demonstrated that downregulation of LONP1, disrupted mitochondrial function and further promoted CKD progression, whereas its overexpression alleviated the injury. Furthermore, they tested a LONP1 activator, identified by computer virtual screening, that attenuated renal fibrosis, and mitochondrial dysfunction, improving thus CKD progression. Some of the provided pictures are impressive and the paper is very well designed. The manuscript could be strengthened with some additional work.

1- My main concern is the quantification of fibrotic areas using Masson's trichome. This is definitely not appropriate! The authors have to use Sirius Red coloration. I have no doubts about the result as it is clearly demonstrated by the provided WB, that fibrotic markers are increased once LONP1 expression is decreased, and these markers are decreased once LONP1 is overexpressed by tubular cells.

2- In figures 2C, and 8I, the provided WB for fibronectin are not acceptable. Please show better pictures.

3- In Fig5C, there are two bands for GAPDH.... Please explain.

4- In fig 8G there is a problem with the Masson's coloration.

5- The authors used two models of experiment nephropathy, the UUO and 5/6Nx, but to test the 84-B10 activator they used the UUO and the unilateral ischemia-reperfusion injury (UIRI) model of acute injury. What is the reason for not using the 5/6Nx model? By the way, both low and high doses of 84-B10, do not seem to ameliorate renal function in the UIRI model (figS9). Arguments on this point are welcome.

6- Is there a correlation between LONP1 decreased expression and renal function in the patient data?

Responses to the Editor:

Physical interaction between 84-B10 and LONP1 should be experimentally shown e.g., by performing Surface Plasmon Resonance.

Response: We thank the Editor for pointing this out, and have quantified the binding affinity of 84-B10 to recombinant LONP1 protein experimentally by Surface Plasmon Resonance (SPR). LONP1 was diluted to 20 µg/mL and immobilized on a CM5 sensor chip. The binding affinity of 84-B10 (0.3125, 0.625, 1.25, 2.5, 5 and 10 µM in PBS/0.01% Tween 20 buffer) to LONP1 was determined using the Biacore T200 SPR biosensor systems. Result revealed reliable binding activity between 84-B10 and LONP1, with the binding affinities (KD value) calculated as 312.5 nM (Fig 7E).

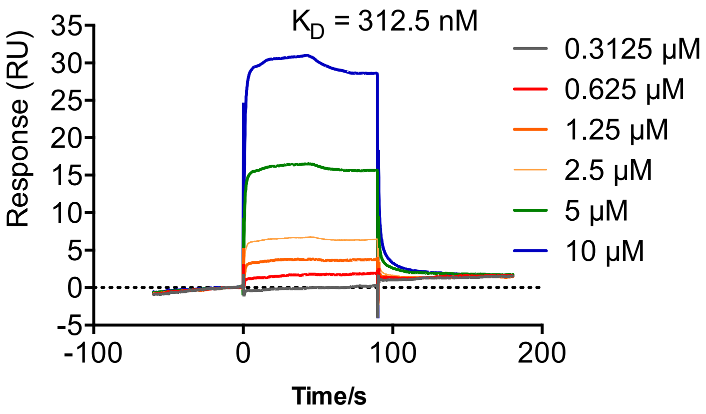

Responses to the Referees:

Responses to the Referee #1:

Injecting 2ml of plasmid DNA into a 25 g mouse via the tail vein within 10 seconds may lead to ethical concerns.

Response: Thanks for this important comment. Hydrodynamic injection in mice is a method to rapidly inject a large volume of plasmid DNA solution into mice through the tail vein of mice to obtain transgenic expression. It has been reported that when the injection volume accounts for 8%~10% of the body mass of mice, the expression efficacy of transferred genes is the best (PMID: 10455434, Gene Ther. 1999 Jul; 6(7): 1258-66). We and other groups have also used this technique to overexpress genes in the kidney (PMID: 32404507, 31318148, 23559584, 15466268 et al). After the

injection, the animal may suffer some bad stress. In our and other groups, this kind of studies was approved by the ethics committees of institutions. Our ethics number is 2007001-7 and 2102005-1, which was approved by the Institutional Animal Care and Use Committee of Nanjing Medical University. However, as you mentioned, we cannot completely exclude the potential ethical problems. During the review period, we bred and obtained Hmgcs2 heterozygous knockout mice to perform the UUO model, and found that renal fibrosis and mitochondrial function were significantly improved (Figure 6H-P). This is consistent with the findings of aggravation of renal fibrosis by overexpressing Hmgcs2 via rapid tail vein injection. Therefore, to avoid the potential ethical concerns, we included the data of knockout mice instead of tail vein injections in our revised MS. Thanks a lot for your important comments for pointing out this potential issue.

In this study, data are presented to suggest a mechanistic pathway involving tubular cell metabolism, transforming growth factor beta 1 (TGF b1) signaling and kidney fibrosis. The results and conclusions support the notion that preservation of renal tubular cell mitochondrial function can block or slow the prognosis of renal fibrosis. Although there is no striking conceptual advance in this study, it demonstrated, using both genetic and pharmacologic approaches, a new pathway which links LONP1 to HMGCS2 regulating the fibrotic gene expression in tubular cells. Considering that the major contributor to kidney scaring is renal fibroblast, the authors should test the pathway in these cells to support the conclusion. In addition, it would be helpful if more unbiased approaches, (e.g., bulk and single cell transcriptome, metabolomics) were used to support the phenotypes they observed; this would provide a stronger rationale for their experimental proposal.

Response: Thanks very much for your support on our study. Following your valuable comments, we analyzed the *Lonp1* mRNA expression in online datasets and our UUO model and found the expression of *Lonp1* was both decreased. We performed untargeted metabolomic analyses of UUO models in WT and Lonp1 cKI mice to

support our results. We also found that tubular LONP1 silencing promoted fibroblast activation, and 84-B10 inhibited TGF-β1-induced fibroblast activation. The plasma pharmacokinetics (PK) and tissue distribution of 84-B10 in mice were evaluated by HPLC coupled with tandem mass spectrometric detection (LC-MS/MS). All the other questions you mentioned have been added to the revised version.

SPECIFIC COMMENTS

1. Figure1: Does downregulation of LONP1 occur at the transcriptional level, or is it due to loss of tubules? Transcriptome analyses, especially at single cell resolution, are needed to confirm these observations. The authors can refer to available datasets, such as E-MTAB-2502 (Nature Medicine Vol.21, pages37-46, 2015) and GSE145053 (JASN Vol. 31, Issue 12, pages 2833-2854,2020). To fully assess the impact of LONP1 on renal metabolism, the authors should profile the changes of mitochondrial genes in tubular cells.

Response: We sincerely thank the reviewer for the valuable comment. Following your suggestion, we analyzed the expression of *Lonp1* in the datasets you mentioned. Consistent with our results, we found that *Lonp1* expression was significantly reduced in CKD patients in the E-MTAB-2502 dataset (Figure EV1A). The mRNA expression of *Lonp1* was also decreased in UUO-7d and R-UUO-4w models in GSE145053 dataset (Figure EV1B), as well as in our UUO models (Figure EV1C). Meanwhile, we analyzed the expression of *Lonp1* in single-cell sequencing data (Gene Atlas of Reversible Unilateral Ureteric Obstruction Model) and found that *Lonp1* was more expressed in the renal tubules and decreased after the UUO model (Figure EV1D). According to the analysis of single cell sequencing results, the gene expression is standardized, representing the expression amount of single renal tubular cells. Therefore, the decrease of LONP1 is not caused by the loss of tubules. Combined with our immunohistochemical results (Figure 1A), we also found that the expression of LONP1 was reduced in renal tubules and tubular cells. Thus, the expression of tubular Lonp1 was reduced under CKD condition independent of tubular loss.

According to your opinion, we detected the expression of mitochondrial genes in the UUO model of Lonp1 cKI mice and found that the expression of almost all mitochondrial genes was decreased in the UUO model, and some genes were up-regulated after Lonp1 overexpression, such as *Nd4*, *Nd4l*, *Nd6*, *Co3*, *Atp6*, while some genes were not differentially affected, such as *Nd1*, *Nd2*, *Nd5*, *Co1*, *Co2* and *Atp8* (Figure EV1H and I).

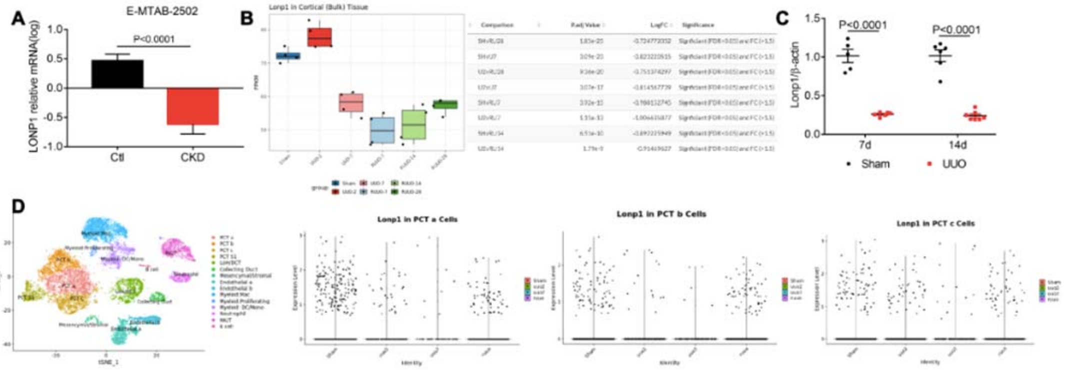

Figure EV1. *Lonp1* expression in database.

A *Lonp1* expression in online human RNA sequencing data (E-MTAB-2502, Nat Med. 2015 Jan;21(1):37-46).

B *Lonp1* expression in online mouse UUO model RNA sequencing data (GSE145053, J Am Soc Nephrol. 2020 Dec; 31(12): 2833–2854).

C The mRNA expression of *Lonp1* in our UUO model (n=5-7, biological replicates).

D *Lonp1* expression in kidney single cell datasets Gene Atlas of Reversible Unilateral Ureteric Obstruction Model (http://www.ruuo-kidney-gene-atlas.com/).

Data information: Data are presented as mean ± SEM. Student's t-test.

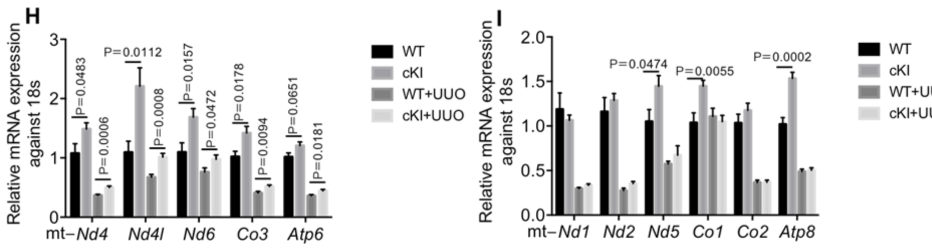

Figure EV1H and I. Mitochondrial genes expression in UUO model of Lonp1 cKI mice.

H qRT-PCR analysis of mitochondrial genes (*Nd4, Nd4l, Nd6, Co3 and Atp6*) in WT and cKI mice

after UUO (n=8 in each group, biological replicates).

I qRT-PCR analysis of mitochondrial genes (*Nd1, Nd2, Nd5, Co1, Co2 and Atp8*) in WT and cKI mice after UUO (n=8 in each group, biological replicates).

2. Figure 2: EM fields of view are notoriously heterogeneous and selective. Please show additional, low magnification images including an intact tubular cell.

Response: Thank you very much for the suggestion. In the revised MS, we showed additional low magnification images (Figure EV2H). We are sorry that we did not photograph the intact renal tubule cells at that time for a clearer view of mitochondria. But we reproduced electron microscopy in UUO model of Lonp1 cKI mice and photographed intact renal tubule cells and mitochondria (Figure EV1J). We hope these additional data could address this comment.

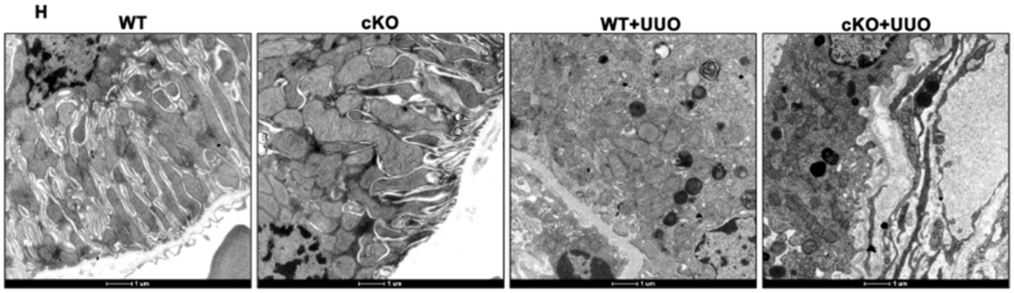

Figure EV2H. Transmission electron microscopy images of the mitochondria in tubular cells. Scale bar: 1μm.

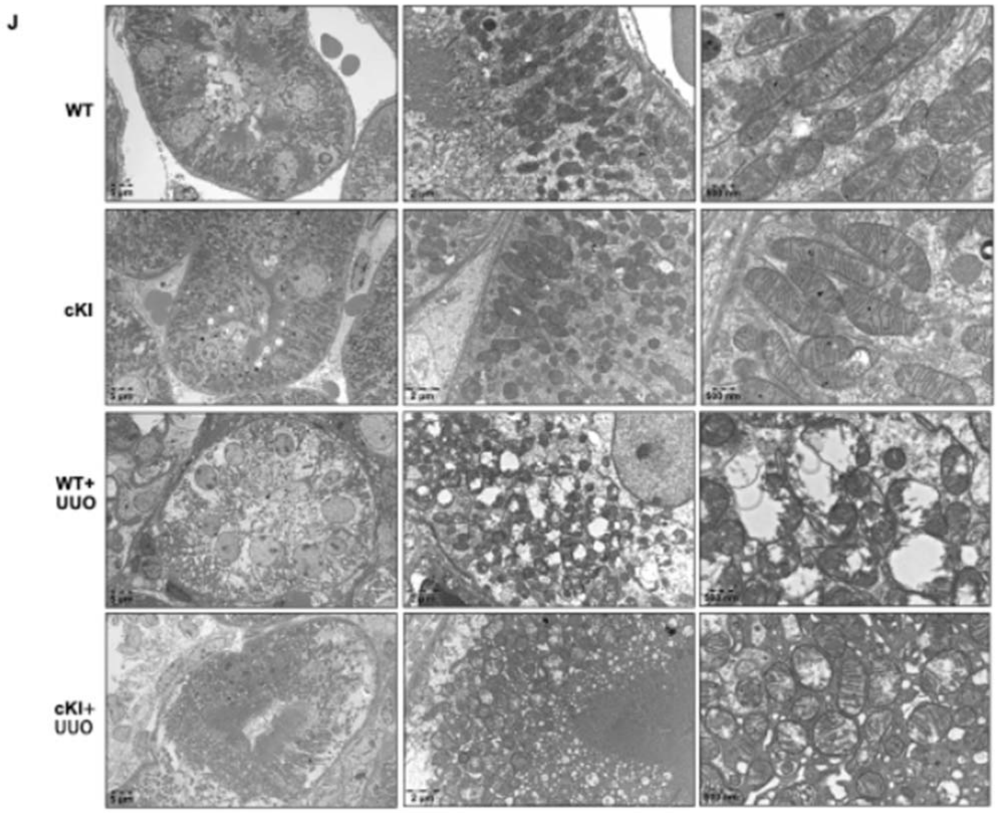

Figure EV1J. Transmission electron microscopy images of intact renal tubule cells and mitochondria. Scale bar: 5µm, 2µm and 500nm.

3. Figure 3: In 5/6 nephrectomy, fibrosis usually companied with glomerular hyper filtration and sclerosis. Please show a low magnification picture to exclude the edge fibrosis area caused by chopping. Please show low magnification images of tubular mitochondria.

Response: Thanks for the suggestion. In the revised MS, we replaced the Masson staining with low magnification images (Figure 3C and G). The additional low magnification images of mitochondria showed in Figure EV3E.

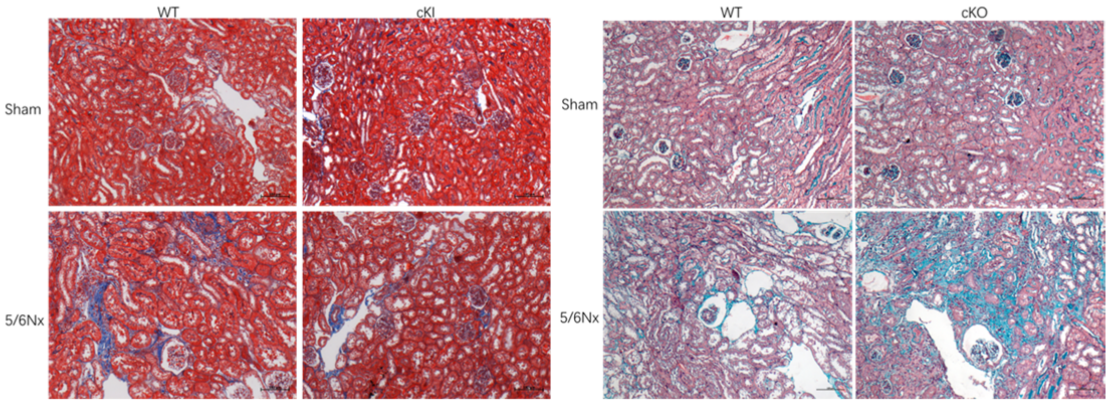

Figure 3C and G. Masson's trichrome staining. Scale bar: 100μm.

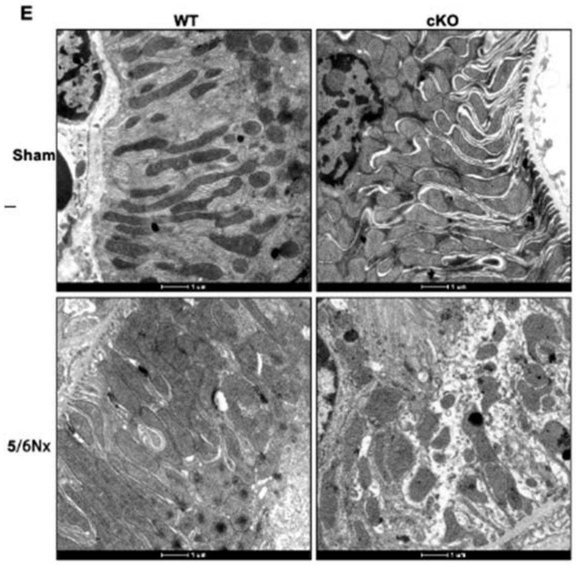

Figure EV3E. Transmission electron microscopy images of the mitochondria in tubular cells. Scale bar: 1μm.

Supplemental Figure S3A: the authors mentioned in Method: "injected 2ml of the plasmids to the mice through the tail vein via high-throughput within 10 seconds". At week 8, the mice weighed approximately 25 g and had a blood volume of approximately 5.6% of their body weight (based on the JAX laboratory database), so the total blood volume of the experimental mice was approximately 1.5 ml. Did this protocol cause any problem for mice?

Response: Thanks for this important comment. Hydrodynamic injection in mice is a method to rapidly inject a large volume of plasmid DNA solution into mice through the tail vein of mice to obtain transgenic expression. It has been reported that when the injection volume accounts for 8%~10% of the body mass of mice, the expression efficacy of transferred genes is the best (PMID: 10455434, Gene Ther. 1999 Jul; 6(7): 1258-66). We and other groups have also used this technique to overexpress genes in the kidney (PMID: 32404507, 31318148, 23559584, 15466268 et al). After the injection, the animal may suffer some bad stress. To avoid the potential ethical concerns as you mentioned, we used knockout mice instead of tail vein injections in our revised MS.

4. Figure 4: the authors showed that LONP1 overexpression in HK2 cells increased the spare respiratory capacity upon TGFb1 treatment. It tends to stand alone as descriptive observations that are not clearly linked mechanistically with suppression of FN, Col, SMA mRNA. Did overexpression of LONP1 also suppress TGFb1 production in vivo?

Response: Thank you very much for raising this important issue. According to your opinion, we detected the expression of TGF-β1 in the UUO model of Lonp1 cKI mice and found that Lonp1 overexpression inhibited the expression of TGF-β1. Therefore, Lonp1 may suppress the marker of fibrosis (FN1, Col1 and α-SMA) by affecting TGF-β1. We have added this result in the revised MS (Figure 1J).

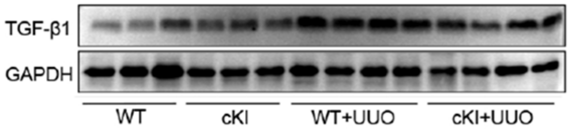

5. Figure 5: Can the authors provide a list of known substrates of LONP1? How many substrate proteins are present in the proteomics results? Metabolomic profiling or targeted metabolome analysis should be added here to corroborate the results of proteomics.

Response: Thank you very much for the suggestion. The known substrates of LONP1 are as follows: aconitase (Aco2), cytochrome c oxidase subunit 4 isoform 1 (Cox4i1), steroidogenic acute regulatory protein (Star), succinate dehydrogenase subunit 5 (Sdhaf2), transcription factor A (Tfam) and glutaminase C (Gls), as well as some heme-related enzymes such as cystathionine β-synthase (Cbs), heme oxygenase 1 (Hmox1) and 5-aminolevulinate synthase (Alas1). Most of the substrates were included in the proteomics results, as shown in Figure EV4A, but the differences in expression of these proteins were very small or non-significant between the WT and cKO groups, whereas the Hmgcs2 was significantly different between both groups.

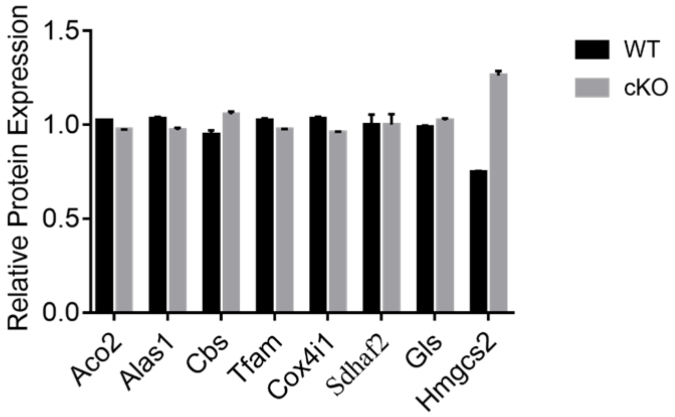

Figure EV4A. The expression of known substrates of LONP1 in our proteomics results.

We performed untargeted metabolomic analyses after UUO models in WT and Lonp1 cKI mice. The differential metabolites screening conditions are as follows: 1) VIP of the first two principal components of the PLS-DA model ≥ 1, 2) Fold-Change ≥ 1.2 or ≤ 0.83, 3) p-value <0.05. Under Positive model, there were 50 differential metabolites, among which 36 were up regulated and 14 were down regulated (Figure A). The enrichment analysis of differential metabolite metabolism pathway based on KEGG database found that pyrimidine metabolism was the most enriched pathway (Figure B), and its products Hypoxanthine, Inosine, Guanosine and Guanine were increased in the cKI-UUO group (Figure C), indicating increased AMP and GMP

catabolism (Figure D). It has been reported that the catabolism of AMP and GMP is decreased in diabetic nephropathy mouse models (PMID: 34900545, Acta Pharm Sin B. 2021 Nov; 11(11): 3665-3677), which may indicate that the increased catabolism of AMP and GMP is related to the improvement of renal injury. In our WT and Lonp1 cKO proteomics results, we found decreased expression of Enpp1 (Ectonucleotide pyrophosphatase/phosphodiesterase family member 1) and Gucy2e (Guanylyl cyclase GC-E), enzymes related to ATP and GTP hydrolysis, and increased expression of enzymes that inhibit the catabolism of AMP and GMP, such as Hprt1 (Hypoxanthine-guanine phosphoribosyltransferase), Adk (Adenosine kinase), Nme1 (Nucleoside diphosphate kinase A) and Nme2 (Nucleoside diphosphate kinase B) (Figure E). These data may indicate reduced catabolism of AMP and GMP in the Lonp1 cKO group, which is consistent with our metabolomics results.

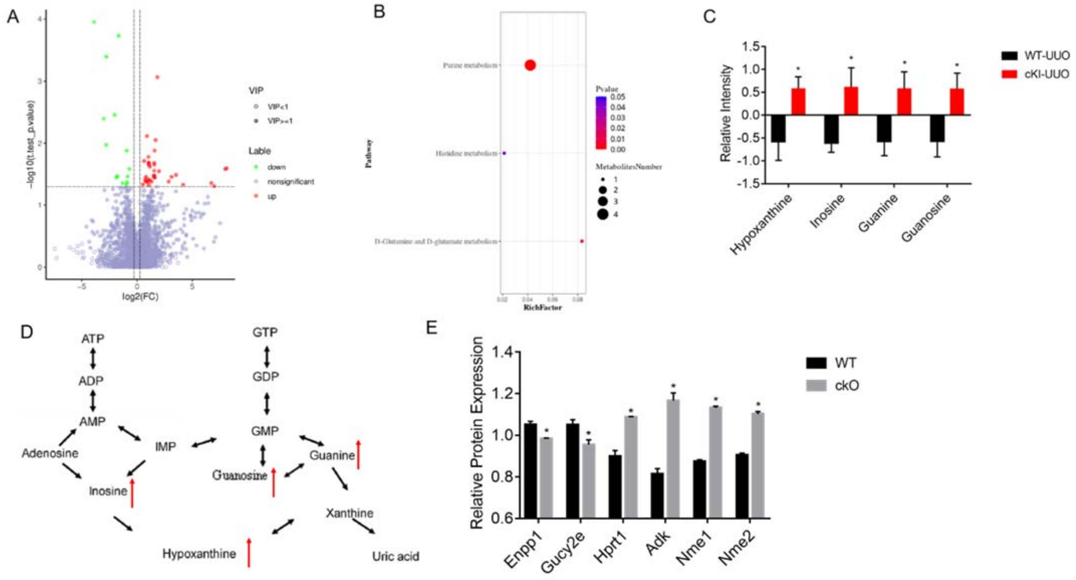

Figure (A) Volcano map of differential metabolites. Green is the down-regulated differential metabolite (labeled green), red is the up-regulated differential metabolite (labeled red), and metabolites without difference are labeled purple-gray. **(B)** Bubble plots for metabolic pathway enrichment analysis. **(C)** The levels of pyrimidine metabolism products hypoxanthine, inosine, guanosine and in WT-UUO and cKI-UUO groups. **(D)** Diagram of pyrimidine metabolism. **(E)** Expression of pyrimidine metabolism-related enzymes in our proteomic data.

6. Figure 6: interveinal delivery of DNA plasmid usually fails to archive the desired results; coupled with the problematic "tail vein hyperbaric injection" protocol, this reviewer suggests to perform bioluminescence imaging to verify the distribution of DNA plasmids.

Response: Thanks for the suggestion. During the review period, we bred and obtained Hmgcs2 heterozygous knockout mice to perform the UUO model and the result is consistent with the findings by overexpressing Hmgcs2 via rapid tail vein injection. Although the method of tail vein injection has been reported in many literatures, in order to avoid the ethical issues, we used Hmgcs2 knockout mice instead of tail vein injection in the revised MS, thus, we did not do bioluminescence experiment due to the deletion of tail vein injection data.

7. Figure 7: What is the half-life and distribution of 84-B10 in the mouse body?

Response: We appreciate the reviewer's important suggestion, and have evaluated the plasma pharmacokinetics (PK) and tissue distribution of 84-B10 in mice by HPLC coupled with tandem mass spectrometric detection (LC-MS/MS). Detailed methodology was stated in the method part. The plasma-concentration versus time profile (including PK parameters) of 84-B10 was shown in Figure EV5A, and the calculated PK parameters were: T1/2, 2.16 h; Tmax, 0.25 h; Cmax, 4523.0168 nM; AUC0-inf 4653.7194 (h × nmol/L). Tissue concentration of 84-B10 was estimated 30 min after 84-B10 injection (5 mg/kg). As shown in Figure EV5B, 84-B10 was mainly distributed in small intestine (91.34 ± 18.68 nM/g tissue weight), liver (41.68 ± 2.09 nM/g tissue weight) and kidney (24.32 ± 1.72 nM/g tissue weight).

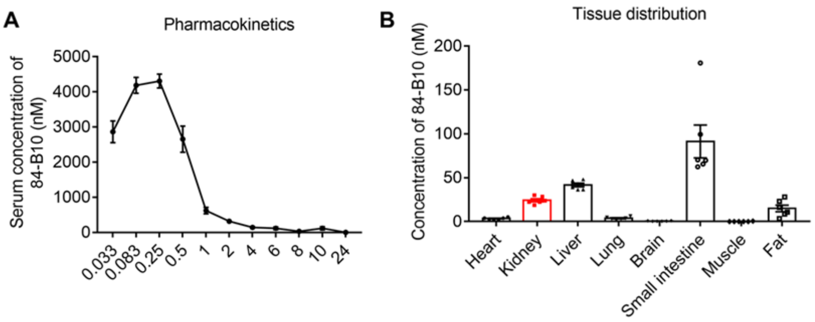

Figure EV5A and B. Plasma pharmacokinetics (PK) and tissue distribution of 84-B10 in mice.

In current mouse models of kidney fibrosis, production of matrix proteins by damaged tubular cells is limited. Whereas, TGF-beta1 stimulated the activation/transformation of interstitial fibroblasts contributing largely to matrix production and fibrosis. Relevant key experiments should be repeated using fibroblasts.

<u>Response:</u> We agree with the reviewer's suggestions and evaluated the influence of 84-B10 on rat kidney fibroblasts NRK-49F. It is known that injured renal tubules can secrete various factors to act on fibroblasts and promote their activation. Therefore, we interfered LONP1 with shRNA in renal tubular cells for 24 hours and then collected the supernatant to stimulate NRK-49F cells and found that it could promote the activation of fibroblasts (evidenced by increased expression of FN1 and Collagen III) (Appendix Fig S1A-D). We also examined the direct action of 84-B10 on NRK-49F cells. As shown in Appendix Fig S1E-G, TGF-β1 stimulated the activation of NRK-49F cells (evidenced by increased expression of FN1 and α-SMA), which was dose dependently inhibited by 84-B10.

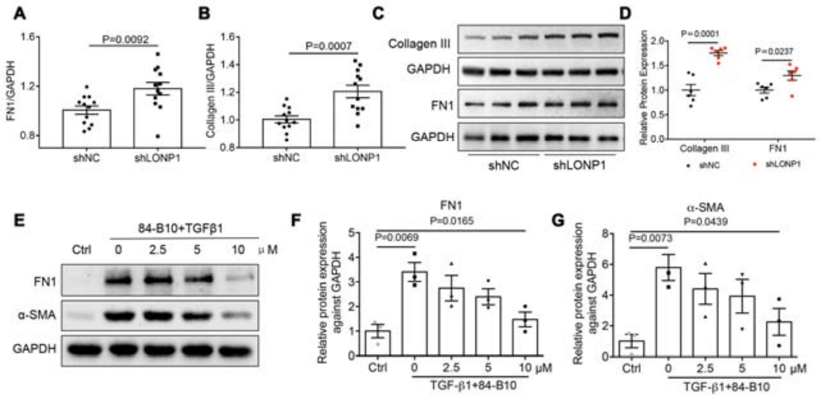

Appendix Figure S1. The effects of tubular LONP1 and 84-B10 on the NRK-49F cells.

A, B qRT-PCR analysis of FN1 and Collagen III transcript levels in NRK-49F cells. After interfering LONP1 with shRNA in mPTC for 24 h, the supernatant was collected to stimulate NRK-49F cells for 24 h. Two independent experiments were carried out (n=12 in each group, biological replicates).

C, D Representative immunoblot and densitometric analysis of FN1 and Collagen III in NRK-49F

cells treated as indicated. Two independent experiments were carried out (n=6 in each group, biological replicates).

E-G Representative immunoblot and densitometric analysis of FN1 and α-SMA in NRK-49F cells treated as indicated. Three independent experiments were carried out and quantification of the abundance of these proteins is shown in panel (n=3 in each group, biological replicates).

Data information: Data are presented as mean ± SEM. Student's t-test.

8. Figure 8: did 84-B10 decrease beta-oxidation in tubules of both Sham and UIRI mice?

Response: Thank you very much for the suggestion. We estimated the effect of 84-B10 on fatty acid β-oxidation (FAO) of both Sham and UIRI mice by estimating the renal expression levels of key enzymes ACOX1 and ACADM. Relative quantification analysis showed that UIRI markedly decreased renal ACOX1 and ACADM expression, while 84-B10 significantly elevated the expression of both proteins, suggesting an effect of 84-B10 in promoting FAO in UIRI kidney. Since FAO primarily takes place in mitochondria, restored FAO level could indicate an improved mitochondrial function. These results were shown as Appendix Fig S3.

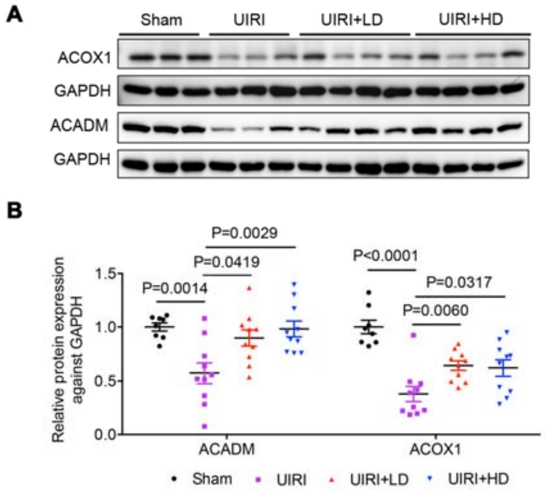

Appendix Figure S3. The effect of 84-B10 on fatty acid β-oxidation (FAO) in UIRI model.

A Representative Western blot images of ACADM and ACOX1 in UIRI model following 84-B10 treatment.

B Densitometric analysis of ACADM and ACOX1 protein levels in UIRI model following 84-B10

treatment (n=8 in Sham group, n=10 in the other three groups, biological replicates).

Data information: Data are presented as mean ± SEM. Student's t-test.

9. Please proofread the entire article carefully as some sentences are misleading, for example: "To ascertain the role of LONP1 in CKD, we generated proximal tubule Lonp1 conditional knock-in mice (cKI) that had approximately two-fold more proximal tubular cells, compared with those of the WT mice (Supplemental Figure S1A and B).

"Firstly, we confirmed EGFP protein expression by fluorescence microscopy were mainly in renal tubules after being overexpressed in the tail vein (Supplemental Figure S3A)".

Response: We apologize for the unclear description. In the revised MS, we carefully proofread the article. We replaced the sentence "To ascertain the role of LONP1 in CKD, we generated proximal tubule Lonp1 conditional knock-in mice (cKI)…" with "To ascertain the role of LONP1 in CKD, we generated proximal tubule Lonp1 conditional knock-in mice (cKI). Compared with wild-type (WT) mice, the expression of LONP1 in renal tubule cells of cKI mice was approximately doubled (Fig EV1G)". The other sentence "Next, we generated proximal tubule Lonp1 conditional-knockout (cKO) mice with an approximate 100% reduction in cultured primary proximal tubular cells, compared with those of WT mice" was replaced with "Next, we generated proximal tubule *Lonp1* conditional knockout (cKO) mice. Compared with WT mice, LONP1 was almost not expressed in primary proximal tubule cells extracted and cultured from cKO mice". Other references to the tail vein injection have been removed from our revised article

Response to Referee #2:

In this study, the authors investigated the role of Lon protease 1 (LONP1), a major mitochondrial protease, in CKD. By using human biopsies from patients suffering from CKD, and the UUO and 5/6Nx models of experimental nephropathy, as well as

in vitro experiments in tubular cells, they demonstrated that downregulation of LONP1, disrupted mitochondrial function and further promoted CKD progression, whereas its overexpression alleviated the injury. Furthermore, they tested a LONP1 activator, identified by computer virtual screening, that attenuated renal fibrosis, and mitochondrial dysfunction, improving thus CKD progression. Some of the provided pictures are impressive and the paper is very well designed. The manuscript could be strengthened with some additional work.

Response: Thank you very much for your support on this research work. We have performed additional experiments and carefully revised the MS according to your valuable suggestions.

1- My main concern is the quantification of fibrotic areas using Masson's trichome. This is definitely not appropriate! The authors have to use Sirius Red coloration. I have no doubts about the result as it is clearly demonstrated by the provided WB, that fibrotic markers are increased once LONP1 expression is decreased, and these markers are decreased once LONP1 is overexpressed by tubular cells.

Response: Thanks very much for bringing out this important issue. In the revised MS, we re-stained Sirius red and counted fibrotic area in all animal models.

2- In figures 2C, and 8I, the provided WB for fibronectin are not acceptable. Please show better pictures.

Response: Thank you very much for the suggestion. We have replaced it with a better picture in the revised MS.

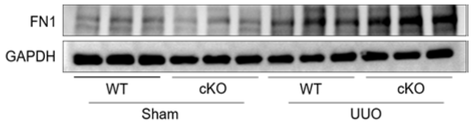

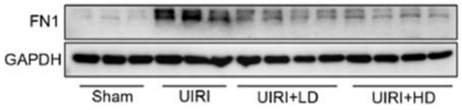

3- In Fig5C, there are two bands for GAPDH.... Please explain.

Response: Thanks for pointing out this phenomenon. The GAPDH antibody we use is a polyclonal antibody, sometimes non-specific bands may appear when the exposure time is long. We are very sorry that we didn't pay attention to this problem previously. In the revised MS we chose a picture with a shorter exposure time instead as follows.

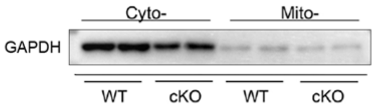

4- In fig 8G there is a problem with the Masson's coloration.

Response: We thank the reviewer for pointing this out, and have replaced Fig 8G with Sirius Red staining in the revised manuscript.

5- The authors used two models of experiment nephropathy, the UUO and 5/6Nx, but to test the 84-B10 activator they used the UUO and the unilateral ischemia-reperfusion injury (UIRI) model of acute injury. What is the reason for not using the 5/6Nx model?

Response: We appreciated the reviewer's nice suggestions. 5/6Nx model is a long-term disease model and needs large dose of drugs. Thus, we did not examine the role of 84-B10 in this model previously. In the revised MS, we identified the role of 84-B10 using the 5/6Nx mice model. Consistent with the effect of 84-B10 in UUO and UIRI model, 84-B10 also attenuated renal fibrosis in 5/6Nx mice (Figure EV5C-G). The anti-fibrosis effect of 84-B10 in different types of CKD models better indicated its potential in clinic use, as the insults leading to CKD is various in patients.

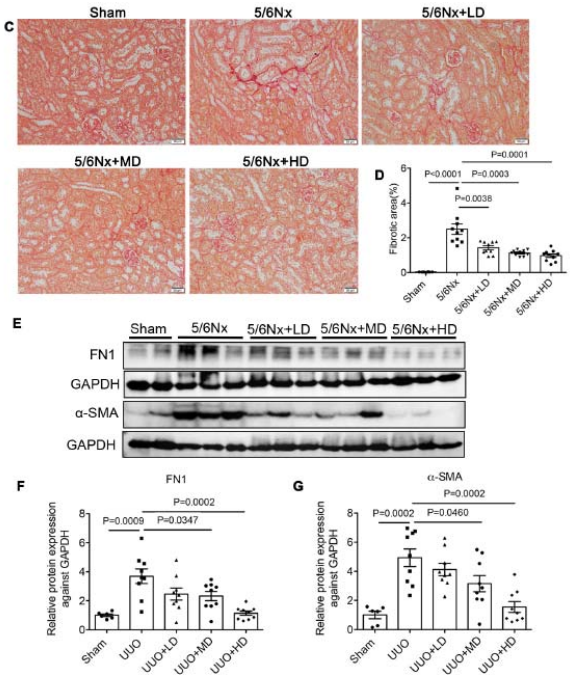

Figure EV5. The anti-fibrosis effect of 84-B10 in 5/6Nx mice

C, D Sirius red staining and fibrotic area statistics of 5/6Nx mice treated with LONP1 activator 84-B10. Scale bar: 50 μm (n=8 in WT group, n=10 in other three groups, biological replicates).

E-G Western blot and densitometric analysis for the expression of FN1 and α-SMA in 5/6Nx mice treated with 84-B10 (n=6 in WT group, n=9 in other three groups, biological replicates).

Data information: Data are presented as mean ± SEM. Student's t-test.

By the way, both low and high doses of 84-B10 do not seem to ameliorate renal function in the UIRI model (figS9). Arguments on this point are welcome.

Response: BUN and Scr are both non-protein nitrogenous waste product and the increase of BUN and Scr in blood reflected decreased renal excretion ability. Although we observed a moderate but significant increase of BUN and Scr levels in UIRI mice, the values are within normal reference ranges of C57BL/6 male mice (BUN: 7.82-13.93 mM and Scr: 4.55 to 18.18 μM) (PMID: 27423143, 14970000). Thus, the increased BUN and Scr levels might reflect a mild re-setup of renal excretion ability in UIRI model because it is known that the healthy kidney in contralateral side is enough to maintain the homeostasis of metabolism.

6- Is there a correlation between LONP1 decreased expression and renal function in the patient data?

Response: Thanks for the suggestion. We analyzed the correlation between LONP1 and BUN and Scr in CKD patients, and found that LONP1 was negatively correlated with BUN and Scr (Figure EV1E and F).

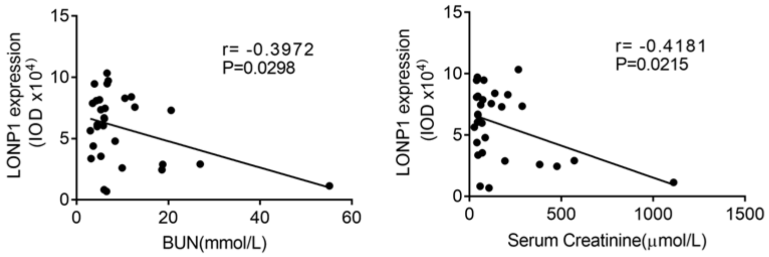

7th Dec 2022

Dear Dr. Zhang,

Thank you for the submission of your revised manuscript to EMBO Molecular Medicine. I am pleased to inform you that we will be able to accept your manuscript pending the following final amendments:

1) Please address the referee #1 minor concerns.
2) Please add Author Name, Journal Name and Manuscript Number in designated fields in the Author Checklist.
3) In the main manuscript file, please do the following:
- Correct/answer the track changes suggested by our data editors by working from the attached document.
- All Figures should be called out in a sequential order. Currently Fig. 7C is called out before Fig 7B, please correct. Also, add callouts for Fig. EV1I.
- In M&M, include a statement that informed consent was obtained from all human subjects and that the experiments conformed to the principles set out in the WMA Declaration of Helsinki and the Department of Health and Human Services Belmont Report.
- Add heading "Expanded View Figure Legends" before EV figure legends.
- Please rename "Competing Interest" to "Disclosure Statement & Competing Interests". We updated our journal's competing interests policy in January 2022 and request authors to consider both actual and perceived competing interests. Please review the policy https://www.embopress.org/competing-interests and update your competing interests if necessary.
- Author contributions: Please remove it from the manuscript and specify author contributions in our submission system. CRediT has replaced the traditional author contributions section because it offers a systematic machine-readable author contributions format that allows for more effective research assessment. You are encouraged to use the free text boxes beneath each contributing author's name to add specific details on the author's contribution. More information is available in our guide to authors:
https://www.embopress.org/page/journal/17574684/authorguide#authorshipguidelines
- Please be aware that all deposited datasets should be made freely available upon acceptance, without restriction. Please check "Author Guidelines" for more information.
https://www.embopress.org/page/journal/17574684/authorguide#availabilityofpublishedmaterial
4) EV Tables: Pease submit EV tables as separate files and remove them from the main manuscript file.
5) Appendix: Please add page numbers.
6) Synopsis:
- Synopsis image: Please resize the visual abstract to 550 px-wide x (250-400)-px high and submit as a high-resolution .jpeg file.
- Synopsis text: I have modified the synopsis text to fit the journal style. Please review it and amend as you see fit by working from the attached document.
- Please check your synopsis text and image before submission with your revised manuscript. Please be aware that in the proof stage minor corrections only are allowed (e.g., typos).
7) For more information: This space should be used to list relevant web links for further consultation by our readers. Could you identify some relevant ones and provide such information as well? Some examples are patient associations, relevant databases, OMIM/proteins/genes links, author's websites, etc...
8) As part of the EMBO Publications transparent editorial process initiative (see our Editorial at http://embomolmed.embopress.org/content/2/9/329), EMBO Molecular Medicine will publish online a Review Process File (RPF) to accompany accepted manuscripts. This file will be published in conjunction with your paper and will include the anonymous referee reports, your point-by-point response and all pertinent correspondence relating to the manuscript. Let us know whether you agree with the publication of the RPF and as here, if you want to remove or not any figures from it prior to publication. Please note that the Authors checklist will be published at the end of the RPF.
9) Please provide a point-by-point letter INCLUDING my comments as well as the reviewer's reports and your detailed responses (as Word file).

I look forward to reading a new revised version of your manuscript as soon as possible.

Yours sincerely,

Zeljko Durdevic

Zeljko Durdevic
Editor
EMBO Molecular Medicine

***** Reviewer's comments *****

Referee #1 (Remarks for Author):

This reviewer is satisfied with the professional and rigorous responses provided by the authors. However, there are two minor concerns with the revision:

1) Figure 1J: there are two forms of Tgfb1 can be detected using western blotting, latent and monomer. Authors should state which form is represented in the Figure 1J.
2) Inhibition of Hmgcs2 reportedly promoted fibrosis in liver (Hepatocyte-Macrophage Acetoacetate Shuttle Protects against Tissue Fibrosis, Cell Metabolism, Puchalska et al., 2019, Cell Metabolism 29, 383-398, February 5, 2019). Considering fibrosis is a general reparative or reactive response of organs, this reviewer suggest discussing this disagreement in Discussion section.

Responses to the Referee #1:

1) Figure 1J: there are two forms of Tgfb1 can be detected using western blotting, latent and monomer. Authors should state which form is represented in the Figure 1J.

Response: Thanks for this important comment. What we showed in Figure 1J is the monomer form of TGF-β1 (15kD), and we annotated it in the figure legends.

2) Inhibition of Hmgcs2 reportedly promoted fibrosis in liver (Hepatocyte-Macrophage Acetoacetate Shuttle Protects against Tissue Fibrosis, Cell Metabolism, Puchalska et al., 2019, Cell Metabolism 29, 383-398, February 5, 2019). Considering fibrosis is a general reparative or reactive response of organs, this reviewer suggest discussing this disagreement in Discussion section.

Response: We sincerely thank the reviewer for the valuable comment. We have discussed this disagreement in Discussion section: "It has been reported that the inhibition of HMGCS2 in the liver will cause the impairment of hepatic ketogenesis, which can promote liver fibrosis through the various factors derived from the injured hepatocytes and activation of macrophages (Mooli & Ramakrishnan, 2022; Puchalska et al, 2019). This is not consistent with our experimental results. The possible reason is that although fibrosis is a general reparative or reactive reaction in organs, the mechanisms involved in fibrosis in different diseases are not the same. HMGCS2 plays a non-ketogenic role in extrahepatic mitochondria contrasting to its ketogenic action in liver mitochondria (Puchalska & Crawford, 2017). Venable et al. found that the expression of HMGCS2 in the kidney increased under fasting conditions but did not affect the production of circulating ketone bodies, suggesting that HMGCS2 in kidney may have different functions (Venable et al, 2022). In our study, HMGCS2 promoted renal fibrosis by triggering mitochondrial dysfunction."

We are pleased to inform you that your manuscript is accepted for publication and is now being sent to our publisher to be included in the next available issue of EMBO Molecular Medicine.

EMBO Press Author Checklist

Corresponding Author Name: Aihua Zhang & Zhanjun Jia	
Journal Submitted to: EMBO Molecular Medicine	
Manuscript Number: EMM-2022-16581	

USEFUL LINKS FOR COMPLETING THIS FORM
The EMBO Journal - Author Guidelines
EMBO Reports - Author Guidelines
Molecular Systems Biology - Author Guidelines
EMBO Molecular Medicine - Author Guidelines

Reporting Checklist for Life Science Articles (updated January 2022)
This checklist is adapted from Materials Design Analysis Reporting (MDAR) Checklist for Authors. MDAR establishes a minimum set of requirements in transparent reporting in the life sciences (see Statement of Task: 10.31222/osf.io/9sm4x). Please follow the journal's guidelines in preparing your manuscript.
Please note that a copy of this checklist will be published alongside your article.

Abridged guidelines for figures
1. Data
The data shown in figures should satisfy the following conditions:
- the data were obtained and processed according to the field's best practice and are presented to reflect the results of the experiments in an accurate and unbiased manner.
- ideally, figure panels should include only measurements that are directly comparable to each other and obtained with the same assay.
- plots include clearly labeled error bars for independent experiments and sample sizes. Unless justified, error bars should not be shown for technical replicates.
- if n<5, the individual data points from each experiment should be plotted. Any statistical test employed should be justified.
- Source Data should be included to report the data underlying figures according to the guidelines set out in the authorship guidelines on Data Presentation.

2. Captions
Each figure caption should contain the following information, for each panel where they are relevant:
- a specification of the experimental system investigated (eg cell line, species name).
- the assay(s) and method(s) used to carry out the reported observations and measurements.
- an explicit mention of the biological and chemical entity(ies) that are being measured.
- an explicit mention of the biological and chemical entity(ies) that are altered/varied/perturbed in a controlled manner.
- the exact sample size (n) for each experimental group/condition, given as a number, not a range;
- a description of the sample collection allowing the reader to understand whether the samples represent technical or biological replicates (including how many animals, litters, cultures, etc.).
- a statement of how many times the experiment shown was independently replicated in the laboratory.
- definitions of statistical methods and measures:
  - common tests, such as t-test (please specify whether paired vs. unpaired), simple χ2 tests, Wilcoxon and Mann-Whitney tests, can be unambiguously identified by name only, but more complex techniques should be described in the methods section;
  - are tests one-sided or two-sided?
  - are there adjustments for multiple comparisons?
  - exact statistical test results, e.g., P values = x but not P values < x;
  - definition of 'center values' as median or average;
  - definition of error bars as s.d. or s.e.m.

Please complete ALL of the questions below.
Select "Not Applicable" only when the requested information is not relevant for your study.

Materials

Newly Created Materials	Information included in the manuscript?	In which section is the information available? (Reagents and Tools Table, Materials and Methods, Figures, Data Availability Section)
New materials and reagents need to be available; do any restrictions apply?	Not Applicable	

Antibodies	Information included in the manuscript?	In which section is the information available? (Reagents and Tools Table, Materials and Methods, Figures, Data Availability Section)
For **antibodies** provide the following information:<br>- Commercial antibodies: RRID (if possible) or supplier name, catalogue number and or/clone number<br>- Non-commercial: RRID or citation	Yes	Mateials and Methods

DNA and RNA sequences	Information included in the manuscript?	In which section is the information available? (Reagents and Tools Table, Materials and Methods, Figures, Data Availability Section)
**Short novel DNA or RNA including primers, probes**: provide the sequences.	Yes	Mateials and Methods

Cell materials	Information included in the manuscript?	In which section is the information available? (Reagents and Tools Table, Materials and Methods, Figures, Data Availability Section)
**Cell lines:** Provide species information, strain. Provide accession number in repository **OR** supplier name, catalog number, clone number, and/**OR** RRID.	Yes	Mateials and Methods
**Primary cultures:** Provide species, strain, sex of origin, genetic modification status.	Yes	Mateials and Methods
Report if the cell lines were recently **authenticated** (e.g., by STR profiling) and tested for mycoplasma contamination.	Yes	Mateials and Methods

Experimental animals	Information included in the manuscript?	In which section is the information available? (Reagents and Tools Table, Materials and Methods, Figures, Data Availability Section)
**Laboratory animals or Model organisms:** Provide species, strain, sex, age, genetic modification status. Provide accession number in repository **OR** supplier name, catalog number, clone number, **OR** RRID.	Yes	Mateials and Methods
**Animal observed in or captured from the field:** Provide species, sex, and age where possible.	Yes	Mateials and Methods
Please detail **housing and husbandry conditions**.	Yes	Mateials and Methods

Plants and microbes	Information included in the manuscript?	In which section is the information available? (Reagents and Tools Table, Materials and Methods, Figures, Data Availability Section)
**Plants:** provide species and strain, ecotype and cultivar where relevant, unique accession number if available, and source (including location for collected wild specimens).	Not Applicable	
**Microbes:** provide species and strain, unique accession number if available, and source.	Not Applicable	

Human research participants	Information included in the manuscript?	In which section is the information available? (Reagents and Tools Table, Materials and Methods, Figures, Data Availability Section)
If collected and within the bounds of privacy constraints report on age, sex and gender or ethnicity for all study participants.	Yes	Mateials and Methods

Core facilities	Information included in the manuscript?	In which section is the information available? (Reagents and Tools Table, Materials and Methods, Figures, Data Availability Section)
If your work benefited from core facilities, was their service mentioned in the acknowledgments section?	Yes	Acknowledgements

Design

Study protocol	Information included in the manuscript?	In which section is the information available? (Reagents and Tools Table, Materials and Methods, Figures, Data Availability Section)
If study protocol has been **pre-registered, provide DOI in the manuscript**. For clinical trials, provide the trial registration number **OR** cite DOI.	Yes	Matetials and Methods
Report the **clinical trial registration number** (at ClinicalTrials.gov or equivalent), where applicable.	Yes	Matetials and Methods

Laboratory protocol	Information included in the manuscript?	In which section is the information available? (Reagents and Tools Table, Materials and Methods, Figures, Data Availability Section)
Provide DOI OR other citation details if **external detailed step-by-step protocols** are available.	Not Applicable	

Experimental study design and statistics	Information included in the manuscript?	In which section is the information available? (Reagents and Tools Table, Materials and Methods, Figures, Data Availability Section)
Include a statement about **sample size** estimate even if no statistical methods were used.	Yes	Figures
Were any steps taken to minimize the effects of subjective bias when allocating animals/samples to treatment (e.g. **randomization procedure**)? If yes, have they been described?	Yes	Matetials and Methods
Include a statement about **blinding** even if no blinding was done.	Yes	Matetials and Methods
Describe **inclusion/exclusion criteria** if samples or animals were excluded from the analysis. Were the criteria pre-established?<br><br>If sample or data points were omitted from analysis, report if this was due to attrition or intentional exclusion and provide justification.	Not Applicable	
For every figure, are **statistical tests** justified as appropriate? Do the data meet the assumptions of the tests (e.g., normal distribution)? Describe any methods used to assess it. Is there an estimate of variation within each group of data? Is the variance similar between the groups that are being statistically compared?	Yes	Matetials and Methods

Sample definition and in-laboratory replication	Information included in the manuscript?	In which section is the information available? (Reagents and Tools Table, Materials and Methods, Figures, Data Availability Section)
In the figure legends: state number of times the experiment was **replicated** in laboratory.	Yes	Figures
In the figure legends: define whether data describe **technical or biological replicates**.	Yes	Figures

Ethics

Ethics	Information included in the manuscript?	In which section is the information available? (Reagents and Tools Table, Materials and Methods, Figures, Data Availability Section)
Studies involving **human participants**: State details of **authority granting ethics approval** (IRB or equivalent committee(s), provide reference number for approval.	Yes	Matetials and Methods
Studies involving **human participants**: Include a statement confirming that **informed consent** was obtained from all subjects and that the experiments conformed to the principles set out in the WMA Declaration of Helsinki and the Department of Health and Human Services Belmont Report.	Yes	Matetials and Methods
Studies involving **human participants:** For publication of **patient photos**, include a statement confirming that consent to publish was obtained.	Not Applicable	
Studies involving experimental **animals**: State details of **authority granting ethics approval** (IRB or equivalent committee(s), provide reference number for approval. Include a statement of compliance with ethical regulations.	Yes	Matetials and Methods
Studies involving **specimen and field samples:** State if relevant **permits** obtained, provide details of authority approving study; if none were required, explain why.	Not Applicable	

Dual Use Research of Concern (DURC)	Information included in the manuscript?	In which section is the information available? (Reagents and Tools Table, Materials and Methods, Figures, Data Availability Section)
Could your study fall under dual use research restrictions? Please check biosecurity documents and list of **select agents and toxins** (CDC): https://www.selectagents.gov/sat/list.htm	Not Applicable	
If you used a select agent, is the security level of the lab appropriate and reported in the manuscript?	Not Applicable	
If a study is subject to dual use research of concern regulations, is the name of the **authority granting approval and reference number** for the regulatory approval provided in the manuscript?	Not Applicable	

Reporting

The MDAR framework recommends adoption of discipline-specific guidelines, established and endorsed through community initiatives. Journals have their own policy about requiring specific guidelines and recommendations to complement MDAR.

Adherence to community standards	Information included in the manuscript?	In which section is the information available? (Reagents and Tools Table, Materials and Methods, Figures, Data Availability Section)
State if relevant guidelines or checklists (e.g., **ICMJE, MIBBI, ARRIVE, PRISMA**) have been followed or provided.	Not Applicable	
For **tumor marker prognostic studies,** we recommend that you follow the **REMARK** reporting guidelines (see link list at top right). See author guidelines, under 'Reporting Guidelines'. Please confirm you have followed these guidelines.	Not Applicable	
For **phase II and III randomized controlled trials**, please refer to the **CONSORT** flow diagram (see link list at top right) and submit the CONSORT checklist (see link list at top right) with your submission. See author guidelines, under 'Reporting Guidelines'. Please confirm you have submitted this list.	Not Applicable	

Data Availability

Data availability	Information included in the manuscript?	In which section is the information available? (Reagents and Tools Table, Materials and Methods, Figures, Data Availability Section)
Have **primary datasets** been deposited according to the journal's guidelines (see 'Data Deposition' section) and the respective accession numbers provided in the Data Availability Section?	Yes	Data Availability
Were **human clinical and genomic datasets** deposited in a public access-controlled repository in accordance to ethical obligations to the patients and to the applicable consent agreement?	Not Applicable	
Are **computational models** that are central and integral to a study available without restrictions in a machine-readable form? Were the relevant accession numbers or links provided?	Not Applicable	
If publicly available data were reused, provide the respective **data citations in the reference list**.	Yes	Reference
