# Supplementary material for: LONP1 targets HMGCS2 to protect mitochondrial function and attenuate chronic kidney disease
Source: EMBO Mol Med. 2023 Jan 11;15(2):e16581. doi: 10.15252/emmm.202216581 (PMC9906428; doi:10.15252/emmm.202216581)

Fig EV4K

HMGCS2-HA

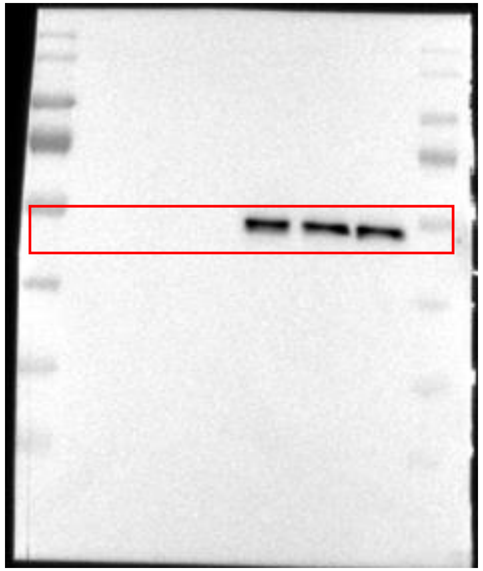

GAPDH

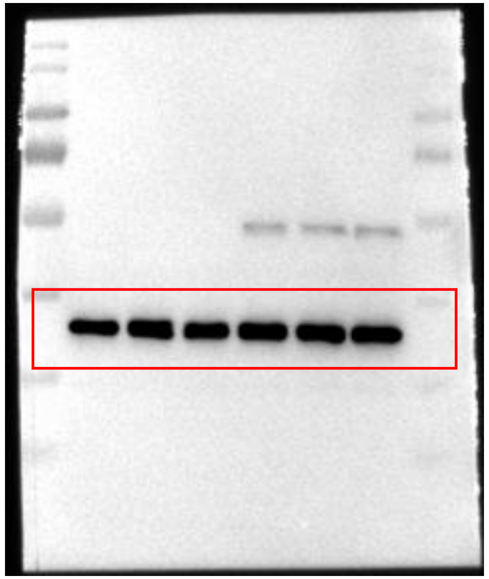

Supplement: Supplementary file 5 — Source Data for Expanded View and Appendix [file EMMM-15-e16581-s003.zip › EMM-2022-16581_EV_Figure_Source_Data.zip!/EMM-2022-16581-V3-Figure_EV4_Source_Data-sd.zip!/Figure EV4/4K/western gel.pdf]
